# Genome wide association joint analysis reveals 99 risk loci for pain susceptibility and pleiotropic relationships with psychiatric, metabolic, and immunological traits

**DOI:** 10.1371/journal.pgen.1010977

**Published:** 2023-10-16

**Authors:** Evelina Mocci, Kathryn Ward, James A. Perry, Angela Starkweather, Laura S. Stone, Siobhan M. Schabrun, Cynthia Renn, Susan G. Dorsey, Seth A. Ament

**Affiliations:** 1 Department of Pain & Translational Symptom Science, University of Maryland School of Nursing, Baltimore, Maryland, United States of America; 2 Center to Advance Chronic Pain Research (CACPR), University of Maryland Baltimore, Baltimore, Maryland, United States of America; 3 Institute for Genome Sciences, University of Maryland School of Medicine, Baltimore, Maryland, United States of America; 4 Department of Medicine, University of Maryland School of Medicine, Baltimore, Maryland, United States of America; 5 Department of Biobehavioral Nursing Science, University of Florida College of Nursing, Gainesville, Florida, United States of America; 6 Department of Anesthesiology, University of Minnesota Medical School, Minneapolis, Minnesota, United States of America; 7 School of Physical Therapy, University of Western Ontario, London, Ontario, Canada; 8 Department of Psychiatry, University of Maryland School of Medicine, Baltimore, Maryland, United States of America; Newcastle University, UNITED KINGDOM

## Abstract

Chronic pain is at epidemic proportions in the United States, represents a significant burden on our public health system, and is coincident with a growing opioid crisis. While numerous genome-wide association studies have been reported for specific pain-related traits, many of these studies were underpowered, and the genetic relationship among these traits remains poorly understood. Here, we conducted a joint analysis of genome-wide association study summary statistics from seventeen pain susceptibility traits in the UK Biobank. This analysis revealed 99 genome-wide significant risk loci, 65 of which overlap loci identified in earlier studies. The remaining 34 loci are novel. We applied leave-one-trait-out meta-analyses to evaluate the influence of each trait on the joint analysis, which suggested that loci fall into four categories: loci associated with nearly all pain-related traits; loci primarily associated with a single trait; loci associated with multiple forms of skeletomuscular pain; and loci associated with headache-related pain. Overall, 664 genes were mapped to the 99 loci by genomic proximity, eQTLs, and chromatin interaction and ~15% of these genes showed differential expression in individuals with acute or chronic pain compared to healthy controls. Risk loci were enriched for genes involved in neurological and inflammatory pathways. Genetic correlation and two-sample Mendelian randomization indicated that psychiatric, metabolic, and immunological traits mediate some of these effects.

## Author summary

Genome-wide association studies (GWASs) of pain-related traits have begun to elucidate genetic risk factors for susceptibility to chronic pain. Here we introduce a meta-analysis of 17 pain-related GWASs from the UK Biobank, selected for their significant heritability and genetic correlation. This approach aims to increase the statistical power to identify and interpret pleiotropic loci. Overall, we detected 99 genomic-wide significant risk loci, of which 65 validate previous findings from pain-related GWASs, while 34 are novel. Leave-one-trait-out meta-analyses, together with MTAG and replication analyses, suggest that the 99 loci range from those that primarily influence a specific pain-related trait to those linked to headache or muscular pain. Fewer loci had shared effects across most or all forms of pain. The pain risk loci identified in this study showed high pleiotropy and significant genetic correlation with psychiatric, metabolic, cardiovascular, and immune/inflammatory traits allowing us to generate hypotheses on the biological mechanisms triggered by pain.

## Introduction

Chronic pain is a public health epidemic, costing more than $600 billion in healthcare and lost work wages [1]. There are substantial individual differences in pain susceptibility, with patients who have seemingly identical injuries often reporting different pain intensities [2–4], likely explained by genetic factors [5,6]. Broad-sense heritability estimates ascribe up to ~50% of pain susceptibility to genetic factors [7–11]. Characterizing the genomic mechanisms that contribute to these individual differences in pain susceptibility may lead to genetic biomarkers to predict outcomes in patients, as well as to the discovery of targets for the development of novel therapeutics.

Genome-wide association studies (GWASs) have identified risk loci for several pain-related phenotypes, including migraine [12–16], headache [17], osteoarthritis [18,19], low back pain [20,21], neck or shoulder pain [22], knee pain [23], and multisite chronic pain [24–26]. Risk loci for the same (or similar) pain phenotypes are reproducible across cohorts, as best established for migraine and related headache phenotypes [12–17]. Other loci have been detected consistently across several forms of musculoskeletal pain such as osteoarthritis, back, neck or shoulder pain GWASs [18–22]. However, the extent of pleiotropy for many risk loci remains unclear. Correlating genetic risk factors with pain phenotype could assist with identification of the biological mechanisms activated in the complex pain process, as well as differentiate between those that act at a systemic level and those that are activated in a specific tissue.

Here, we describe a joint GWAS analysis of 17 pain-related traits from the UK Biobank. The goals of our joint analysis were two-fold. First, joint analysis can maximize statistical power to detect risk loci across genetically correlated traits. Second, joint analysis can reveal genetic pleiotropy among pain-related traits. The traits selected, other than having significant heritability, covered a broad spectrum of the pain phenotypes, from the body site to the duration and intensity, as well as pain secondary to other pathologies and commonly used pain relief medications as proxies. Our study identified 99 genomic regions associated with pain, of which 65 validate previous findings and 34 are novel. We performed statistical fine-mapping to identify potential risk genes at these loci and their enriched functional categories, as well as analyses of genetic correlations and Mendelian randomization to gain insight into pleiotropic relationships among pain susceptibility and other phenotypes.

## Results

### Discovery of 99 risk loci for pain susceptibility in the UK Biobank

**Fig 1:**
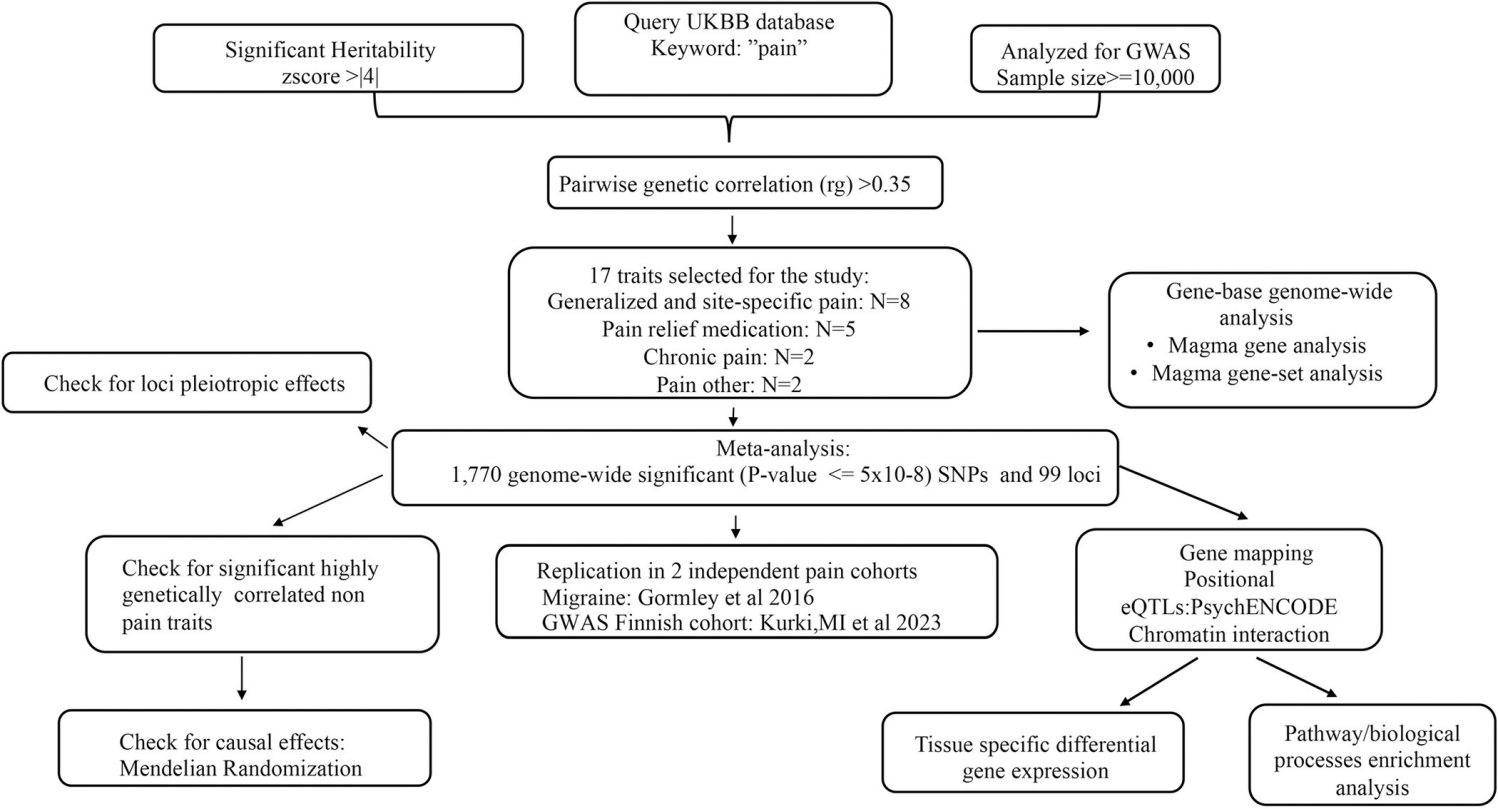
Study overview. A joint GWAS analysis was performed across 17 pain-related traits, followed by post-GWAS analyses to 1. annotate the risk loci and 2. explore the genetic relationships between pain susceptibility and non-pain traits that show high genetic correlation and pleiotropy with pain.

**Fig 2:**
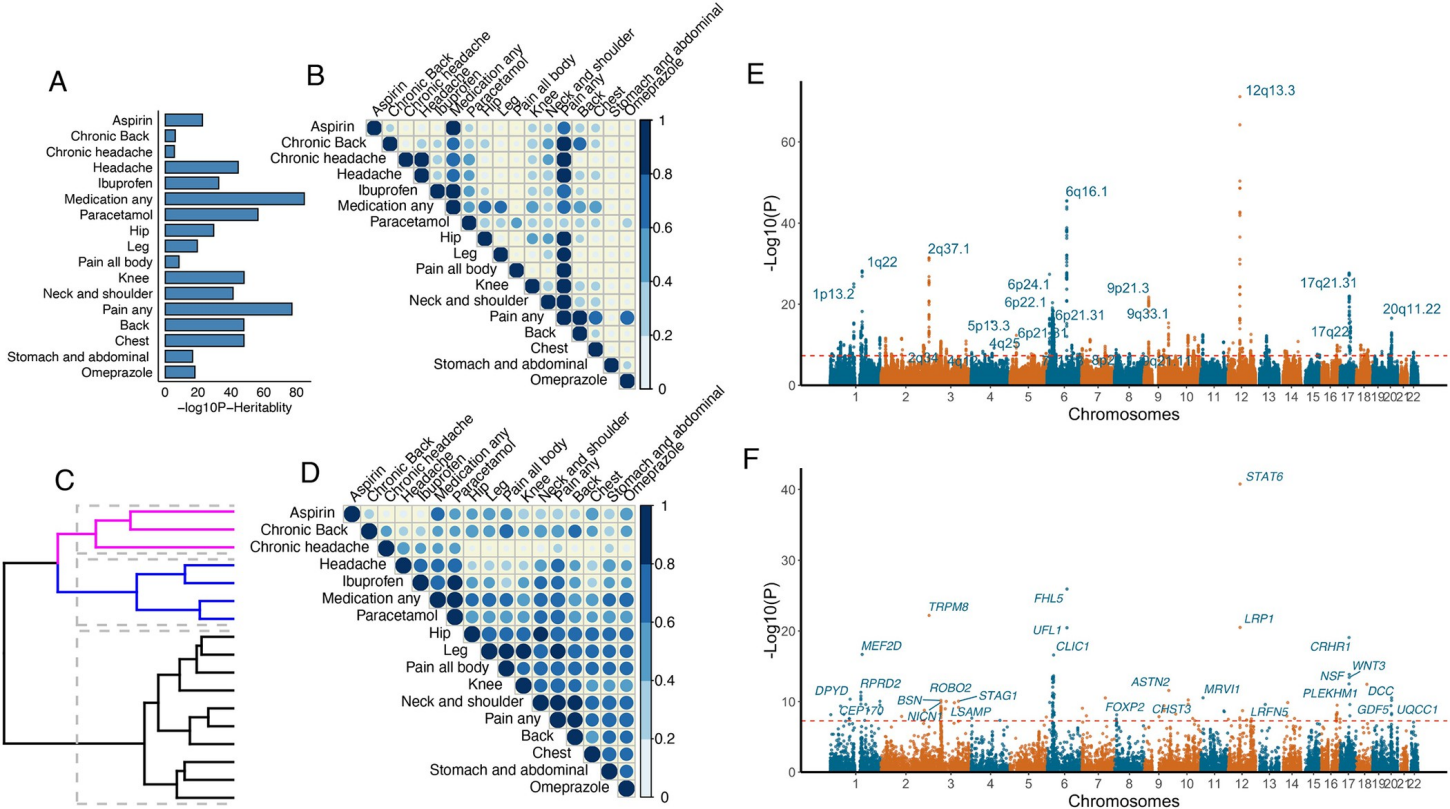
Joint GWAS analysis of 17 pain-related traits. **A**. SNP heritability estimates for 17 pain-related traits in the UK Biobank. Y axis indicates the trait, and the X-axis indicates the -log_10_(p-value) of the heritability estimate as calculated with LD Score Regression. **B.** Proportion of cases overlapping among the 17 traits. **C.** Dendrogram of the pairwise genetic correlation among the 17 UKBB traits showed three main clusters: cluster1 in pink (Aspirin, Chronic headache and back pain), cluster2 in blue (headache and pain medications), cluster3 in black (pain all body and pain localized to hip, leg, chest, neck. shoulder, back, and stomach/abdominal). Distance between clusters was measured as Euclidean distance. **D.** Pairwise genetic correlation estimates between all 17 pain related traits. blue = positive genetic correlation. Shade of blue and size of the circles are proportional to the coefficient of genetic correlation, larger and darker circles indicate higher genetic correlations. **E**. Manhattan plot derived from joint analysis of the 17 traits with PLEIO. The x-axis indicates chromosomal position, and the y-axis indicates -log_10_(p-value). **F.** Manhattan plot displaying the results of MAGMA gene-based analysis conducted from PLEIO 17 traits summary statistics.

The UK Biobank (n ~500,000) obtained numerous self-reported traits relevant to pain [27]. We selected 17 traits for joint GWAS analysis based on their relevance to pain phenotypes and substantial heritability (**Figs 1 and 2A, and Tables 1**, **A, and B in S1 Data and S1 Text).** Eight phenotypes describe types of pain experienced in the past month. Two phenotypes describe the emergence of chronic pain lasting at least three months, compared to individuals who experienced pain that resolved in less time. Five phenotypes related to the use of pain relief medications as proxies of experiencing pain. The remaining two phenotypes related to cardiovascular pain (chest pain and leg pain on walking) (**Table 1)**.

**Table 1:** UKBB pain related studies used in the meta-analysis.

Phenotype	N Controls	N Cases
Chest pain or discomfort	301274	56233
Back pain for 3+ months*	28500^	62055
Headaches for 3+ months*	38106^	32075
Leg pain on walking	92908	25997
Aspirin**	306880	50204
Ibuprofen (e.g. Nurofen) **	304035	53049
Paracetamol**	279148	77936
Omeprazole (e.g. Zanprol) **	335620	21464
Medication any**	159041	198043
Headache***	288719	71672
Neck or shoulder pain***	278271	82120
Back pain***	269042	91349
Stomach or abdominal pain***	330085	30306
Hip pain***	319891	40500
Knee pain***	283763	76628
Pain all over the body***	354861	5530
Pain any***	214877	145514

Notes: * Pain that lasted for 3 months or longer

** Medication for pain relief, constipation, heartburn

*** Pain type(s) experienced in last month

^ Controls are samples with acute pain defined as pain experienced during the last month.

We examined the extent to which individual participants reported multiple types of pain and the extent of case overlap among the 17 traits (**Figs 2B and S1**). ‘Pain any’ and ‘medication any’ represent the unions of the individual pain and medication use traits, respectively. Chronic headache and chronic back pain cases represent perfect subsets of the participants who reported those types of pain for any duration. The trait ‘pain all body’ had no overlapping cases with pain reported in specific body locations, as participants reporting this category were automatically excluded from the selection of pain traits at specific body locations as part of the UKBB survey. Across the remaining traits, we found an average of ~20% case overlap among pairs of traits (**Fig 2B**).

We calculated relative risk as a measure of comorbidity and identified relationships among specific pairs of pain-related traits (**S2 Fig**). Many individuals that experienced leg pain also reported hip pain (RR = 4.26, 95%CI = 4.13–4.40) and knee pain (RR = 3.63, 95%CI3.56–3.71) (**S2 Fig**). Participants who reported ibuprofen use often experienced headache (RR = 2.13, 95%CI 2.10–2.16), and similar trends were observed for omeprazol and stomach/abdominal pain (RR = 2. 73, 95%CI 2.65–2.81), paracetamol and headeache (RR = 2.75, 95%CI 2.72–2.79), and aspirin and chest pain (RR = 1.76, 95%CI 1.73–1.79) (**S3 Fig**). Overall, these results describe the complex and phenotypically heterogeneous presentation of pain susceptibility in the UKBB sample.

Next, we considered the extent to which risk for pain-related traits is genetically correlated. We found strong genetic correlations among most pain-related traits (**Fig 2C and Table C in S1 Data**). This analysis also provided evidence for clusters of traits with especially strong genetic correlations, including headache-related traits and muscular pain-related traits (**Fig 2C and Table C in S1 Data**). Except for a small number of traits with a high proportion of overlapping cases (‘pain any’, ‘medication any’, ‘chronic headache’ and ‘chronic back pain’), there was no evidence for inflation of genetic correlation estimates due to sample overlap (**Fig 2D**). Taken together, these analyses suggest a shared genetic component for susceptibility to many forms of pain that are captured by traits in the UK Biobank. It is notable that the genetic correlations are much stronger than the extent of case overlap, suggesting that shared genetic factors may manifest in heterogeneous presentations of pain across individuals.

**Fig 3:**
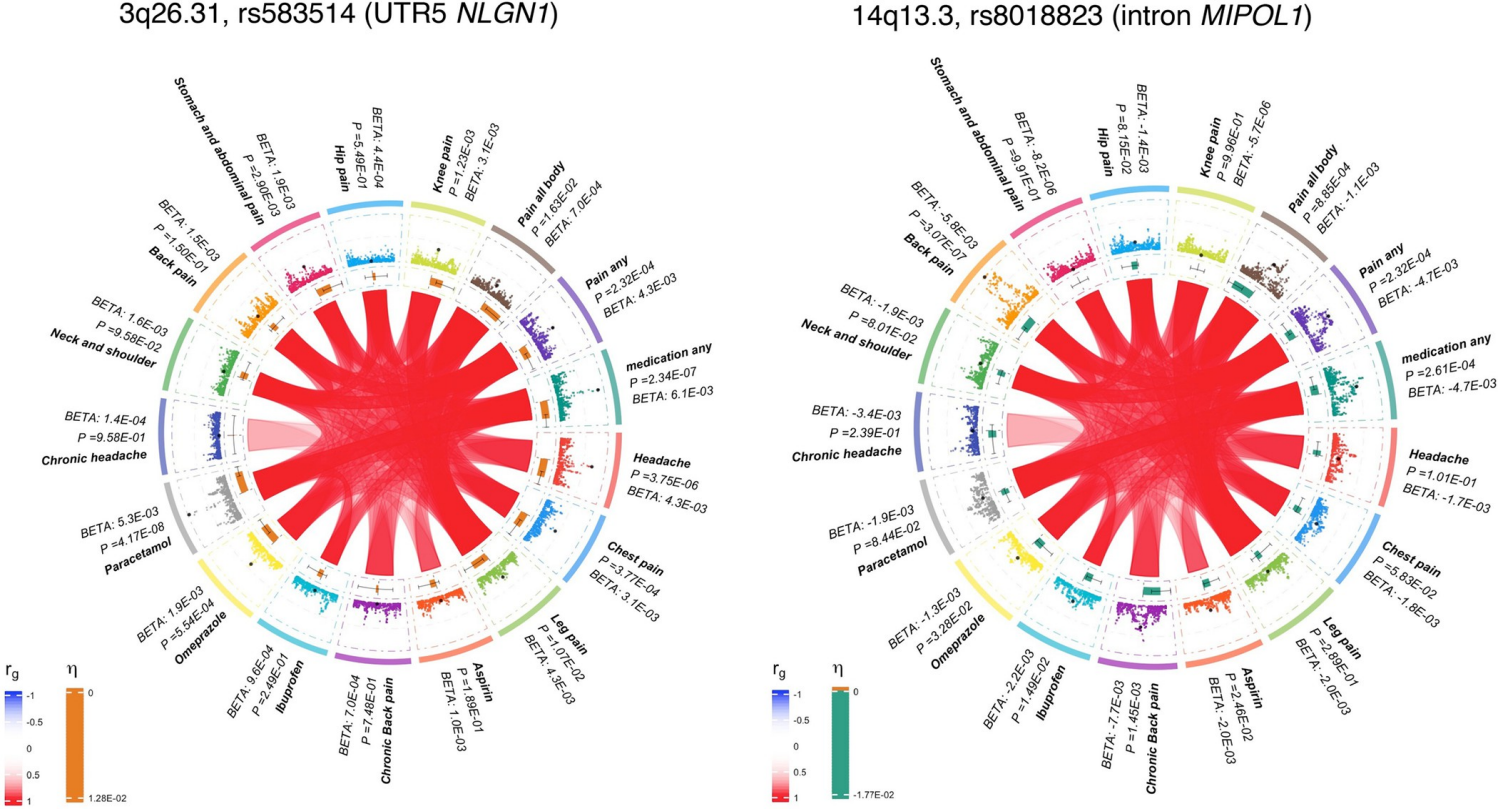
Circoplots of two representative loci. Region plots for two novel pain risk loci as representative of those associated with multiple pain traits (A) vs. those with greater trait specificity (B). A. Locus associated with both Headache/Migraine and Muscular pain centered on chromosome 3q26.31 at the UTR5 of *NLGN1*, encoding neuroligin-1. B. Risk locus for Back pain centered on chromosome 14q13.3 in an intron of *MIPOL1*, encoding mirror-image polydactyly 1.

We conducted a joint analysis of the 17 pain-related traits with PLEIO [28] to elucidate this shared genetic architecture and boost statistical power to identify specific risk loci. This analysis identified 1,770 genome-wide significant pain-associated SNPs (*P* < 5e-8) at 99 approximately linkage-disequilibrium (LD) independent loci (**Figs 2E, S4 and S5 and Tables D and E in S1 Data**). 65 of these 99 risk loci overlap loci from previous GWAS of pain-related traits [12–25] **(Table F in S1 Data)**, while the remaining 34 loci are novel (**Tables 2 and G in S1 Data)**. Notably, at 51 of the risk loci from our joint analysis, none of the underlying GWASs of individual traits reached genome-wide significance (**Table H in S1 Data**), demonstrating that the joint analysis resulted in a substantial increase in statistical power compared to GWAS of individual pain-related traits. We provide region plots to illustrate the genetic architecture of all 17 traits at each locus (**Figs 3 and S4)**. As expected, most loci appear to have associations in these regions for many of the individual traits, and we further explore these relationships below. We also conducted sex-stratified GWAS meta-analyses, which identified 16 and 27 genome-wide significant loci in males and females, respectively (**S7 and S8 Figs)**. Two loci, 1q24.2 (intergenic) and 10p13 (*CAMK1D*) reached genome-wide significance only in women, and two loci, 6q22.32 (*CENPW*) and, 15q26.1 (*FES*), were genome-wide significant only in men. At most risk loci, both male and female populations contributed to the signal (**Table I in S1 Data).**

**Table 2:** Novel pain risk loci.

cytoBand	Replication	SNP	pos	effect allele	Non effect allele	eaf	beta*	se	Pvalue	nearest gene	nSNPs	sSNPs
1p36.23		rs301805	8481016	G	T	0.59	0.0135	0.0024	1.56E-08	*RERE*	142	24
1p31.3	**β**	rs1325266	67160602	G	A	0.40	0.0156	0.0024	8.94E-11	*SGIP1*	49	9
1p22.1		rs10782959	93811569	G	A	0.73	0.0147	0.0026	2.61E-08	*RP4-717I23*.*3*:*DR1*	296	40
1p21.3	**β**	rs1198583	98529378	G	A	0.83	0.0199	0.0031	1.67E-10	*NFU1P2*	277	60
1p13.3	**β**	rs583104	109821307	T	G	0.77	0.0161	0.0028	1.09E-08	*PSRC1*	12	7
1q21.3	**β**	rs9426902	153718746	A	G	0.43	0.0130	0.0024	4.75E-08	*INTS3*	19	8
2p24.1	**δ**	rs1876767	22600932	T	G	0.38	0.0170	0.0024	2.32E-12	*AC068490*.*2*:*AC096570*.*2*	250	47
2p23.3		rs12612492	24093756	T	C	0.13	0.0197	0.0035	1.58E-08	*ATAD2B*	392	29
2p16.3	**β**	rs3914722	50991039	A	G	0.11	0.0212	0.0038	1.83E-08	*NRXN1*	66	8
2q12.1	**β**	rs17343925	104426377	T	G	0.45	0.0142	0.0024	1.74E-09	*AC013727*.*1*	132	12
2q33.3	**δ**	rs2111592	208049581	A	G	0.31	0.0164	0.0025	1.25E-10	*AC007879*.*1*	78	18
2q34	**β**	rs17822981	211355495	G	A	0.25	0.0170	0.0027	5.23E-10	*CPS1*	220	32
3p12.3	**β**	rs775760	77586597	C	A	0.70	0.0157	0.0026	1.19E-09	*ROBO2*	76	12
3q26.31	**δ**	rs583514	173114167	C	T	0.51	0.0160	0.0024	1.26E-11	*NLGN1*	19	6
4p16.3	**β**	rs3021146	2958186	G	A	0.33	0.0145	0.0025	7.86E-09	*NOP14-AS1*:*NOP14*	152	34
4q13.2	**β**	rs4860809	67870395	C	T	0.58	0.0133	0.0024	2.43E-08	*RNU6-699P*	135	12
4q25	**β**	rs11729080	112503872	A	G	0.17	0.0192	0.0031	6.34E-10	*RP11-255I10*.*1*	8	1
5q22.3	**β**	rs554843	113654597	A	C	0.41	0.0133	0.0024	2.91E-08	*KCNN2*	3	1
6q22.33	**β**	rs4895846	129384367	C	T	0.58	0.0137	0.0024	9.18E-09	*LAMA2*	34	6
6q25.3		rs3918286	160868668	G	A	0.39	0.0135	0.0024	2.04E-08	*SLC22A3*	23	11
7p22.3	**δ**	rs11764590	2032803	T	C	0.18	0.0186	0.0030	8.69E-10	*MAD1L1*	148	15
8p21.3	**β**	rs1552284	21962607	G	A	0.48	0.0130	0.0024	3.33E-08	*FAM160B2*	26	9
8q13.3	**β**	rs3863241	73890335	T	C	0.53	0.0134	0.0024	1.49E-08	*RP11-531A24*.*3*	42	9
12p13.33	**β**	rs16931821	192990	C	T	0.60	0.0138	0.0024	9.71E-09	*IQSEC3*	33	9
12q13.2	**β**	rs773108	56369911	G	A	0.34	0.0141	0.0025	1.55E-08	*RAB5B*	49	14
12q24.12	**β**	rs10774625	111910219	G	A	0.50	0.0172	0.0024	2.70E-13	*ATXN2*	33	8
13q21.32	**β**	rs1413573	66951829	A	G	0.48	0.0142	0.0024	1.98E-09	*PCDH9*	40	13
14q12	**β**	rs1951537	29560821	G	A	0.86	0.0200	0.0034	2.92E-09	*RP11-148E17*.*1*	70	14
14q13.3	**β**	rs8018823	37718177	A	G	0.29	0.0153	0.0026	3.98E-09	*MIPOL1*	397	72
16q22.2		rs8057124	71972409	C	T	0.74	0.0149	0.0027	2.90E-08	*PKD1L3*	115	14
18q12.2	**δ**	rs11665070	35152563	A	G	0.67	0.0150	0.0025	2.05E-09	*CELF4*	49	12
18q21.2	**δ**	rs618869	53248151	C	T	0.86	0.0184	0.0034	4.14E-08	*TCF4*	30	12
19q13.32	**ε**	rs4420638	45422946	G	A	0.19	0.0176	0.0030	4.18E-09	*APOC1*	29	4
22q12.2		rs16988333	30552813	G	A	0.09	0.0235	0.0040	6.74E-09	*HORMAD2*	188	19

Notes and abbreviations: pos: SNP position (GRCh37); eaf: effect allele frequency; beta = effect size, computed for the effect allele; se: standard error of beta; nSNPs = number of SNPs in linkage disequilibrium with the lead SNP at the locus; sSNPs = number of genome-wide significant SNPs located within the locus; β = loci found genome-wide significant at MTAG analysis; δ = loci found genome-wide significant at MTAG analysis and validated with multiple test adjusted P-value (P = 0.0005) in the Finnish pain cohort; ε = loci validated with multiple test adjusted P-value (P = 0.0005) in the Finnish pain cohort.

We conducted four analyses to validate our primary GWAS results. First, while PLEIO is explicitly designed to produce valid p-values in the presence of sample overlap across traits, as in our analysis, there is a need to check for p-value inflation. As expected, the distribution of p-values corresponds to a substantial deviation from a null distribution (**S6 Fig**). We used Linkage Disequilibrium Score regression (LDSC) to estimate the proportion of this deviation from a null distribution that could be attributed to true polygenicity vs. confounding due to model misspecification. The LDSC intercept term was below 1 (0.87), suggesting that our model may be somewhat conservative but has little p-value inflation. The LDSC ratio statistic was 0.01, indicating that ~99% of deviation from a null distribution can be explained by polygenicity and only ~1% by inflation of test statistics. In addition, based on LDSC, we estimate that ~13% of trait variance (*h*^*2*^) was explained by the genotyped and imputed SNPs.

Second, we compared the loci discovered in our primary analysis to a joint analysis of the same 17 pain-related traits using an alternative tool, Multi-Trait Analysis of GWAS (MTAG). Like PLEIO, MTAG is designed to adjust for sample overlap, relatedness, and population stratification. Unlike PLEIO, which combines the GWAS statistics of the jointly-analyzed traits into a single final statistic, MTAG produces a separate set of “boosted” summary statistics for each trait analyzed. In our MTAG analysis, 83 of the 99 loci detected with PLEIO were genome-wide significant in at least one of the 17 traits (**Table J in S1 Data**). Of note, 27 of the 34 novel pain loci identified by PLEIO also reached genome-wide significance in the MTAG analysis Therefore, most of the loci we identified are robust to the joint analysis methodology.

Third, we evaluated the robustness of our results when removing a subset of the traits from the joint analysis. Specifically, we conducted a joint analysis of 13 traits, leaving out the four traits with the highest proportion of overlapping cases (‘pain any’, ‘medication any’, ‘chronic headache’ and ‘chronic back pain’). This analysis had somewhat reduced statistical power, but nonetheless identified 75 out the 99 loci as genome-wide significant (P<5e-08) (**Table K in S1 Data**). The LDSC intercept (0.84) and ratio (0.012) is this analysis were similar to those obtained in the joint analysis of all 17 traits, suggesting that PLEIO correctly controlled for sample overlap in both analyses.

Fourth, we performed replication analyses in independent GWAS of pain-related traits, utilizing summary statistics both from a GWAS of migraine in 29,209 European ancestry cases and 172,931 controls [13], as well as a GWAS of limb, back, neck, head and abdominal pain in 151,484 cases and 190,313 controls from Finland [29] (**Table L in S1 Data**). Seventeen of the 99 loci were associated with migraine at a replication threshold, *P* < 0.0005 (0.05 / 99). 18 loci were replicated in the Finnish pain GWAS study (P<0.0005). Of note, there was no overlap between the loci replicated in the two replication samples, so a total of 35 loci could be replicated in independent samples (**Table 2**). Overall, our validation analyses support the robustness of our joint analysis.

### Pleiotropic associations of risk loci among pain-related traits

The genetic correlations among pain-related traits, as well as the success of our joint GWAS analysis in boosting statistical power, suggested that many risk loci influence multiple pain-related traits. To further explore these relationships, we conducted “leave-one-trait-out” joint analyses. That is, we used PLEIO to conduct 16-trait joint analyses, leaving out each of the 17 traits in turn, and we examined the changes in p-values to gain insight into the influence of each trait on our results. Thirty-seven of the 99 loci reached genome-wide significance in all leave-one-trait-out analyses (**Table L in S1 Data**), including many of those with the strongest joint p-values, e.g., 12q13.3 (*LRP1*, *STAT6*), 1q22 (*MEF2D*), and 17q21.31 (*CRHR1*). 22 risk loci were mostly driven by a single trait, including 12p12.1 (*SOX5*) specific for back pain, 20q11.22 (*GDF5*) and 7p14.1 (*SUGCT*) (**Table M in S1 Data**). The remaining loci had intermediate levels of sensitivity, suggesting they influence some but not all of the pain-related traits.

**Fig 4:**
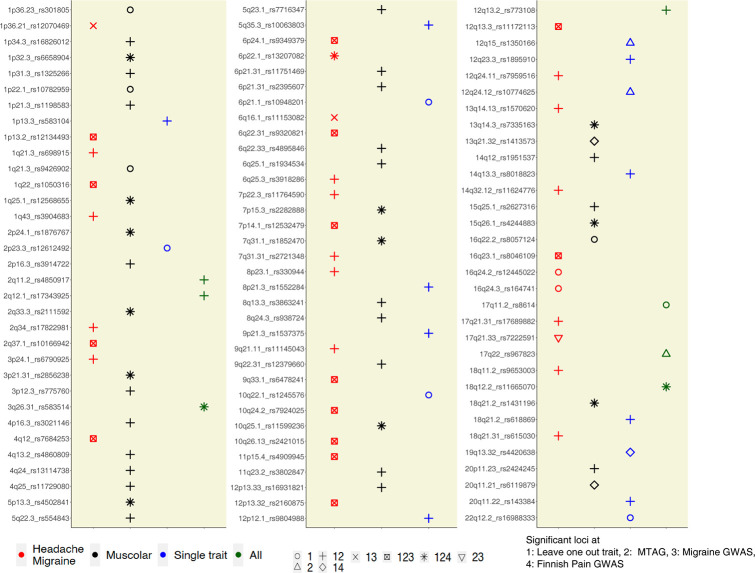
Summary of results from sensitivity, validation, and replication analyses. We grouped the 99 loci into four categories corresponding to the extent of pleiotropy across pain-related traits. **“**All” indicates loci with very broad pleiotropy across pain-related phenotypes; “Headache/Migraine” and “Muscular” loci had pleiotropic effects on multiple traits within one of those broad categories; “Single trait” loci were primarily associated with a single trait. These annotations are based on the results of four analyses: 1. Leave one out sensitivity analysis with PLEIO, 2. technical validation with MTAG, 3. Replication in an independent migraine GWAS, 4. Replication in an independent musculoskeletal pain GWAS. Symbols indicate which of the four analyses support the category for each locus, including six possible combinations of significance in the four analyses (1,1+2,1+3,1+2+3,1+2+4,1+2+3+4).

We further quantified the importance of each trait to the associations at each locus by calculating the change in the -log10(p-value) in each leave-one-out analysis compared to the primary GWAS. Headache was the most influential trait for many loci, followed by chronic headache, knee pain, and back pain (**S9 Fig**). We noticed that many of the loci were associated with similar combination of traits, providing evidence for two genetically distinct categories: Headache/Migraine versus Muscular pain (**Table M in S1 Data)**. To build further evidence for these patterns, we compiled the locus-specific results from all of the validation and replication analyses into a single figure (**Fig 4**). Four categories of loci emerge from this analysis: seven “all trait” loci with a very high rate of pleiotropy across the 17 traits; 16 “single-trait” loci that are primarily associated with a single pain-related trait; 41 “muscular pain” loci that are associated with several forms of skeletomuscular pain (e.g., pain in multiple sites across the body); and 35 “headache” loci that are more strongly associated with headache than other forms of pain. The genetic distinction between headache and muscular pain at specific loci is consistent with the patterns of genome-wide genetic correlation (**Fig 2C**). Moreover, we predominantly replicated “headache” loci in the headache replication cohort, whereas we predominantly replicated “muscular pain” loci in the Finnish body pain replication cohort (**Table L in S1 Data and Fig 4**). Thus, the potential distinction between headache and muscular pain may explain why we replicated non-overlapping sets of loci in the two replication cohorts. The potential distinctions between headache and other types of pain merit further investigation.

### Pain susceptibility risk loci are enriched for genes with neurological and immunological functions

We functionally annotated the risk-associated variants, genes, and gene sets from our joint analysis to gain biological insights into pain susceptibility. First, we predicted the genes impacted by the SNPs at each risk locus based on proximity to risk-associated SNPs, as well as by integration with expression quantitative trait loci (eQTLs) and chromatin interaction data from the human cortex [30]. This analysis identified a total of 664 genes at the 99 risk loci (**Table N in S1 Data and S10 Fig)**. In parallel, we performed gene-based analyses with MAGMA [31], which aggregates the associations of multiple SNPs in the vicinity of each gene. MAGMA identified 300 pain-associated genes at a genome-wide significance threshold, *P* < 2.8x10-6, adjusting for 17,620 protein-coding genes (**Fig 2F and Table O in S1 Data**). Of these, 46 were the closest gene to an LD-independent lead SNP at one of the 99 genome-wide significant risk loci (**Table P in S1 Data)**, while the remaining genes were distal to these SNPs or located outside the genome-wide significant risk loci.

To facilitate the identification of causal genes within the risk loci, we checked whether the 664 genes showed differential expression (DE) in individuals with acute or chronic pain compared to healthy participants (HP). We found that 99 genes–significantly more than expected by chance (Fisher’s exact test: *P* = 1.2e-7)–overlapped the bounds of 41 risk loci were up or downregulated in subjects with acute or chronic pain compared to HP (**Table Q in S1 Data**). 50% of these genes were predicted as potentially causal by one or more gene mapping methods (**Table N in S1 Data)**.

We tested for functional enrichments of the genes at pain susceptibility loci using MAGMA. First, we tested for overlap with genes that have been previously shown to influence pain susceptibility. We found that established pain-related genes both from clinical studies in humans [32] (*P* = 1.65e-4**)** and functional experiments in mice [33] (*P* = 2.0e-2**)** were enriched at pain susceptibility loci (**Table R in S1 Data)**. Genes with prior evidence from functional studies had diverse molecular functions. For instance, *TRPM8* (*P* = 6.4e-23) encodes a cold-sensing cation channel. *LRP1* (*P* = 3.1e-21) encodes low-density lipoprotein-related protein 1. *NCAM1* (*P* = 2.0e-9) encodes neural cell adhesion molecule 1. Amongst genes at previously unreported risk loci, *SGIP1* (*P* = 6.3e-9) encodes Src homology 3-domain growth factor receptor-bound 2-like endophilin interacting protein 1, which interacts with cannabinoid CB1 receptors and has been shown to modulate nociception in mice [34]. *ROBO2* (*P* = 8.9e-11) encodes roundabout guidance receptor 2, a cell adhesion molecule that has been implicated in pain via its roles in the development of sensory ganglia, required for the sensation of touch, taste and pain [35]. *NLGN1* (*P* = 7.8e-4) encodes neuroligin 1, yet another cell adhesion molecule, which has been implicated in inflammatory pain via its activity-dependent synaptic recruitment within neurons of the spinal dorsal horn [36]. There is a need for additional studies in animal models to elucidate such mechanisms, especially for genes at novel risk loci.

Next, we conducted unbiased enrichment analyses for genes with tissue-specific gene expression and in functional categories. Tissue-specificity analysis identified enrichments (False Discovery Rate [FDR] < 0.05) for genes expressed in the cerebellum (**Table S in S1 Data and S11 Fig)**. Analyses of functional categories identified significant (FDR < 0.05) enrichments for gene sets related to neuronal development and function (e.g., neurogenesis, un-adjusted *P* = 3.2e-08, neuron differentiation, *P* = 1.4e-06, post synapse, *P* = 4.6e-07; FDR-adjusted q-values shown in **Table T in S1 Data**). Top pain-associated neurogenesis genes included *LRP1* (*P* = 3.1e-21), *UFL1* (*P* = 3.7e-14), *WNT3* (*P* = 1.7e-13), *SLC44A4* (*P* = 3.5e-13), *DCC* (*P* = 6.4e-11) and *GDF5* (*P* = 1.2e-10) (**Table U in S1 Data**). Additionally, we found significant enrichment (FDR < 0.05) in categories related to immunological functions, e.g., chemokine secretion (*P* = 1.41e-4), T-helper T-cell differentiation (*P* = 1.3e-04) and Reactome T-cell receptor (TCR) signaling, P = 1.1e-04). Top pain-associated genes with immunological functions included *STAT6 (P = 1*.*7e-41)* and *CRHR1 (P = 8*.*4e-20)* (**Table O in S1 Data)**. These results support dual neurological and immunological etiology for pain susceptibility.

### Genetic effects in pain are mediated by neuropsychiatric, immunological, cardiometabolic and respiratory traits

**Fig 5:**
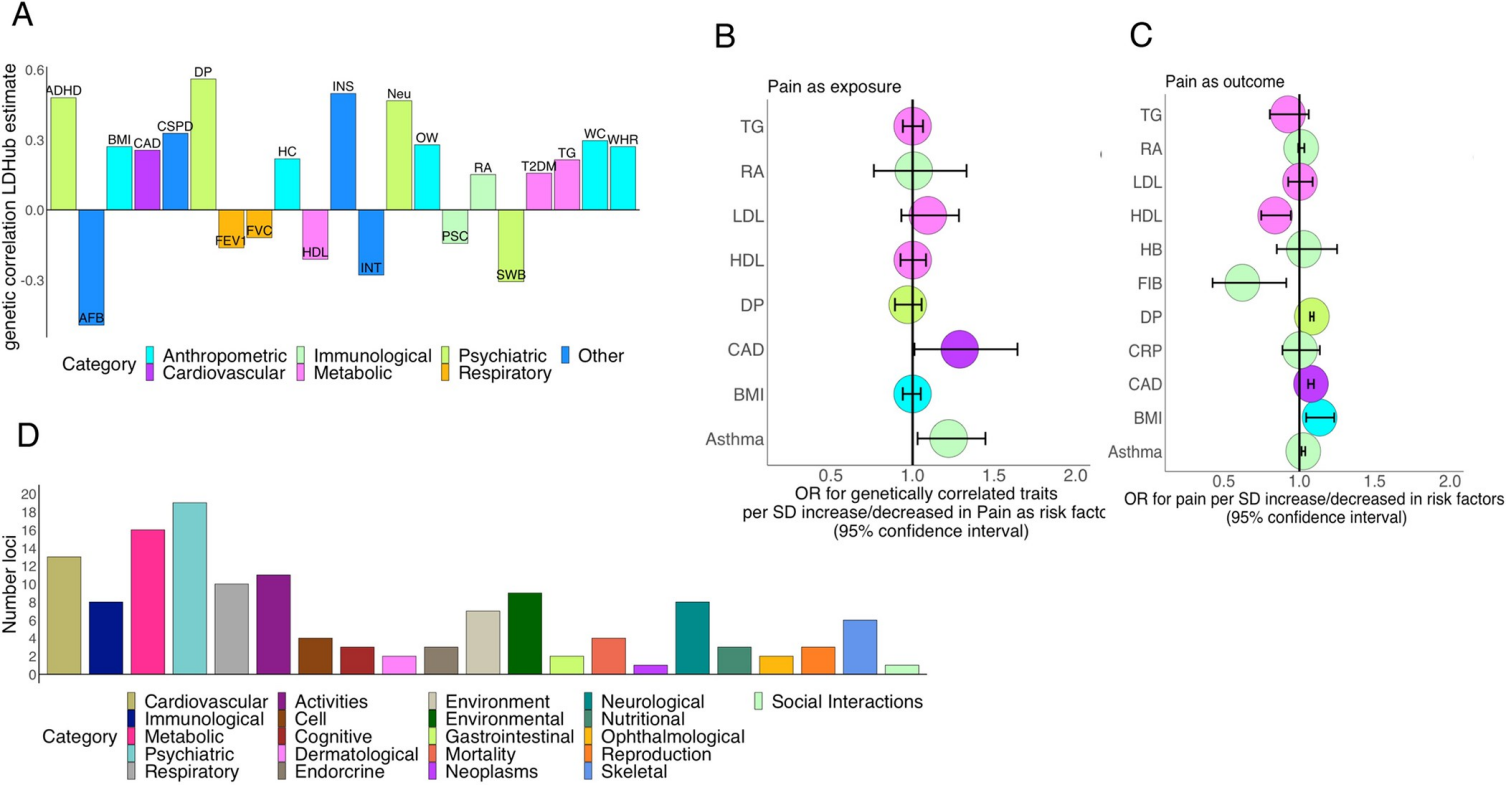
Genetic correlation and predicted causal interactions between pain susceptibility and non-pain traits. **A**. Genetic correlations between pain and non-pain traits. Traits are grouped by domains indicated by bar color. **B.** Two-sample Mendelian Randomization (MR) results with pain as exposure. **C.** Two-sample MR results with pain as outcome. For B and C, the X axis indicates the odds ratio (OR) for non-pain traits and its 95% confidence interval (CI). **D.** Number of pain risk loci showing pleiotropic effects with non-pain traits in the GWAS Atlas, grouped by trait category. ADHD: Attention deficit hyperactivity disorder, AFB: Age of first birth, BMI: Body max index, CAD: Cardiovascular disease, CRP: C-Reactive Protein, CSPD: Cigarettes smoked per day, DP: Depression, FEV1: Forced expiratory volume in 1 second, FIB: Fibrinogen, FVC: Forced vital capacity, HB: Hemoglobin, HC: Hip circumference, HDL: high density lipoprotein, INS: Insomnia, INT: Intelligence, Neu: Neuroticism, OW: Overweight, PSC: Primary sclerosing cholangitis, RA: Rheumatoid Arthritis, SWB: Subjective well-being, T2DM: Type 2 Diabetes, TG: triglycerides, WC: Waist circumference, WHR: Waist-to-hip ratio.

Pain often occurs in the context of other diseases or conditions. We investigated these relationships through analyses of genetic correlations and genetic mediation. First, pairwise genetic correlation analyses were performed between pain susceptibility and 254 diverse traits collected in LDHub [37]. We identified genetic correlations with 62 traits (**Fig 5A and Table V in S1 Data**). Many of the traits genetically correlated with pain susceptibility were neuropsychiatric, metabolic, cardiovascular, or immunological. These genetic correlations are consistent with established patterns of comorbidity [38–42] and suggest that genetic susceptibility to experiencing pain involves both brain-related and peripheral mechanisms.

To further explore these relationships, we tested for causal relationships between pain and non-pain traits using two-sample Mendelian randomization (MR) [43–45]. In MR analyses, genetic variants are used as instrumental variables to assess causal relationships between an exposure that may mediate an outcome. In our case, the propensity to experience pain could arise as a consequence of being exposed to various other heritable traits. In addition, the experience of pain may itself predispose to other outcomes. We focused on eight traits that showed significant genetic correlations with pain susceptibility and for which well-powered GWAS summary statistics were available from independent cohorts: BMI, depression, asthma, coronary heart disease, fibrinogen levels, C-reactive protein, hemoglobin, and rheumatoid arthritis. Below, we summarize the four main results from this analysis.

First, MR analysis suggested that exposure to depression increases the likelihood of reporting pain (MR-PRESSO OR = 1.08, 95% CI = 1.07–1.10, P = 3.12E-05) **(Table W in S1 Data and Figs 5B, 5C, and S12**). Nine SNPs were included in this analysis (F-statistic = 33) because of their highly significant association with depression (**Table X in S1 Data).** These results are consistent with previous MR analyses of pain [40,41]. Our analyses do not support the reverse effect, i.e., that exposure to pain increases risk for depression.

Second, we found evidence for causal relationships involving several cardiometabolic traits, including coronary artery disease, body mass index, and lipoprotein levels (Fig **5B and 5C**). The strongest effect in this category was that exposure to coronary heart disease increases the likelihood of reporting pain (IVW OR = 1.048, 95% CI = 1.005–1.092, P = 2.66E-02) **(Tables W and X in S1 Data and Figs 5C and S13**). Reverse MR analysis suggested that exposure to pain may also increase risk for coronary heart disease (IVW OR = 1.23, 95% CI = 1.01–1.50, P = 3,78 E-02) **(Table Y in S1 Data and Figs 5B and S14),** consistent with a recent report [42]. We carried out the Steiger (directional) test to assess whether the instrumental variants used in the bidirectional analysis of pain and coronary artery disease were primarily associated with the exposure or the outcome, and this test indicated that coronary artery disease had a causal effect on pain and not the inverse (**Tables W, Z, and AA in S1 Data**). Our analysis also suggested that exposure to elevated BMI modestly increases the likelihood of reporting pain (MR-PRESSO Odds Ratio (OR) 1.13, 95% CI = 1.04–1.23, p = 6.54E-03 per SD increase of BMI) **(Table Y in S1 Data and Figs 5C and S15)**. We tested for causal relationships of pain with LDL, HDL, and total cholesterol using multivariable Mendelian randomization [46,47], enabling us to assess independent effects of each lipoprortein despite their strong genetic correlations (LDL vs. total cholesterol (TC), rg ~ 1 TG vs. HDL, rg = -0.65; TG vs. LDL, rg = 0.382). These analyses suggested that exposure to higher levels of HDL reduced the likelihood of reporting pain (IVW OR 0.84, 95% CI = 0.74, 0.94, P = 3.16E-03 per SD increase in the HDL) **(Table BB in S1 Data and Fig 5C)**. We note, however, that the low value of the conditional F-statistic [41] (F<10) indicates that the SNPs included in the analysis (**Table CC in S1 Data),** had low power to detect a causal effect. Overall, these analyses suggest that exposure to a variety of cardiometabolic conditions mediates genetic risk for pain.

Third, we tested for causal effects of several immune and inflammation-related traits. We found that exposure to asthma increased the likelihood of reporting pain (MR-PRESSO, OR = 1.026, 95% CI = 1.014–1.039, P = 1.53E-02) **(Table Y in S1 Data and Figs 5C and S16**). We also found evidence for the inverse relationship, wherein exposure to pain increases risk for asthma, suggesting horizontal pleiotropy (IVW OR = 1.22, 95% CI = 1.03–1.45, P = 2.09E-02) **(Figs 5D and S17 and Table Y in S1 Data).** The Steiger test suggested that asthma had a causal effect on pain and not vice versa (**Table AA in S1 Data)**. None of the other tested immune and inflammation traits (C-reactive protein, fibrinogen levels, hemoglobin, and rheumatoid arthritis) showed evidence of a causal effect on pain **(Table X in S1 Data and Fig 5C)**.

### Pleiotropic effects of risk variants

Next, we analyzed the pleiotropic associations of individual SNPs on pain and non-pain traits. We started by examining the effects of pain-associated SNPs from our analysis on >4,000 non-pain traits collected in the GWAS Atlas [48]. Pain-associated SNPs from our analysis were significantly overrepresented among SNPs associated with any trait in the GWAS Atlas (Fisher’s exact test: odds ratio = 69.9; *P* < 1e-300, including multiple shared associations with neuropsychiatric, immunological, cardiovascular, and metabolic traits (**Fig 5D and S2 Data).** For instance, SNPs at 24 of the 99 pain susceptibility loci also had genome-wide significant effects on psychiatric or neurological traits. These analyses support that a substantial number of the associations with pain susceptibility involve shared associations with other traits. We note, however, that many of these associations are not fully independent of our pain GWAS, as many of the studies in the GWAS Atlas [48] were conducted using samples from the UK Biobank.

To characterize these pleiotropic effects in a fully independent sample, we examined non-UKBB GWASs of eight traits identified in the MR analysis as exposures mediating pain susceptibility: major depressive disorder [49], body mass index [50,51], coronary artery disease [52], asthma [53], and four lipoprotein traits (LDL, HDL, TC, and triglycerides) [54]. Lead SNPs or genetically correlated tag SNPs at 30 of the 99 pain-associated loci were pleiotropically associated with at least one of these traits (P < 5e-4, alpha = 0.05 after adjusting for 99 SNPs), including pleiotropic effects on asthma (N = 5), BMI (N = 5), depression (N = 5), coronary artery disease (N = 8), HDL (N = 9), LDL (N = 8), TG (N = 4), and TC (N = 10) (**Table DD in S1 Data**). Most SNPs had pleiotropic effects on a single mediating trait. These results demonstrate that the pleiotropic effects of these SNPs on non-pain traits are replicable outside the UK Biobank and provide evidence for diverse mediating effects, wherein the mediating effects of these traits are distinct and locus-specific. Notably, these pleiotropic effects outside the UK Biobank represent a form of replication for these 30 loci. Thus, in total, 68 of the 99 loci could be replicated at *P* < 5e-4 in independent cohorts with pain or genetically correlated traits.

## Discussion

A joint analytic approach enabled us to identify 99 genomic regions associated with pain, of which 34 have not been reported previously in the peer-reviewed literature. Gene set enrichment analysis, genetic correlation analysis, and genetic mediation analyses provided biological insights into the pathogenic mechanisms. Our results provide preliminary evidence that some of the genetic factors contributing to pain susceptibility are mediated by depression, obesity, asthma, and cardiovascular diseases.

Joint analysis boosted statistical power primarily because of the strong genetic correlations among diverse pain-related traits. Overall, our study supports that most pain-related traits are genetically correlated, and that most risk loci influence susceptibility to multiple types of pain. This is expected, given prior evidence for both phenotypic and genetic correlations among pain phenotypes [55], and suggests that the lack of overlap of loci reported in previous studies primarily reflected insufficient statistical power. Nonetheless, our data suggest that some SNPs have reproducible associations primarily with a specific type of pain. In particular, it appears that genetic risk for headache involved a number of loci that are distinct from musculoskeletal pain experienced in various parts of the body.

Neuronal genes and mediation by neuropsychiatric phenotypes appear to play a substantial role in genetic risk for pain. Gene set enrichment analyses revealed enrichments for genes expressed in several brain regions and for functional categories related to the development and functions of neurons. Specific genes at pain risk loci implicate mechanisms in both the central and peripheral nervous systems. For instance, *TRPM8* encodes a cold-sensitive cation channel expressed primarily in peripheral nerves [56]. Knockdown of *Trpm8* in mice demonstrated an essential role in the suppression of sensitized pain responses [57]. By contrast, *NCAM1*, encoding neuronal cell adhesion molecule 1, is expressed primarily in the central nervous system and is involved in cell adhesion and synaptogenesis. Cortically expressed NCAM1 has been shown to regulate synaptic reorganization after peripheral nerve injury [58]. Mendelian randomization suggested that the effects of many of these variants on pain may be mediated by their effects on major depression. Somatization, defined as the tendency of some individuals to experience and communicate somatic distress in response to psychosocial stress, occurs frequently in patients with major depression and related disorders, perhaps explaining these effects [59]. Chronic pain is also associated with changes in brain structure and function independent of depression, detectable through human brain imaging [60].

Inflammatory and immunological mechanisms were also supported by multiple analyses. Gene set enrichment analyses revealed an over-representation of risk loci near genes with functions in immune cells. Mendelian randomization identified bidirectional association of asthma and pain. Bronchial asthma often presents with chest pain or discomfort [61]. This result is consistent with the high incidence of pain related conditions such as osteoarthritis in patients with asthma [62]. Chronic activation of immune cells in damaged tissues (i.e., inflammation) is known to cause hypersensitivity of peripheral pain-sensing neurons by several mechanisms [63]. This inflammatory pain normally resolves as tissues heal, while a failure of inflammation to resolve in conditions such as arthritis contributes to chronic pain [63]. We detected mediating effects of several traits associated with persistent systemic inflammation, including asthma and obesity. Several genes at risk loci have known roles in modulating inflammatory pain. For instance, *CRHR1*, encoding corticotropin-releasing hormone (CRH) receptor 1 contributes both peripherally to the excitation of pain-sensing neurons and centrally to setting the inflammatory tone within the hypothalamus-pituitary-adrenal axis [64].

Our analyses suggest a complex genetic architecture linking pain phenotypes with other traits. Genetic correlation and genetic mediation results identified pleiotropic associations of pain risk loci with mood disorders, inflammatory factors, and metabolic and other factors. Interestingly, these pleiotropic relationships appear to be trait and locus specific. That is, distinct loci may explain the comorbidity between pain and mood disorders, compared to the comorbidity between pain and inflammatory conditions. Further studies are needed to investigate these relationships.

There are limitations associated with our approach. GWAS summary statistics available for joint analysis included only autosomes, so there is a need for future analyses to investigate X chromosome loci. Our analysis is focused on common variants, which individually have very small effect sizes and are predominantly located in non-coding regions of the genome, as is typical of GWAS findings. Bioinformatic tools remain insufficient to accurately assess the functional impacts and biological mechanisms. Rare protein-coding variants, potentially with larger effects, could be assessed by exome sequencing. Assessing pain in biobank-scale cohorts inevitably leads to a tradeoff in terms of the depth of phenotyping and knowledge of pain-specific information on individual participants. Thus, there will be value in continuing to develop cohorts with more detailed clinical assessments of pain, both cross-sectional and longitudinal. Mendelian randomization for some traits relied on a small number of SNPs, and these effects may be unstable. Also, although we were able to detect independent associations of many pain-associated SNPs, there is a lack of sufficiently large independent cohorts to replicate SNPs. Larger sample sizes will likely lead to the identification of yet more loci.

The management of chronic pain represents a substantial medical challenge. Many existing pain medications are highly addictive, especially opioids. Thus, there is an urgent need to develop new, non-addictive pain medications. Our discovery of scores of pain risk loci has the potential to identify novel therapeutic targets. Moreover, as these loci were discovered through joint analysis of multiple pain-related traits, it is likely these loci involve mechanisms that are relevant to many forms of pain. Encouragingly, genes at several of the risk loci are already being targeted therapeutically (e.g., *TRPM8*). Genes at many other loci are not yet being targeted but could represent novel strategies. To exploit this potential, it will be essential to continue to refine our understanding of the underlying mechanisms through fine-mapping and functional experiments.

## Methods

### Ethical approval

The present study, which involved deidentified data obtained from the UK Biobank Resource under Application Number 49852, received the proper ethical oversight, including the determination by the University of Maryland, Baltimore Institutional Review Board that the study is not human research (IRB #: HF-00088022).

### Sample and phenotype selection

The UK Biobank study generated genome-wide genotypes and comprehensive phenotypic data in a sample of 194,174 females and 167,020 males of ‘white British’ ancestry, who were 40–69 years old at the time of assessment [27]. GWAS summary statistics were available for >4,200 traits, including >13 million genotyped and imputed SNPs (http://www.nealelab.is/uk-biobank, GWAS round 2, accessed 10/01/20). Pain-related phenotypes were initially selected using the keyword ‘pain’. We selected traits with a sample size > 10,000 and statistically significant heritability (z>4) based on LD Score Regression (LDSC) [65] analyses performed by the Neale lab (https://nealelab.github.io/UKBB_ldsc/h2_browser.html). We calculated pairwise genetic correlations among the selected traits using LDSC [65] and set a threshold of *r*_*g*_ > 0.35 to remove traits with low genetic correlation. We analyzed autosomal markers with minor allele frequency (MAF) > = 0.01, without significant deviation from Hardy Weinberg Equilibrium (p-value>0.00001).

### Joint-analysis of 17 pain-related UKBB GWASs

A combined analysis of the 17 selected pain-related traits was carried out using PLEIO [28], which corrects for environmental correlations due to sample overlap. PLEIO relies on standardized effect sizes to express all phenotypes in the same units. Since our phenotypes are binary, we standardized effect sizes by converting them to a liability scale, assuming prevalence in the UKBB cohort is equivalent to that of the broader UK population. We first ran the meta-analysis in the whole sample and then, to evaluate the robustness of findings and detect sex-specific effects, we analyzed males and females, separately. Furthermore, we carried out PLEIO analysis combining 13 traits, removed the four phenotypes with the highest case overlap. To estimate the influence of each trait on the joint analysis we applied a leave-one-trait-out meta-analysis approach. We used the joint analysis statistics to estimate pain heritability explained by the additive effects of the SNPs using LD score regression [65].

To test the robustness of our results we carried out the combined analysis of the 17 UKBB pain GWASs using Multi-Trait Analysis of GWAS (MTAG) [66], since it allows to analyze simultaneously multiple traits GWAS summary statistics from the same cohort. As well as PLEIO [28], MTAG [66] uses LD score regression [65] to adjust for sample overlap between the traits. MTAG analyzes one trait at a time and achieves maximum statistical power when the traits included in the analysis have a high genetic correlation; in our analysis we generated 17 MTAG output for each trait analyzed.

### Risk loci and gene mapping

Post-GWAS analyses of risk loci, gene mapping, and functional annotation were performed with FUMA SNP2GENE [30]. Risk loci were defined using 1000 Genomes Phase 3 LD through the following steps: 1. Selection of all genome-wide significant SNPs (p-value < 5e-8) independent from each other (r2<0.6); 2. Inclusion of additional candidate SNPs in LD (r^2^ > = 0.6) with SNPs selected in step1; 3. Definition of lead SNPs as independent from each other at r^2^ < 0.1. LD blocks of independent significant SNPs <250kb apart were merged into a single locus. SNPs in LD with an independent significant SNP were functionally annotated using ANNOVAR [67], based on Ensembl build 85. Gene mapping was performed using three complementary approaches: 1. “Positional”: selects any gene +/- 10kb from the tag SNP, 2. “Expression quantitative trait locus (eQTL)”: we used the PsychENCODE [68] and Genotype-Tissue Expression Consortium [69] databases and considered only cis-eQTLs, defined as genes located < 1MB from the tag SNP. 3. “Chromatin interaction”: we used data from Psych ENCODE [68] and considered only one-way enhancer-promoter interactions within 500 kb of the tag SNP.

### Human pain transcriptome datasets

We examined the expression patterns of the 664 genes mapped at risk loci using our published RNA sequencing data from the blood of human patients with low back pain [70]. The transcriptome dataset included 64 samples divided into 4 groups: patients at the onset of low back pain who no longer had pain within 6 weeks after onset (acute), baseline in patients who developed chronic pain at 6 months (n = 13, chronic T1 group); and at 6 months after baseline in patients with chronic pain (n = 19, chronic T5 group) and healthy pain-free controls (n = 21, normal group).

### Gene set enrichment analysis

We performed gene set enrichment analysis of GWAS summary statistics using MAGMA [31]. Initial steps, including SNP annotation and calculation of gene-based p-values, were performed in FUMA [30] using default parameters and 1000 Genomes Phase 3 as reference. Next, competitive gene-set analysis was carried out with gene sets annotated using MSigDB v7.0 (https://www.gsea-msigdb.org/gsea/msigdb) [71]. Similarly, a tissue expression analysis was run where the previously computed gene-based p-values were conditioned by the average expression of genes per tissue type; FUMA [30] implements these analyses using both 30 general and 53 specific tissue types from the GTEx consortium [69]. In addition, we tested if the genes detected in our study were enriched for genes previously found linked to pain. For this purpose, we used two lists of genes: (i) genes derived from human pain genetic studies [32]; (ii) pain genes identified in murine knockout studies [33].

### Pleiotropic effects of individual SNPs

Independent significant SNPs and other SNPs in LD with them at each risk locus were checked for association with non-pain-related traits using the GWAS Atlas [48]. SNP-trait associations from the GWAS Atlas (release3, “gwasATLAS_v20191115”) were obtained from the GWAS Atlas website, https://atlas.ctglab.nl/. The GWAS Atlas includes only genome-wide significant SNPs.

### Genetic correlation and Mendelian Randomization

We used LDHub [37] (accessed on October 21, 2021) to conduct an unbiased genetic correlation analysis between pain and all 254 non-pain traits in the database, excluding UKBB cohort studies. Next, we conducted Mendelian randomization (MR) analysis to investigate whether the high genetic correlation and pleiotropy observed between pain and non-pain traits is due to a shared genetic etiology or rather is the result of a causal link between them.

Among the non-pain traits showing the highest genetic correlation and pleiotropy with our study, we selected quantitative traits including BMI, lipoproteins, C-reactive protein, hemoglobin, and fibrinogen as biomarkers of cardiovascular, metabolic, as well as binary phenotypes as depression, asthma, coronary heart disease, and rheumatoid arthritis. To test the causal hypothesis, we conducted two-sample Mendelian randomization (MR) using the R packages TwoSampleMR (version 0.4.26) [43]. Since our analysis on pain was performed using summary statistics from the UKBB cohort, we looked for GWAS summary statistics of the targeted traits carried out in independent European samples that did not include UKBB. The most critical step in the MR analysis is the selection of the genetic variants. To be considered as valid instruments (VI) for testing the causal hypothesis, genetic variants must satisfy three main assumptions: i. “Relevance”; shows a robust association with the risk factor, ii. “Independence”; there is no confounding factor in the association with the outcome; iii. “Exclusion”; influence the outcome only through exposure. Deviation from this assumption is defined as “horizontal pleiotropy” or simply “pleiotropy”. We used the MRInstrument (version 0.3.2) R package [43] to select the exposure GWASs and extracted the SNPs with the most significant statistical association. SNPs in high LD were pruned, and effect alleles were harmonized in the exposure and outcome datasets. The instrumental variants selected for the analysis of pain as outcome are listed in **Table X in S1 Data**. We used the leading SNPs at the 99 loci detected in this report as instrumental variants in the MR analysis where genetic exposure to pain was tested as risk factor for cardiometabolic, psychiatric and inflammatory conditions (**Table Z in S1 Data**). As additional parameter in our MR analysis we implemented the R2 and F-statistics. R2 indicates the power of a MR study and is measured as the proportion of the variability of the exposure trait explained by the SNPs [72]. F-statistic estimates the strength of the genetic variants for each exposure conditional on the other exposures included in the model [46,72]; we considered instruments to have sufficient strength if their corresponding F-statistic is >10.

There are numerous analysis methods for MR, which mainly differ in the assumption about pleiotropy. We followed recent best-practice guidelines [45], as follows. We applied the variance weighted (IVW) with multiplicative random effects as the principal method, since it has the greatest power when SNPs show balanced or no pleiotropy. We applied several additional methods that make different assumptions on pleiotropy: MR-Egger [73] is based on the InSIDE assumption that allows all genetic variants to have pleiotropic effects if these are independent of the variant–exposure association. In addition, we used weighted median and mode methods that respectively assume the median and majority of the SNPs used in the analysis are valid instruments [73]. Finally, we used the MR-PRESSO R package (version 1.0),which computes a casual estimate like IVW but without outliers [74]. Together with the MR statistics, we report measures of heterozygosity between variant-specific causal estimates, such as Cochran’s Q statistics [46] using IVW and Egger causal estimates and the I2 statistic computed from Cochran’s Q statistics[46] Q and test degree of freedom (df) as I2 = (Q-df/Q)*100, and pleiotropy as the intercept of Egger regression [73]. For each trait analyzed, we applied leave-one-out analyses to verify that the estimate of the causal effect is not due to a single genetic variant strongly associated with the exposure [38,40].

Our MR analysis was conducted using both continuous and binary exposure variables. Exposure to a binary measure is “conceptualized” as the dichotomization of an underlying continuous risk factor. Assuming that the genetic associations with the binary exposure have been estimated using logistic regression and are expressed as log odds ratios, a unit increase in the log odds of a variable corresponds to an exp (1) = 2.72 -fold multiplicative increase in the prevalence of the exposure. We multiplied MR causal estimates obtained with binary exposure by 0.693 (log_e_ 2) and interpreted the results as average change in the outcome per doubling (2-fold increase) in the prevalence of exposure [75]. Finally, to rule out the possibility that the genetic variants influence the exposure via the outcome, we performed a reverse analysis testing the causal effect of pain on the metabolic, cardiovascular, inflammatory/immunological, and psychiatric traits [38,40], and we further applied the MR-Steiger method [76], implemented in TwoSampleMR R package, that recognizes instrumental variables of exposure that are primarily associated with the outcome and remove them from the analysis.

We conducted a two-sample multivariable MR analysis to test causality between exposure to high levels of lipoproteins and pain. As VI we selected SNPs associated at a genome-wide significant level with either HDL, LDL, or Triglycerides (Table CC in S1 Data). We used MVMR (version 0.2.0) and Mendelian Randomization (version 0.5.0) [44] R packages, which extend IVW, MR-Egger, and Cochran’s Q statistical tests to multivariable MR analysis.

### Replication in independent cohorts

We conducted replication analyses in independent, European ancestry cohorts to evaluate the robustness of our lead SNPs associations with two pain traits: GWAS of “Pain(limb, back, neck, head abdominally) “in the FinnGen cohort (gs://finngen-public-data-r8/summary_stats/finngen_R8_PAIN.gz); GWAS of migraine headache [13]. We also considered their pleiotropic effects on five genetically correlated traits: BMI [50,51], lipoproteins [54], CAD [47], asthma [53] and depression [49]. For each trait, we studied the lead SNPs at each of our 99 genome-wide significant pain loci. For each replication analysis, we set a p-value threshold, *P* < 0.0005 (0.05 / 99).

## Supporting information

S1 Text Considerations on the selection and analysis of pain-related phenotypes. (DOCX)

S1 Data Index of Supplementary Tables by order of appearance. (XLSX)

S2 Data Pleiotropic effects of pain risk SNPs on non-pain traits. Pleiotropic effects were mapped by integration with the GWAS Atlas. Traits are grouped in 21 categories listed on different sheets in the table.
(XLSX)

S1 Fig Proportion of cases (A) and controls (B) shared by the 17 phenotypes.
(PDF)

S2 Fig Comorbidity pain traits measured as Relative Risk and its 95%CI (axis X) of developing pain at one site given the exposure to another pain site. (PDF)

S3 Fig Risk of suffering a type of pain given the intake of a specific pain medicine. X-axis Risk indicates Relative Risk of pain and its 95%CI. (PDF)

S4 Fig Pleio circo-plots of all 99 loci identified in the study. Each plot illustrates the contribute of each trait to the signal, as p-value, size, and direction of the effect. (PDF)

S5 Fig Zoom plots of all 99 loci identified in the study. Each plot illustrates the genes mapping the locus, CADD score of the top significant genes, eQTL and chromatin interaction information estimated using PsychENCODE database.
(PDF)

S6 Fig Quantile-quantile (QQ) plot for the joint GWAS analysis shown in Fig 1C. (PDF)

S7 Fig Sex-specific joint GWAS analysis of pain-related traits. (PDF)

S8 Fig Quantile-quantile (QQ) plots for sex-specific analyses. A. Female only. B. Male only.
(PDF)

S9 Fig Sensitivity analysis heatmap. The matrix is represented by the difference in significance (-log10P-value) between the value obtained in the meta-analysis run with the correspondent left out trait (columns) and the higher significance value at that locus. All 99 are listed in the rows. (PDF)

S10 Fig Venn diagram showing the overlap of genes selected by position, eQTLs and chromatin interaction gene mapping methods. (PDF)

S11 Fig Enrichment of pain risk loci for tissue-specific gene expression. The X axis indicates the significance of the test and in the Y axis indicates tissues analyzed. (PDF)

S12 Fig Mendelian randomization depression exposure pain outcome. (PDF)

S13 Fig Mendelian randomization coronary heart disease exposure pain outcome. (PDF)

S14 Fig Mendelian randomization pain exposure coronary heart disease outcome. (PDF)

S15 Fig Mendelian randomization BMI. (PDF)

S16 Fig Mendelian randomization Asthma exposure pain outcome. (PDF)

S17 Fig Mendelian randomization pain exposure asthma outcome. (PDF)
