# Supplementary material for: Genome wide association joint analysis reveals 99 risk loci for pain susceptibility and pleiotropic relationships with psychiatric, metabolic, and immunological traits
Source: PLoS Genet. 2023 Oct 16;19(10):e1010977. doi: 10.1371/journal.pgen.1010977 (PMC10602383; doi:10.1371/journal.pgen.1010977)
Supplement: S1 Text — (DOCX) [file pgen.1010977.s001.docx]

**S1 Text. Considerations on the selection and analysis of pain-related phenotypes.** Ten pain phenotypes were created through a touchscreen questionnaire with the main question: “pain types experienced in the last month” (field ID 6159). The possible answers were: ‘None of the above’ (renamed in the present study as “pain any” and coded as 6159_100); ‘Prefer not to answer’ (6159_9); pain at seven different body sites (head (6159_1)), face (6159_2), neck/shoulder (6159_3), back (6159_4), stomach/abdominal (6159_5), hip (6159_6), knee (6159_7 ); or ‘all over the body’ (6159_8) . With the exception of the field 6159_8, all the others are not mutually exclusive since a participant can experience pain in multiple parts of the body and thereby, they can select multiple answers. Patients that experienced pain in the last month were further asked whether the pain had lasted for 3 months or longer. This condition is described as chronic pain and indicated with the category ID 100048; for this study we selected back and headache chronic pain sites, coded as "3571” and “3799” respectively. In addition, we included five traits symbolized with code “6154”, specify distinct pain relief medications: Aspirin (6154_1), Ibuprofen (6154_2), Paracetamol (6154_3), Omeprazol (6154_5) and in addition Medication none of the above (that we named as medication any) (6154_100). Finally, we included two pain-related phenotypes to our combined analysis, “chest pain or discomfort” marked with the code “2335” and extracted from the category chest pain and “leg pain on walking” derived from the category claudication and peripheral artery disease and identified with the code “4728”. We note that our choice of phenotypic coding could influence interpretation.

First, a small number of individuals who did not respond to pain-related questions are included in

the analysis, coded as “population controls”. The inclusion of population controls maximizes sample size. In practice, since response rates to all the pain-related traits were very high, this affects at most a few percent of individuals in the analysis. Second, individuals with chronic pain for 3+ months are compared to individuals who experienced pain for shorter amounts of time (i.e., “pain-exposed” controls). We believe this definition leads to a cleaner phenotype than a comparison with individuals that did not experience pain at all, but we note that it leads to a small sample size for the chronic pain traits. Third, the analysis treats each trait separately. As such, certain individuals are treated as controls for one trait and cases for another; i.e., an individual who only experiences pain in their back but not in their chest would be considered a case for back pain but a control for chest pain. While potentially confusing, we feel that this is a justifiable choice to explore potential differences between pain location risk susceptibility.
