# Supplementary figures and images for: Genome wide association joint analysis reveals 99 risk loci for pain susceptibility and pleiotropic relationships with psychiatric, metabolic, and immunological traits
Source: PLoS Genet. 2023 Oct 16;19(10):e1010977. doi: 10.1371/journal.pgen.1010977 (PMC10602383; doi:10.1371/journal.pgen.1010977)

## Cases

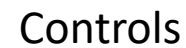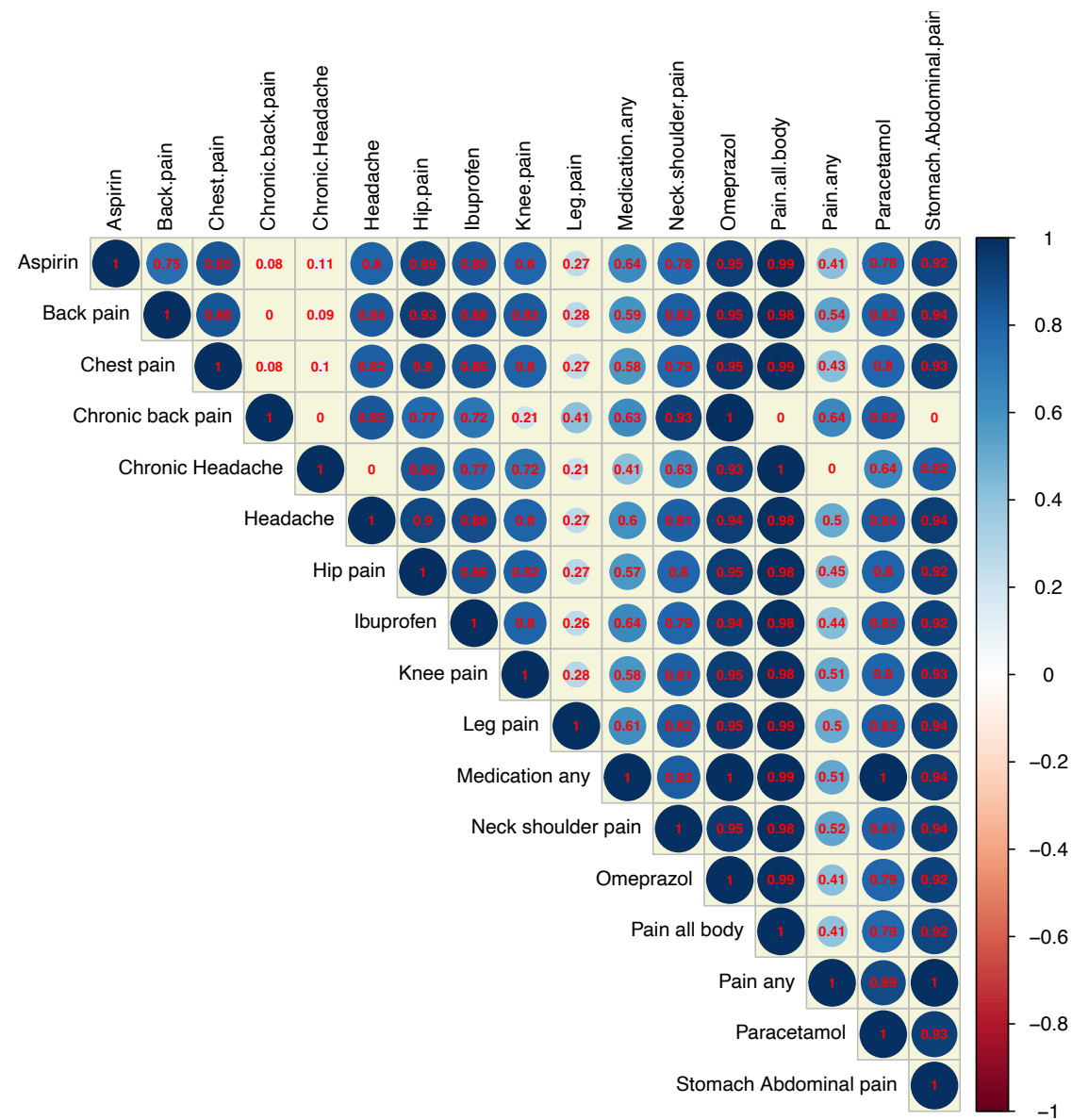

Supplement: S1 Fig — Proportion of cases (A) and controls (B) shared by the 17 phenotypes. (PDF) [file pgen.1010977.s004.pdf]

**S3 Figure. Pain medication–Risk of pain**

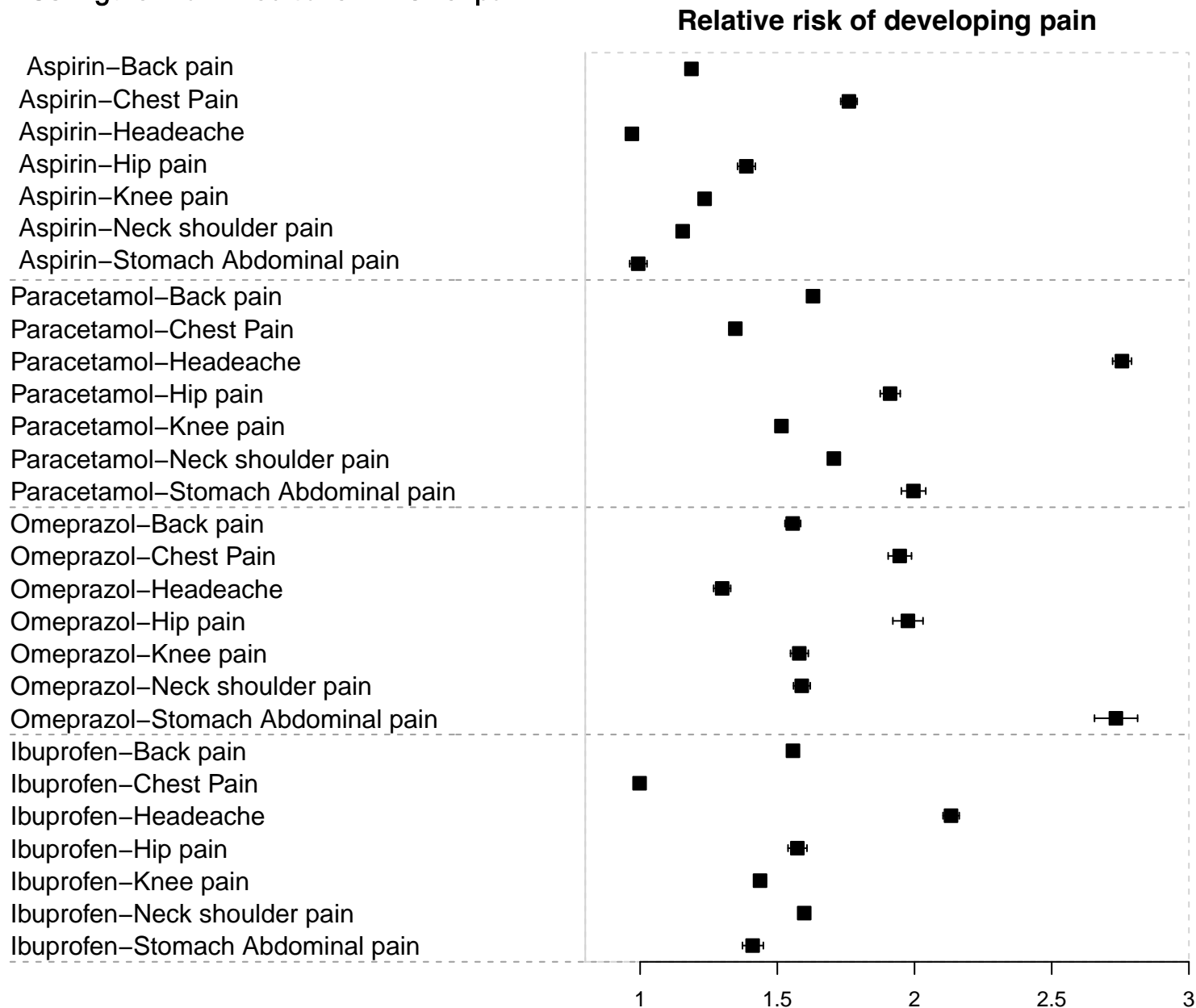

Supplement: S3 Fig — (PDF) [file pgen.1010977.s006.pdf]

**S6\_Figure.** Quantile-quantile (QQ) plot for the joint GWAS analysis shown in **Figure 2E**.

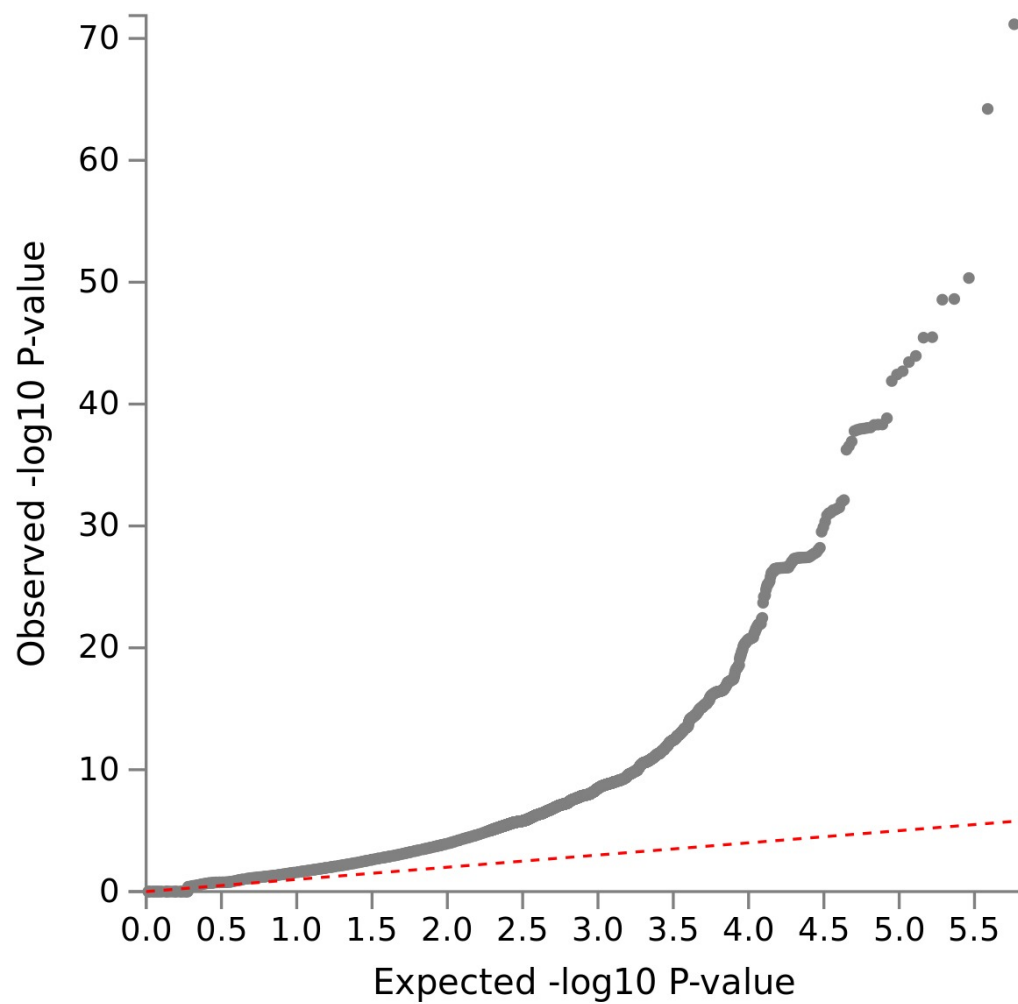

Supplement: S6 Fig — (PDF) [file pgen.1010977.s009.pdf]

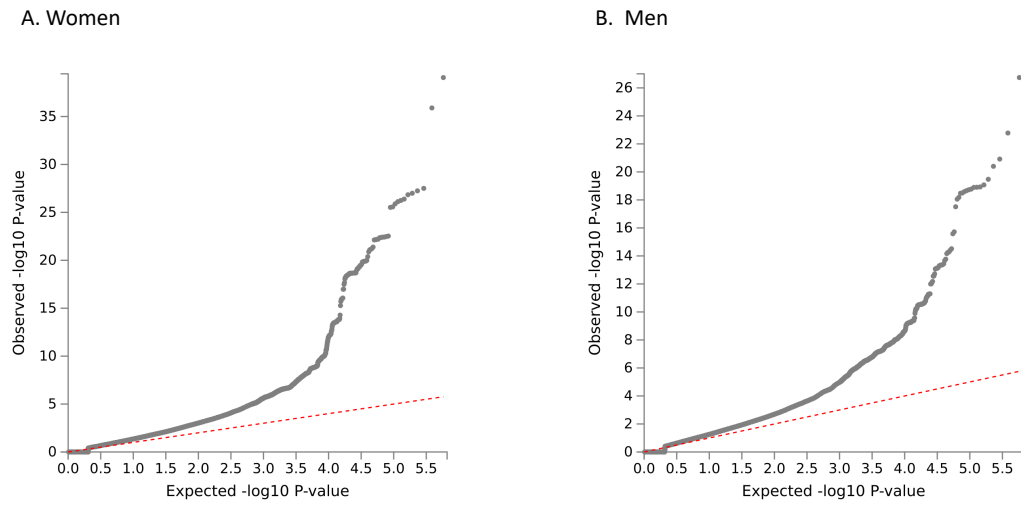

**S8\_Figure. Quantile-quantile (QQ) plots for sex-specific analyses. A. Female-only. B. Male-only.**

Supplement: S8 Fig — A. Female only. B. Male only. (PDF) [file pgen.1010977.s011.pdf]
