# Supplementary material for: Genome wide association joint analysis reveals 99 risk loci for pain susceptibility and pleiotropic relationships with psychiatric, metabolic, and immunological traits
Source: PLoS Genet. 2023 Oct 16;19(10):e1010977. doi: 10.1371/journal.pgen.1010977 (PMC10602383; doi:10.1371/journal.pgen.1010977)

S4_Figure. Circoplots of the 99 genome-wide significant loci

Locus1 1p36.23, rs301805 Pleio-P= 1.56E-08, intronic to *RERE* gene

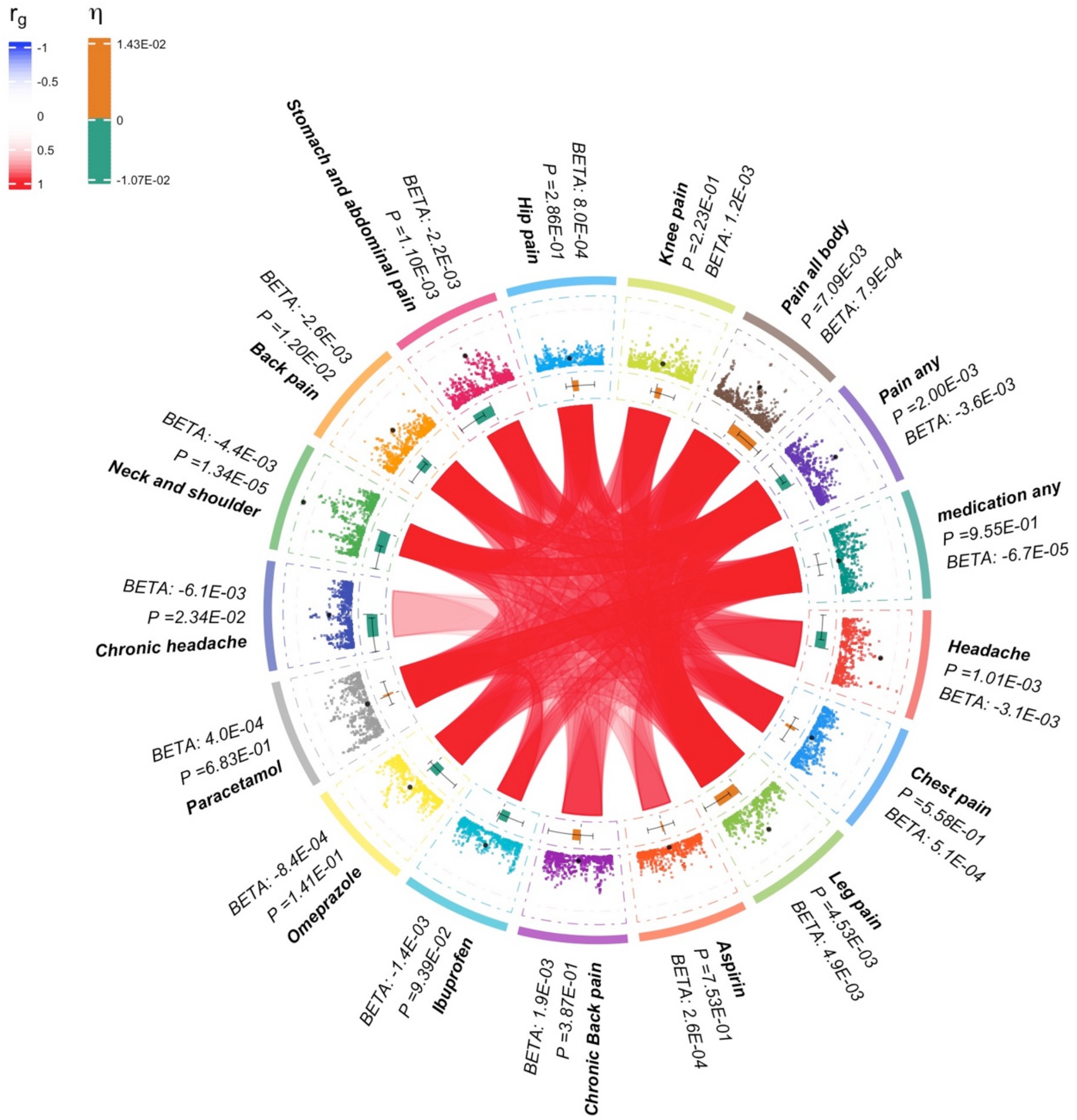

Locus2 1p36.21, rs12070469 Pleio-P= 4.63E-08, intronic to *TMEM51* gene

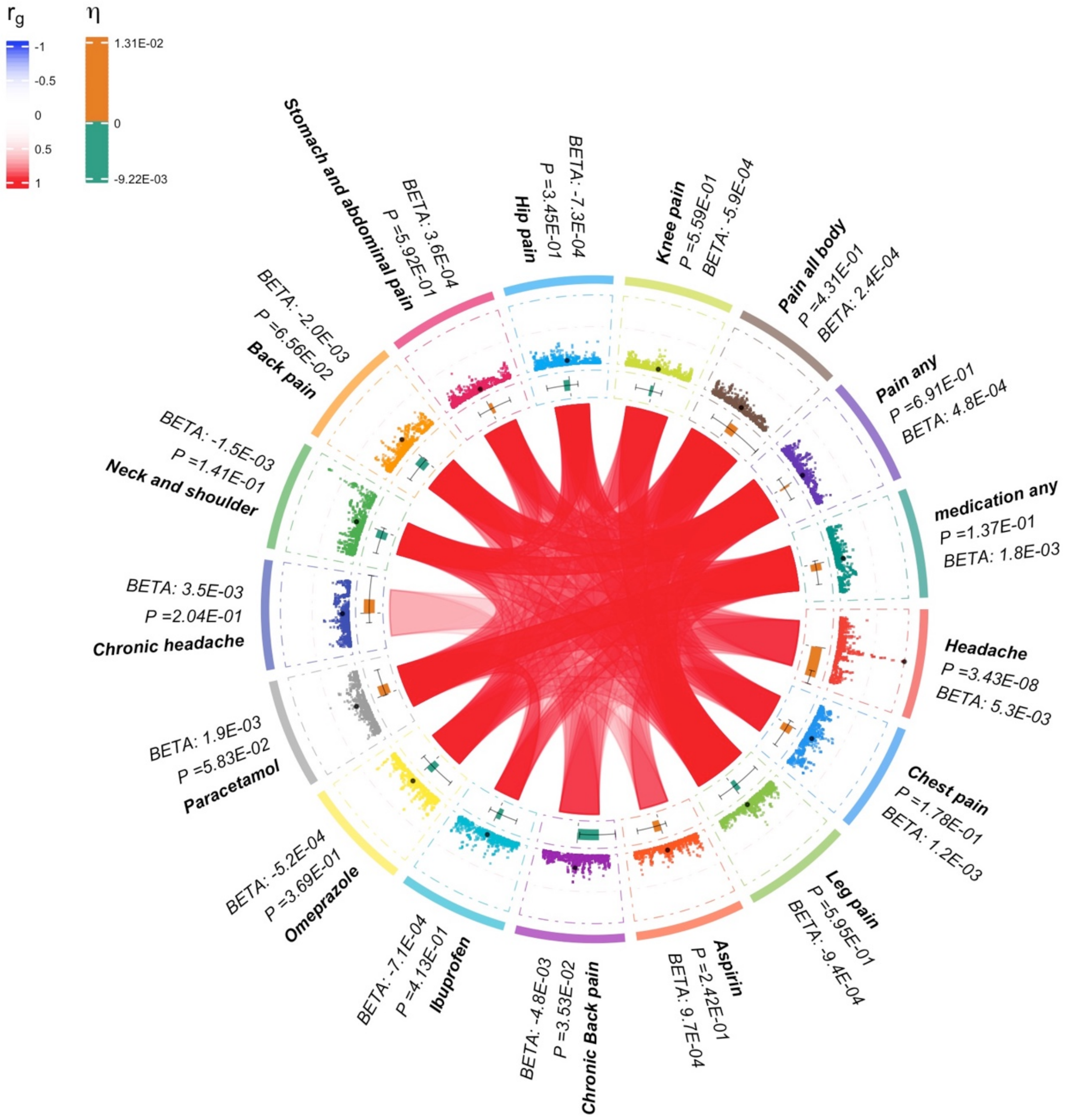

Locus3 1p34.3, rs16826012 Pleio-P= 7.03E-10, ncRNA_intronic to *MACF1:RP11-420K8.1* gene

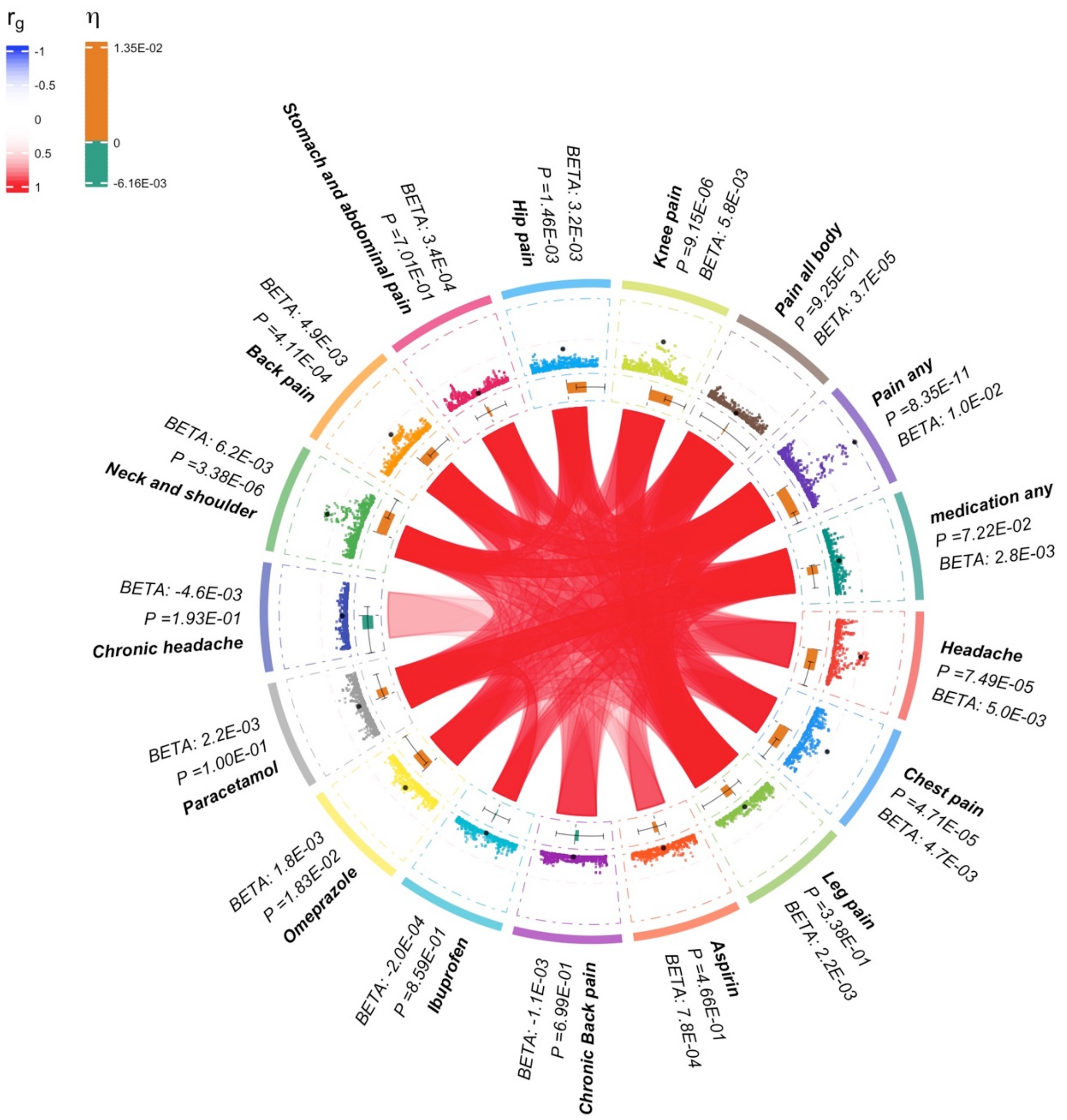

Locus4 1p32.3, rs6658904 Pleio-P= 2.49E-11, intronic to *FAF1* gene

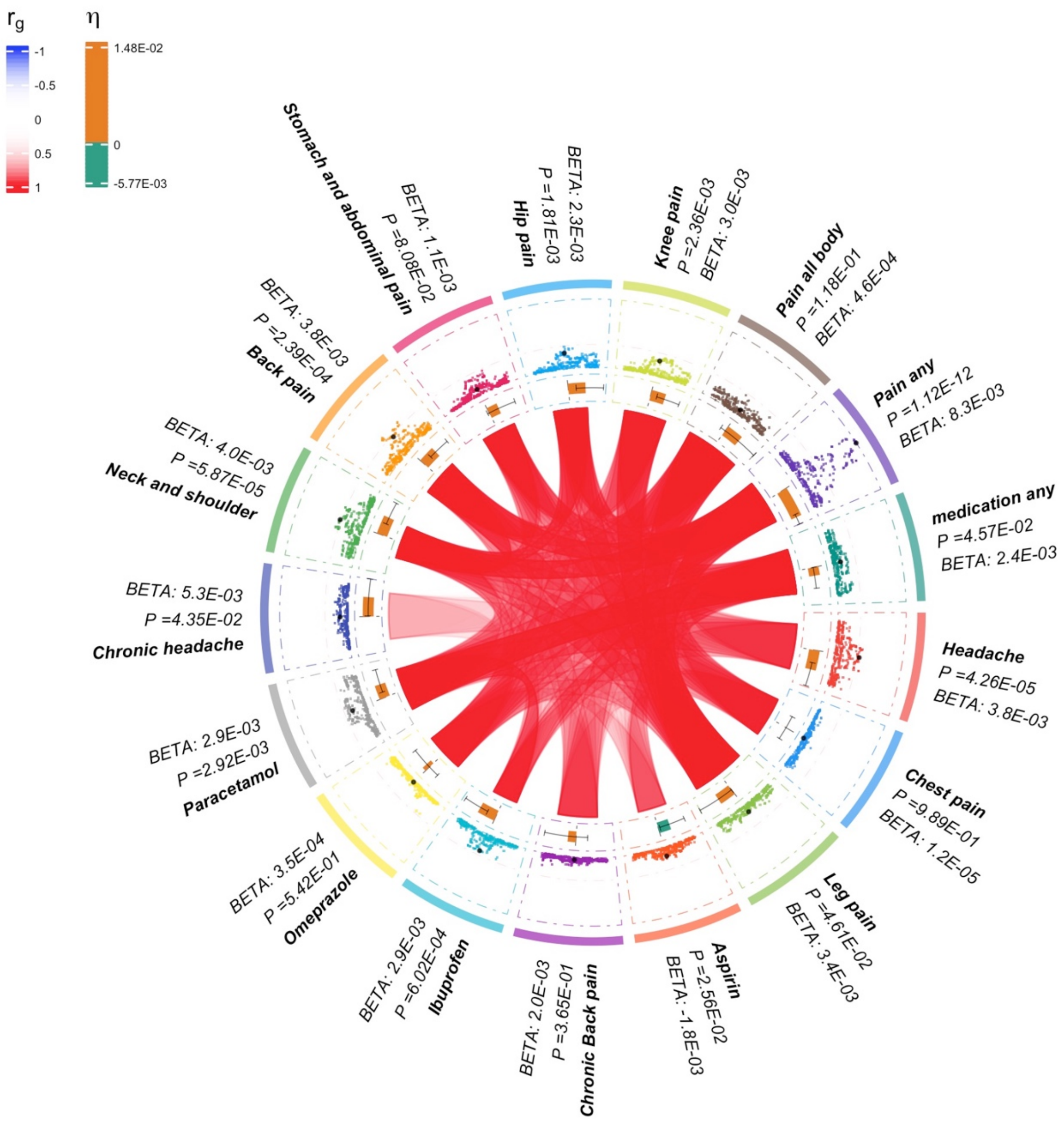

Locus5 1p31.3, rs1325266 Pleio-P= 8.94E-11, UTR5 to *SGIP1* gene

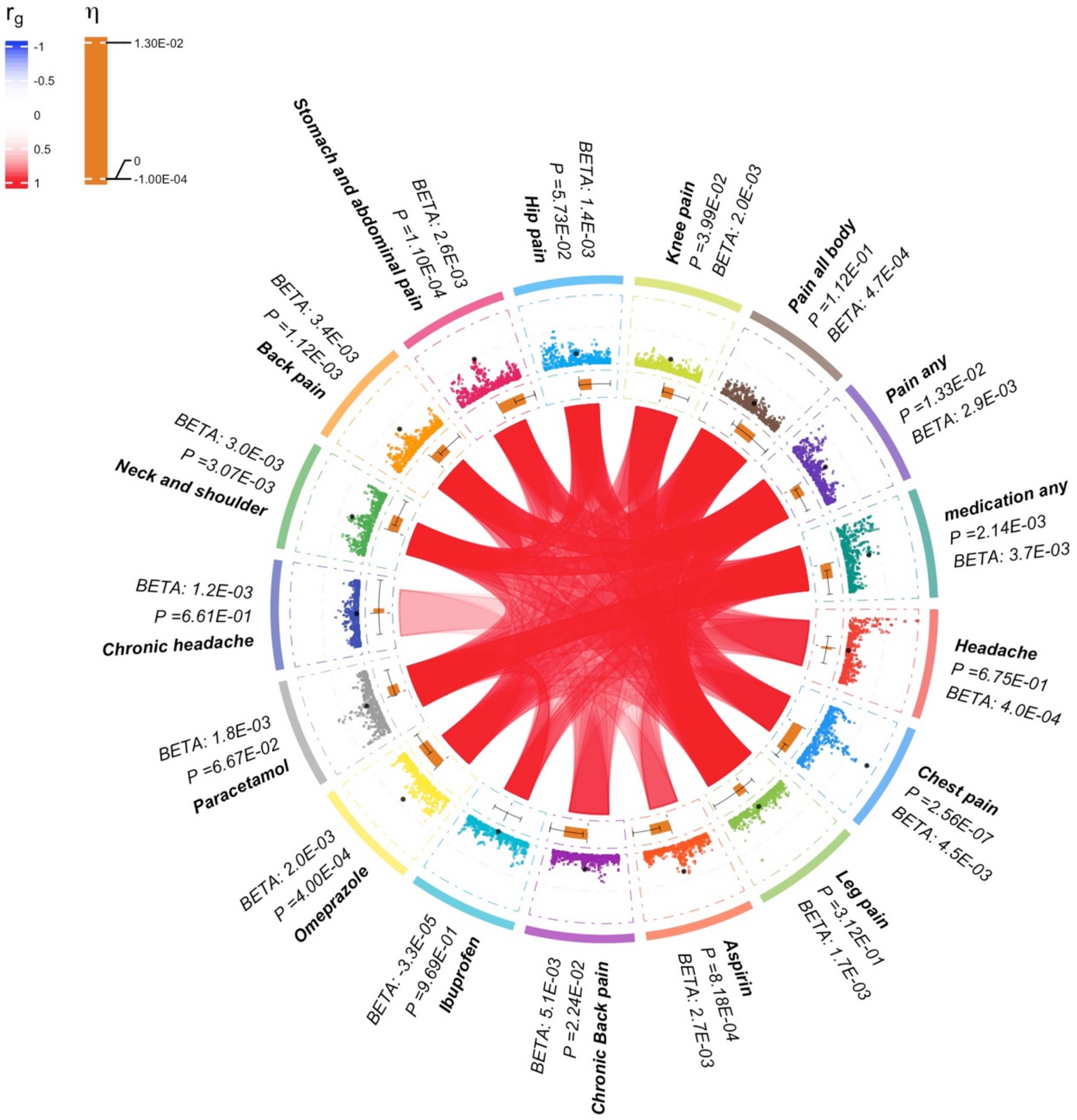

Locus6 1p22.1, rs10782959 Pleio-P= 2.61E-08, ncRNA_exonic to *RP4-717I23.3:DR1* gene

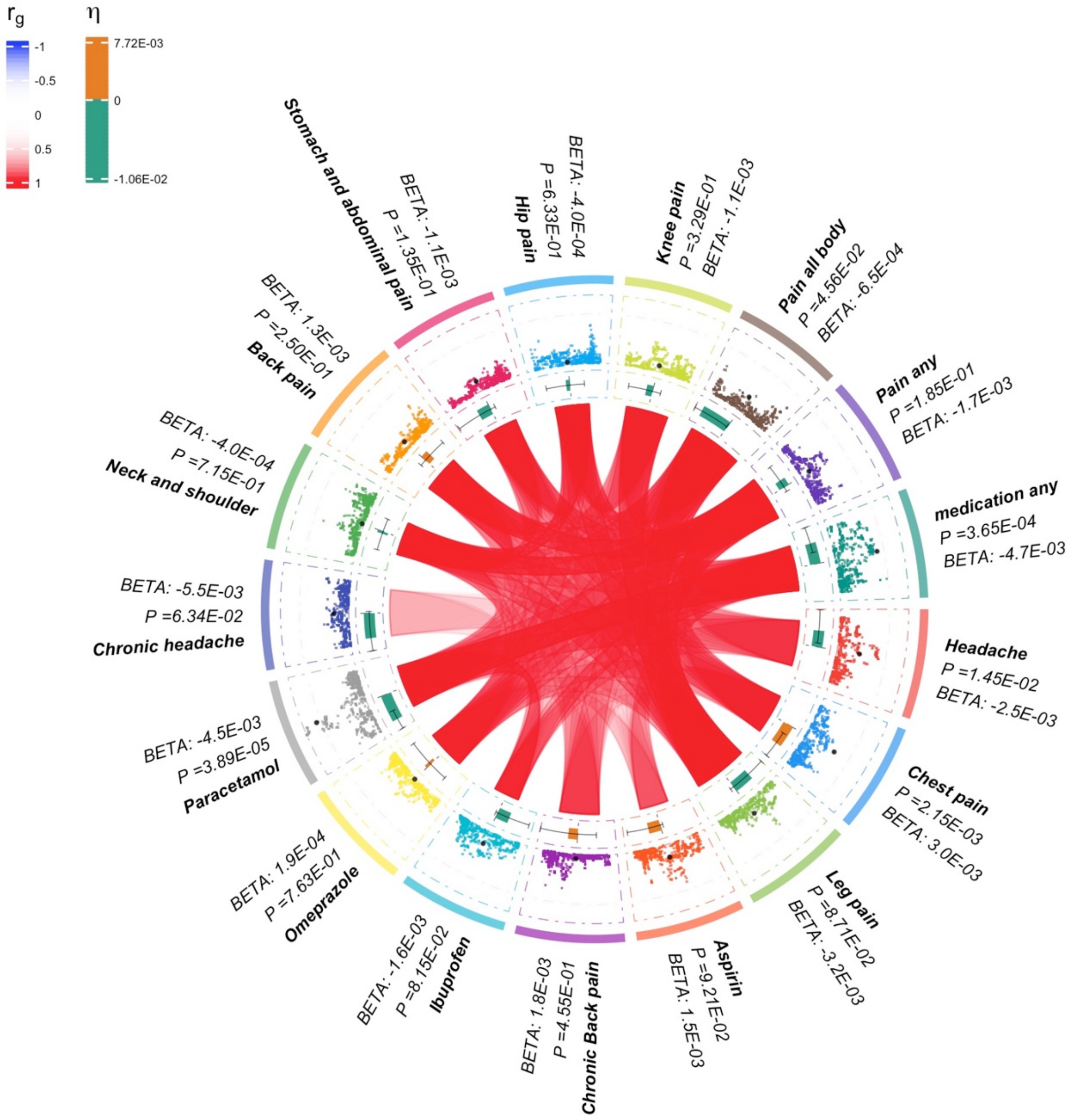

Locus7 1p21.3, rs1198583 Pleio-P= 1.67E-10, intergenic to *NFU1P2* gene

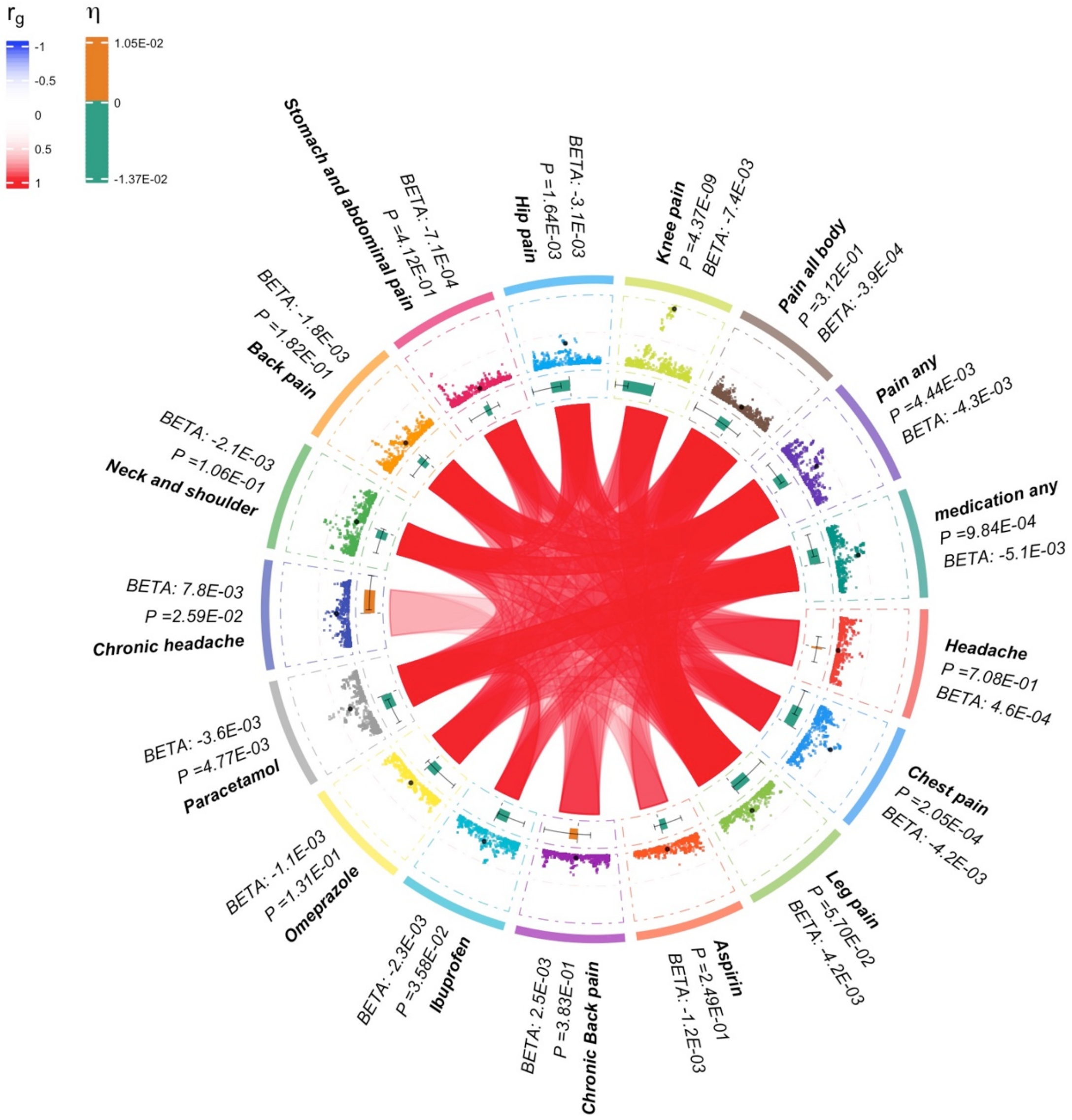

Locus8 1p13.3, rs583104 Pleio-P= 1.09E-08, downstream to *PSRC1* gene

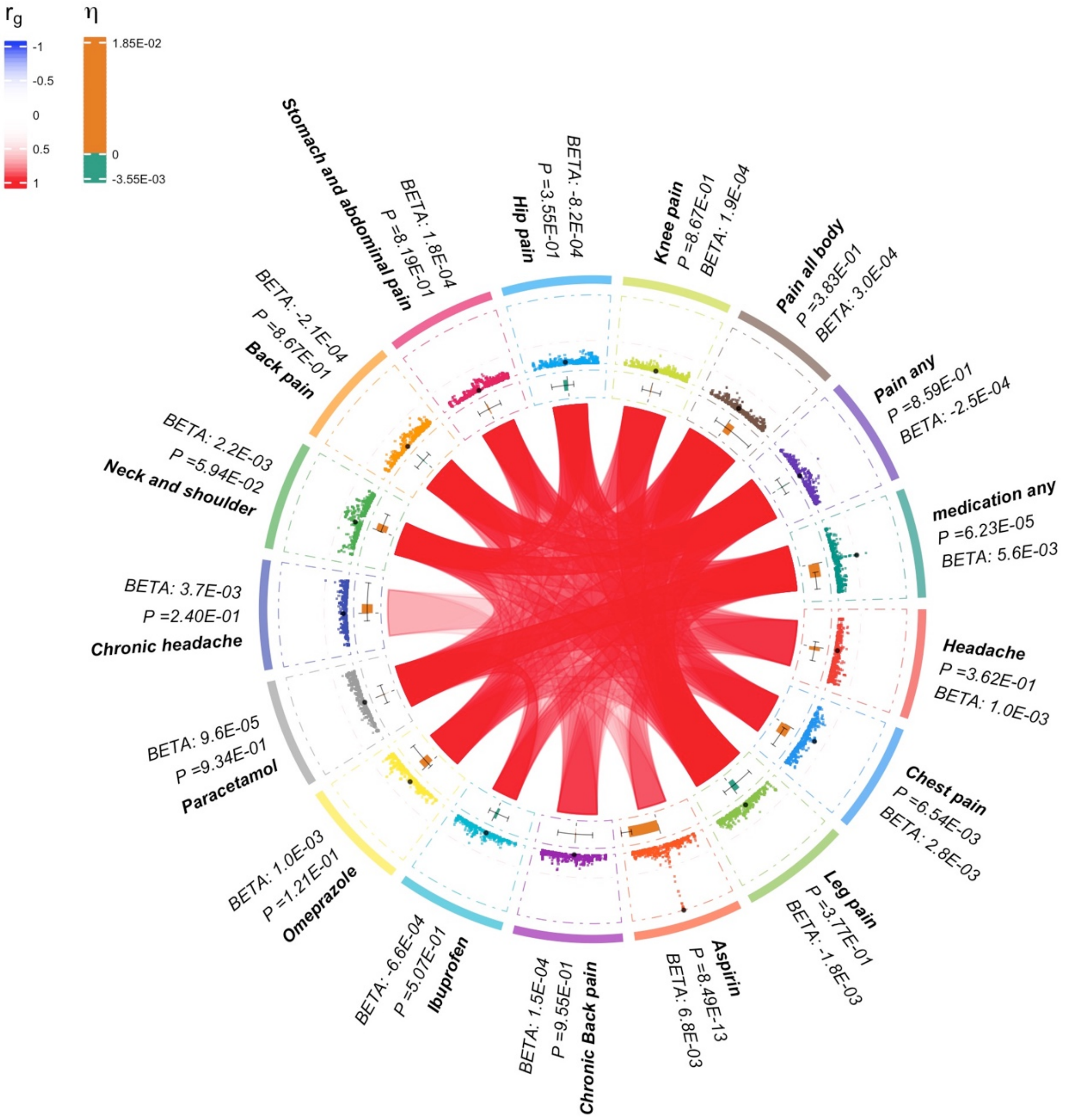

Locus9 1p13.2, rs12134493 Pleio-P= 9.60E-26, intergenic to *RP4-666F24.3* gene

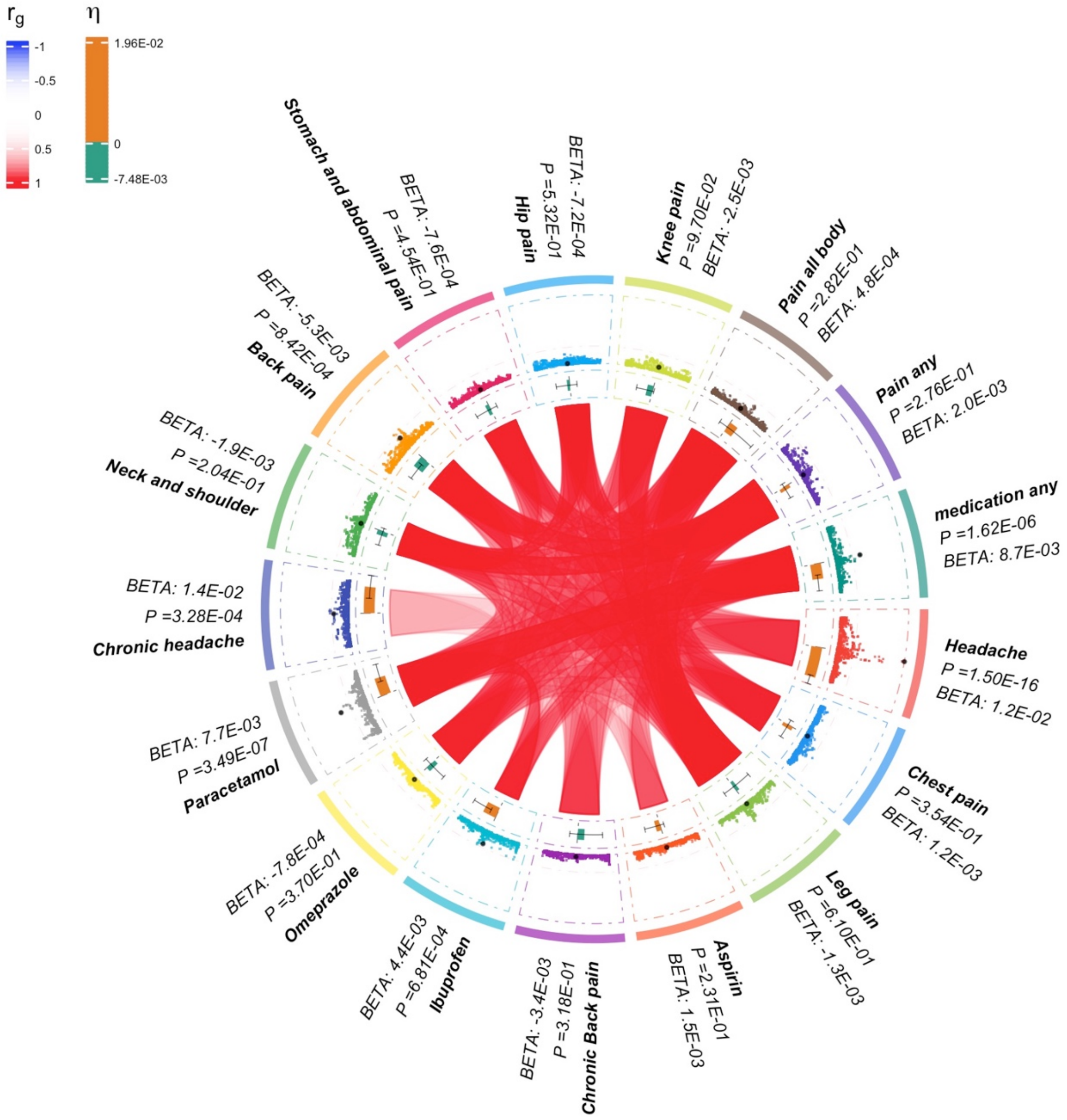

Locus10 1q21.3, rs698915 Pleio-P= 4.09E-14, intronic to *RPRD2* gene

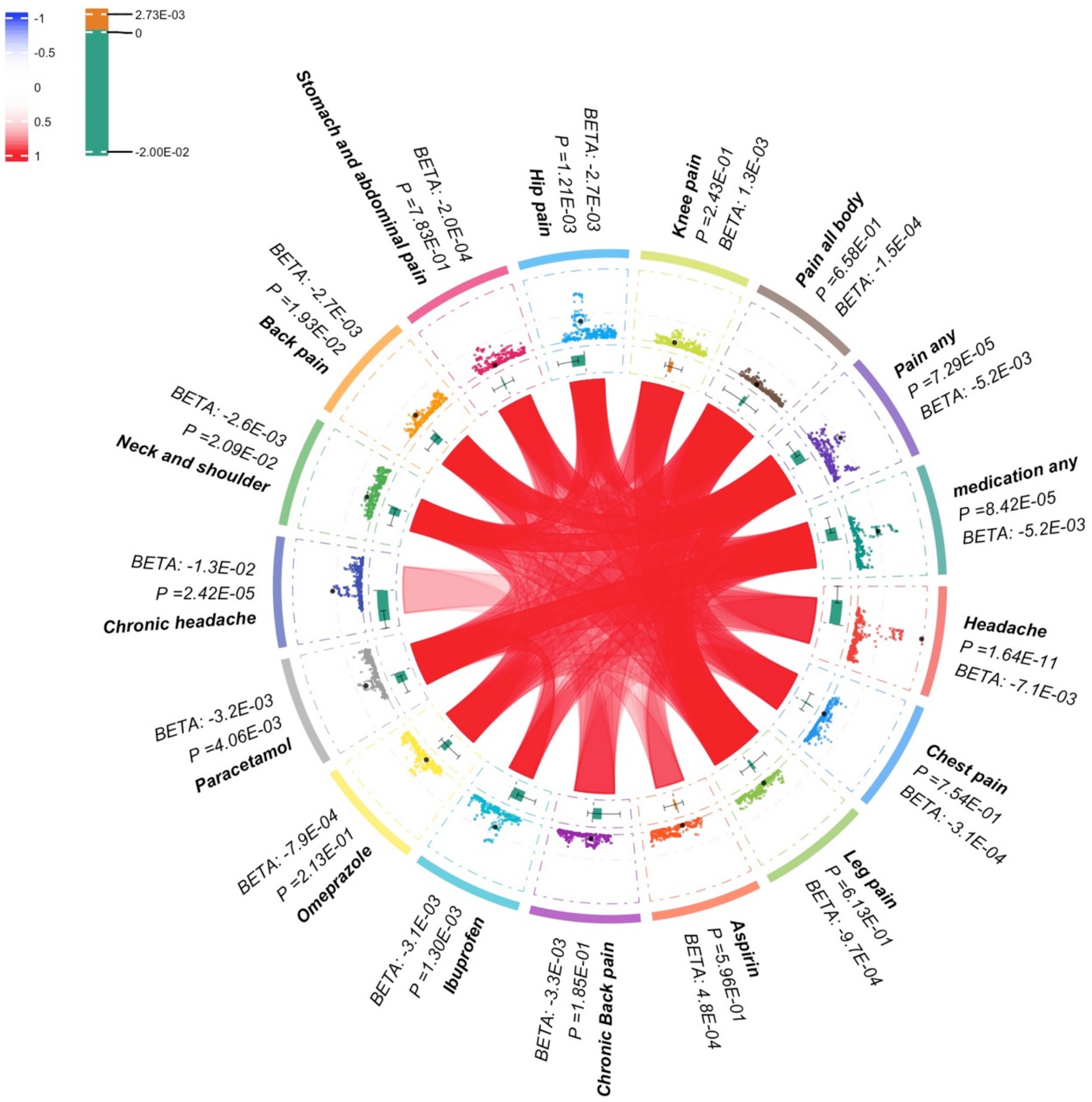

Locus11 1q21.3, rs9426902 Pleio-P= 4.75E-08, intronic to *INTS3* gene

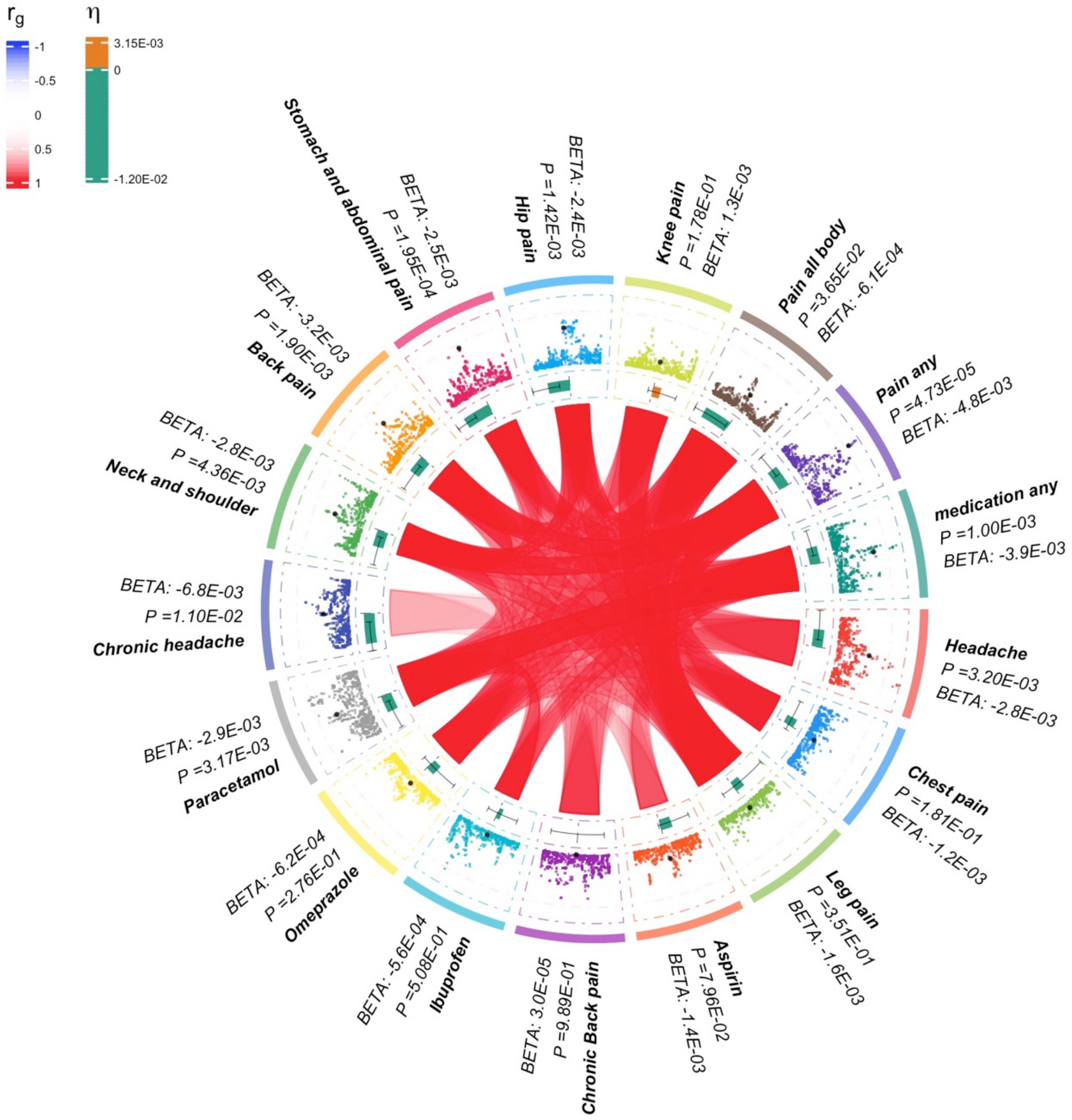

Locus12 1q22, rs1050316 Pleio-P= 6.16E-29, UTR3 to *MEF2D* gene

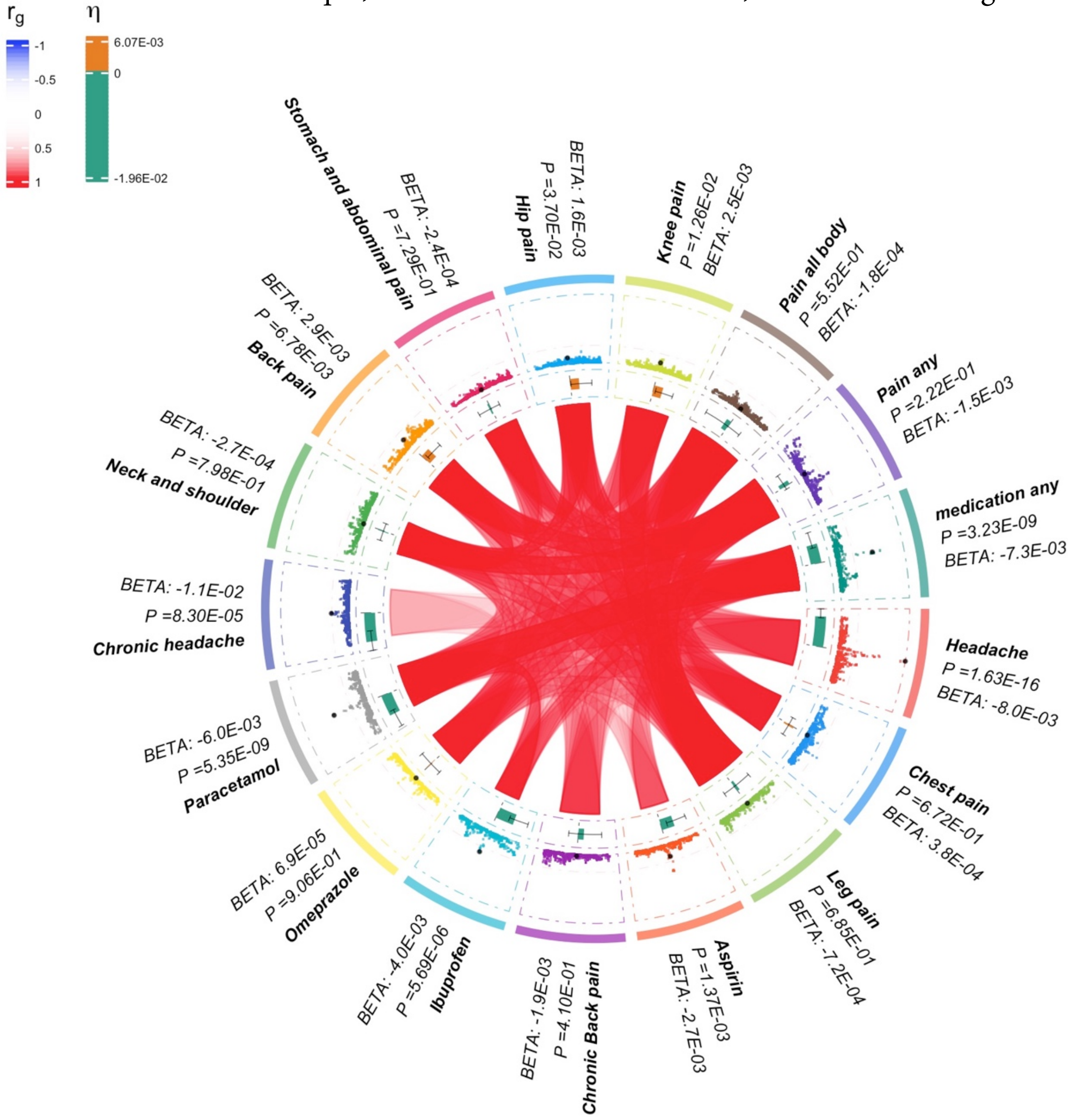

Locus13 1q25.1, rs12568655 Pleio-P= 5.10E-12, intronic to *RABGAP1L* gene

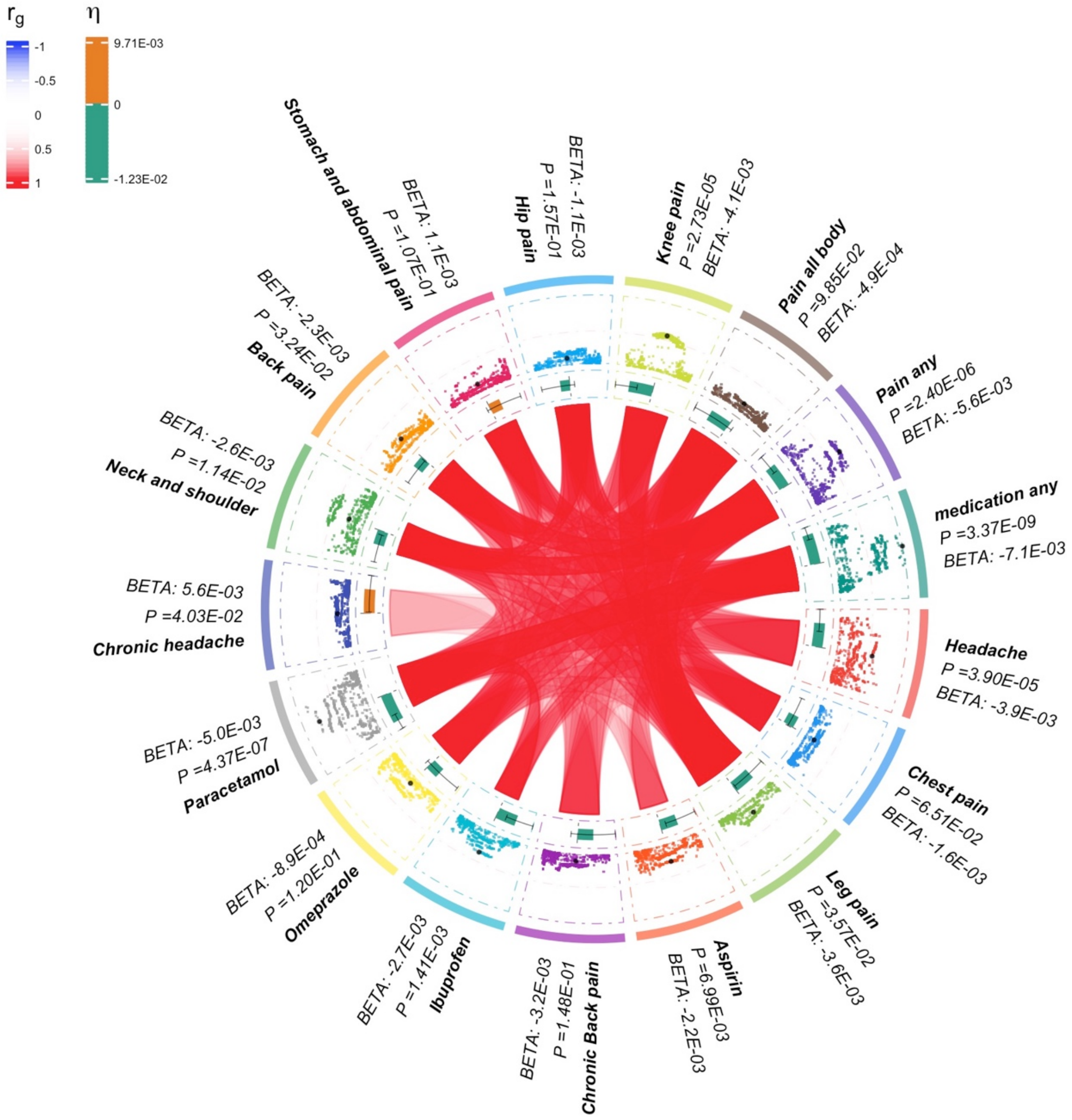

Locus14 1q43, rs3904683 Pleio-P= 1.54E-12, UTR5 to *CEP170* gene

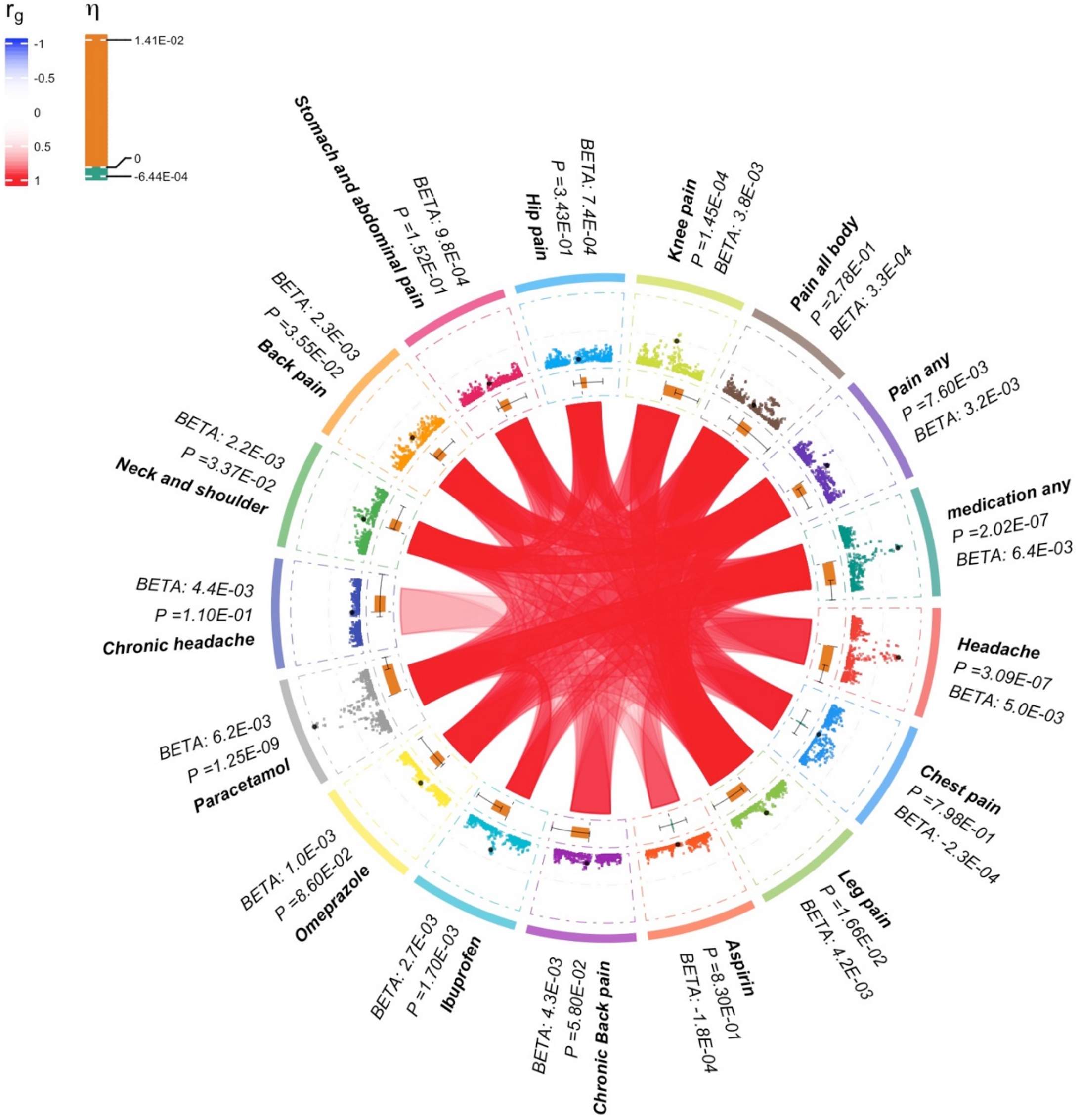

Locus15 2p24.1, rs1876767 Pleio-P= 2.32E-12, ncRNA_intronic to AC068490.2:AC096570.2 gene

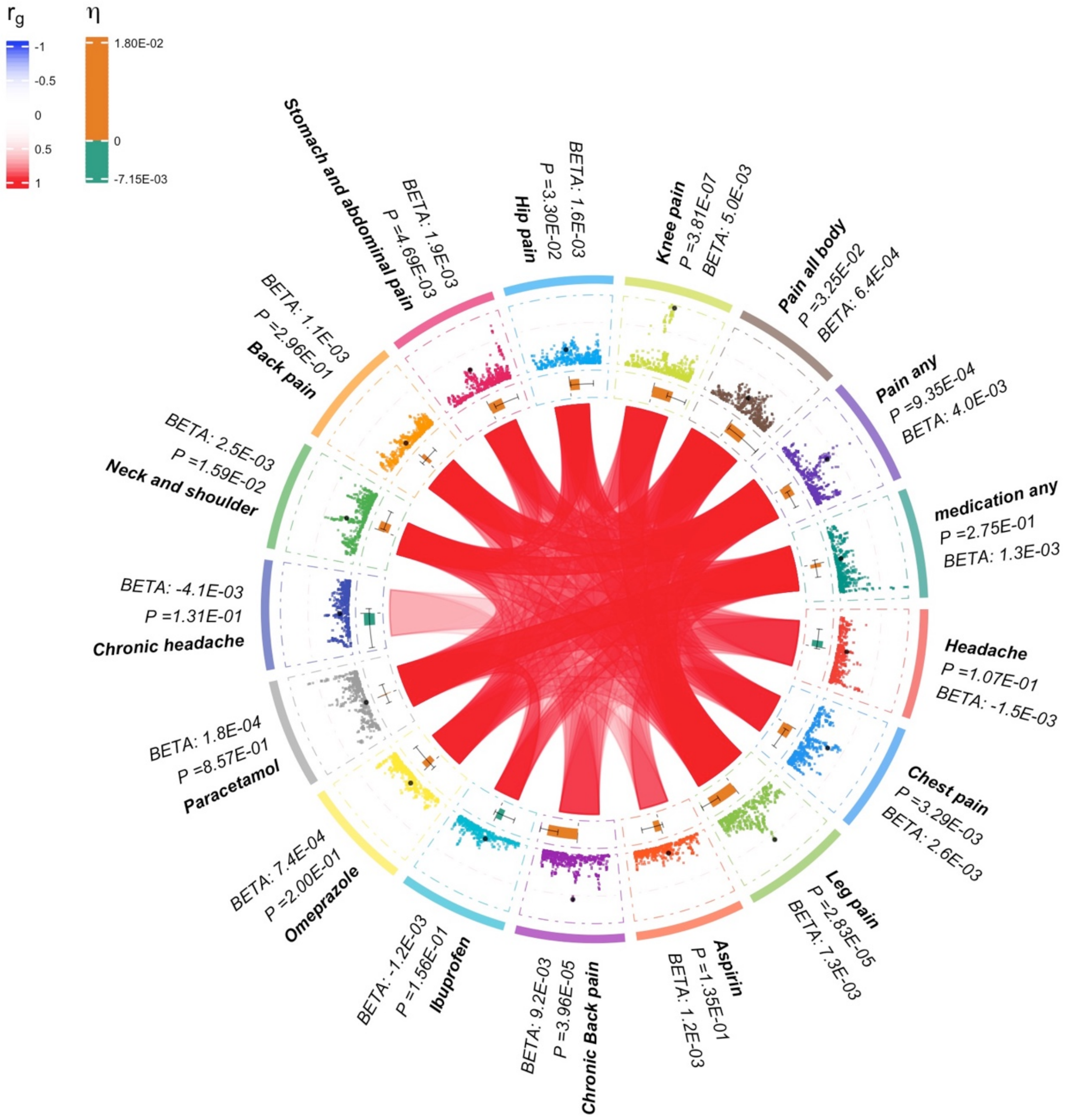

Locus16 2p23.3, rs12612492 Pleio-P= 1.58E-08, intronic to *ATAD2B* gene

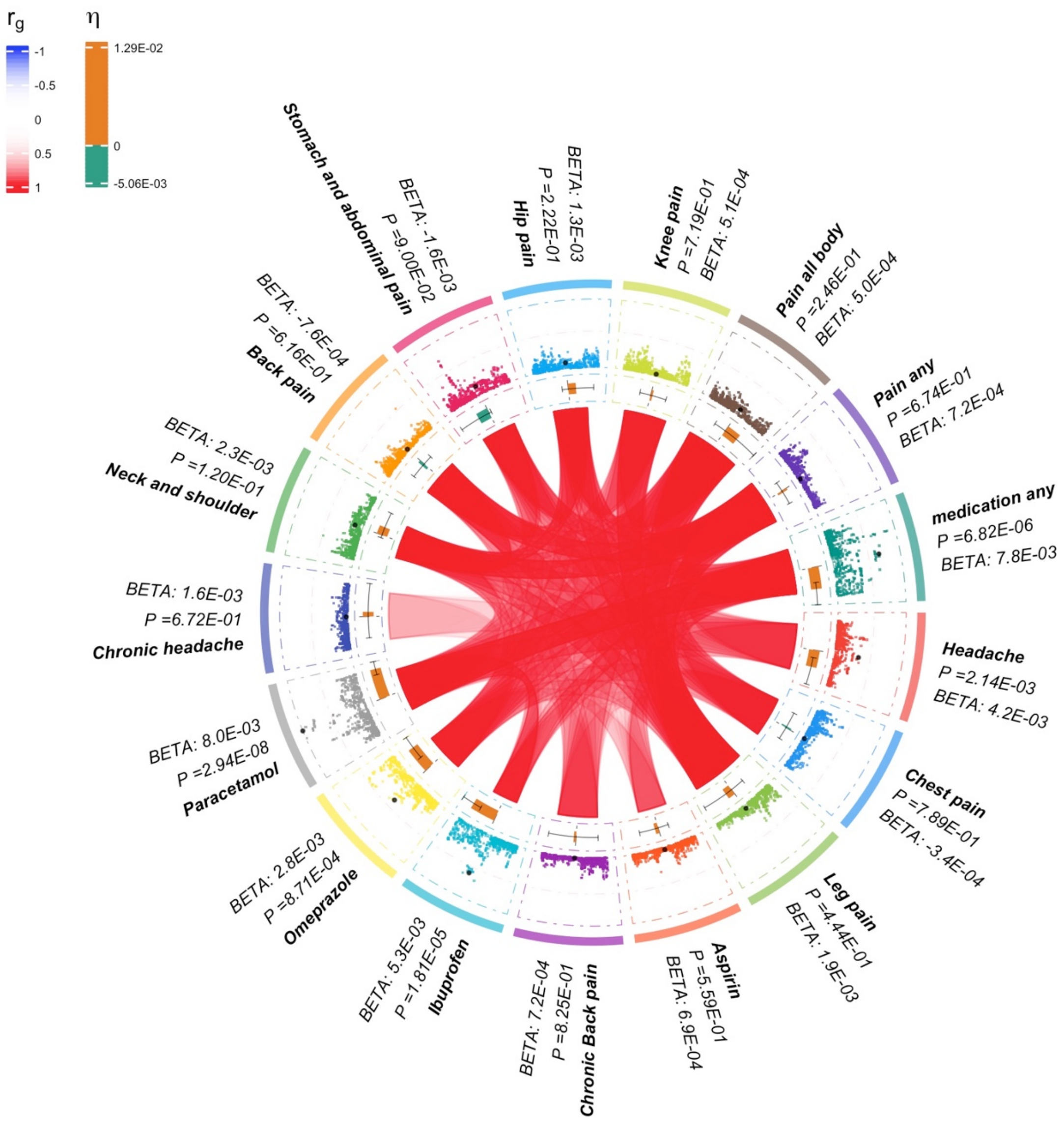

Locus17 2p16.3, rs3914722 Pleio-P= 1.83E-08, intronic to *NRXN1* gene

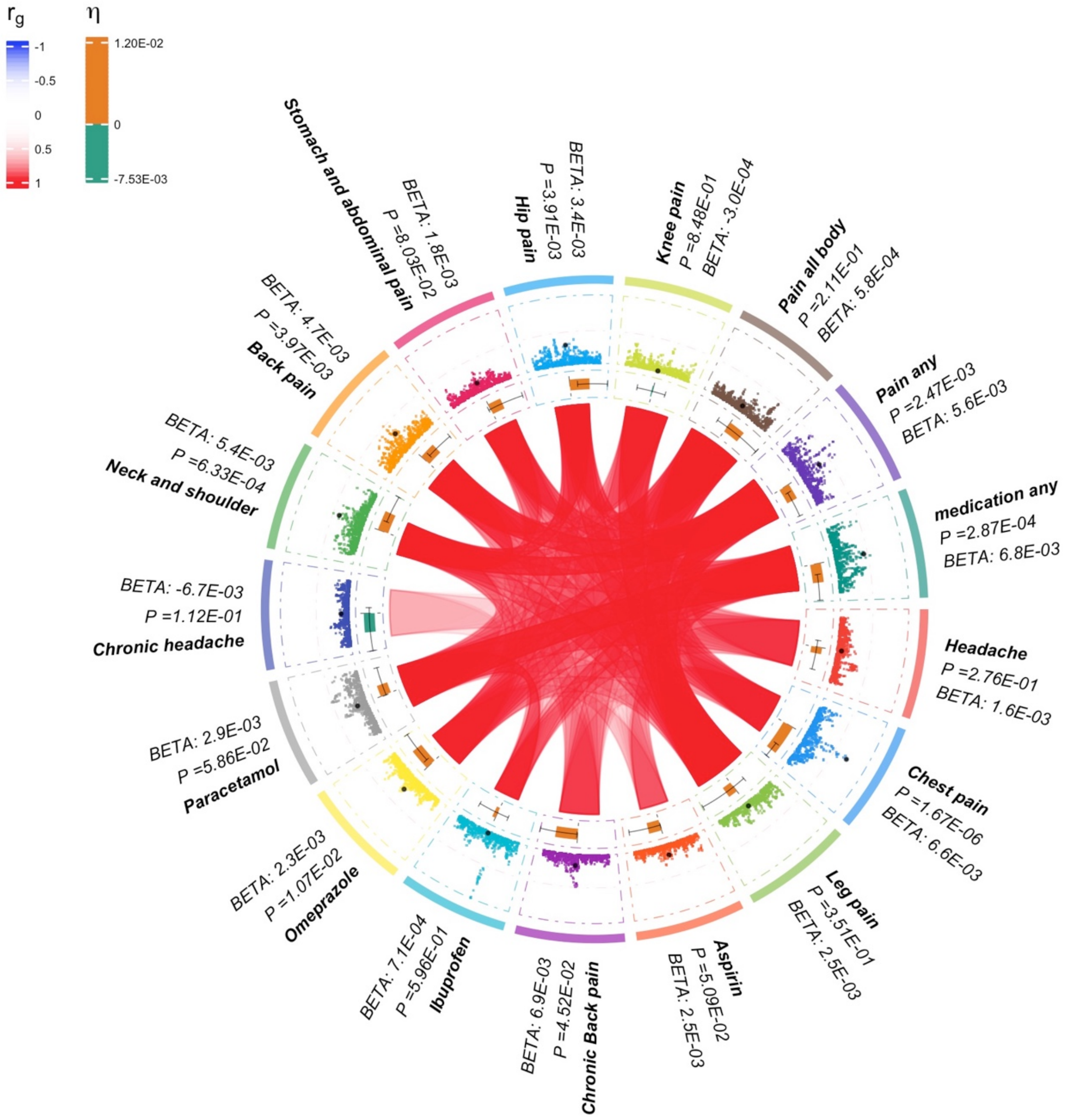

Locus18 2q11.2, rs4850917 Pleio-P= 2.62E-12, intronic to *AFF3* gene

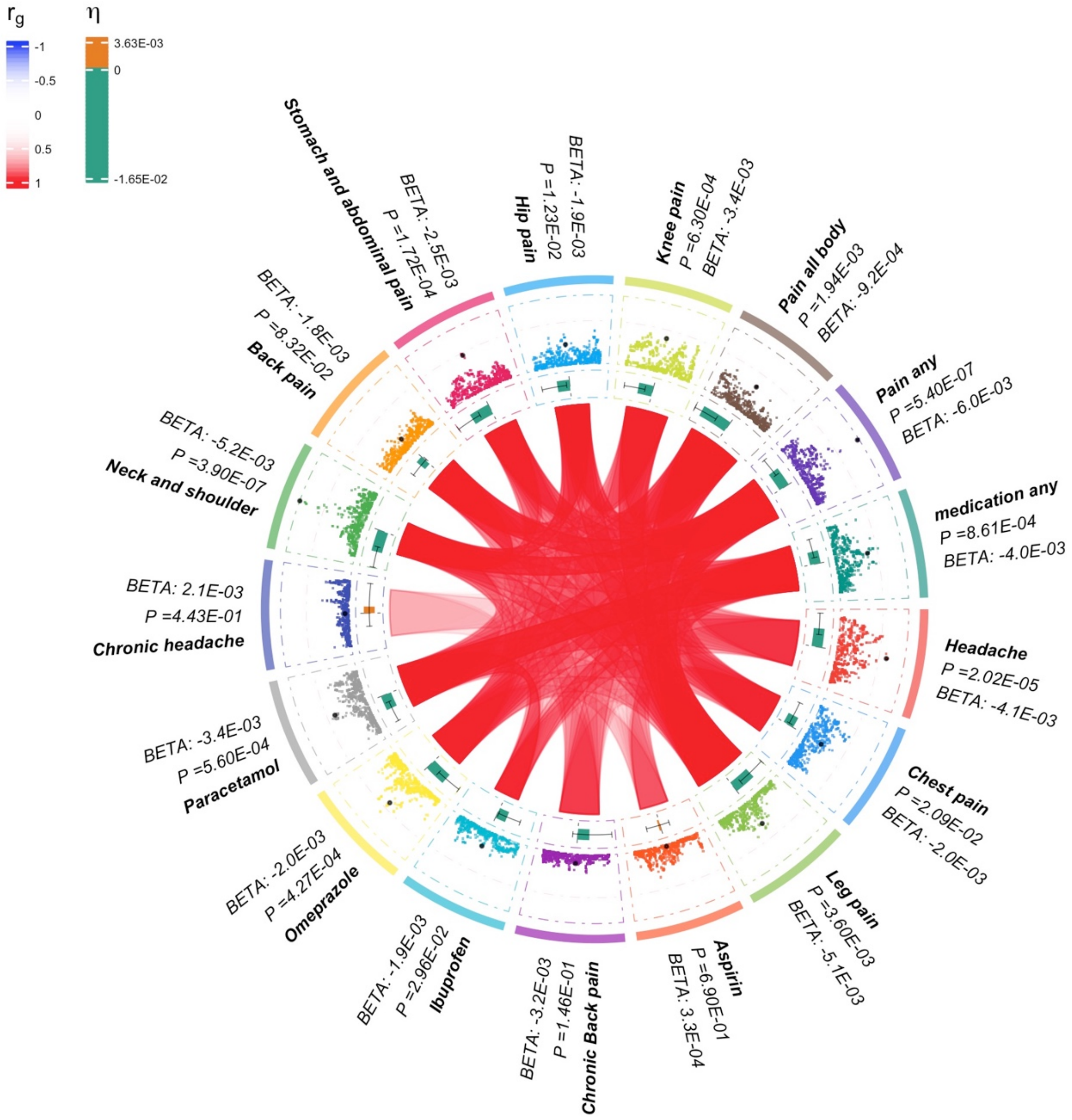

Locus19 2q12.1, rs17343925 Pleio-P= 1.74E-09, intergenic to AC013727.1 gene

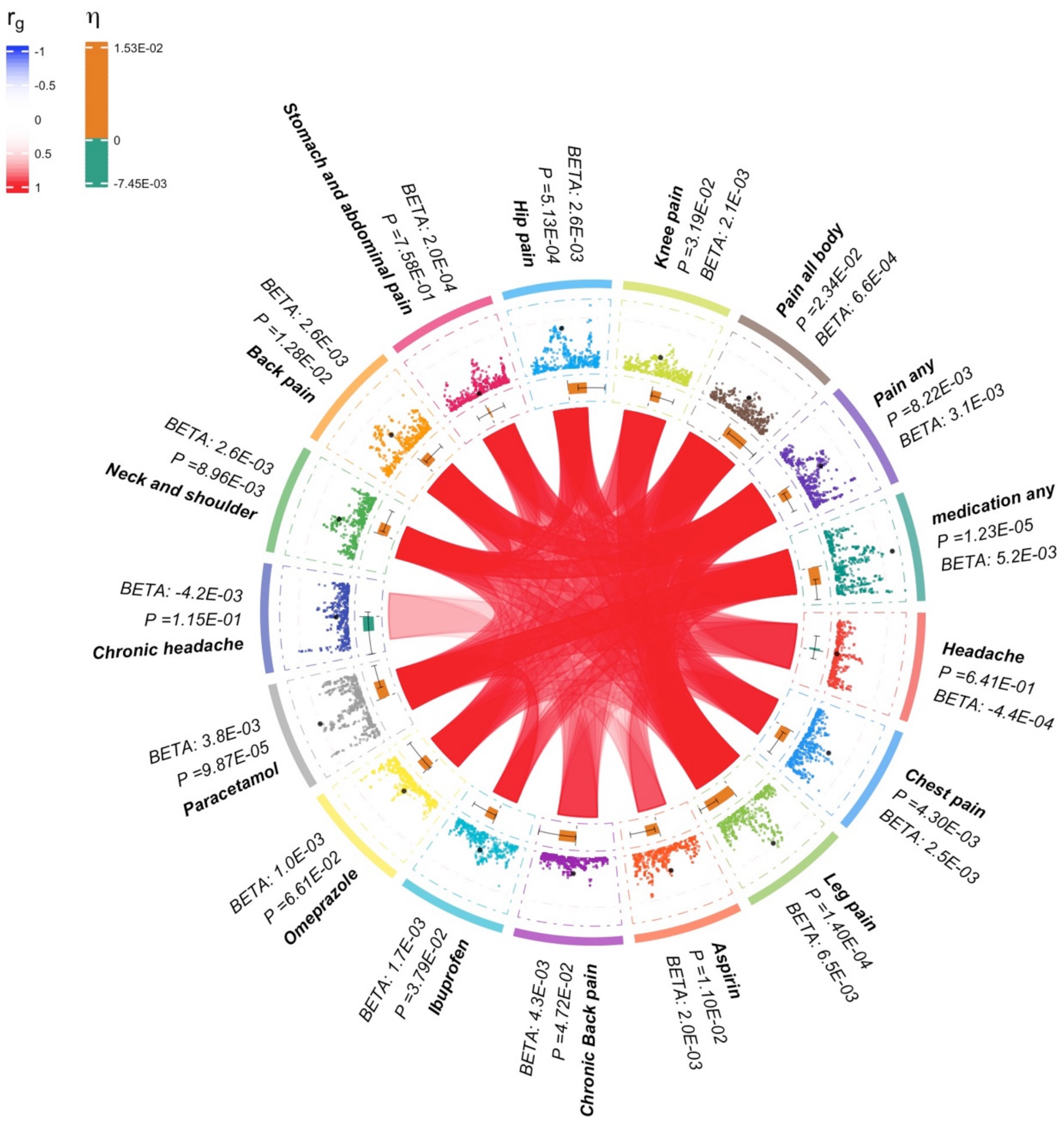

Locus20 2q33.3, rs2111592 Pleio-P= 1.25E-10, ncRNA_intronic to AC007879.1 gene

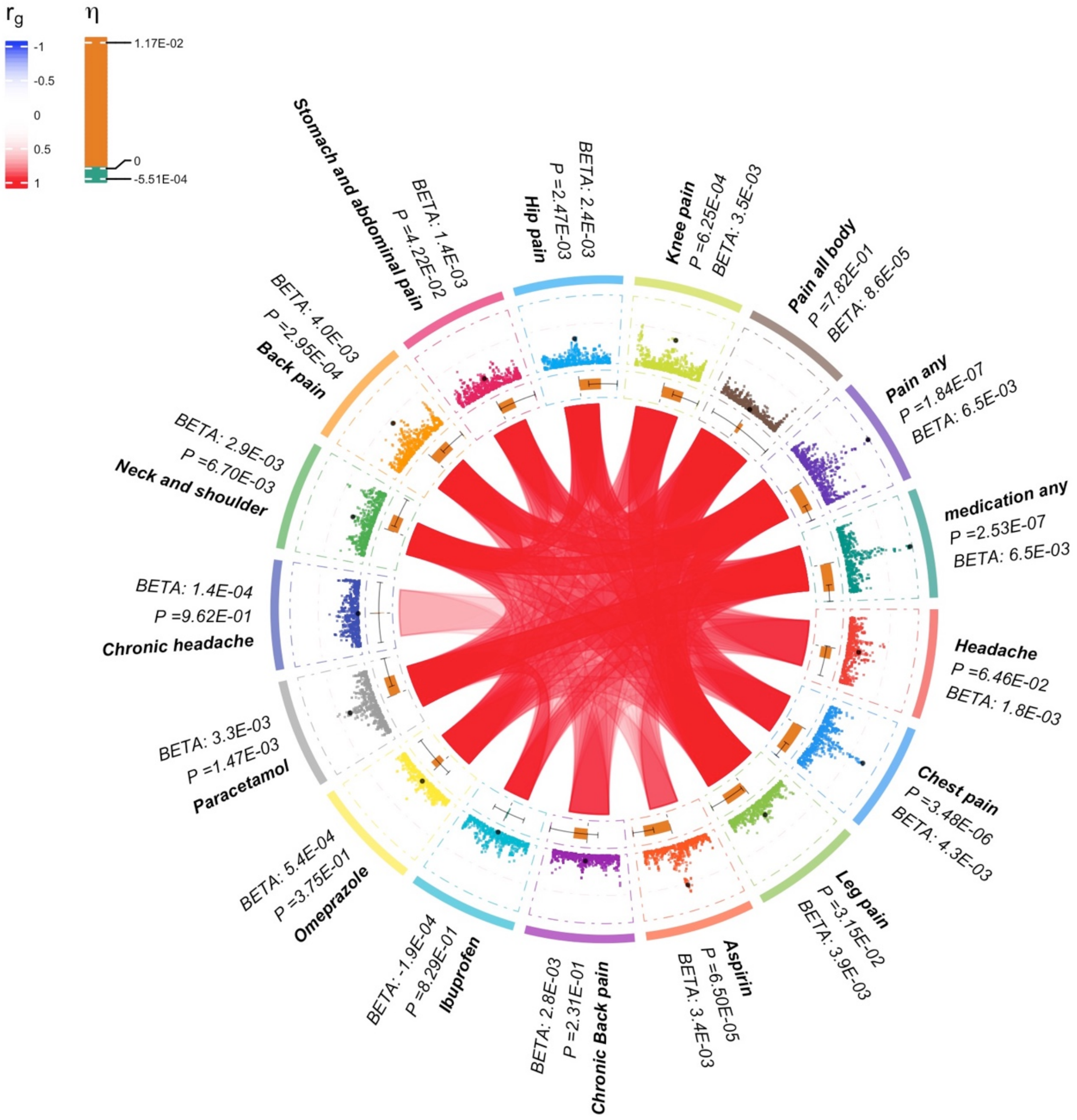

Locus21 2q34, rs17822981 Pleio-P= 5.23E-10, intronic to *CPS1* gene

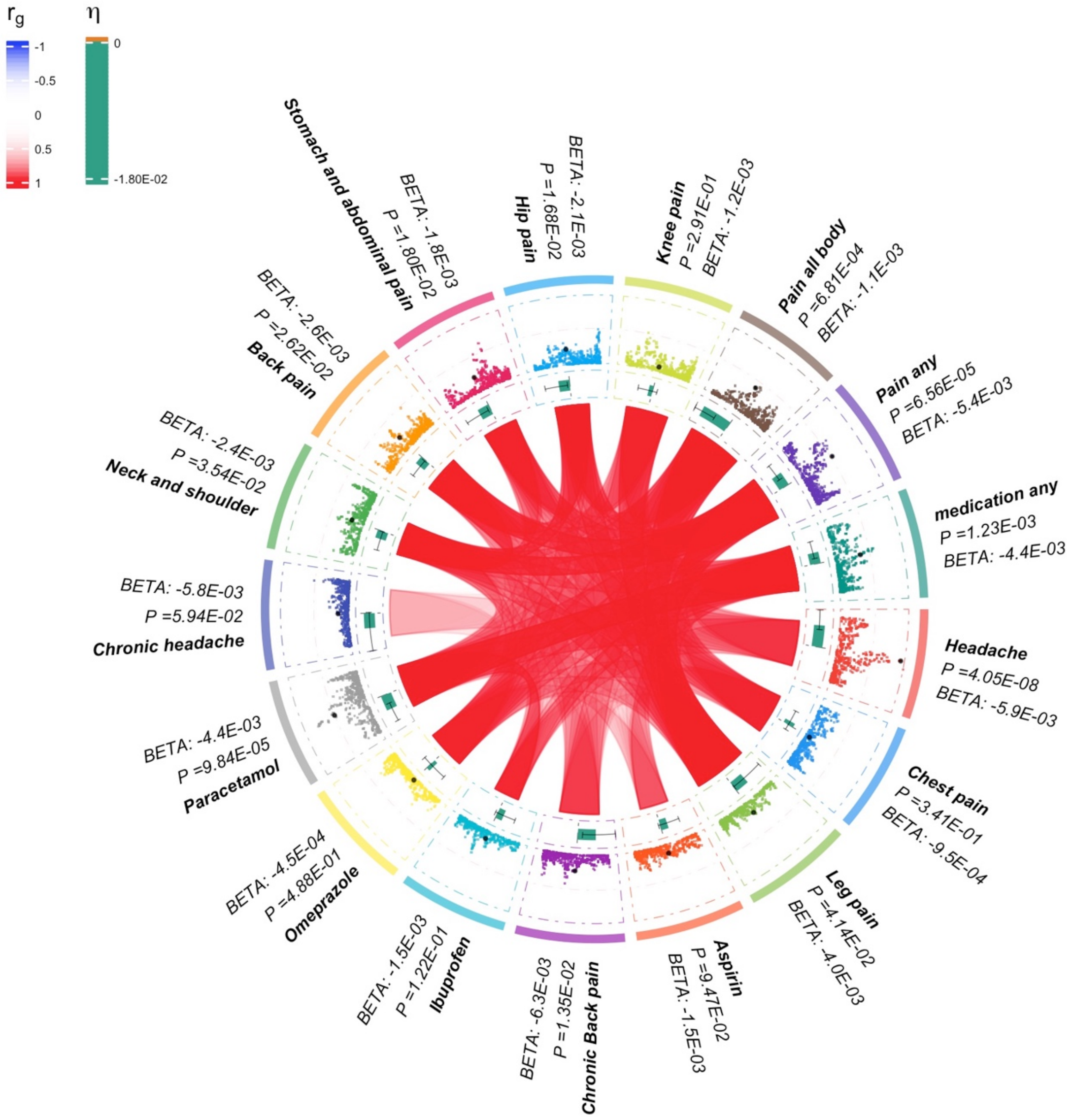

Locus22 2q37.1, rs10166942 Pleio-P= 3.78E-32, upstream to *TRPM8* gene

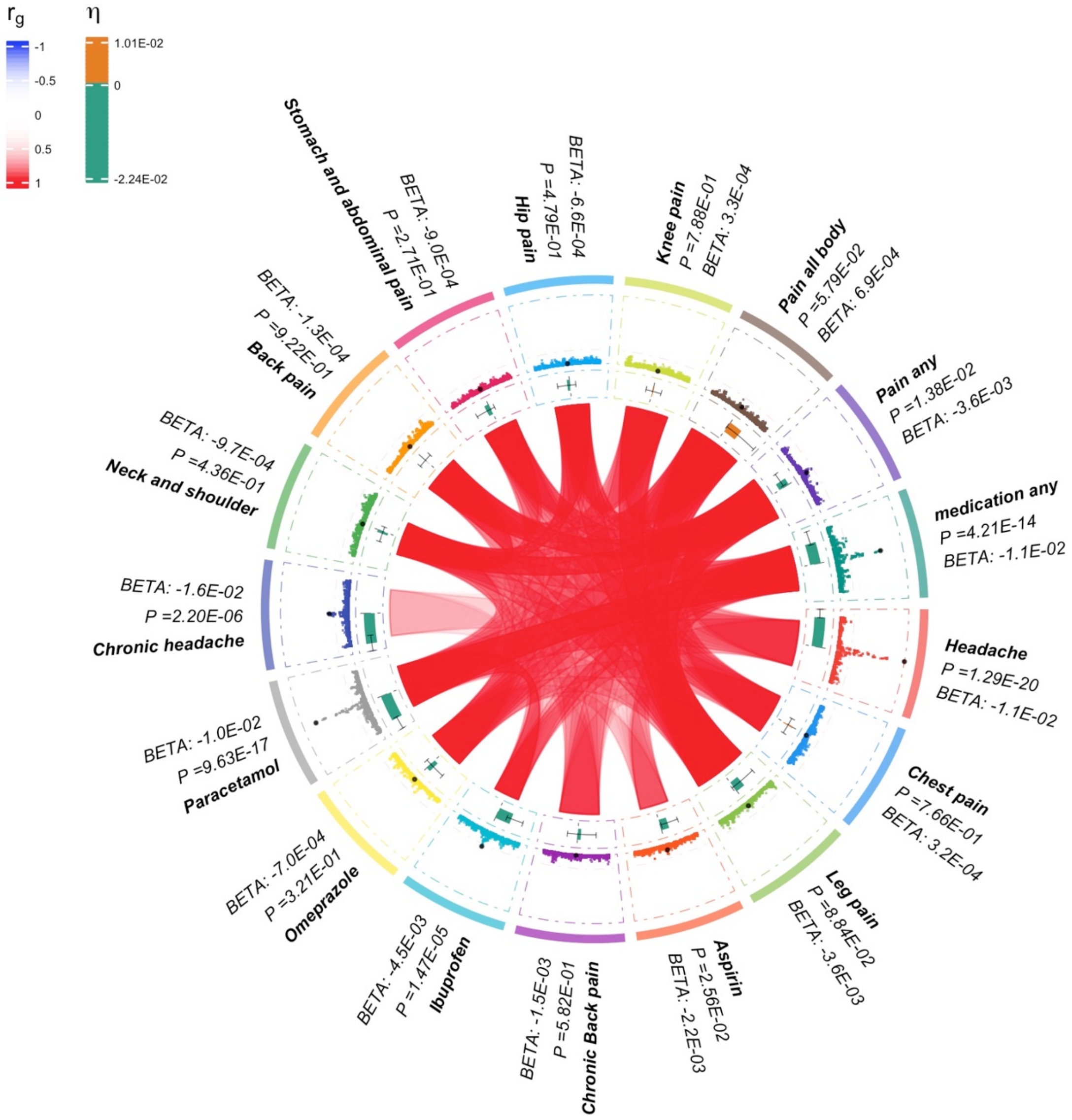

Locus23 3p24.1, rs6790925 Pleio-P= 3.06E-08, intergenic to AC116035.1 gene

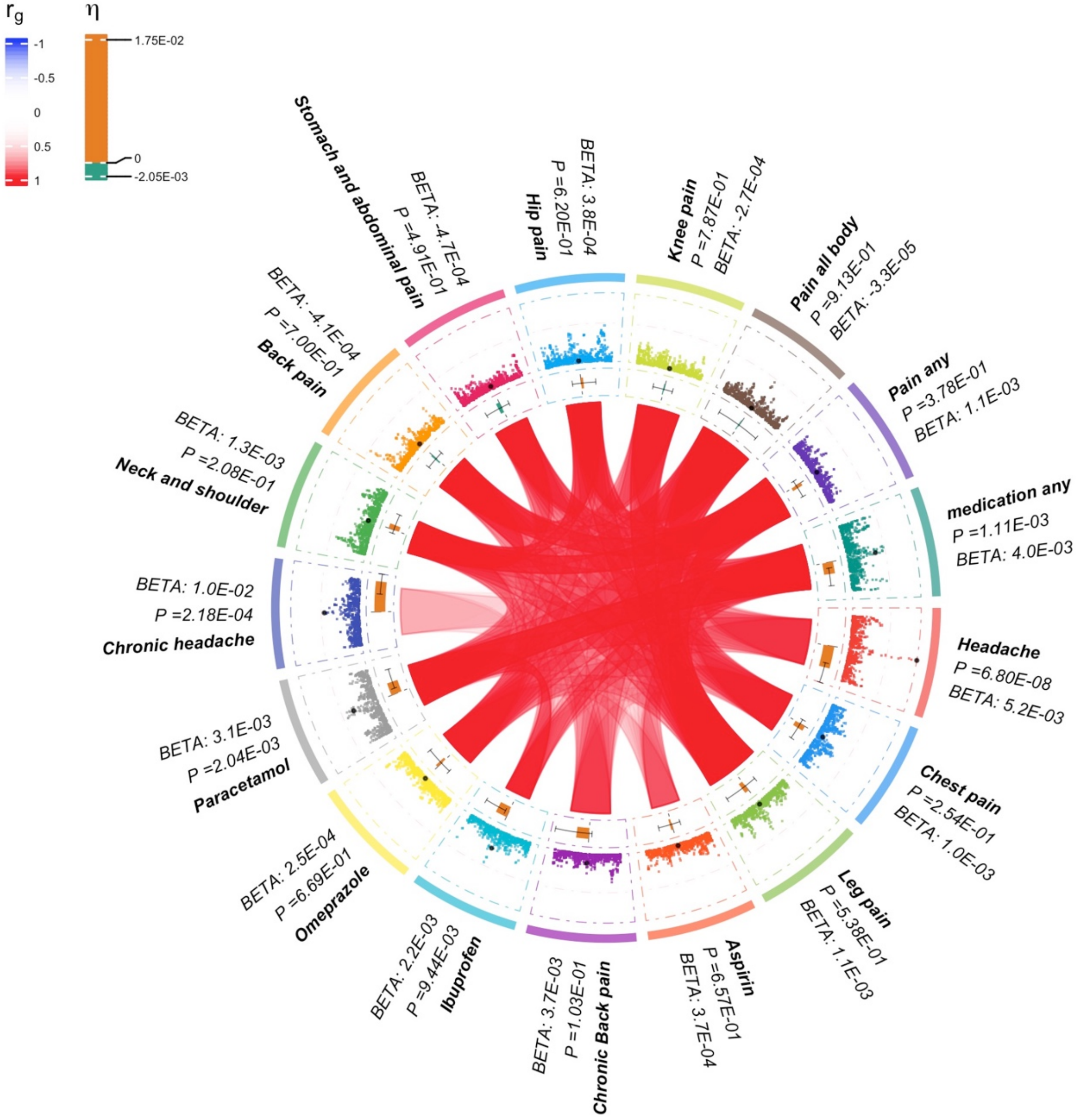

Locus24 3p21.31, rs2856238 Pleio-P= 1.37E-11, ncRNA_intronic to RP11-493K19.3 gene

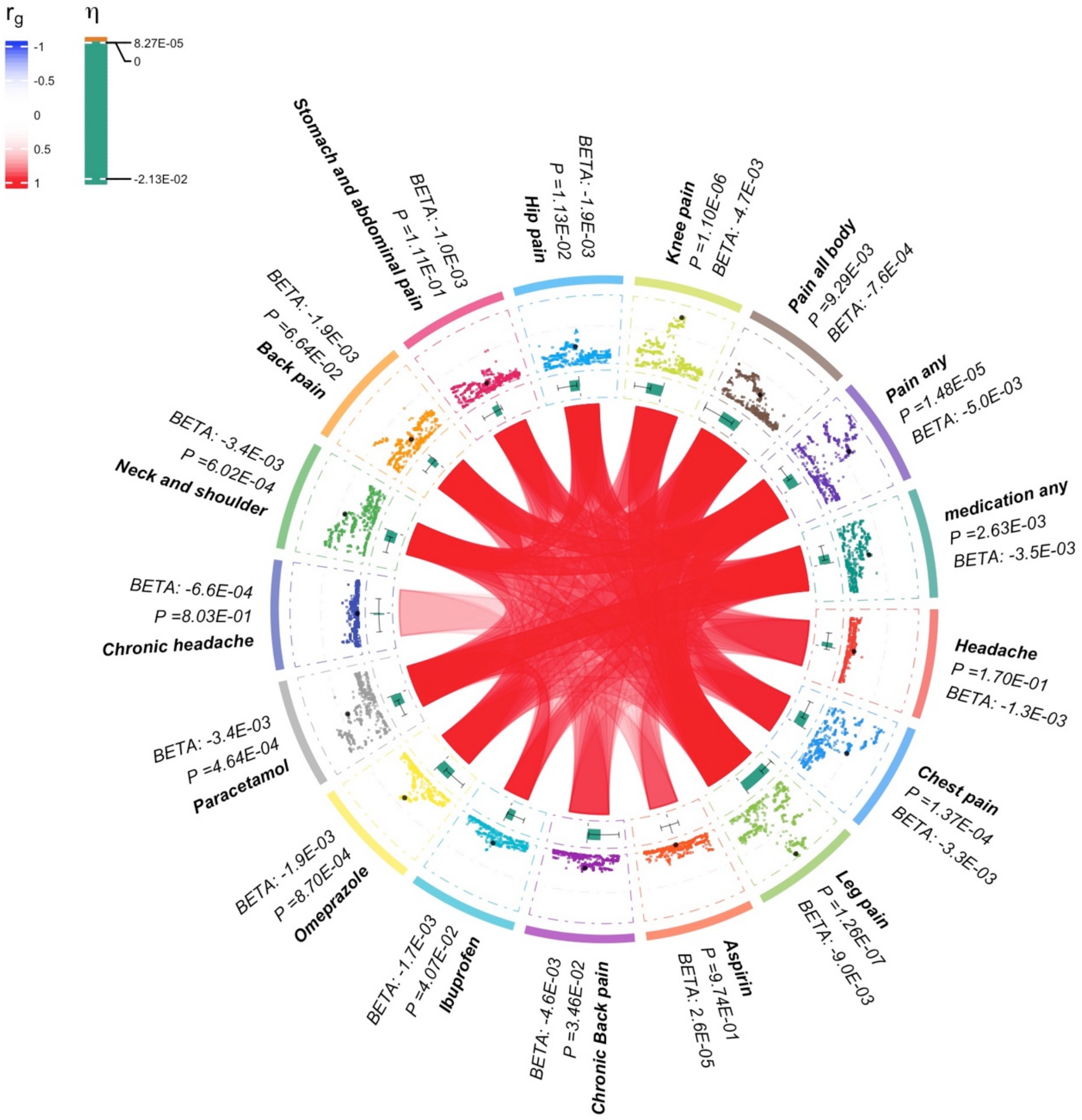

Locus25 3p12.3, rs775760 Pleio-P= 1.19E-09, intronic to *ROBO2* gene

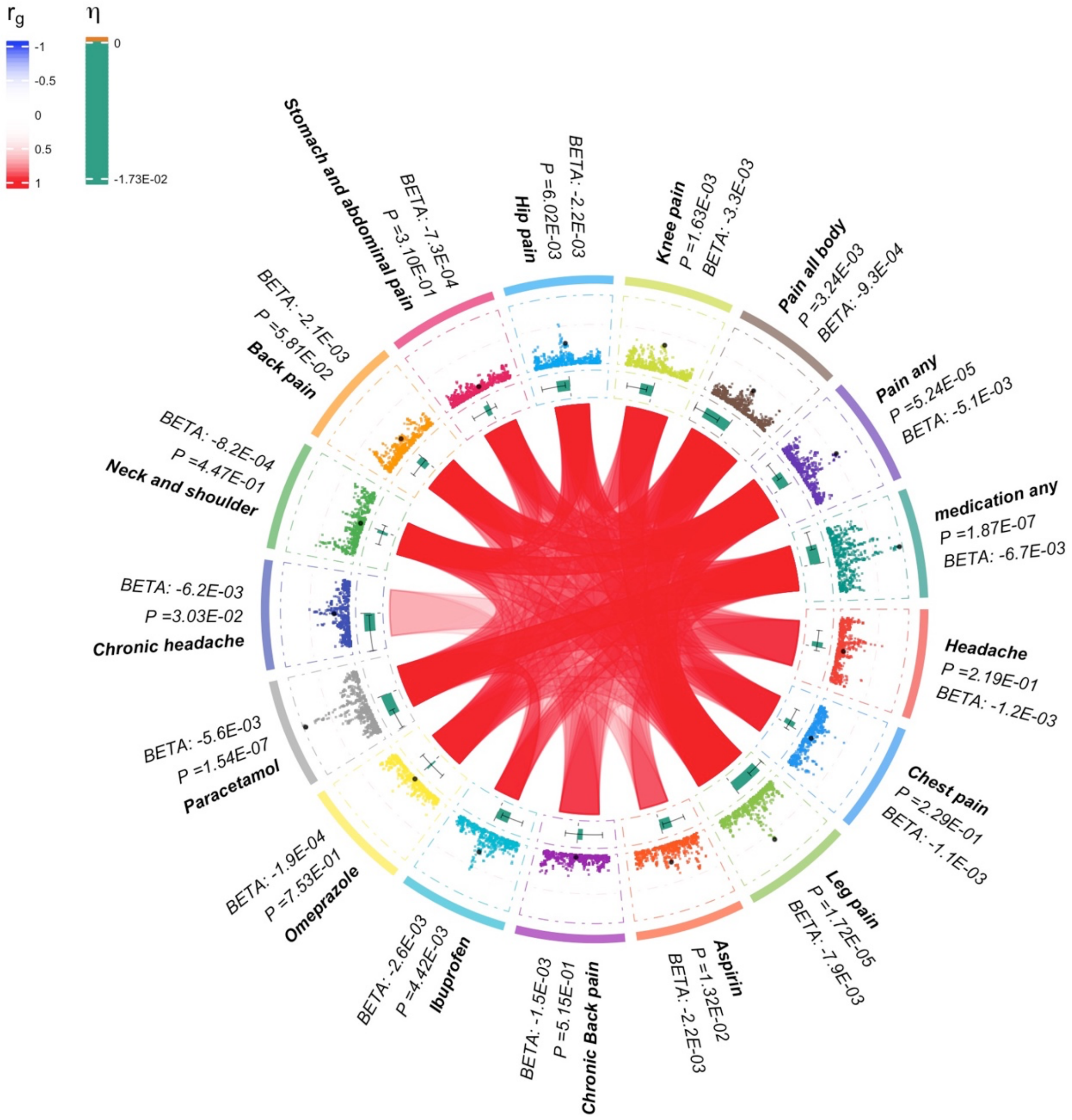

Locus26 3q26.31, rs583514 Pleio-P= 1.26E-11, UTR5 to *NLGN1* gene

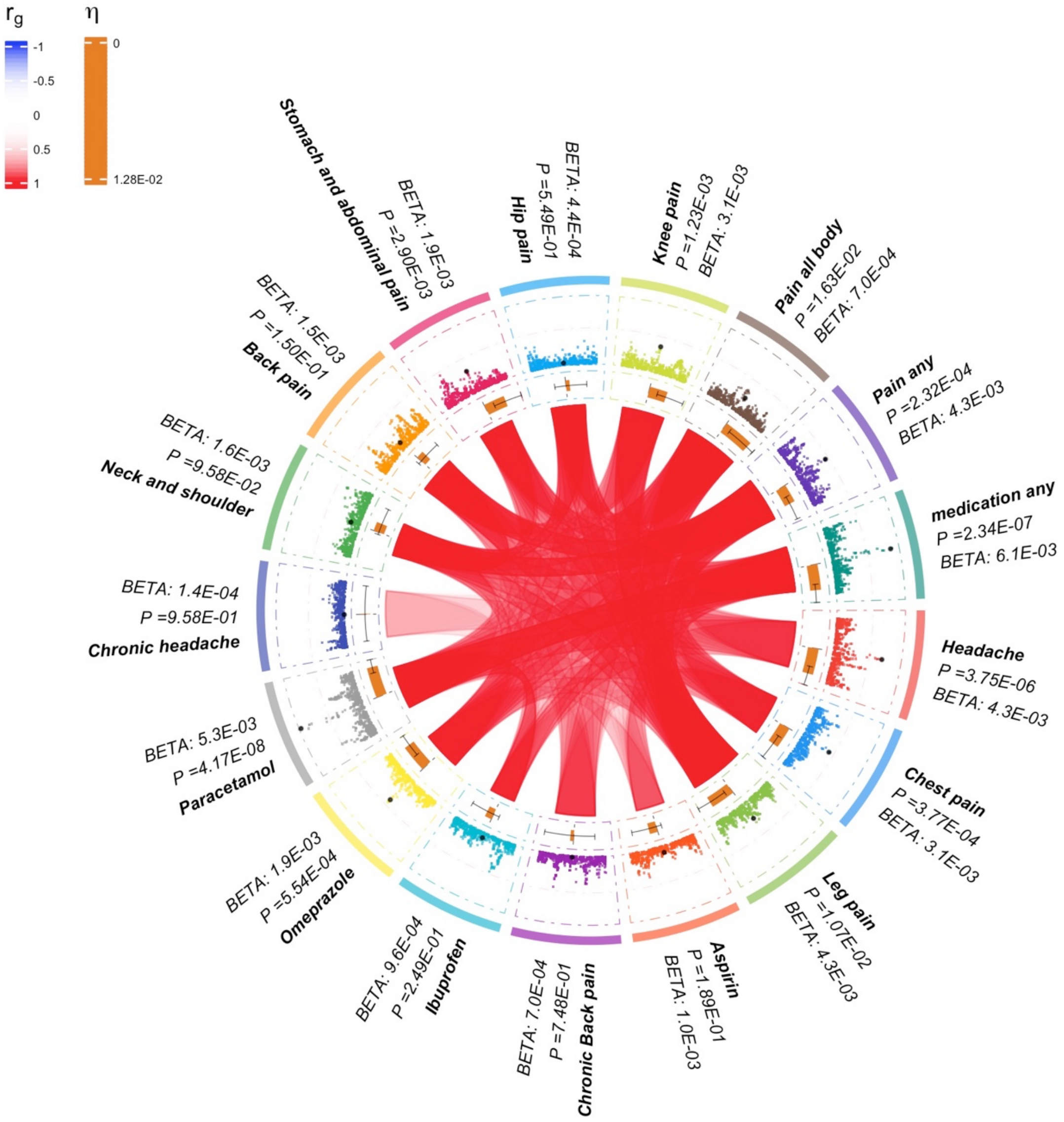

Locus27 4p16.3, rs3021146 Pleio-P= 7.86E-09, ncRNA_intronic to *NOP14-AS1:NOP14* gene

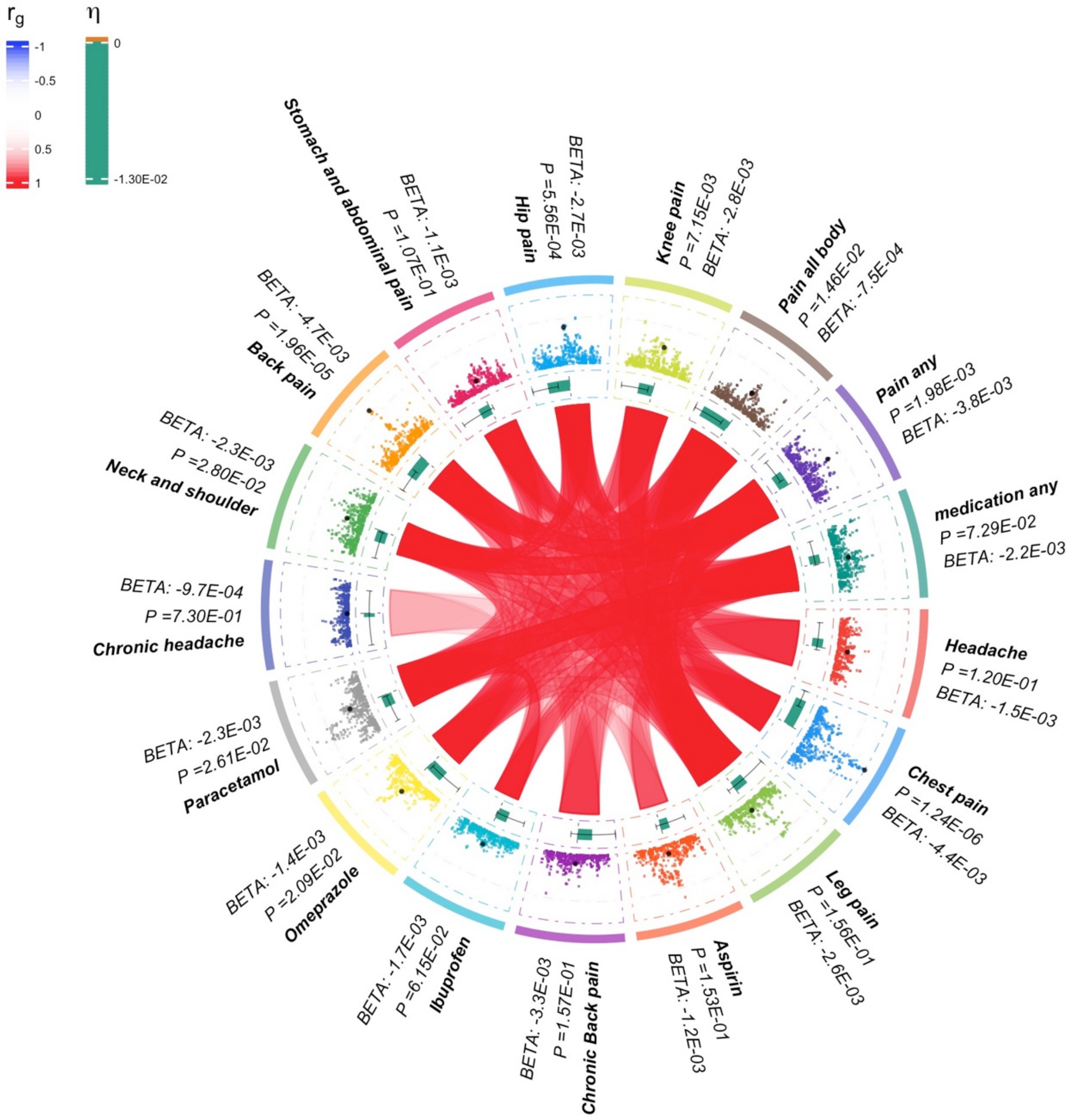

Locus28 4q12, rs7684253 Pleio-P= 3.87E-09, intergenic to *SPINK2* gene

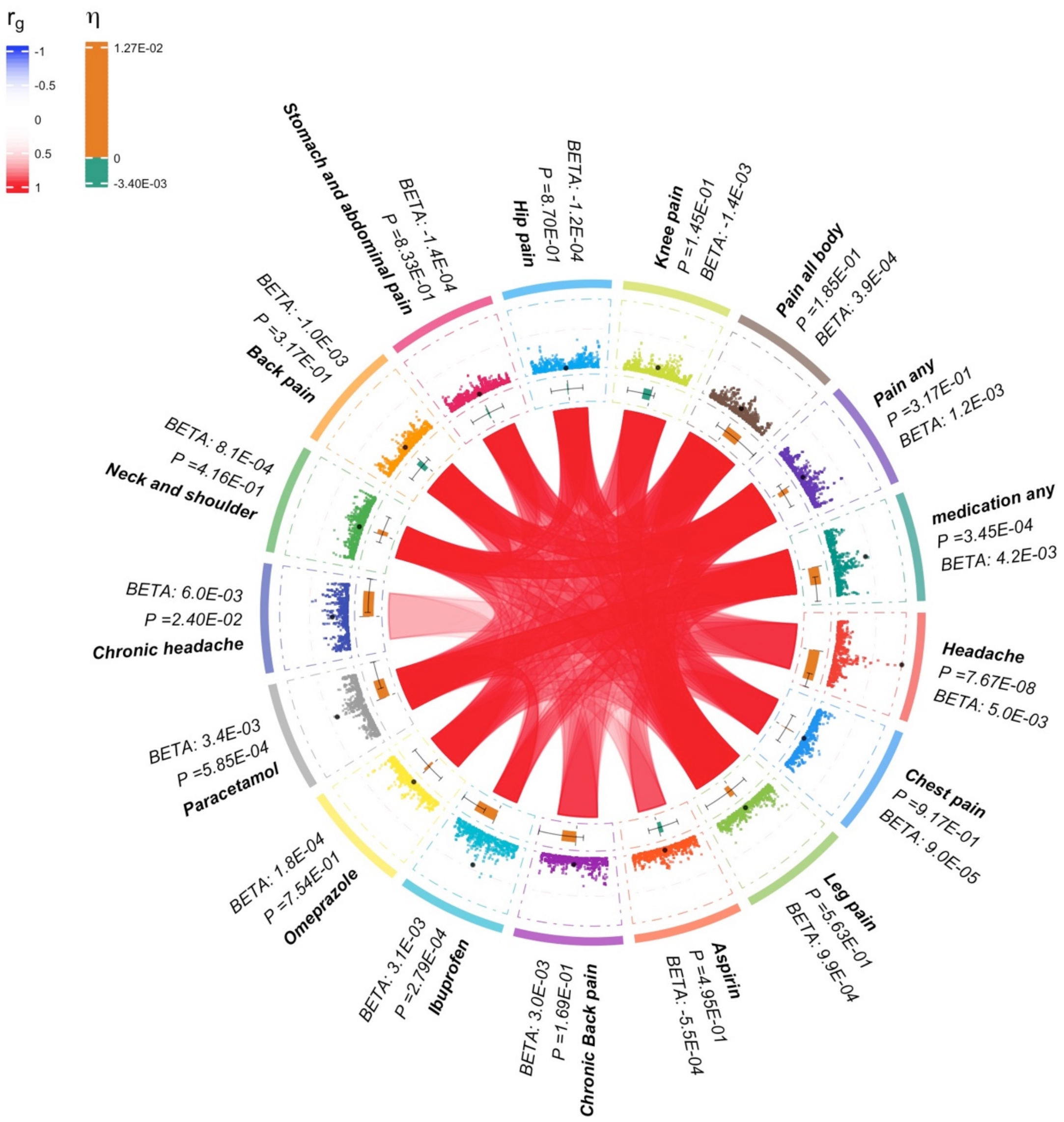

Locus29 4q13.2, rs4860809 Pleio-P= 2.43E-08, intergenic to *RNU6-699P* gene

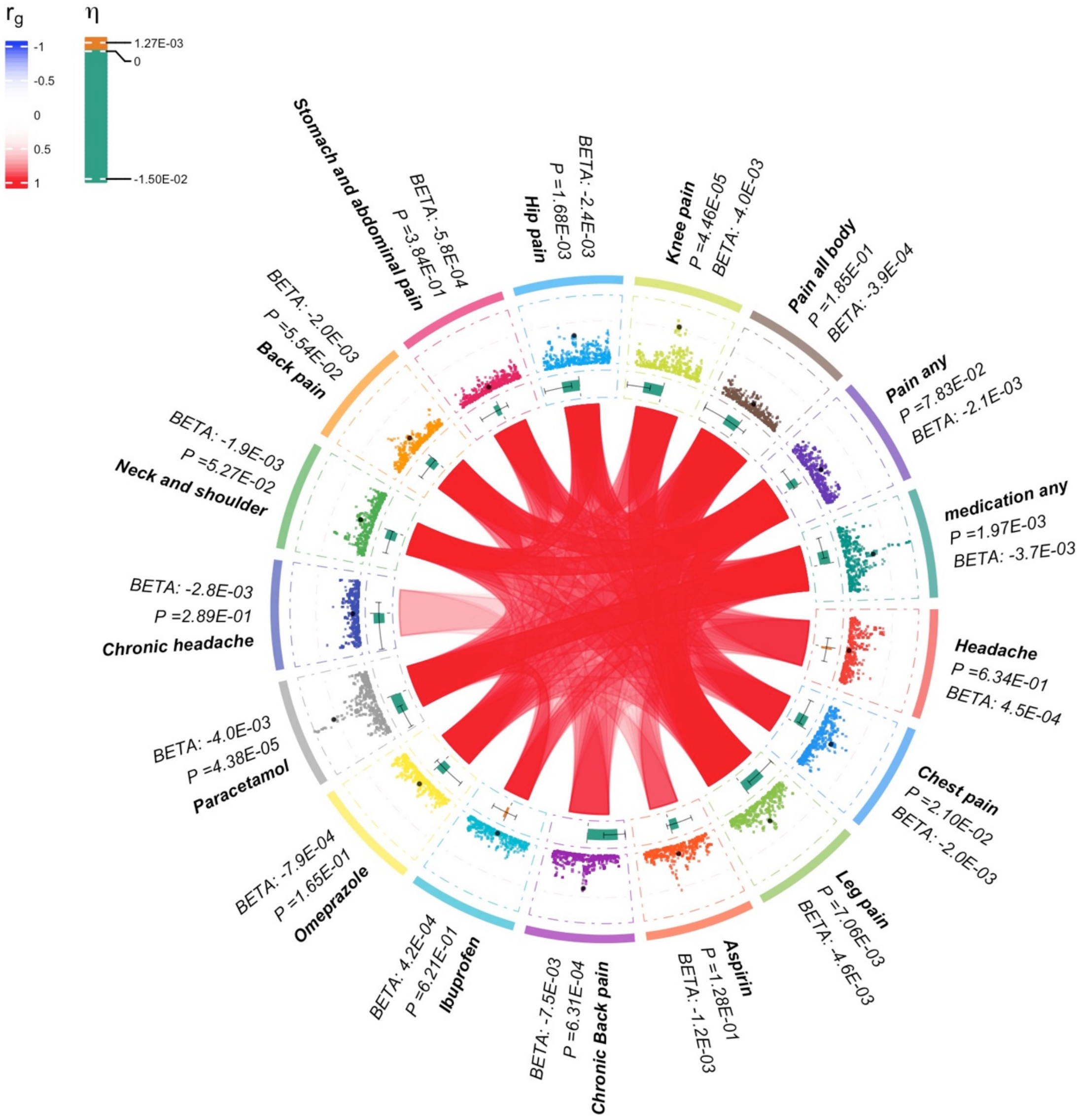

Locus30 4q24, rs13114738 Pleio-P= 1.18E-08, intergenic to *SLC39A8* gene

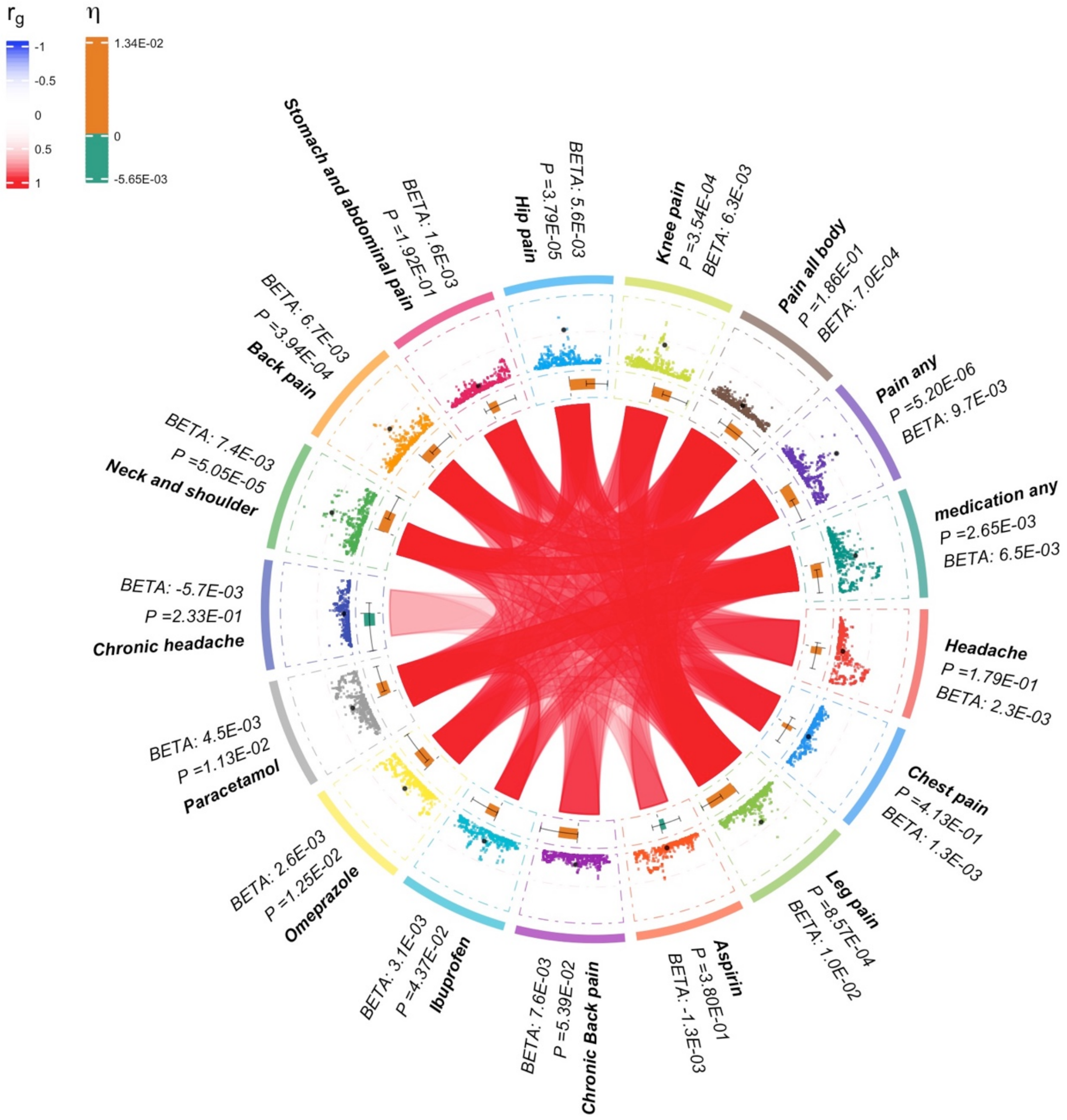

Locus31 4q25, rs11729080 Pleio-P= 6.34E-10, intergenic to RP11-255I10.1 gene

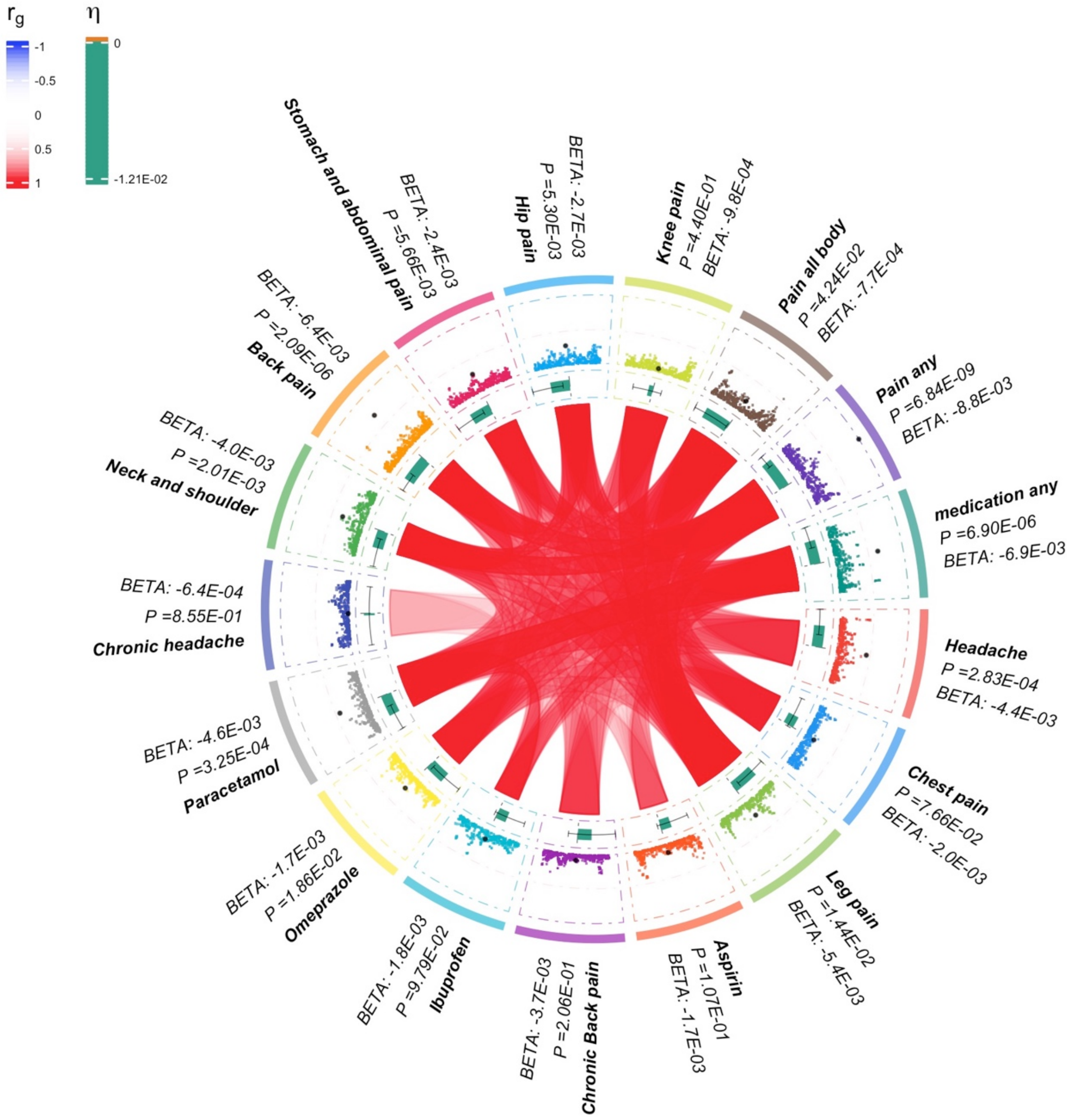

Locus32 5p13.3, rs4502841 Pleio-P= 4.55E-13, intergenic to RPL19P11 gene

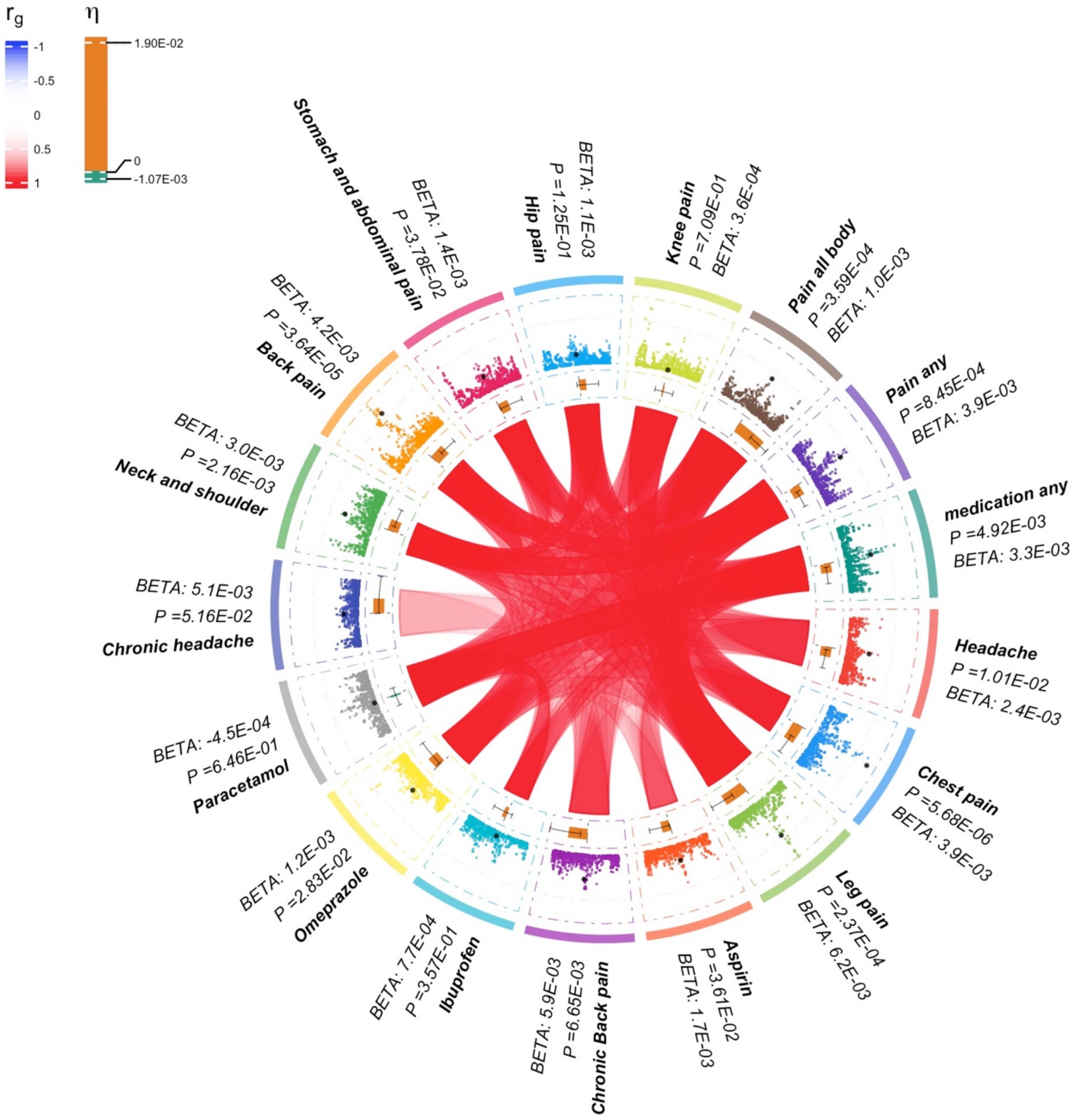

Locus33 5q22.3, rs554843 Pleio-P= 2.91E-08, intergenic to *KCNN2* gene

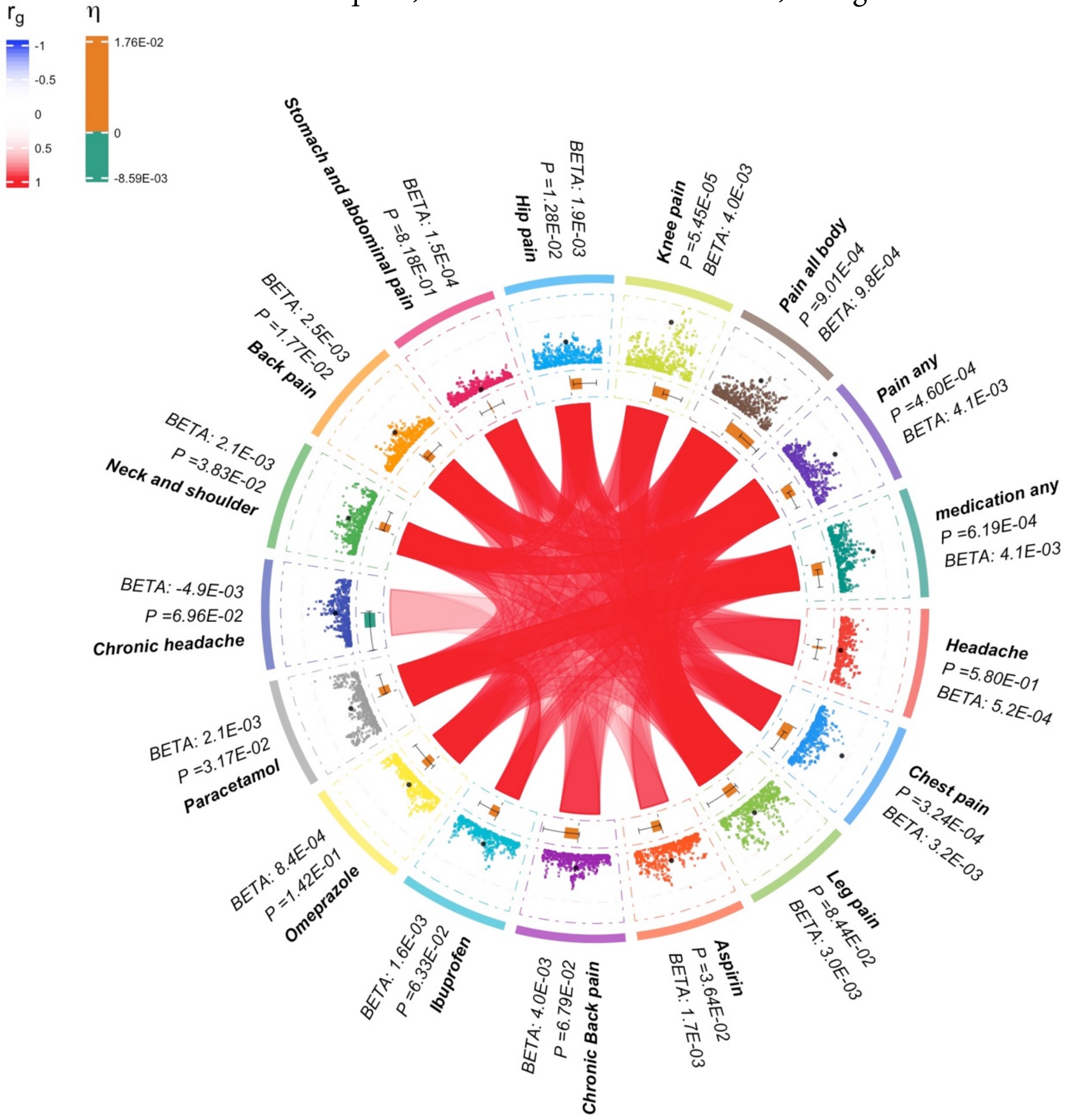

Locus34 5q23.1, rs7716347 Pleio-P= 4.56E-08, intergenic to CTD-2334D19.1 gene

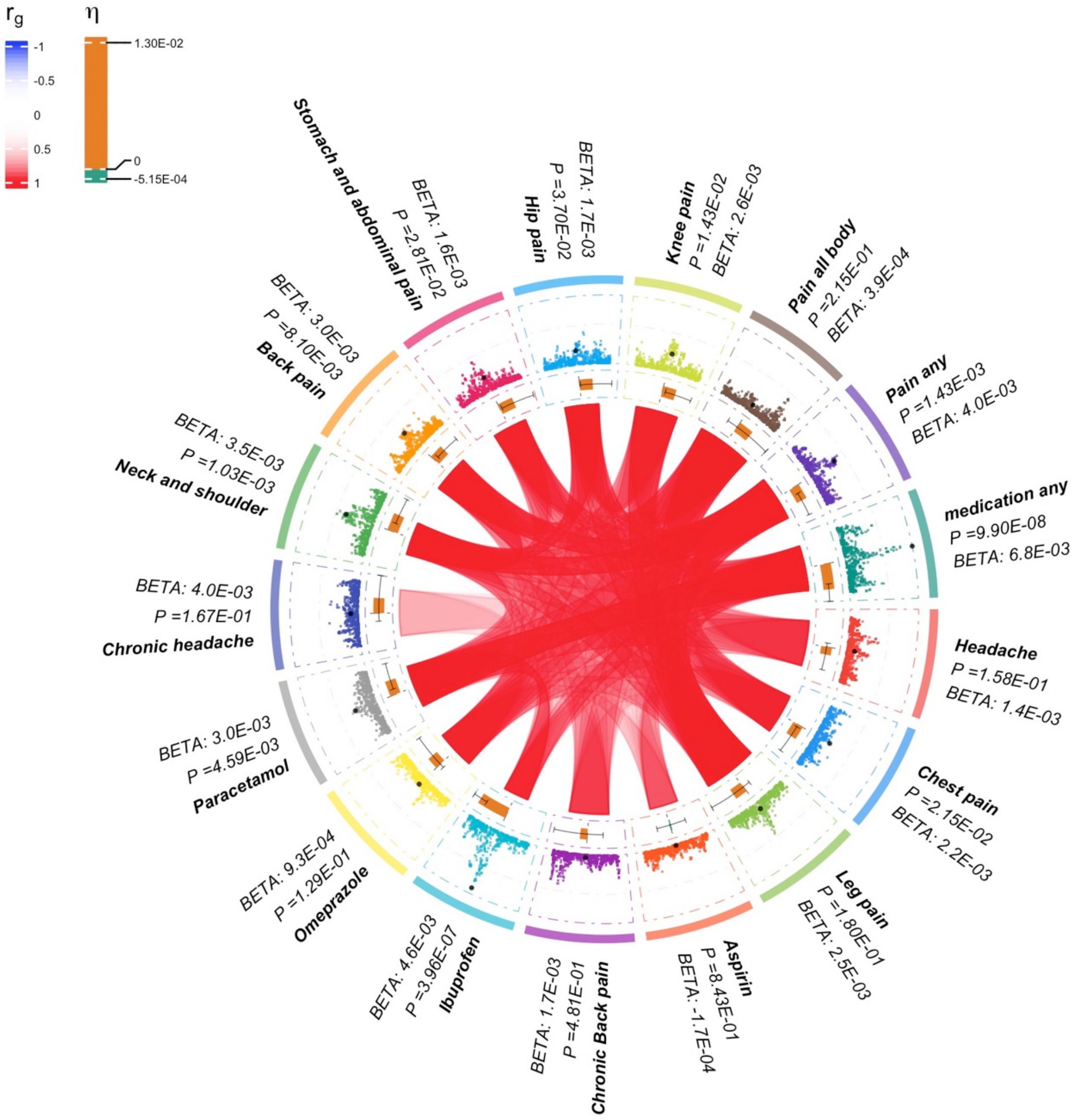

Locus35 5q35.3, rs10063803 Pleio-P= 1.34E-08, intronic to *NSD1* gene

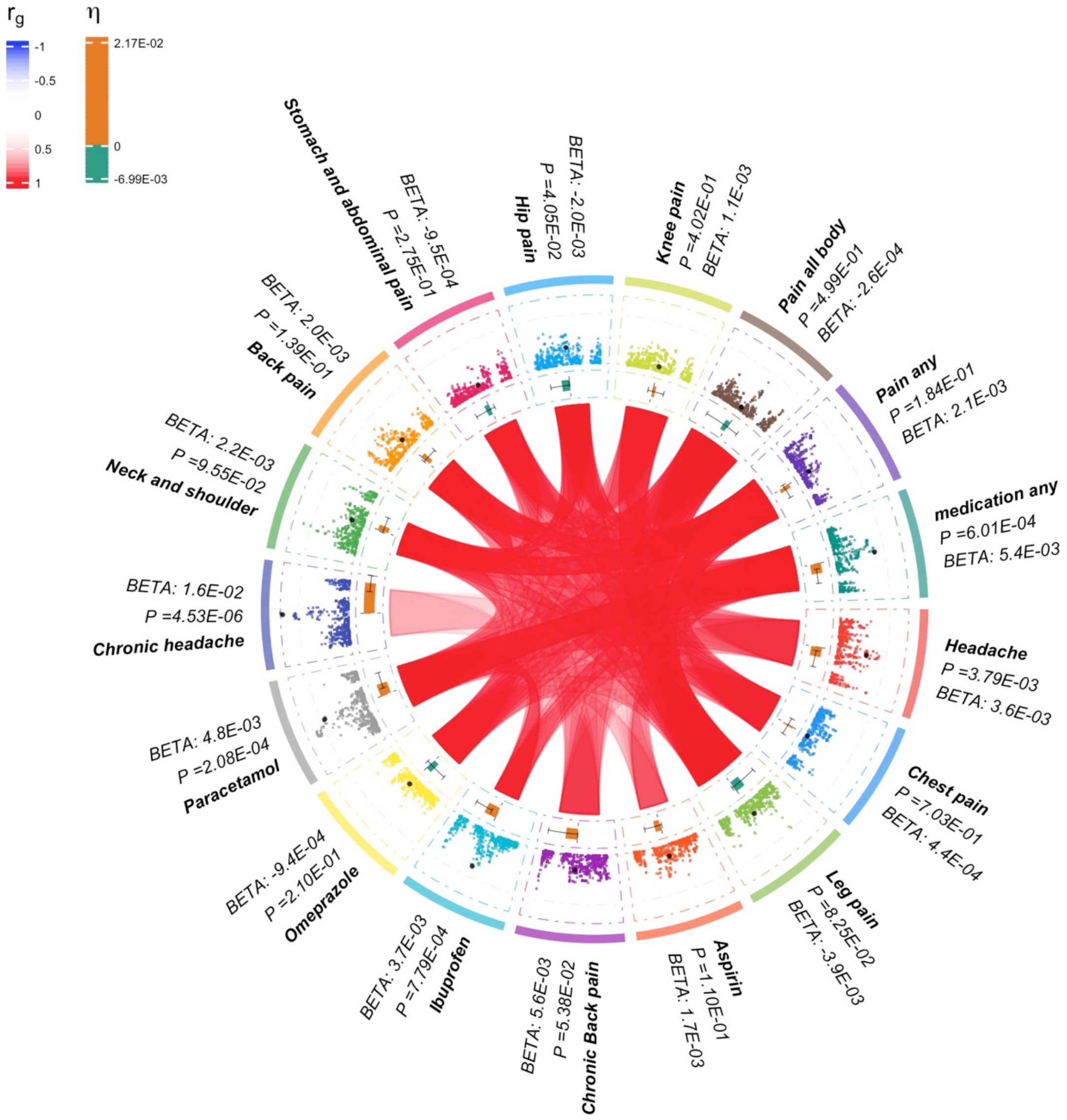

Locus36 6p24.1, rs9349379 Pleio-P= 4.22E-28, intronic to *PHACTR1* gene

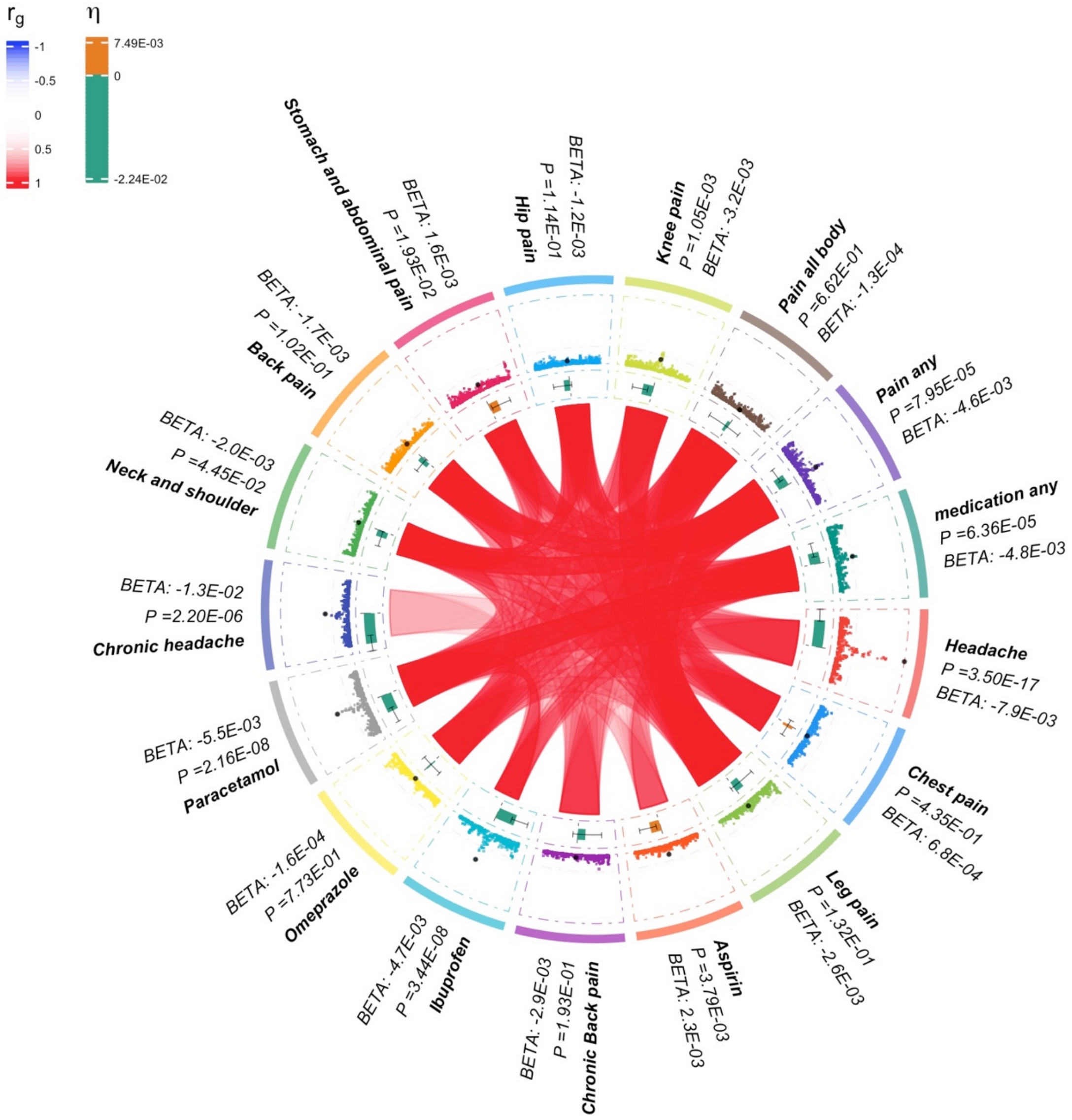

Locus37 6p22.1, rs13207082 Pleio-P= 4.34E-21, intergenic to *POM121L2* gene

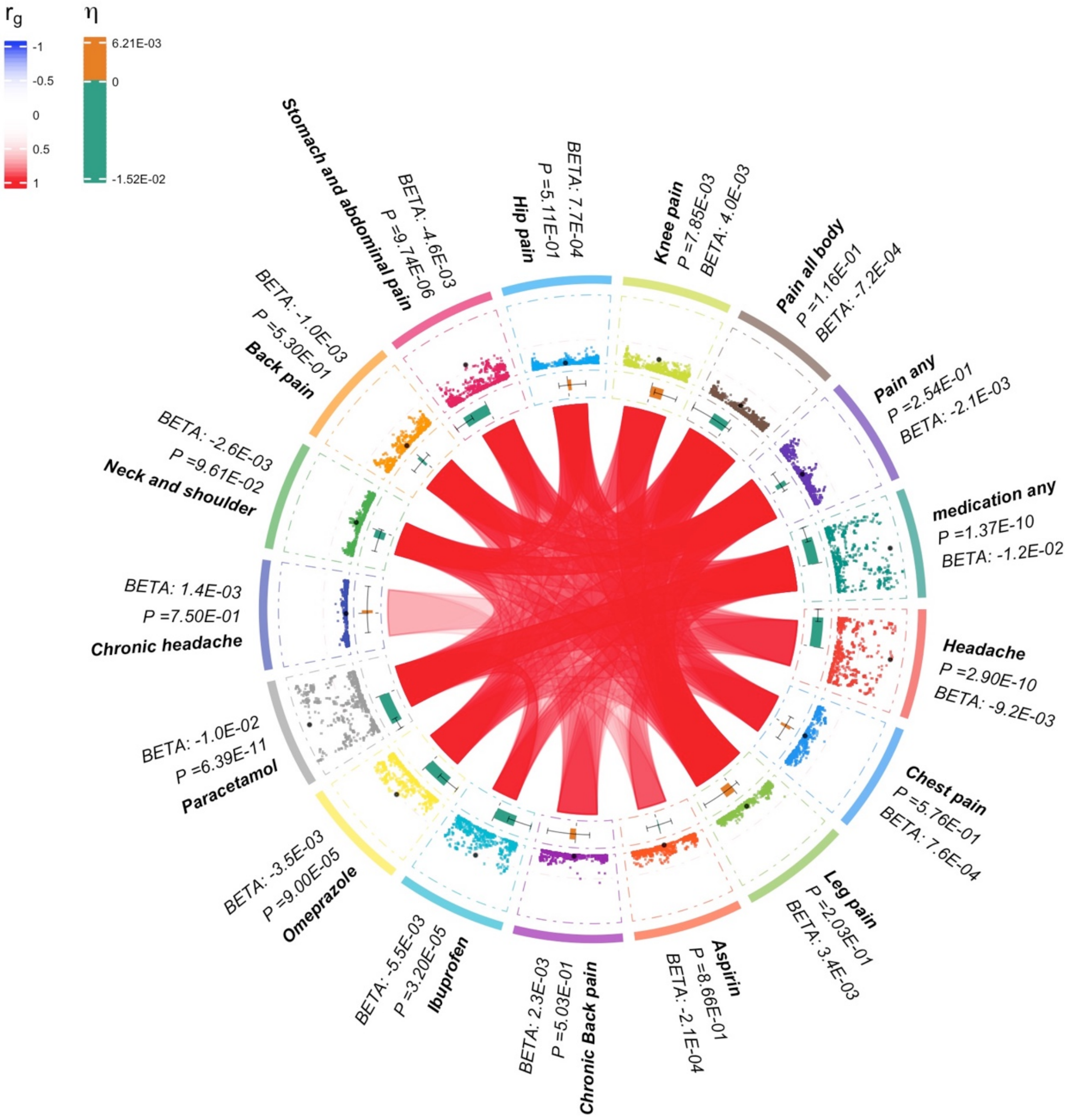

Locus38 6p21.31, rs11751469 Pleio-P= 2.32E-11, intergenic to *LINC01016* gene

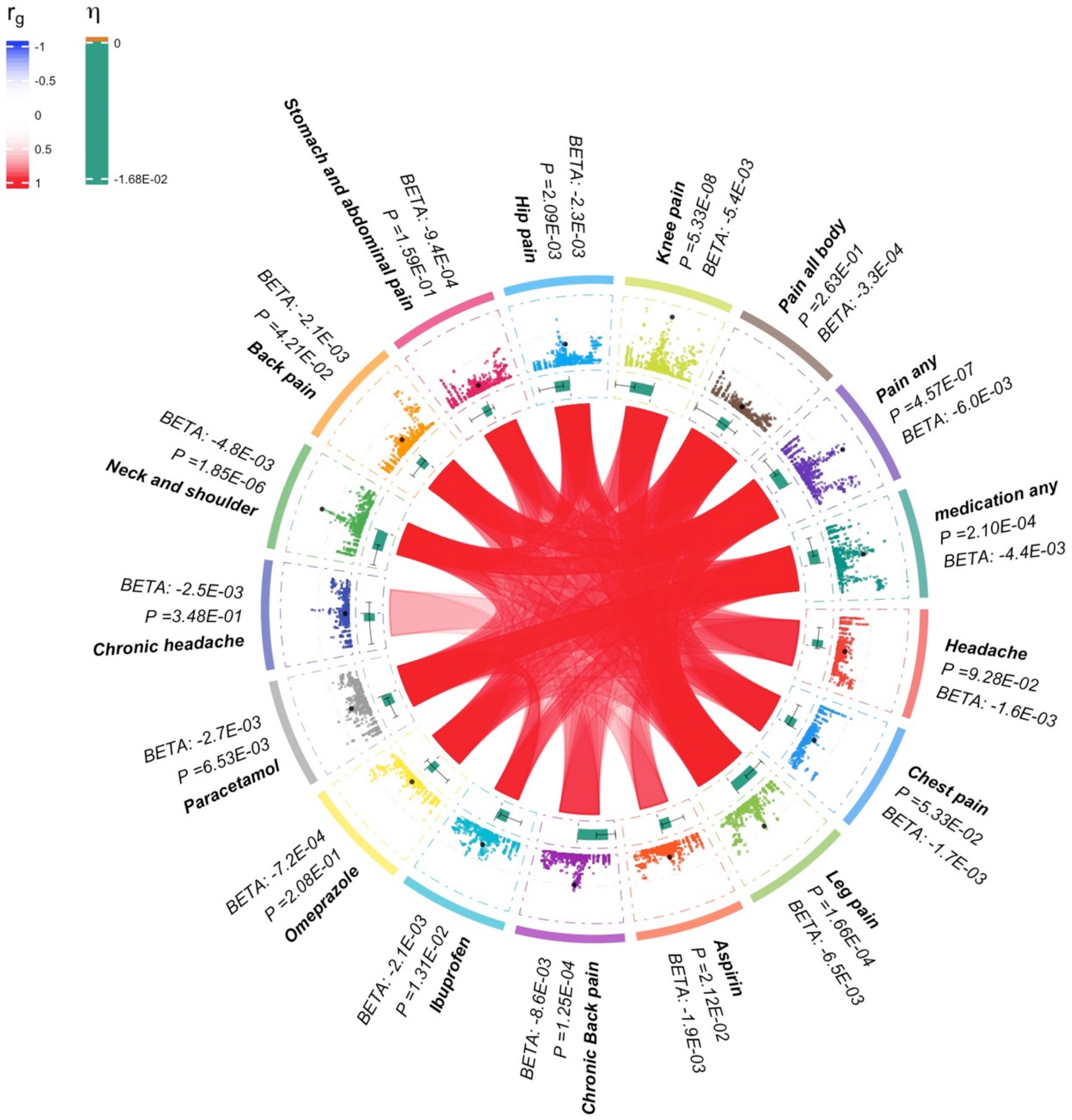

Locus39 6p21.31, rs2395607 Pleio-P= 1.01E-15, intronic to *UHRF1BP1* gene

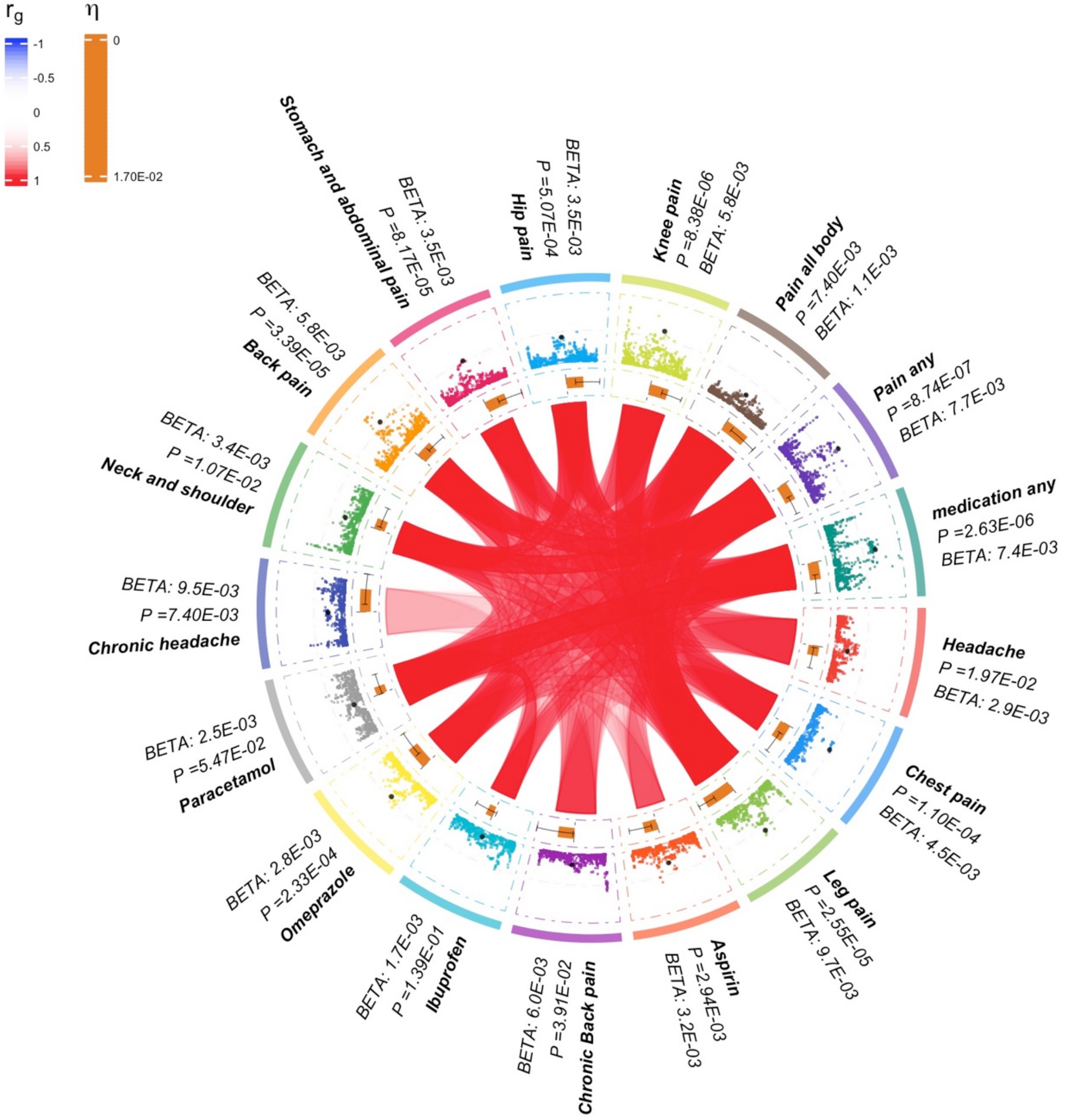

Locus40 6p21.1, rs10948201 Pleio-P= 2.01E-08, intronic to *SUPT3H* gene

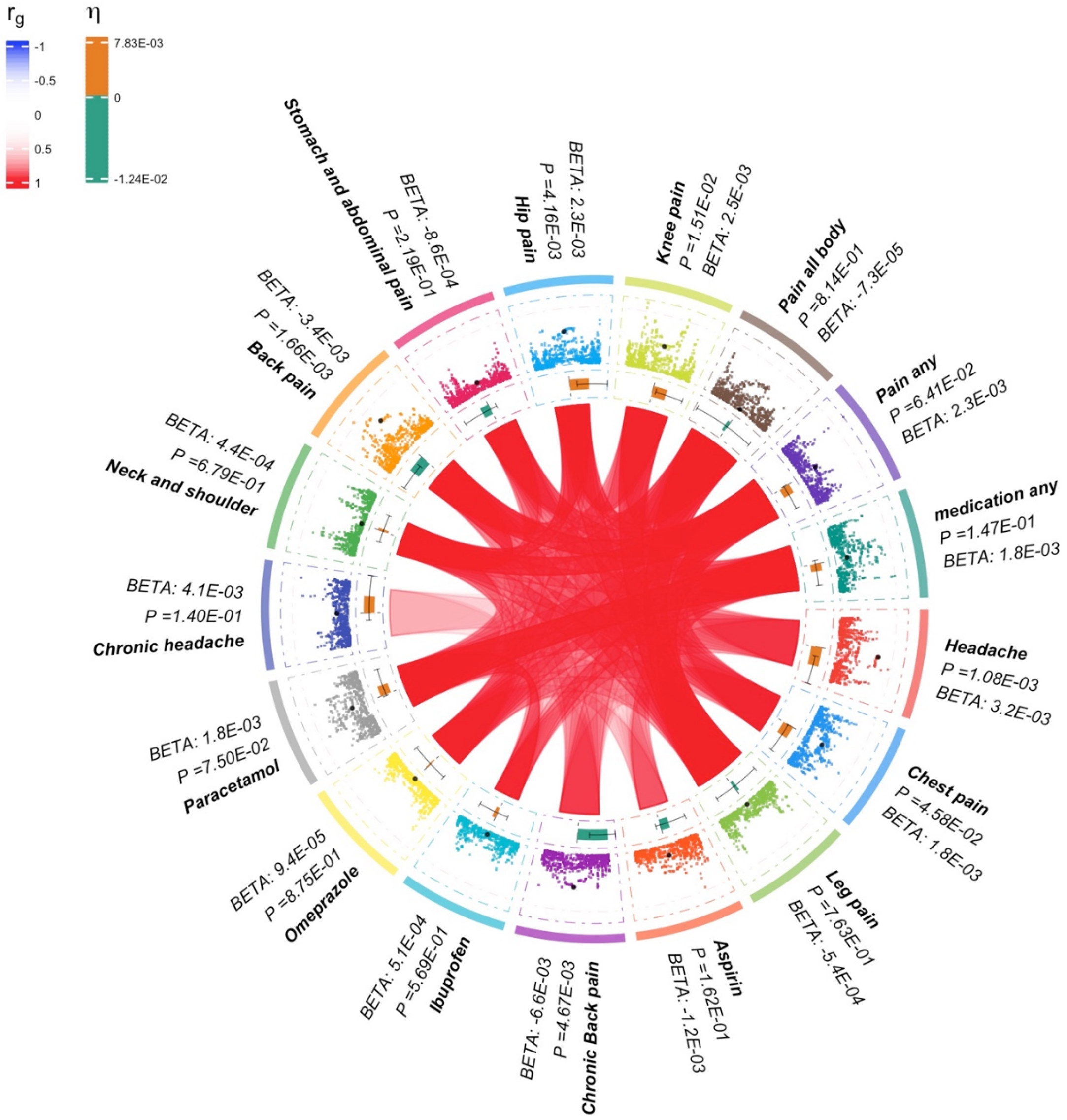

Locus41 6q16.1, rs11153082 Pleio-P= 3.25E-46, intronic to *FHL5* gene

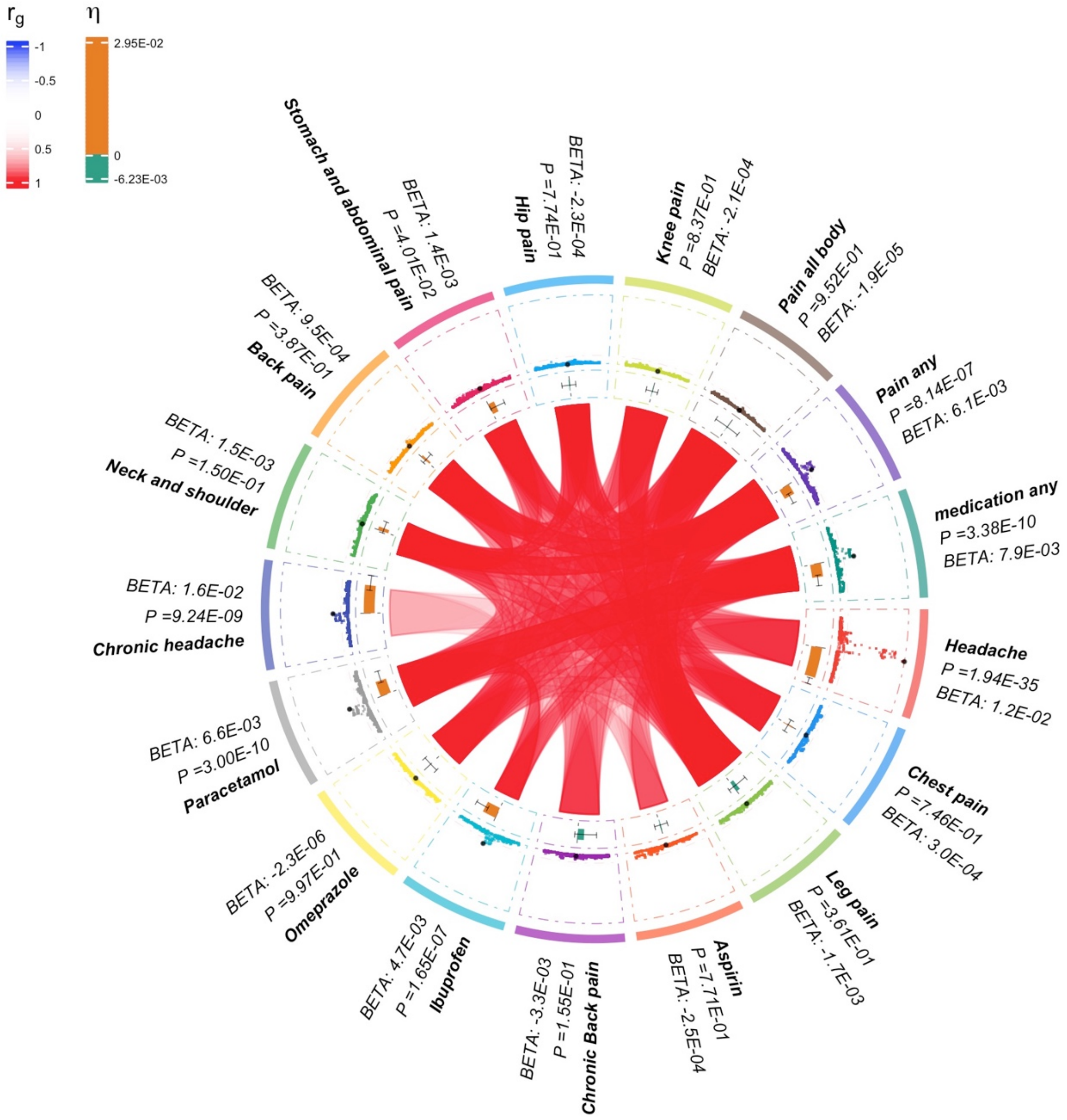

Locus42 6q22.31, rs9320821 Pleio-P= 1.21E-10, intergenic to RNU4-76P gene

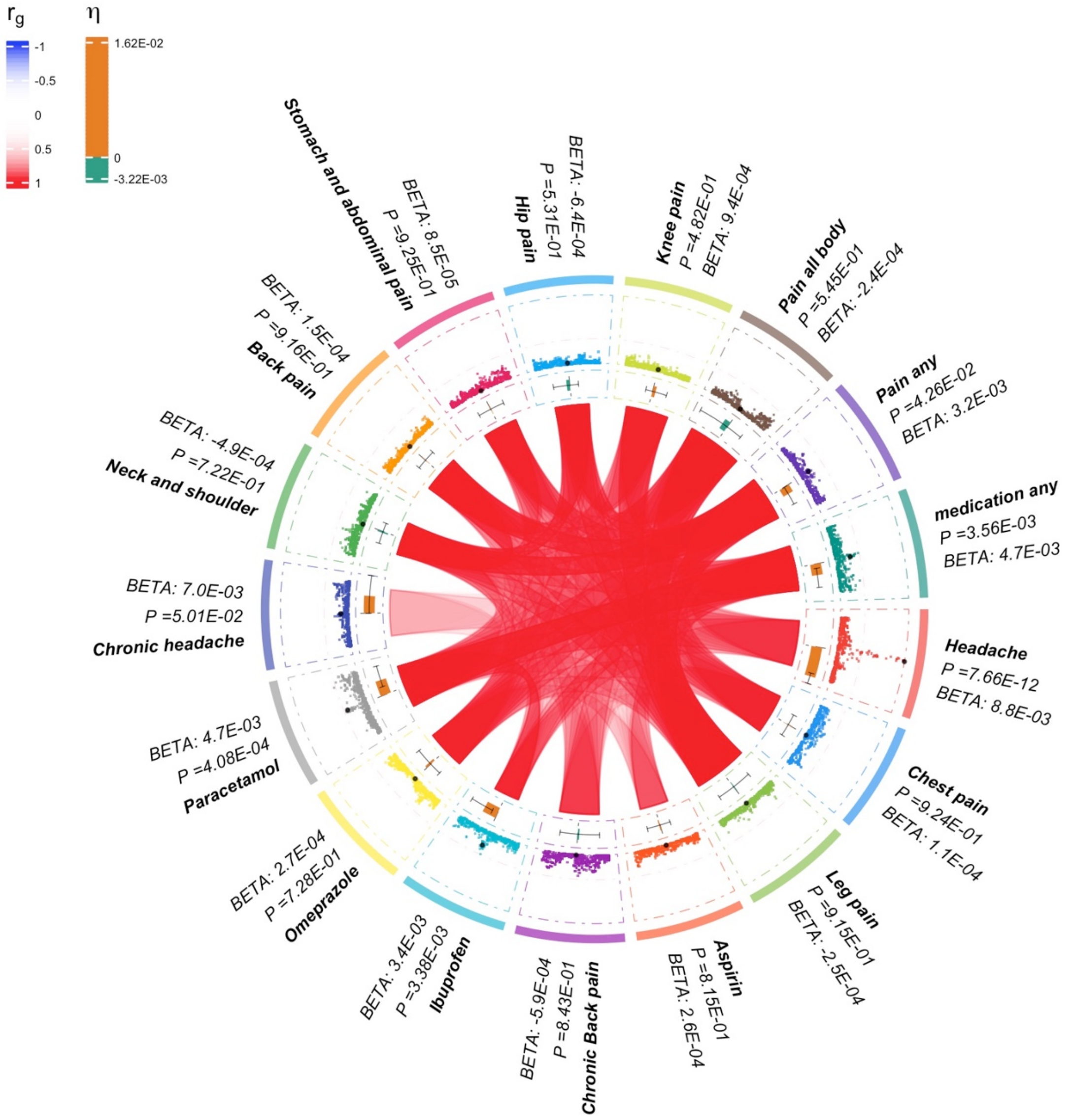

Locus43 6q22.33, rs4895846 Pleio-P= 9.18E-09, intronic to *LAMA2* gene

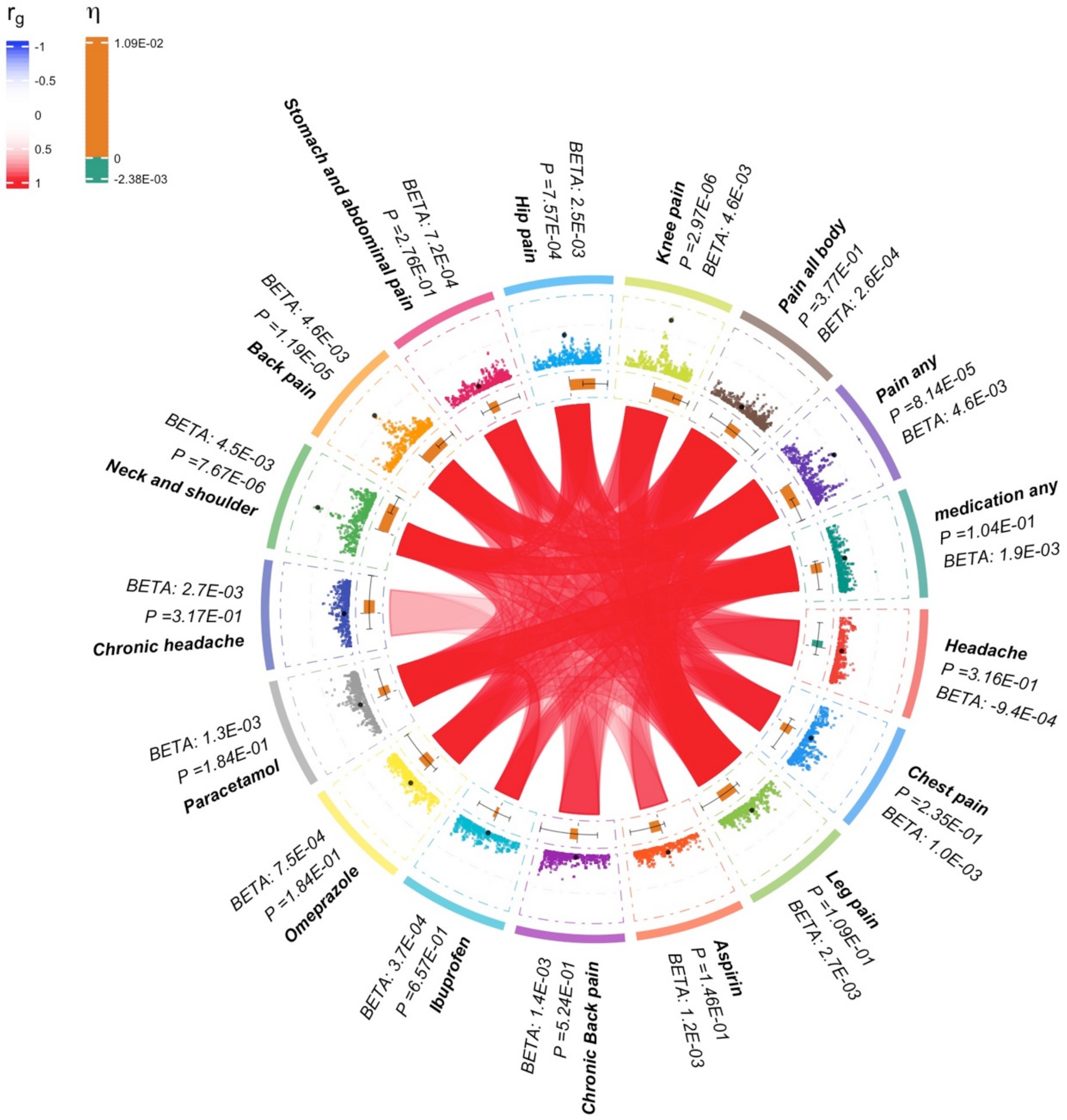

Locus44 6q25.1, rs1934534 Pleio-P= 2.38E-08, intronic to *LATS1* gene

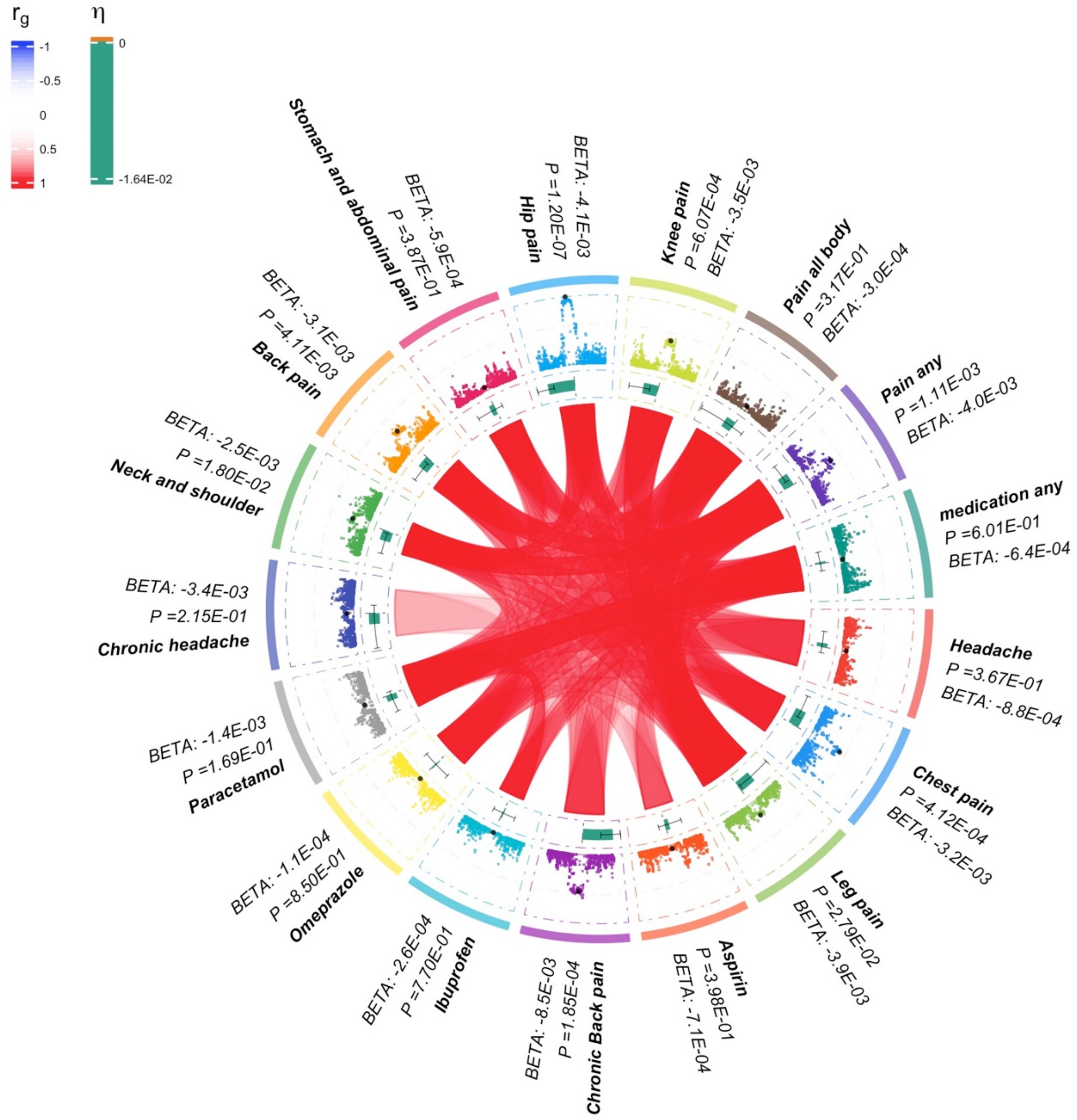

Locus45 6q25.3, rs3918286 Pleio-P= 2.04E-08, intronic to *SLC22A3* gene

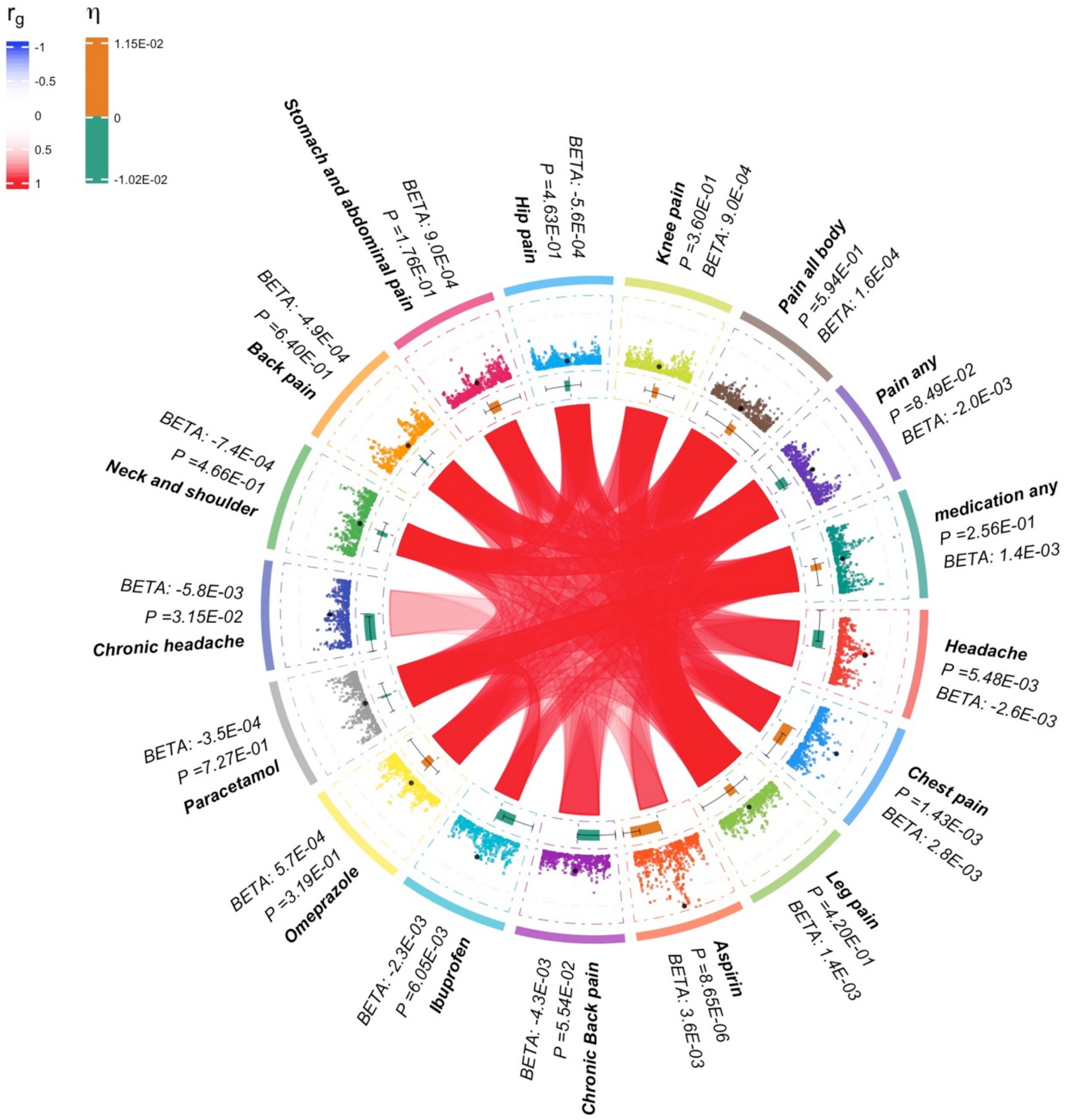

Locus46 7p22.3, rs11764590 Pleio-P= 8.69E-10, intronic to MAD1L1 gene

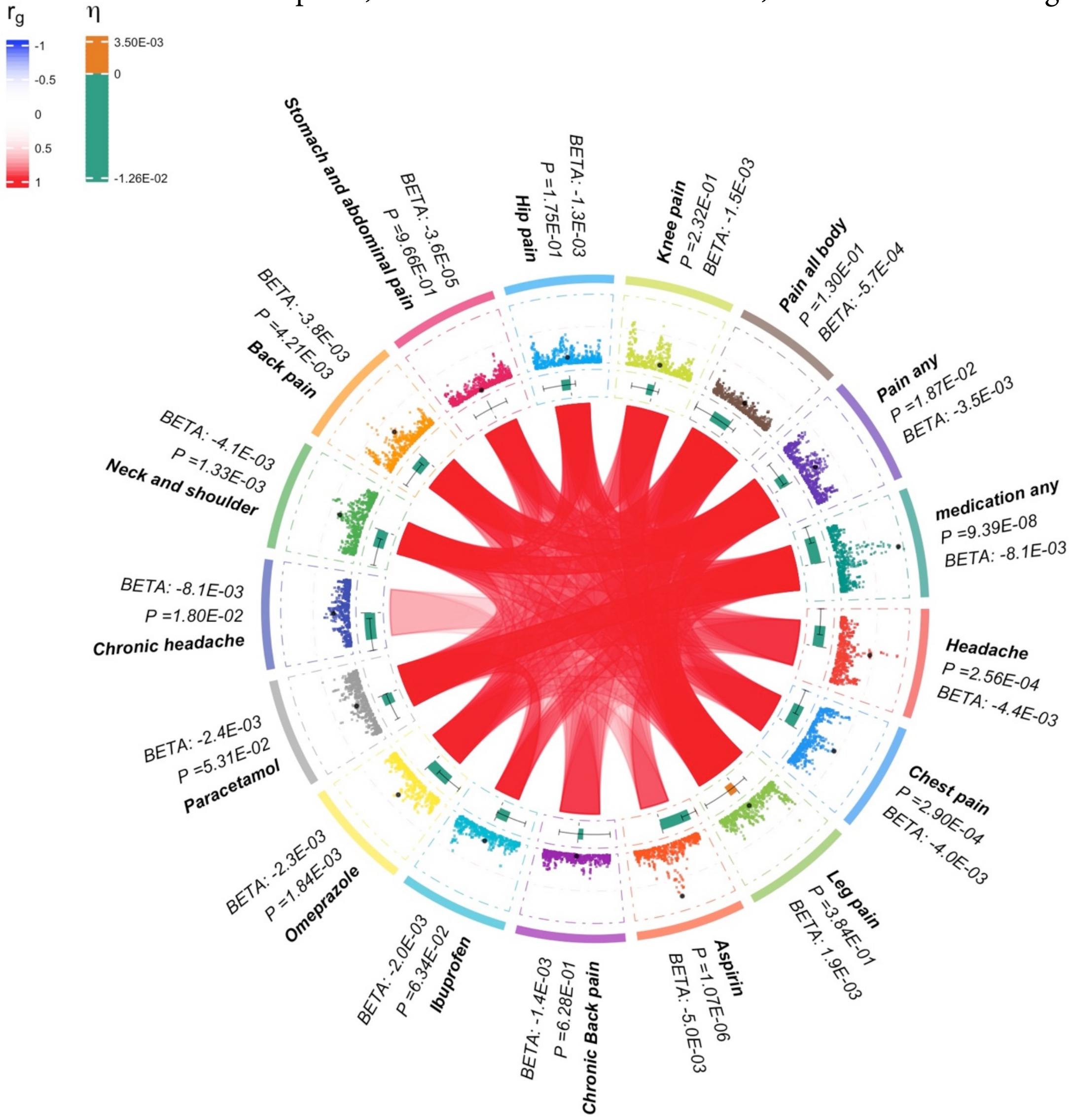

Locus47 7p15.3, rs2282888 Pleio-P= 2.83E-09, intronic to *SP4* gene

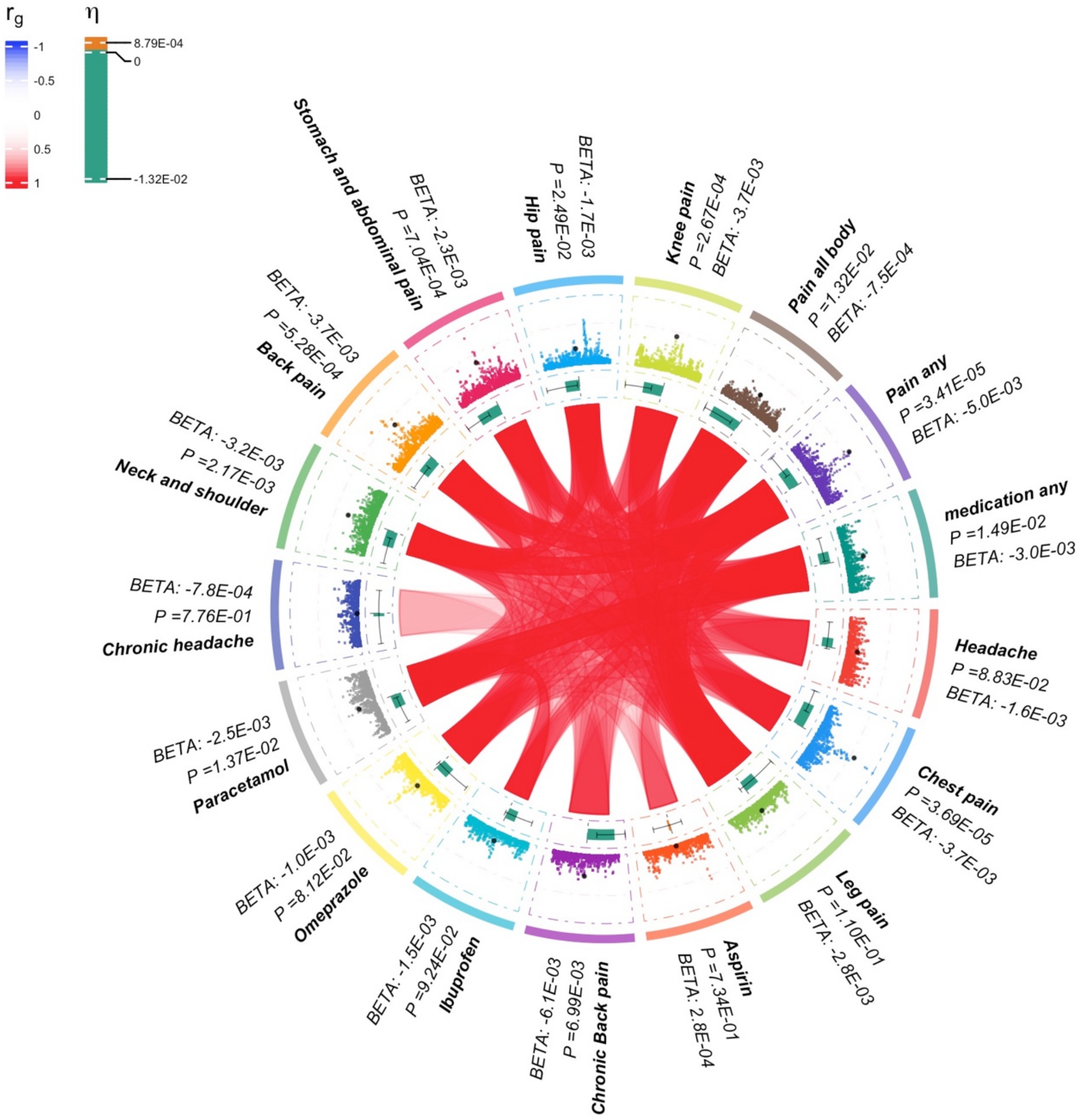

Locus48 7p14.1, rs12532479 Pleio-P= 4.73E-12, intronic to *SUGCT* gene

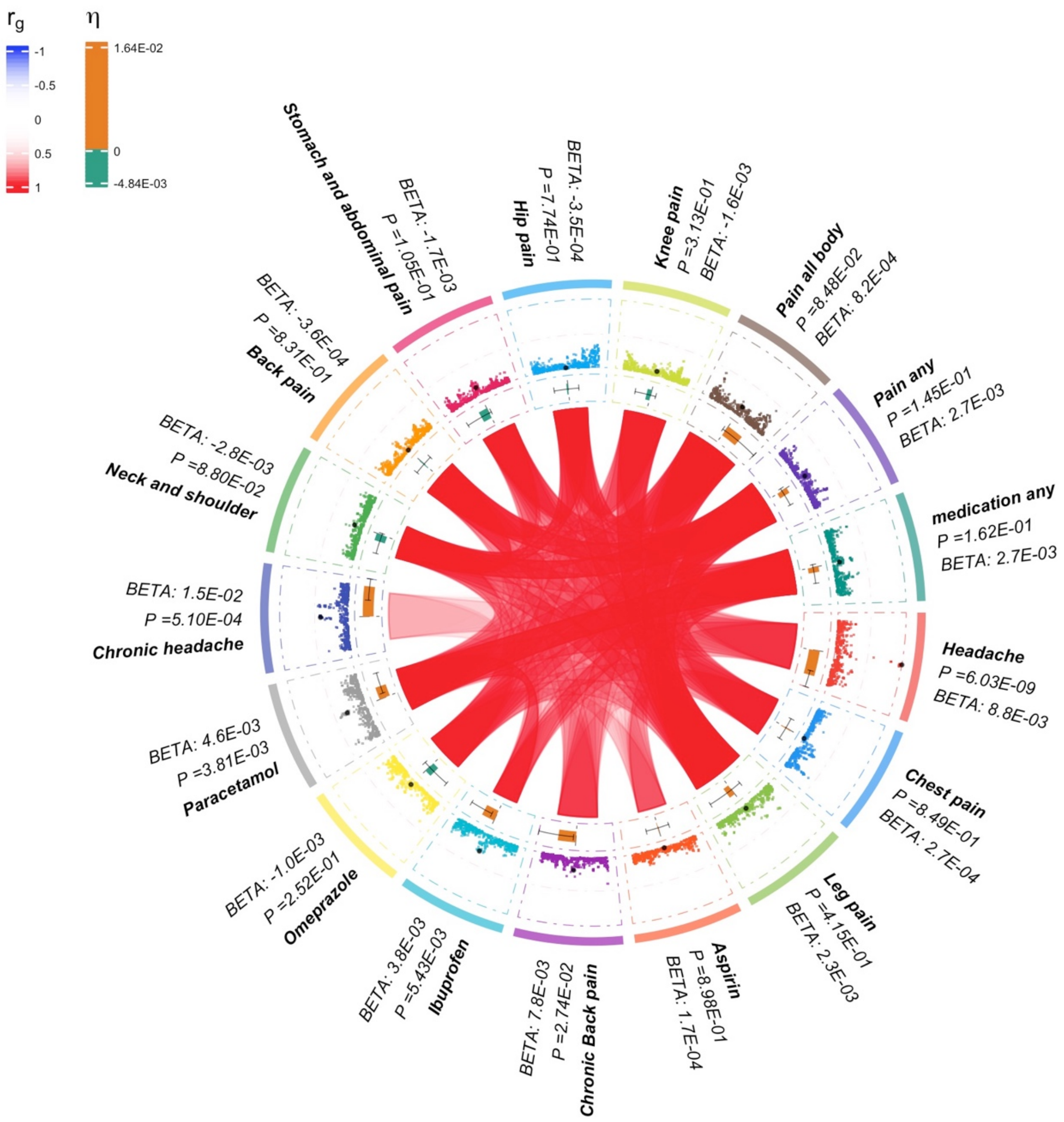

Locus49 7q31.1, rs1852470 Pleio-P= 2.39E-10, intronic to *FOXP2* gene

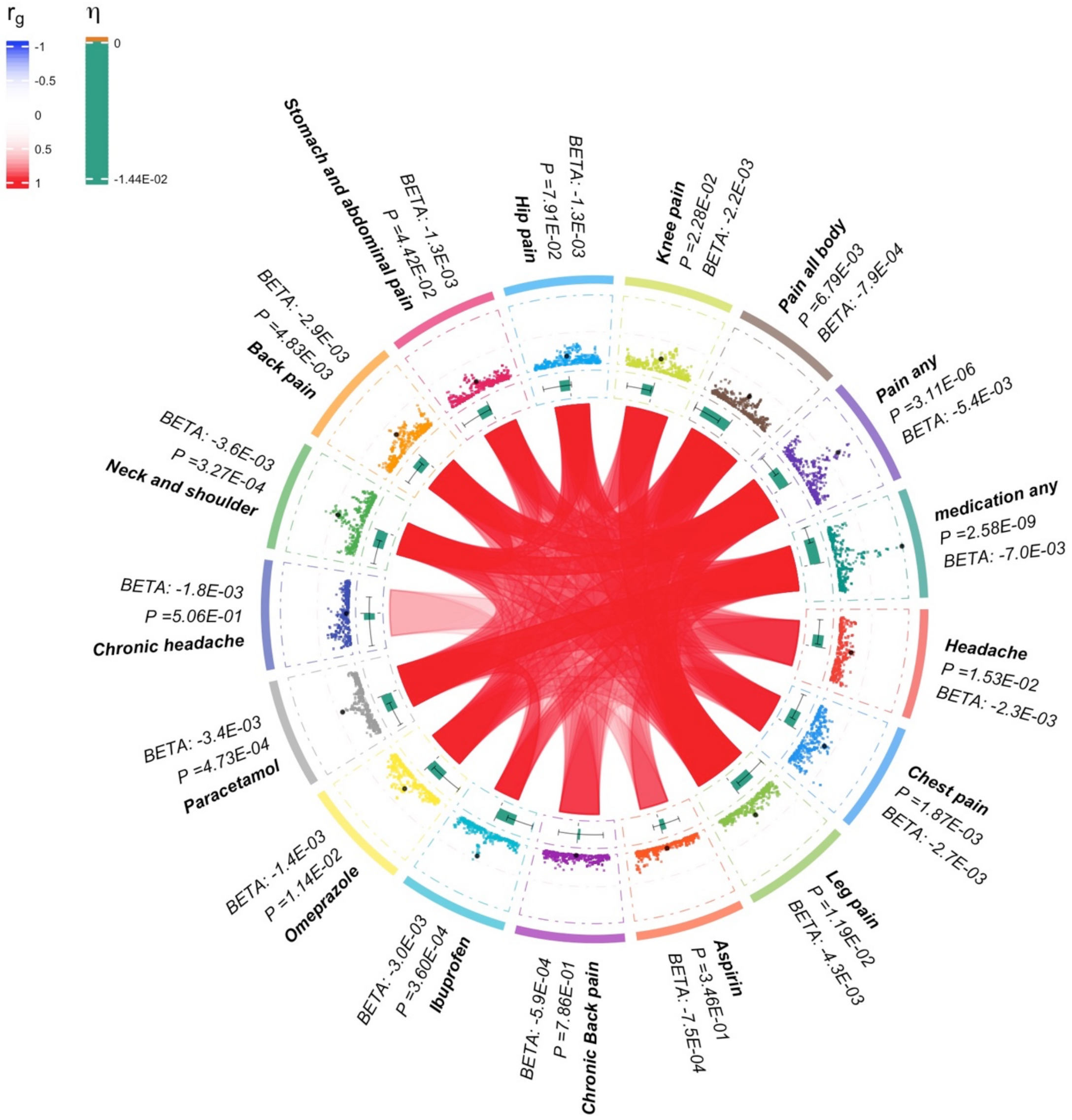

Locus50 7q31.31, rs2721348 Pleio-P= 4.19E-08, intergenic to *TSPAN12* gene

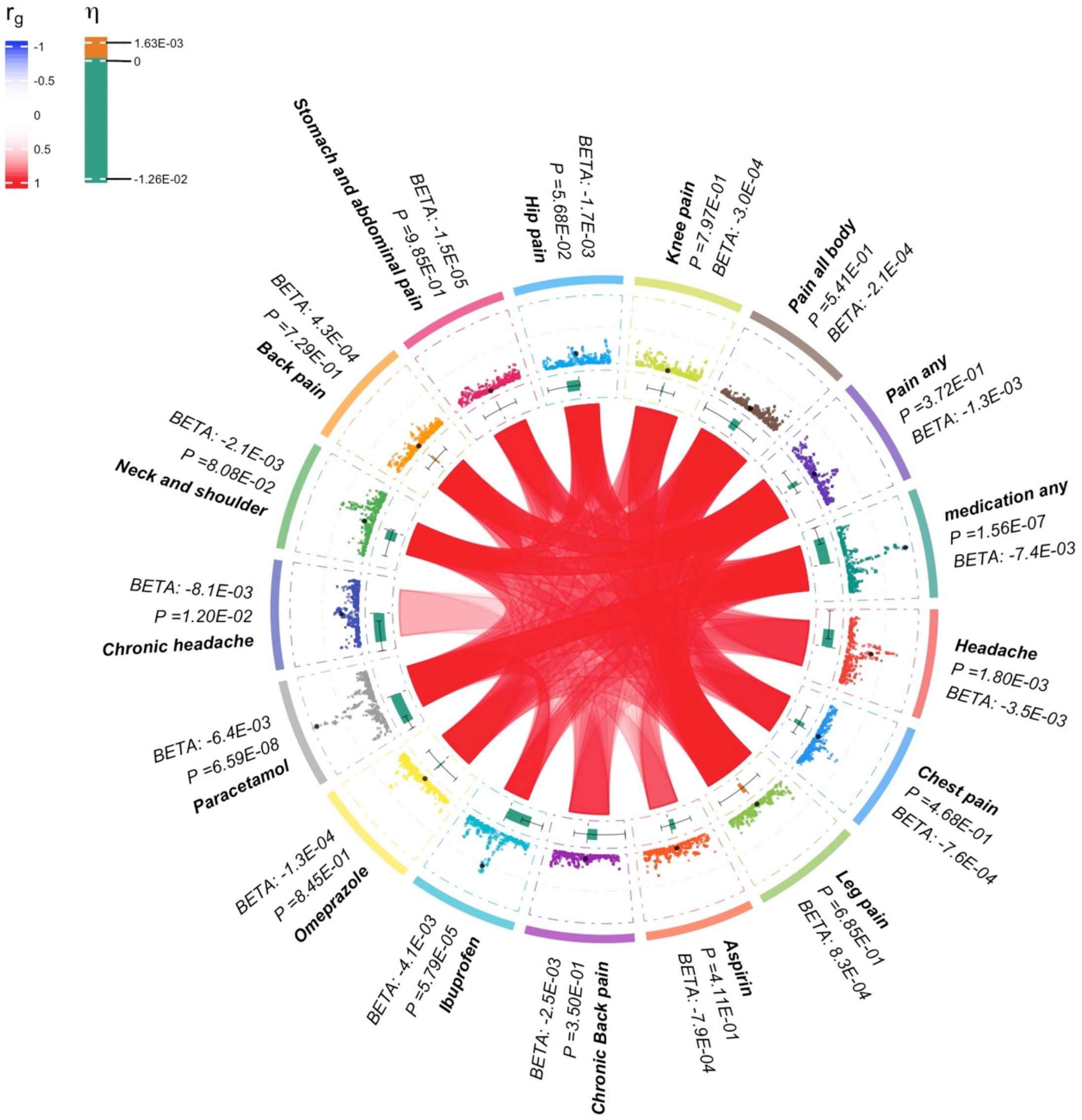

Locus51 8p23.1, rs330944 Pleio-P= 1.64E-09, intronic to RP11-10A14.4 gene

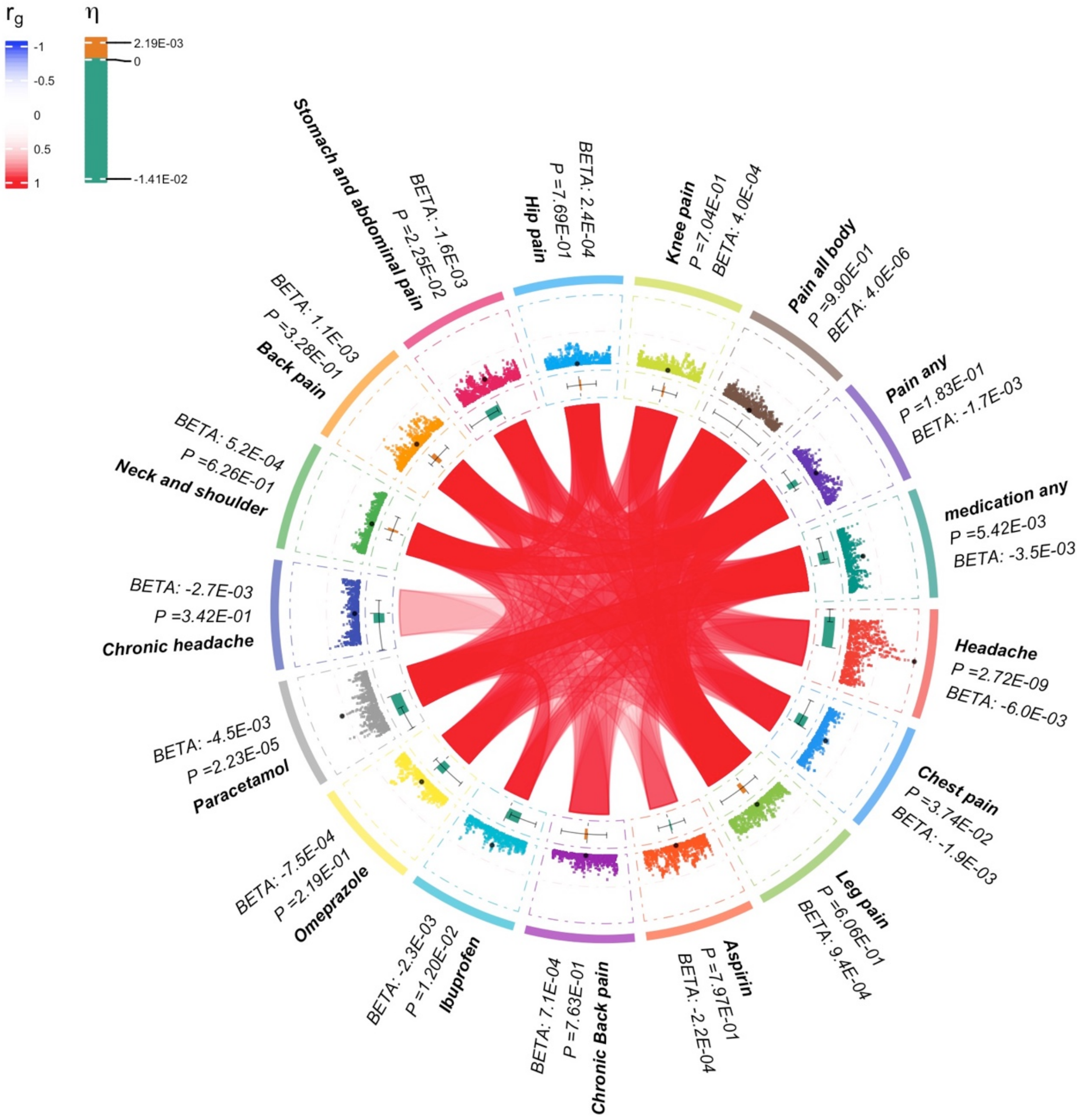

Locus52 8p21.3, rs1552284 Pleio-P= 3.33E-08, downstream to *FAM160B2* gene

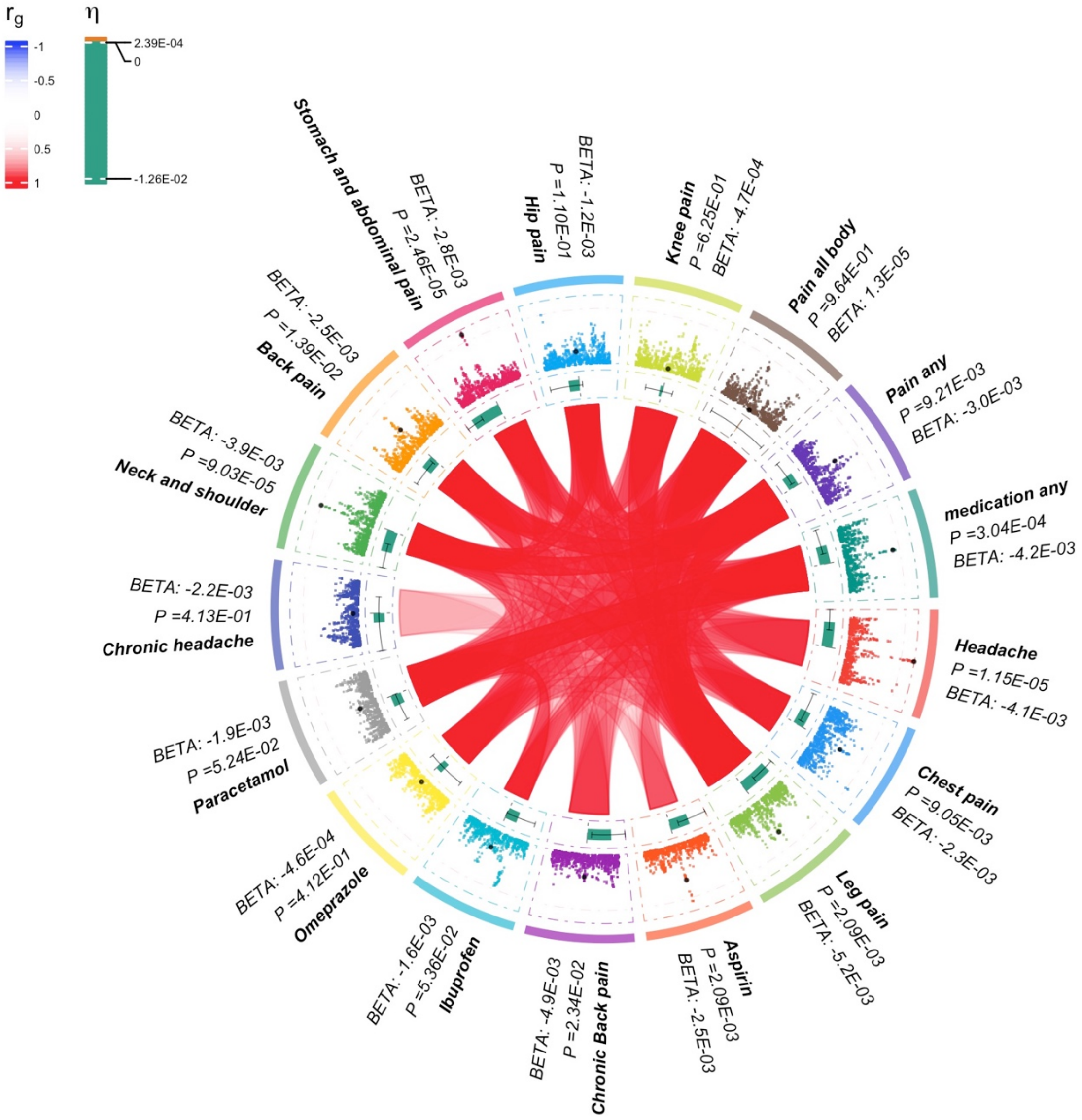

Locus53 8q13.3, rs3863241 Pleio-P= 1.49E-08, intergenic to RP11-531A24.3 gene

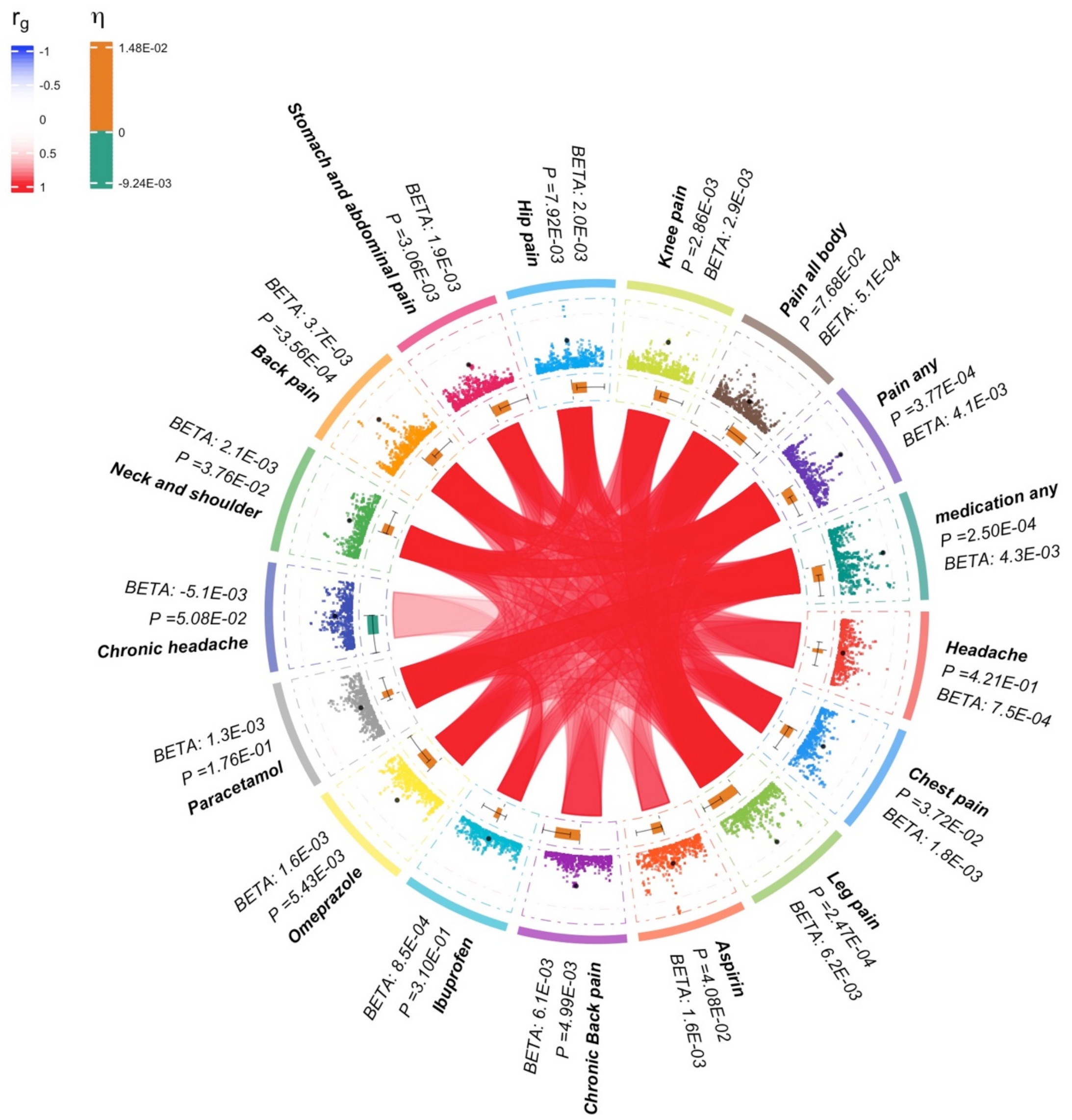

Locus54 8q24.3, rs938724 Pleio-P= 1.54E-08, intergenic to *PTK2* gene

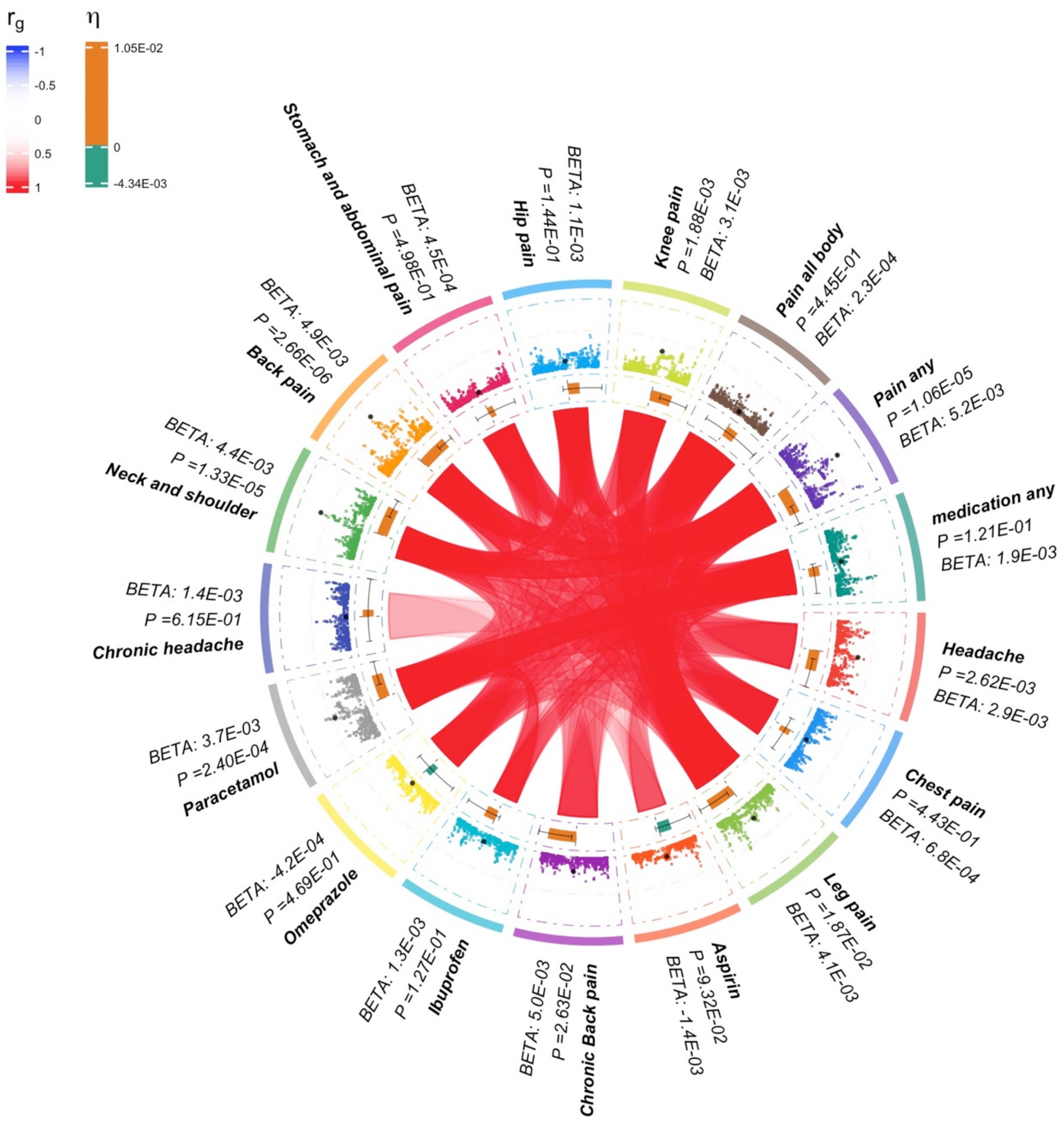

Locus55 9p21.3, rs1537375 Pleio-P= 1.78E-22, ncRNA_intronic to *CDKN2B*-AS1 gene

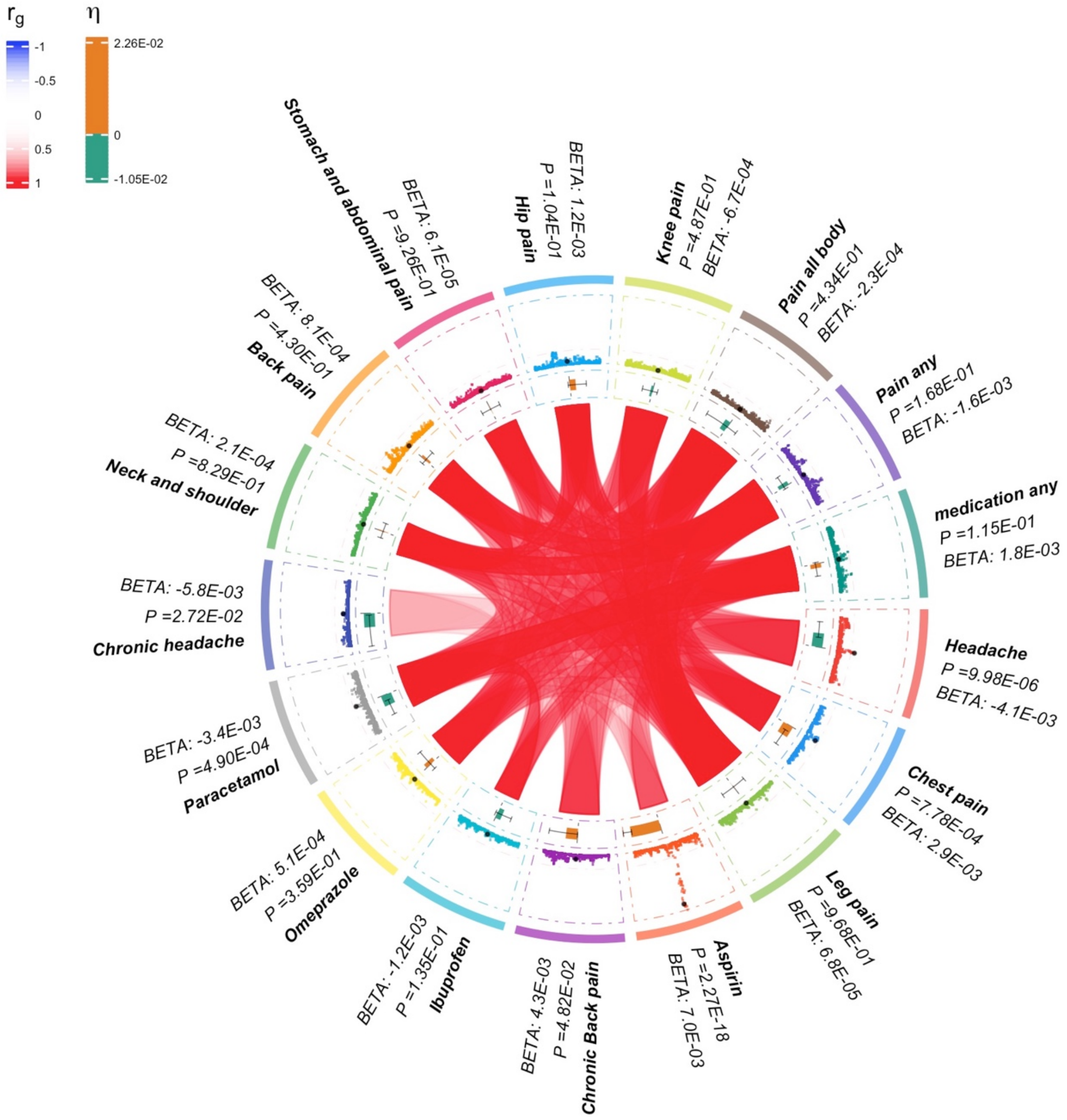

Locus56 9q21.11, rs11145043 Pleio-P= 2.89E-09, UTR3 to *FXN* gene

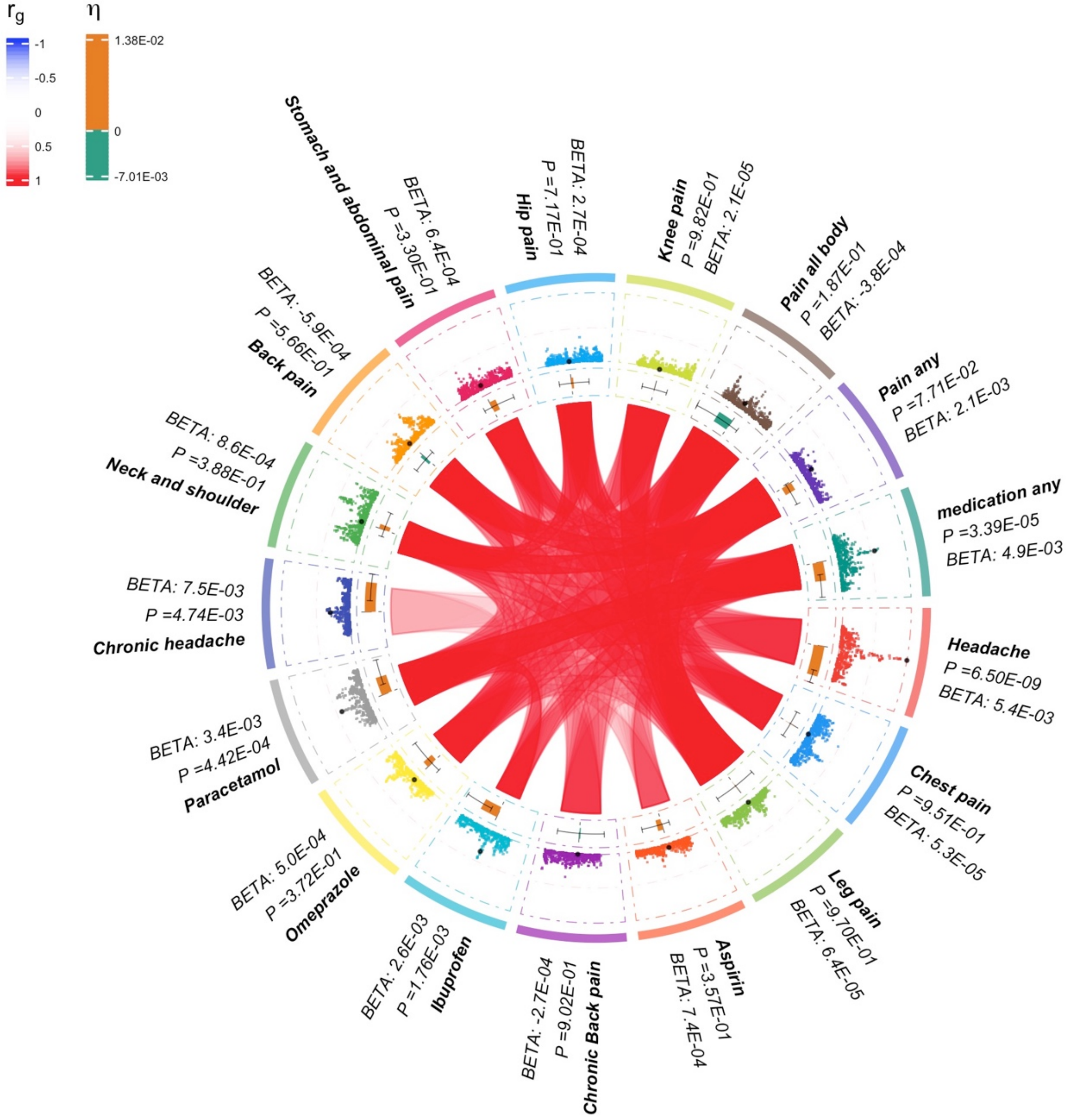

Locus57 9q22.31, rs12379660 Pleio-P= 1.78E-09, intergenic to Y_RNA gene

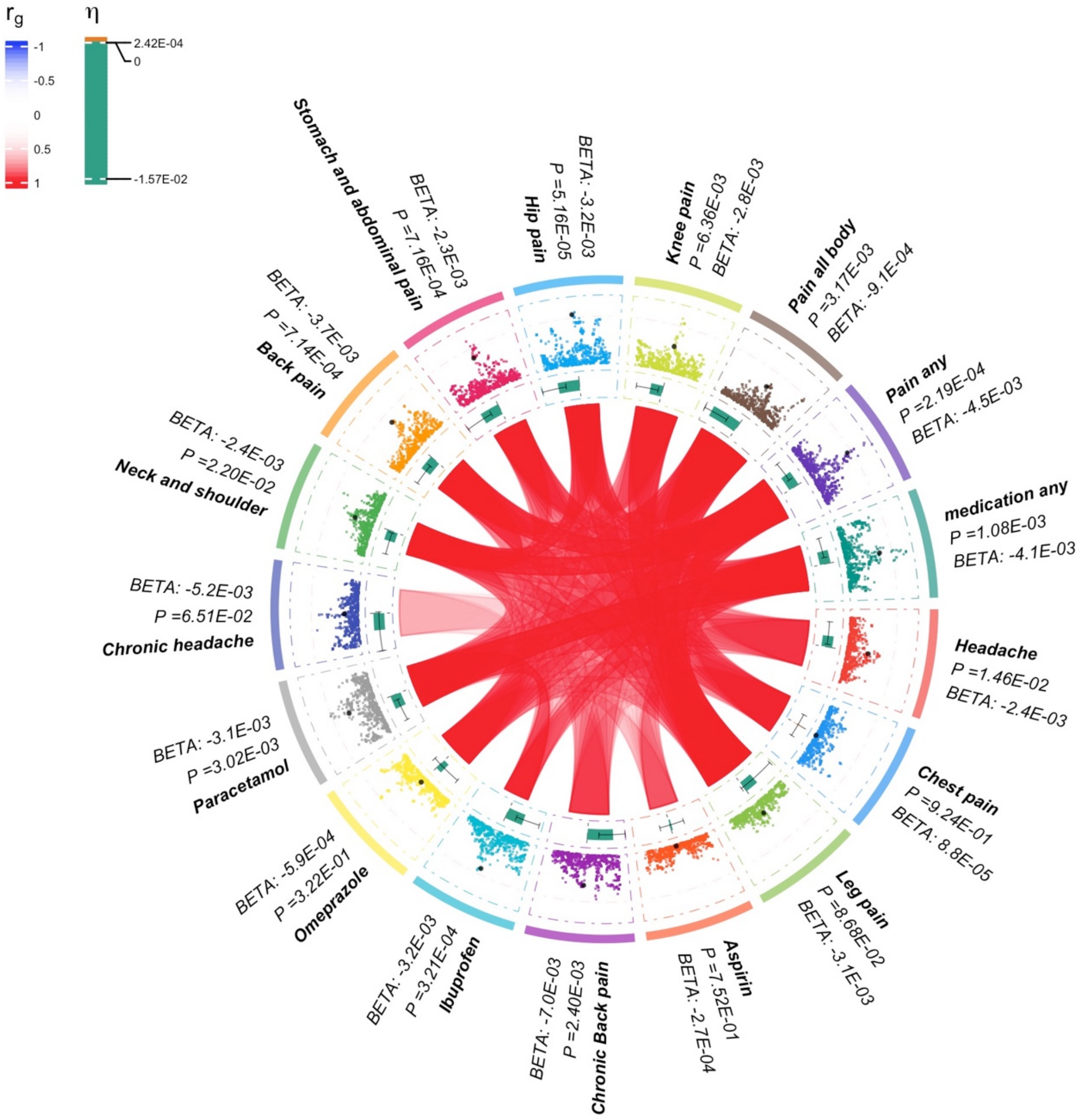

Locus58 9q33.1, rs6478241 Pleio-P= 4.33E-16, intronic to *ASTN2* gene

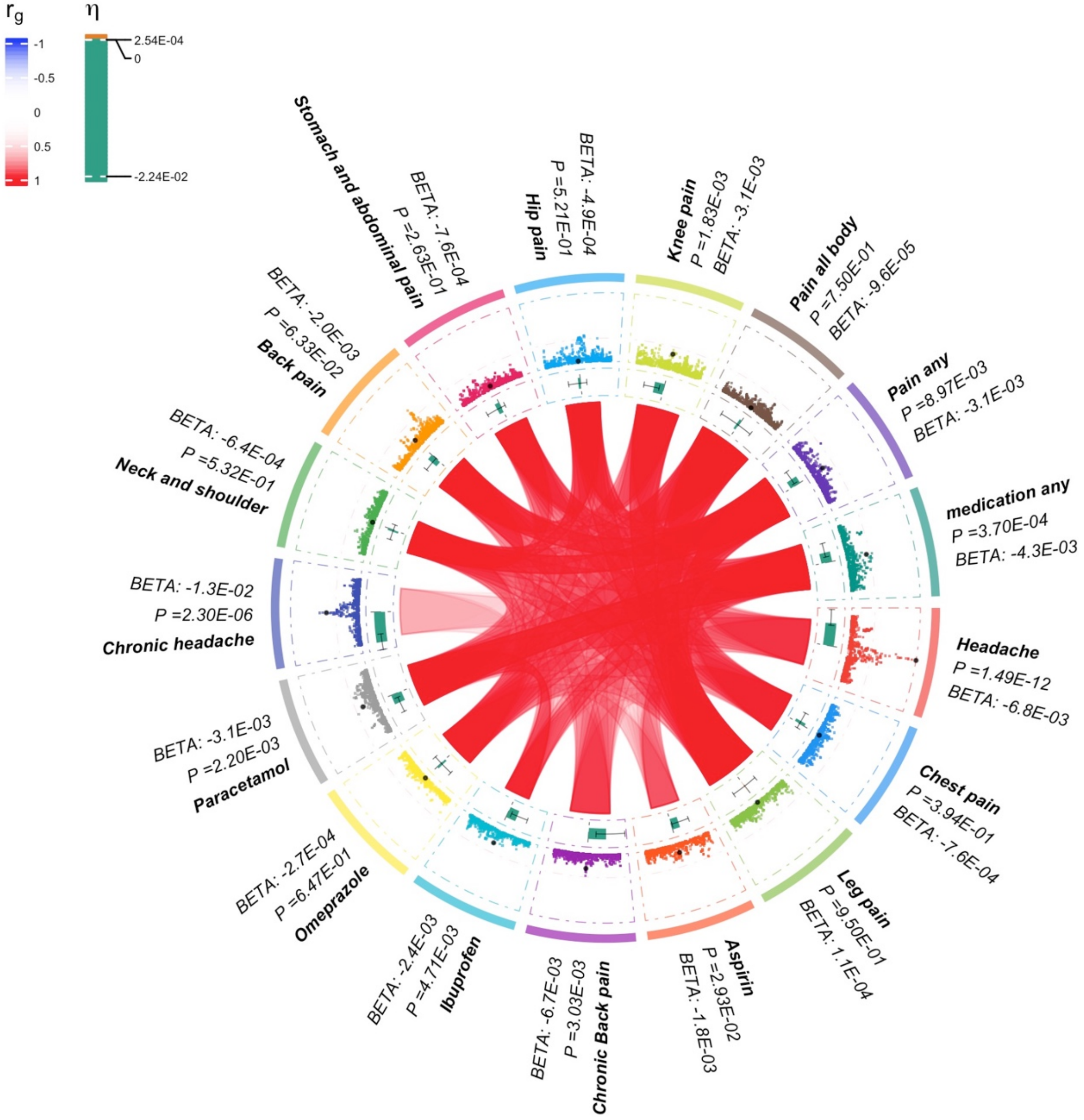

Locus59 10q22.1, rs1245576 Pleio-P= 4.71E-13, intergenic to RP11-150D20.5 gene

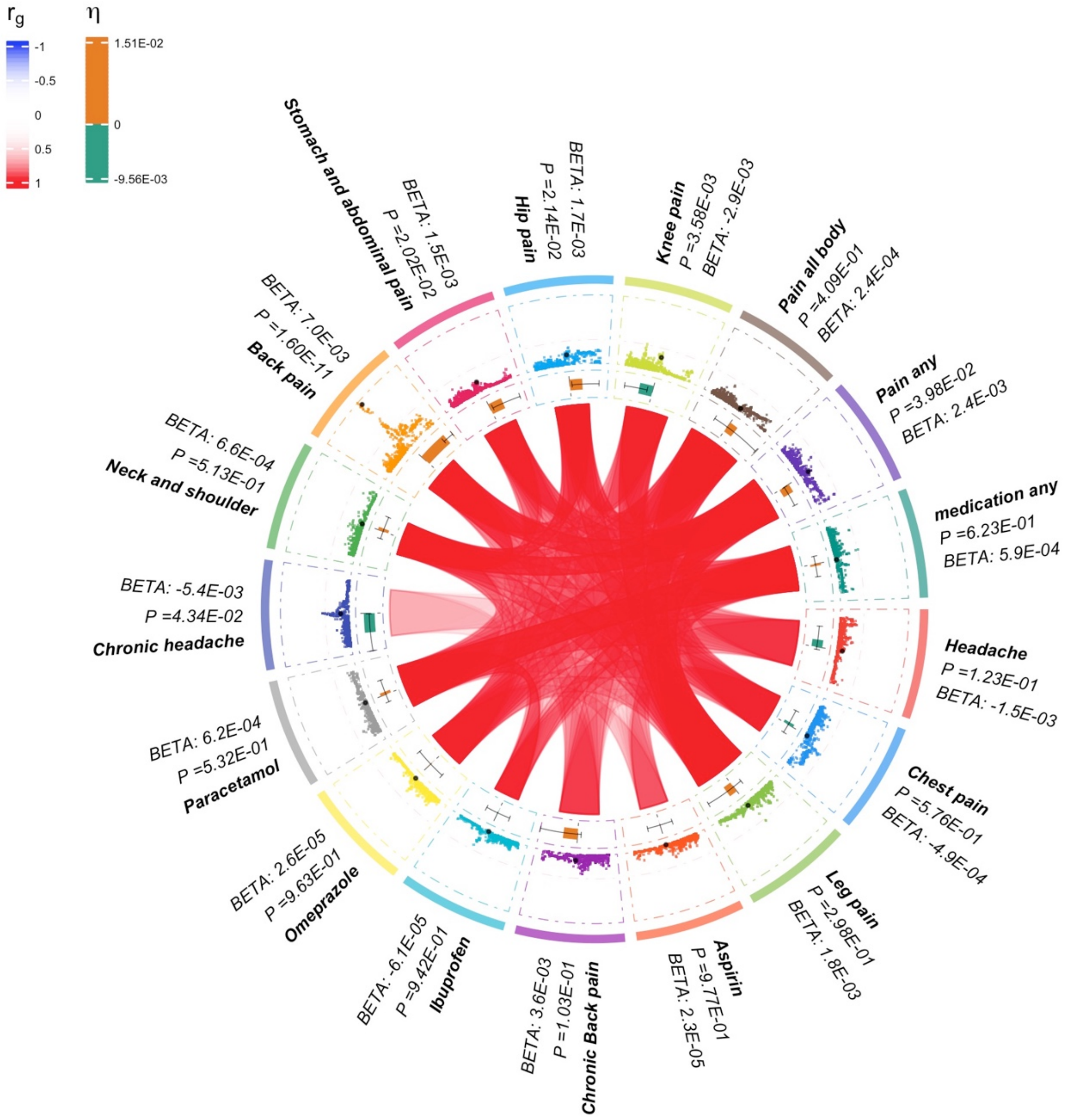

Locus60 10q24.2, rs7924025 Pleio-P= 2.94E-09, intronic to *HPSE2* gene

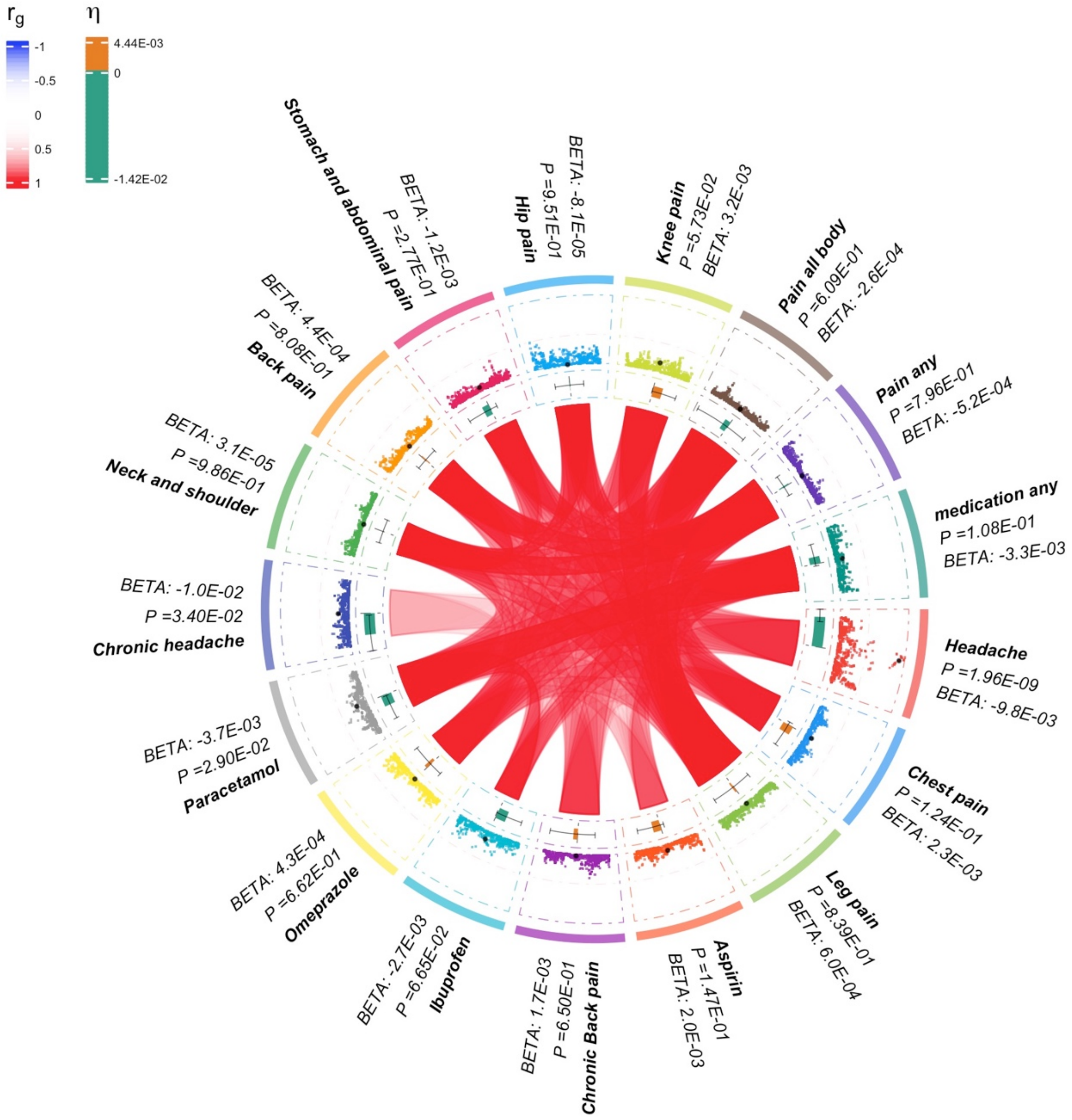

Locus61 10q25.1, rs11599236 Pleio-P= 1.44E-12, intronic to *SORCS3* gene

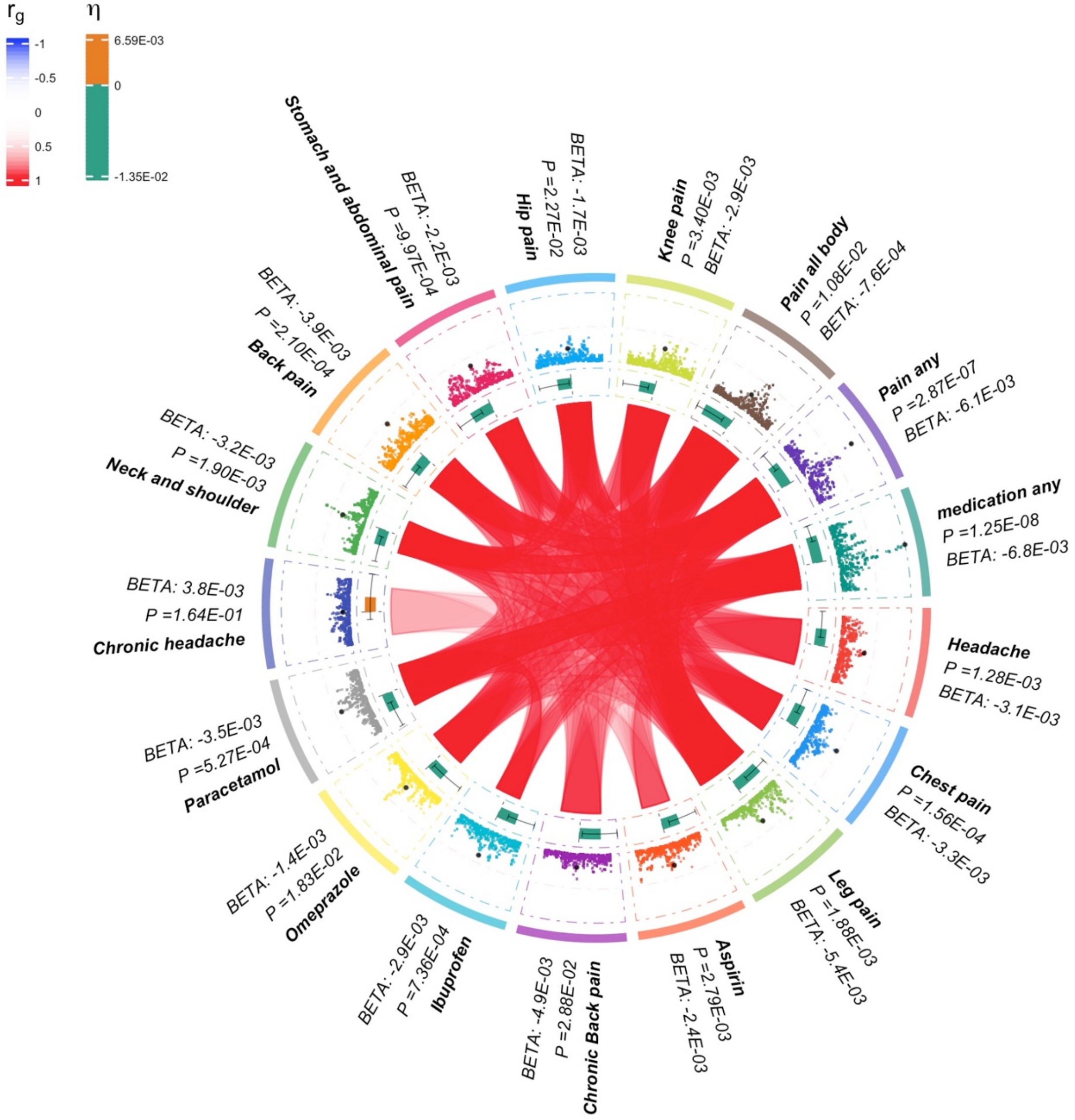

Locus62 10q26.13, rs2421015 Pleio-P= 1.31E-10, intronic to *PLEKHA1* gene

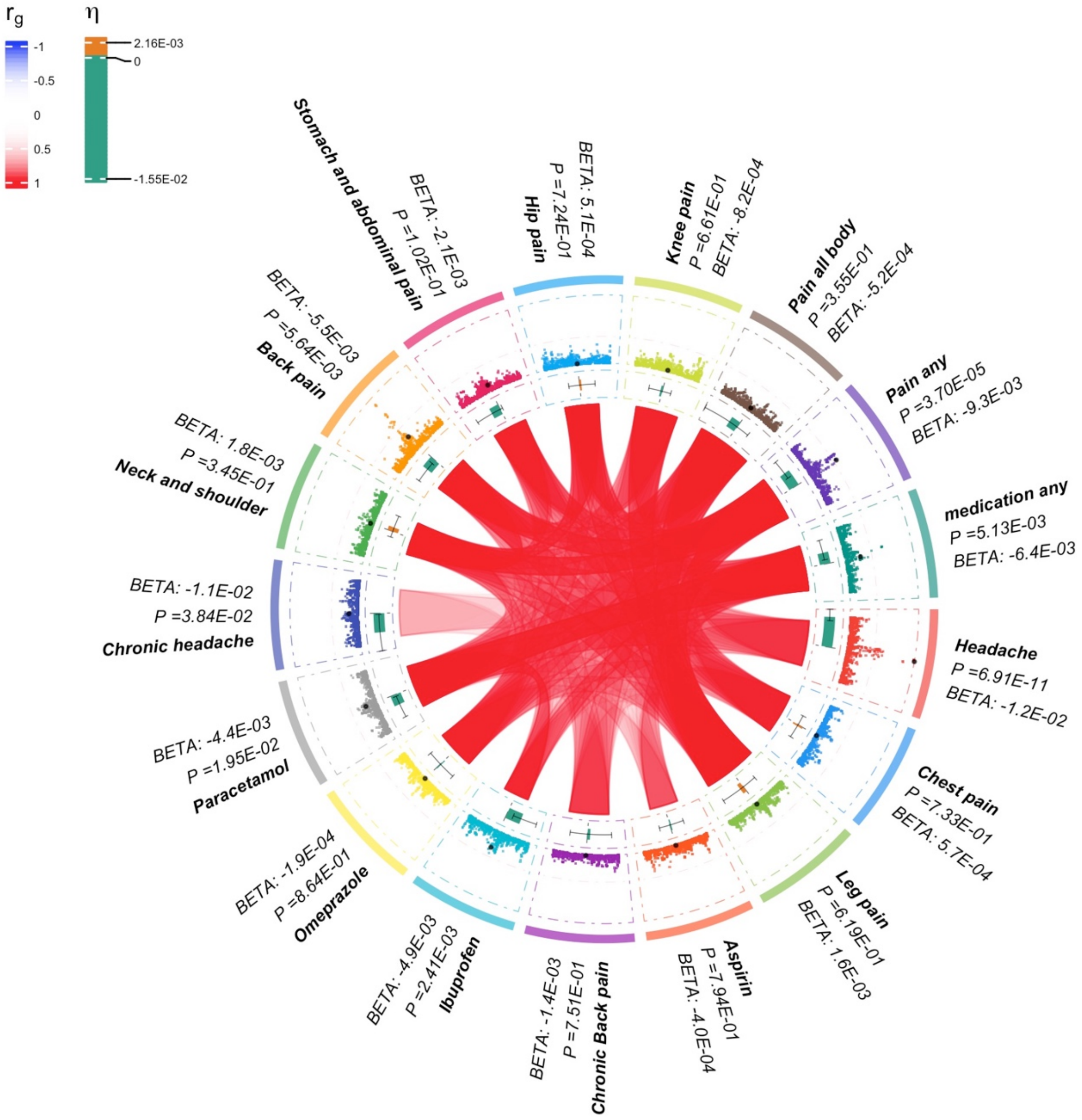

Locus63 11p15.4, rs4909945 Pleio-P= 3.36E-13, exonic to *MRVI1* gene

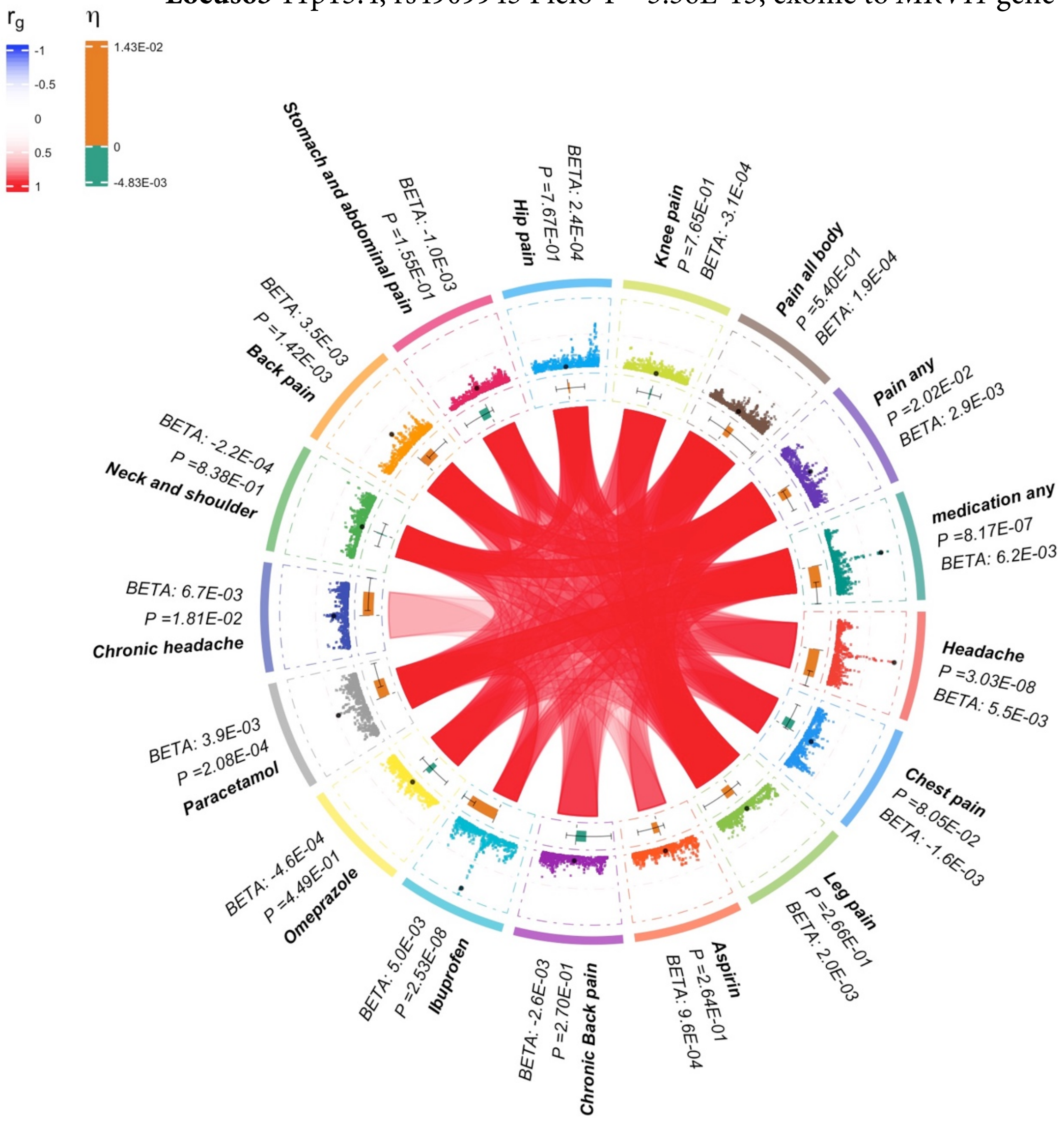

Locus64 11q23.2, rs3802847 Pleio-P= 2.12E-11, intronic to *NCAM1* gene

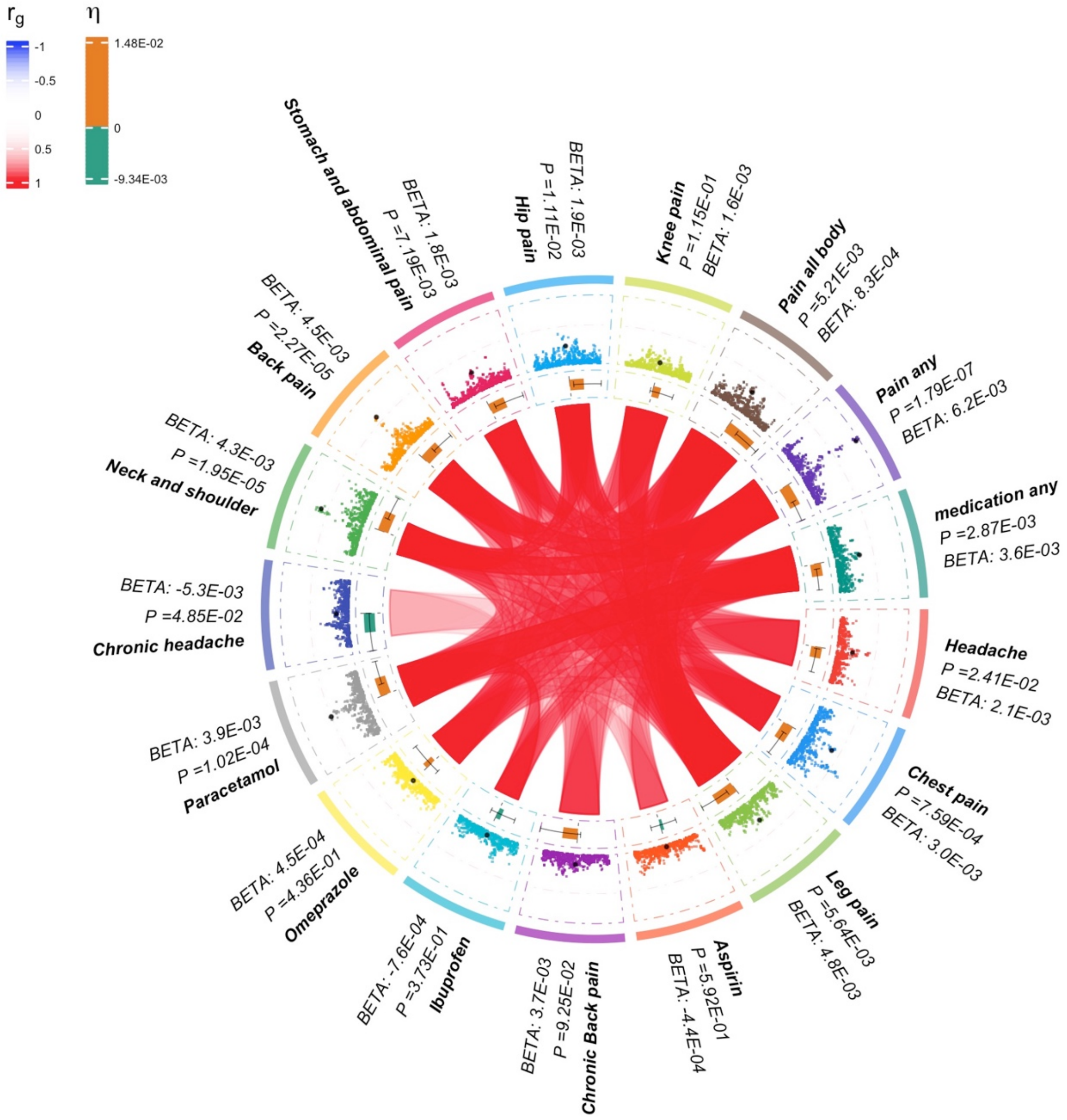

Locus65 12p13.33, rs16931821 Pleio-P= 9.71E-09, intronic to *IQSEC3* gene

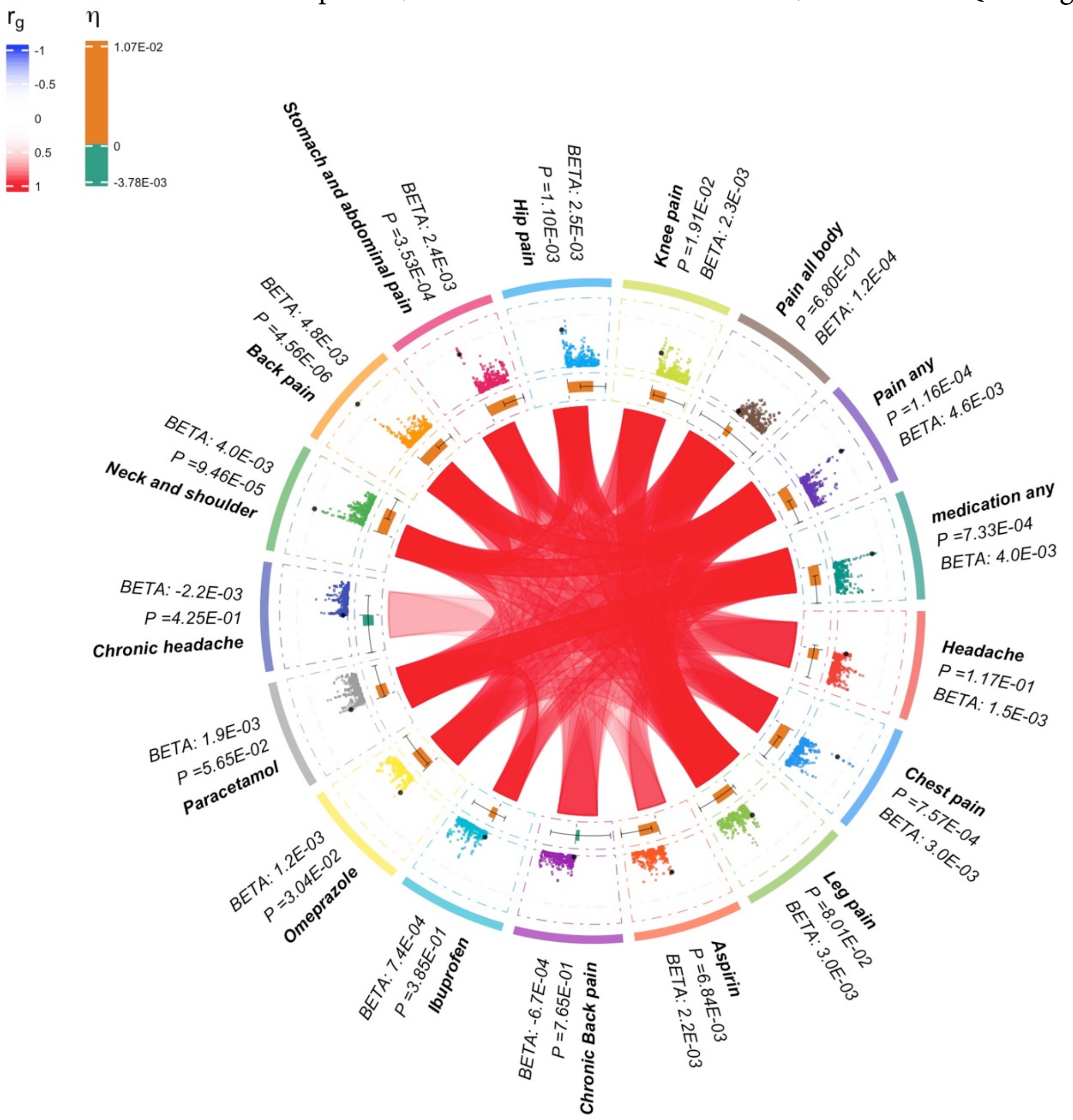

Locus66 12p13.32, rs2160875 Pleio-P= 1.00E-09, intergenic to *FGF6* gene

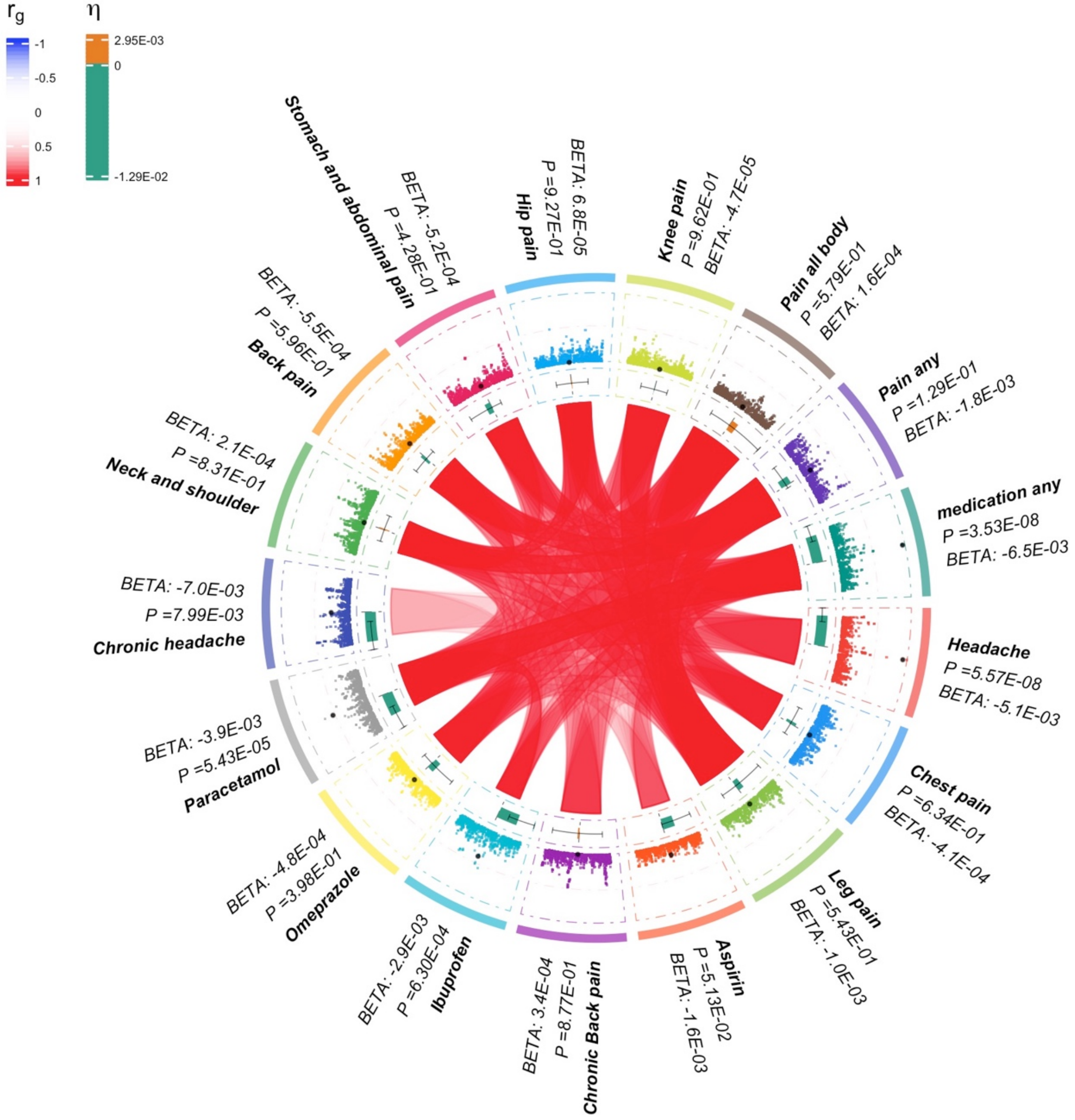

Locus67 12p12.1, rs9804988 Pleio-P= 3.56E-11, intronic to *SOX5* gene

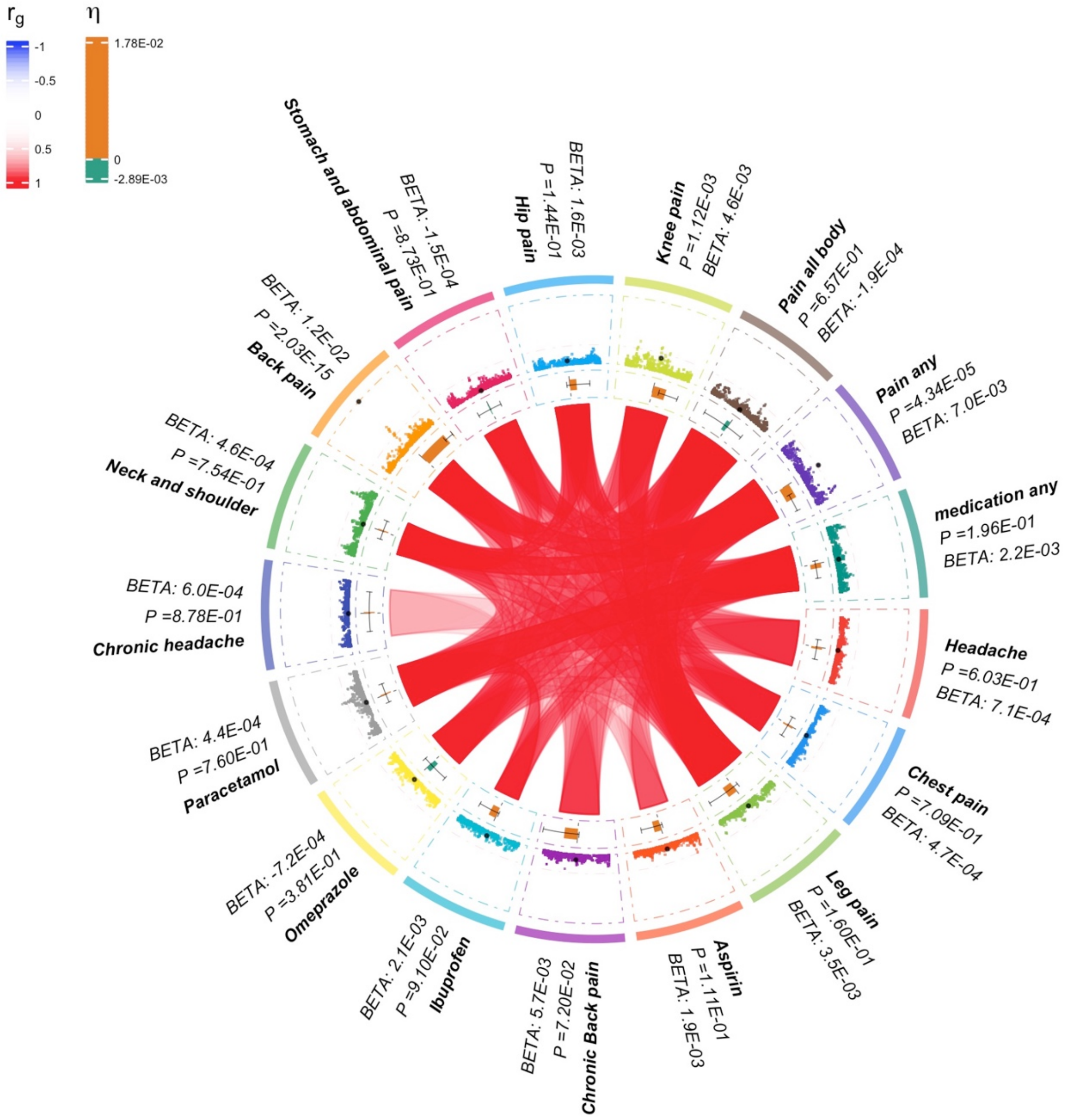

Locus68 12q13.2, rs773108 Pleio-P= 1.55E-08, intronic to *RAB5B* gene

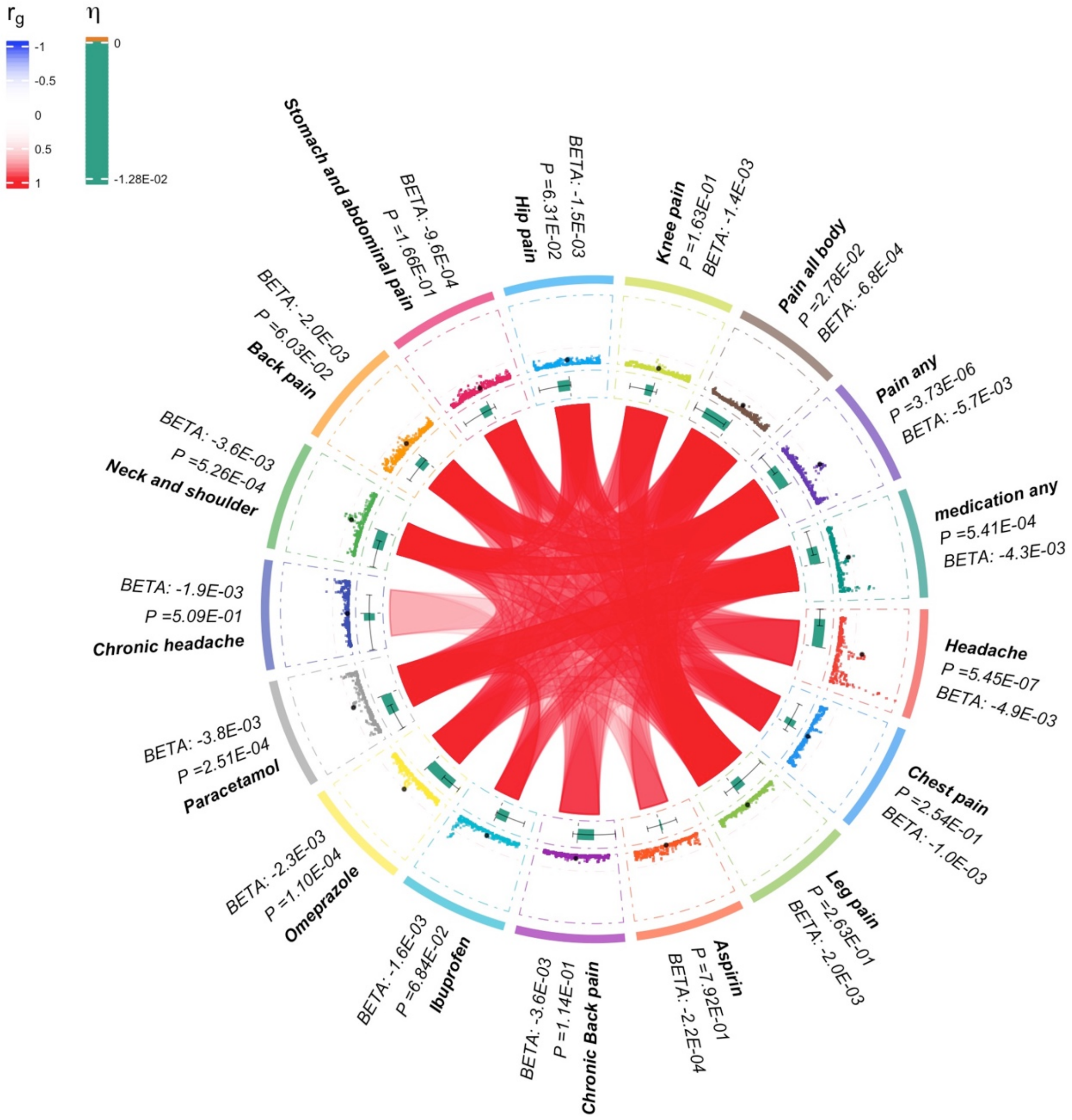

Locus69 12q13.3, rs11172113 Pleio-P= 6.86E-72, intronic to *LRP1* gene

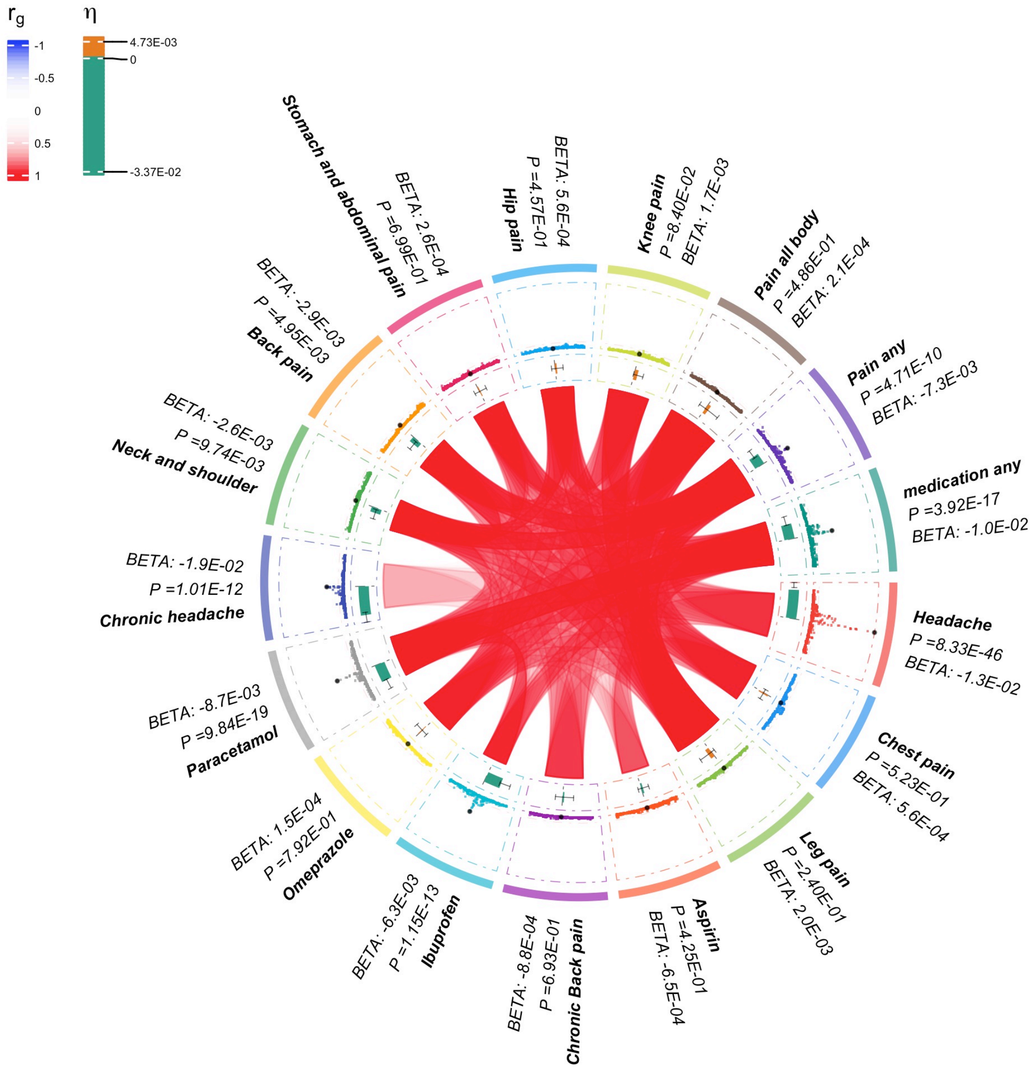

Locus70 12q15, rs1350166 Pleio-P= 7.36E-10, intronic to *CPSF6* gene

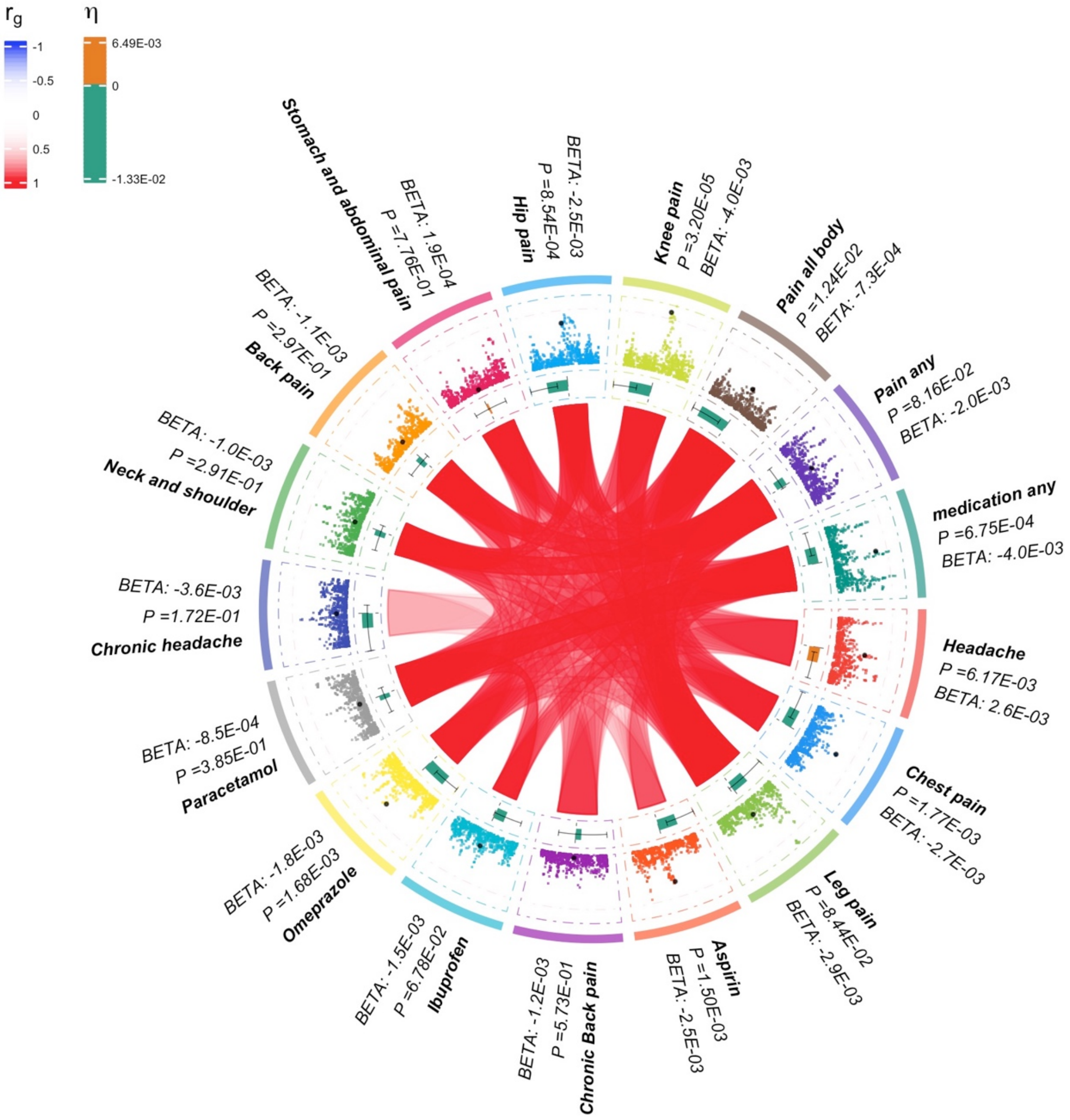

Locus71 12q23.3, rs1895910 Pleio-P= 1.16E-08, intronic to *WSCD2* gene

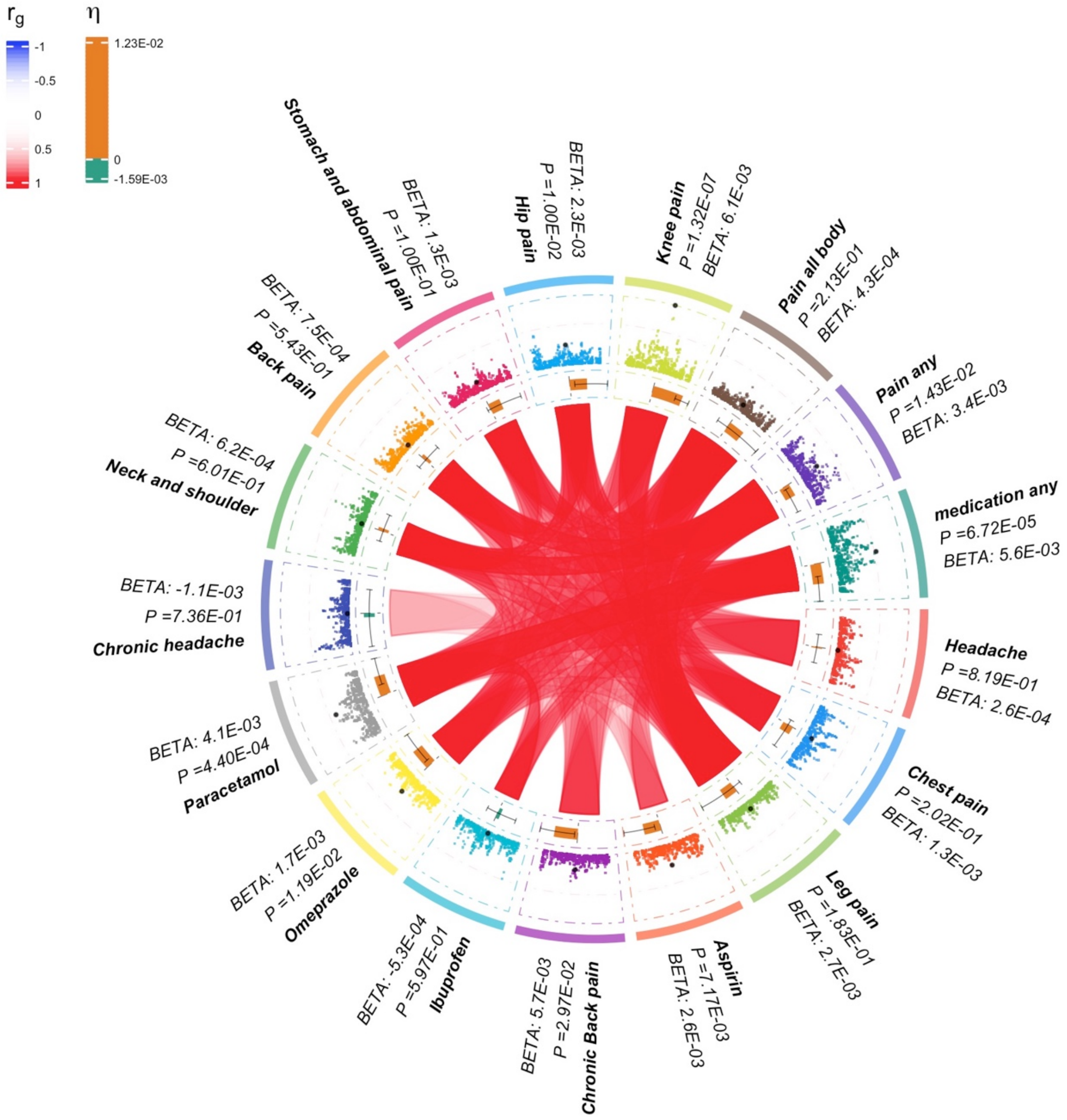

Locus72 12q24.11, rs7959516 Pleio-P= 2.57E-09, intronic to *IFT81* gene

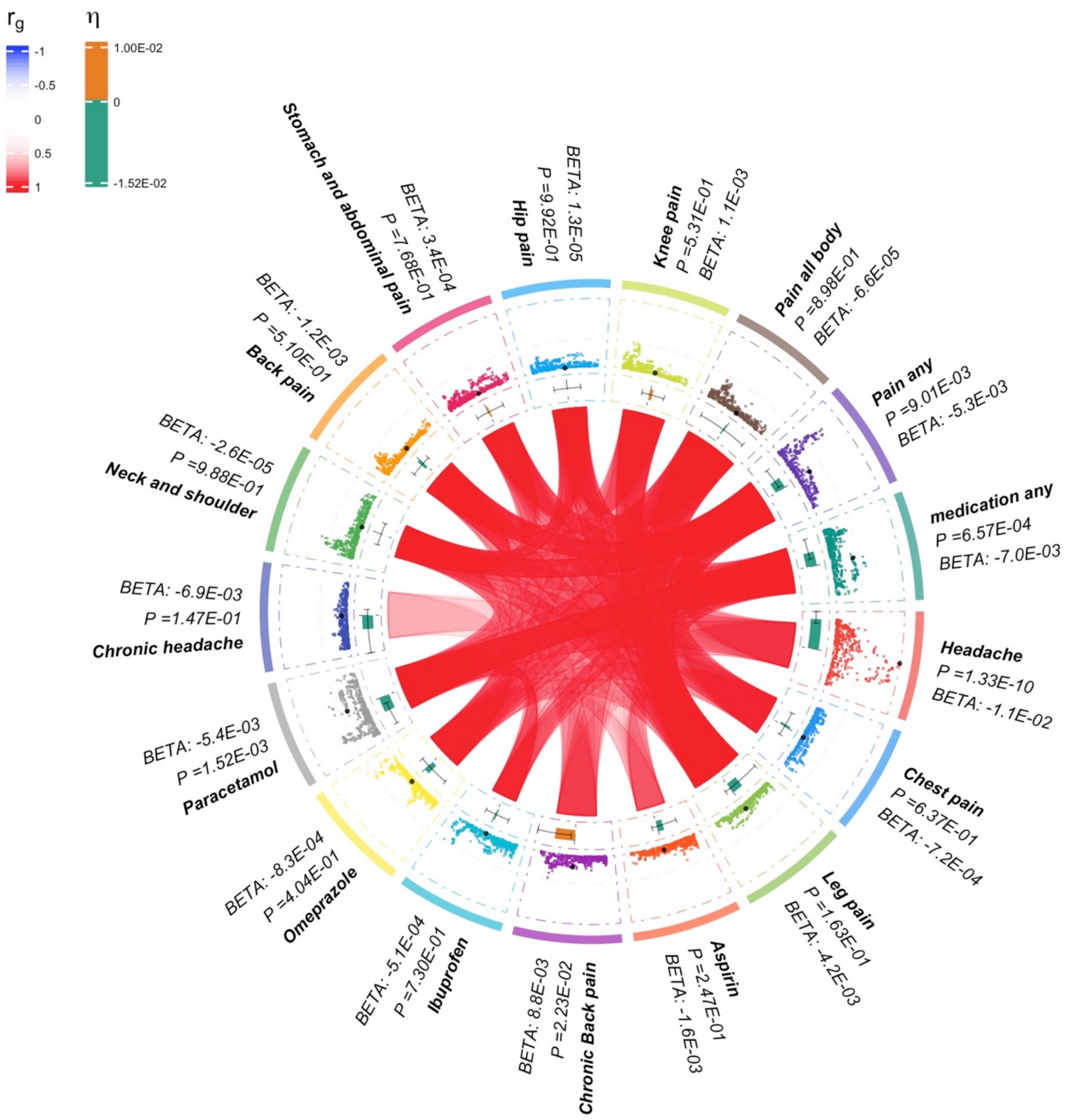

Locus73 12q24.12, rs10774625 Pleio-P= 2.70E-13, intronic to *ATXN2* gene

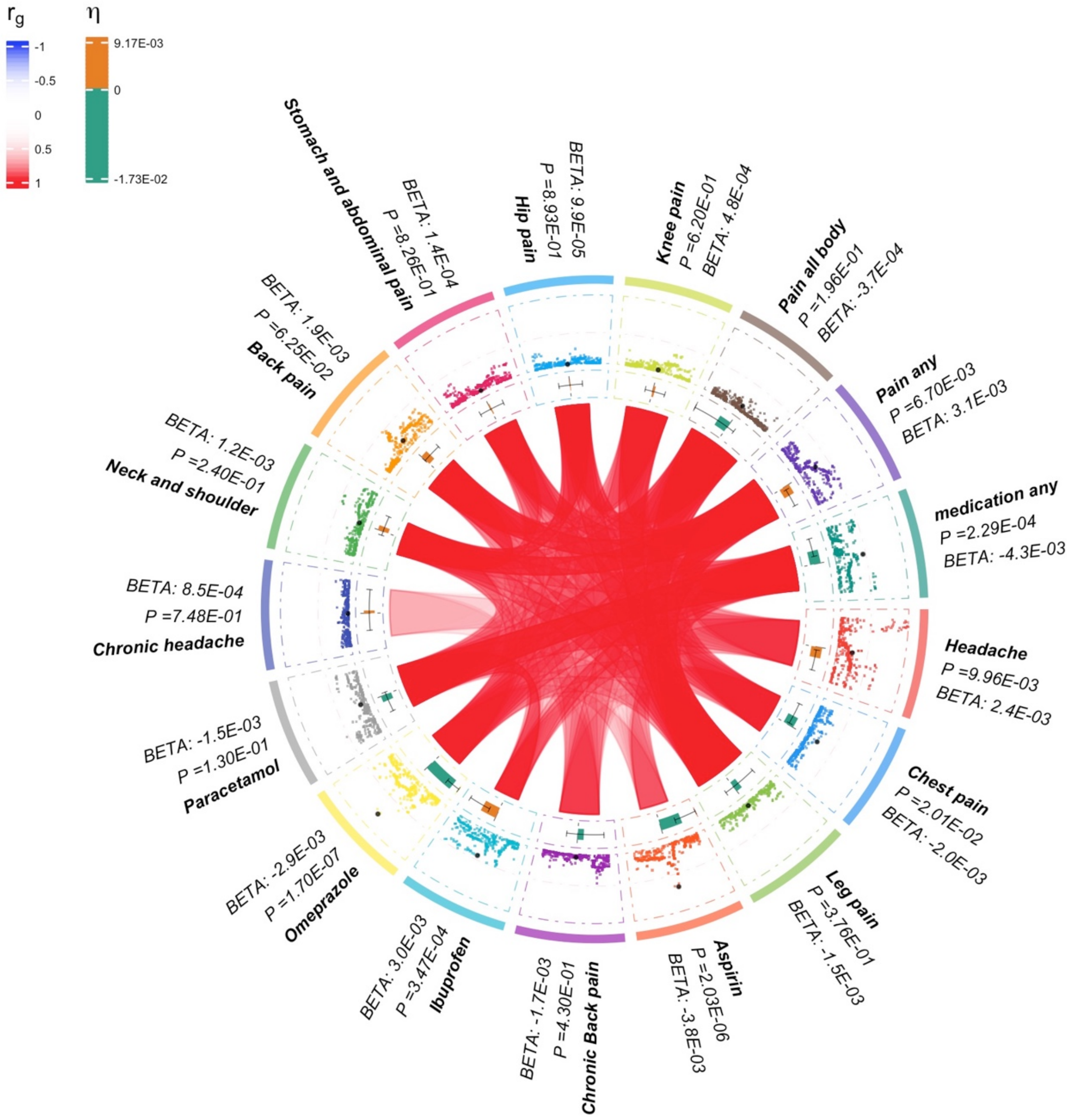

Locus74 13q14.13, rs1570620 Pleio-P= 8.73E-09, intronic to *LRCH1* gene

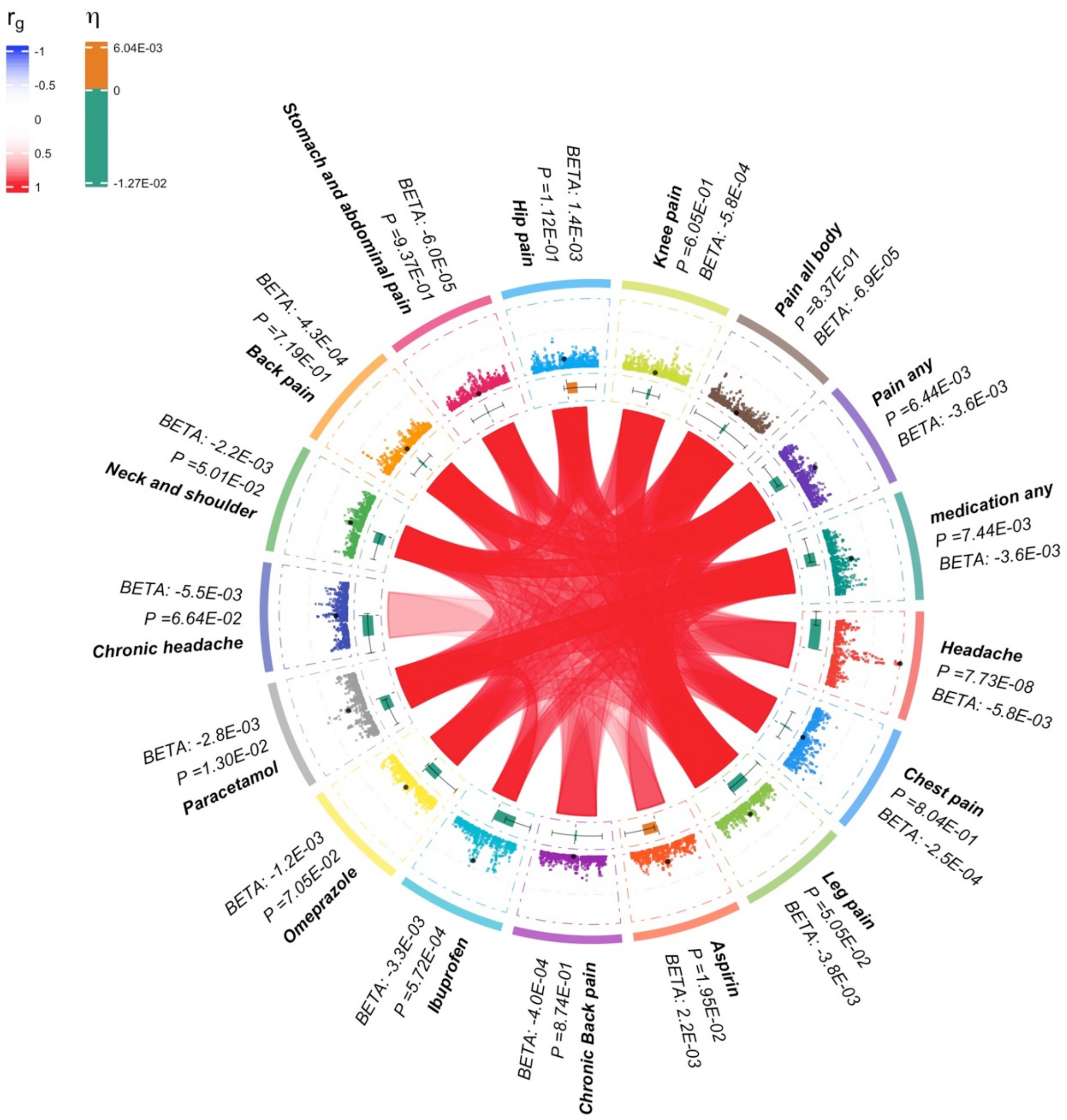

Locus75 13q14.3, rs7335163 Pleio-P= 5.64E-13, intergenic to AL450423.1 gene

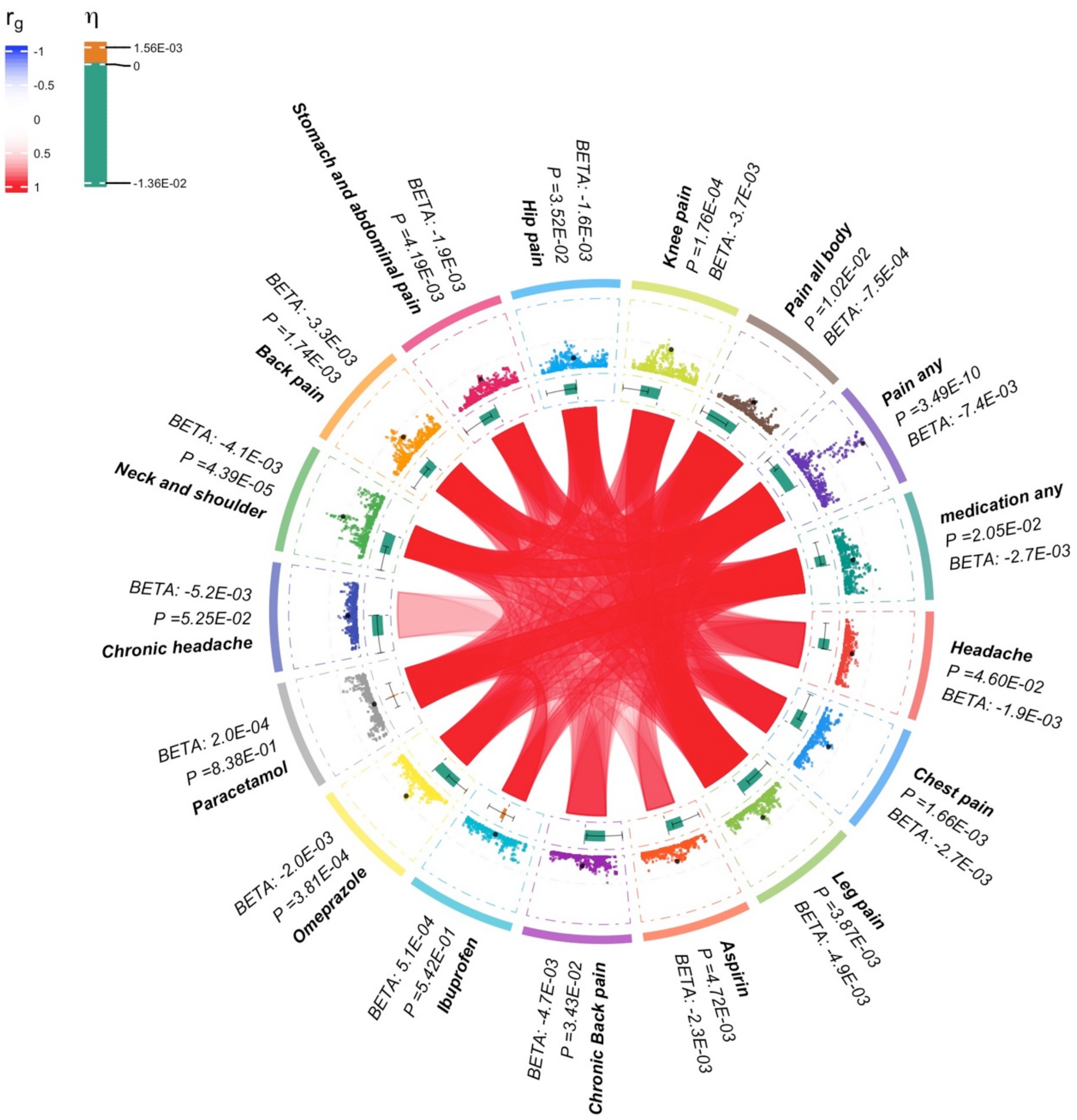

Locus76 13q21.32, rs1413573 Pleio-P= 1.98E-09, intronic to *PCDH9* gene

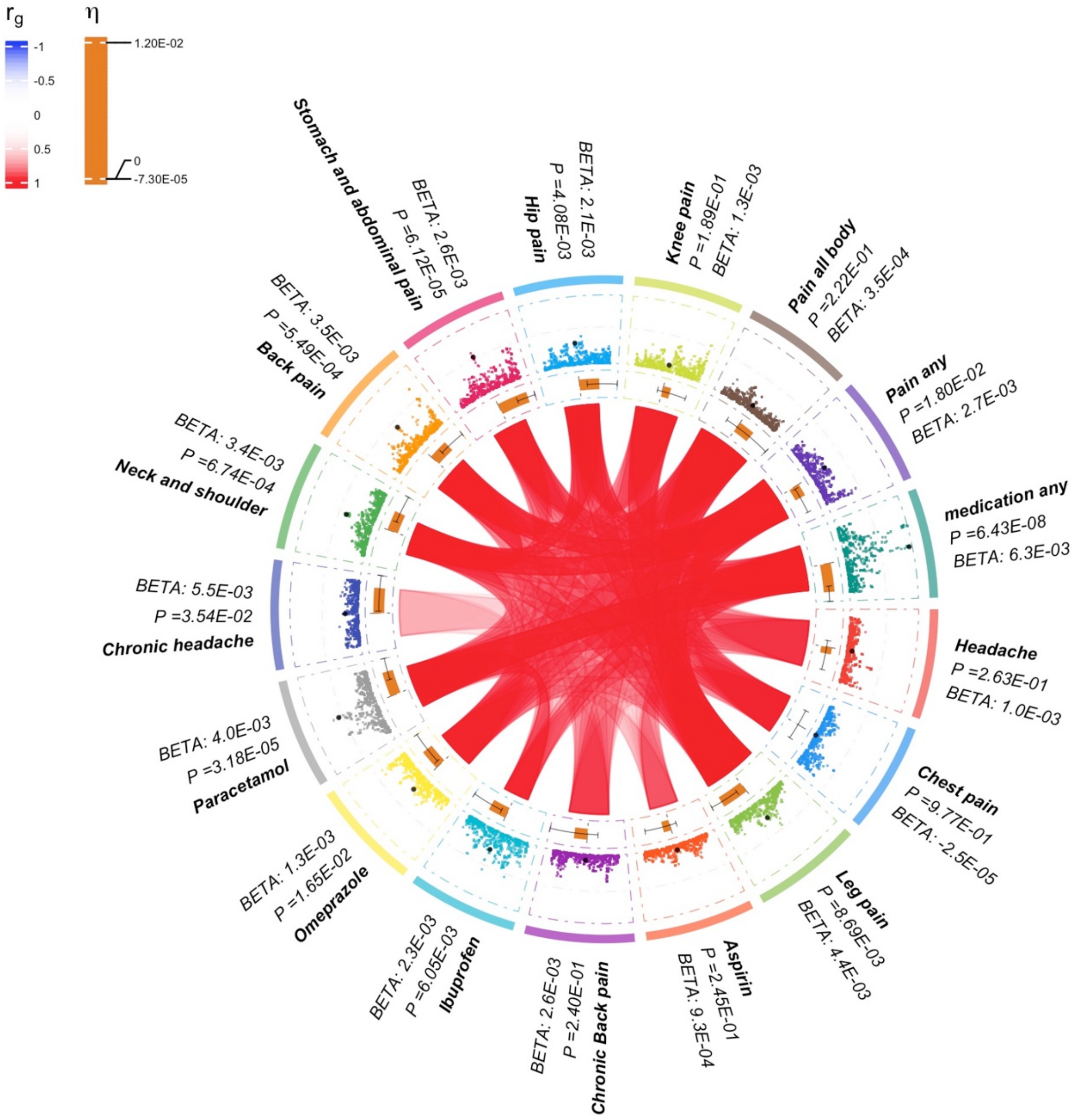

Locus77 14q12, rs1951537 Pleio-P= 2.92E-09, intergenic to RP11-148E17.1 gene

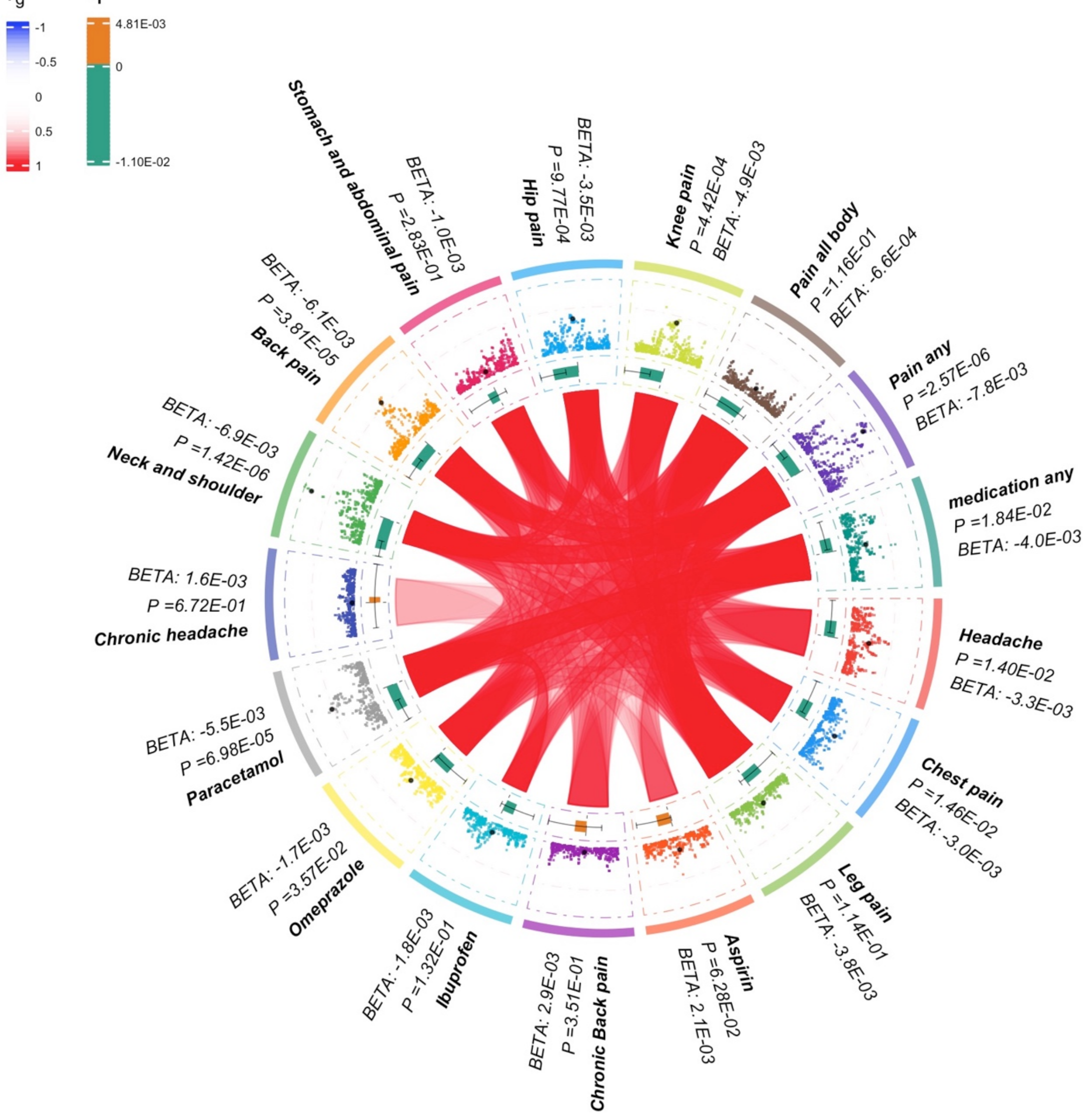

Locus78 14q13.3, rs8018823 Pleio-P= 3.98E-09, intronic to *MIPOL1* gene

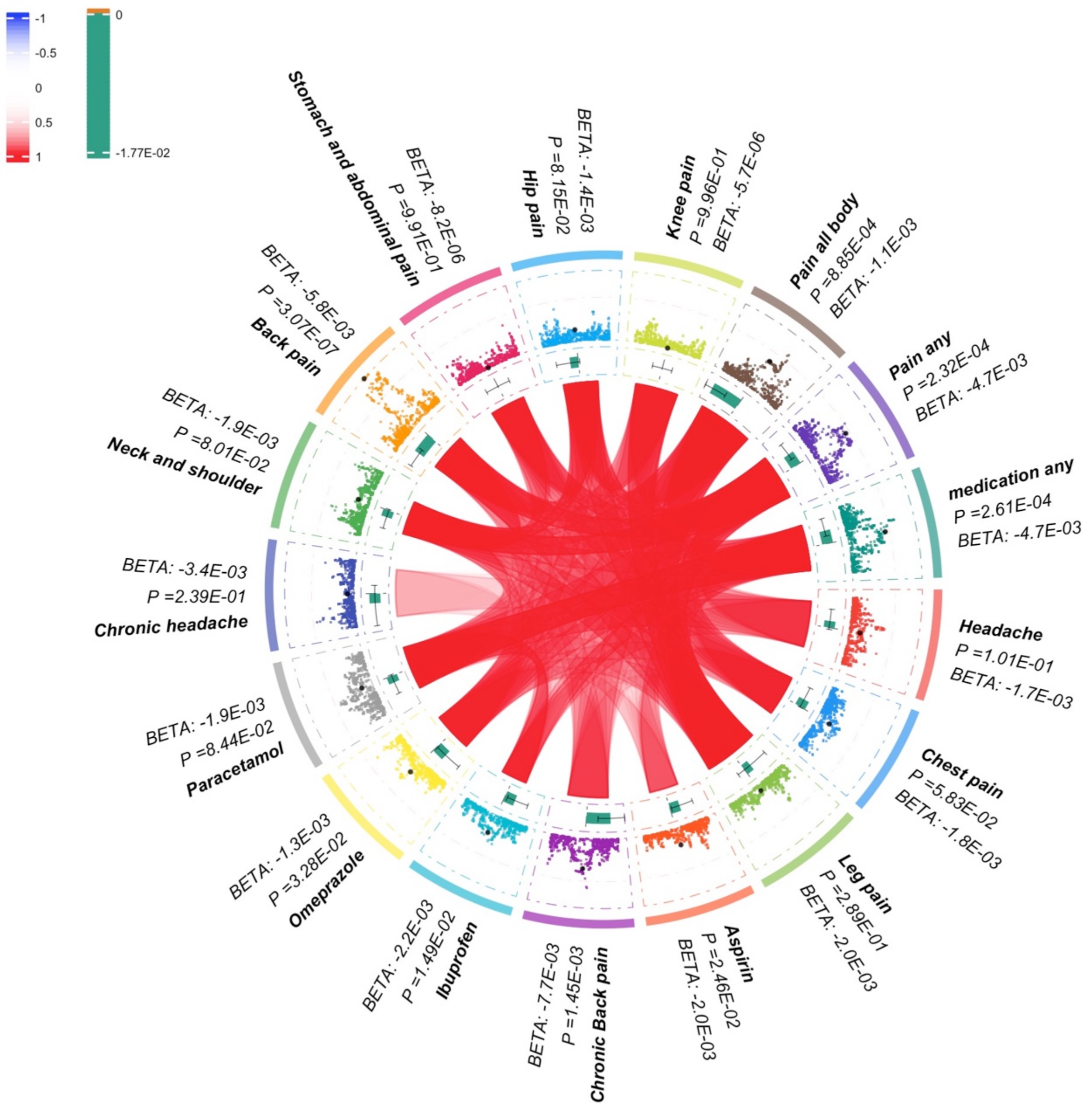

Locus79 14q32.12, rs11624776 Pleio-P= 1.60E-11, intergenic to RP11-371E8.2 gene

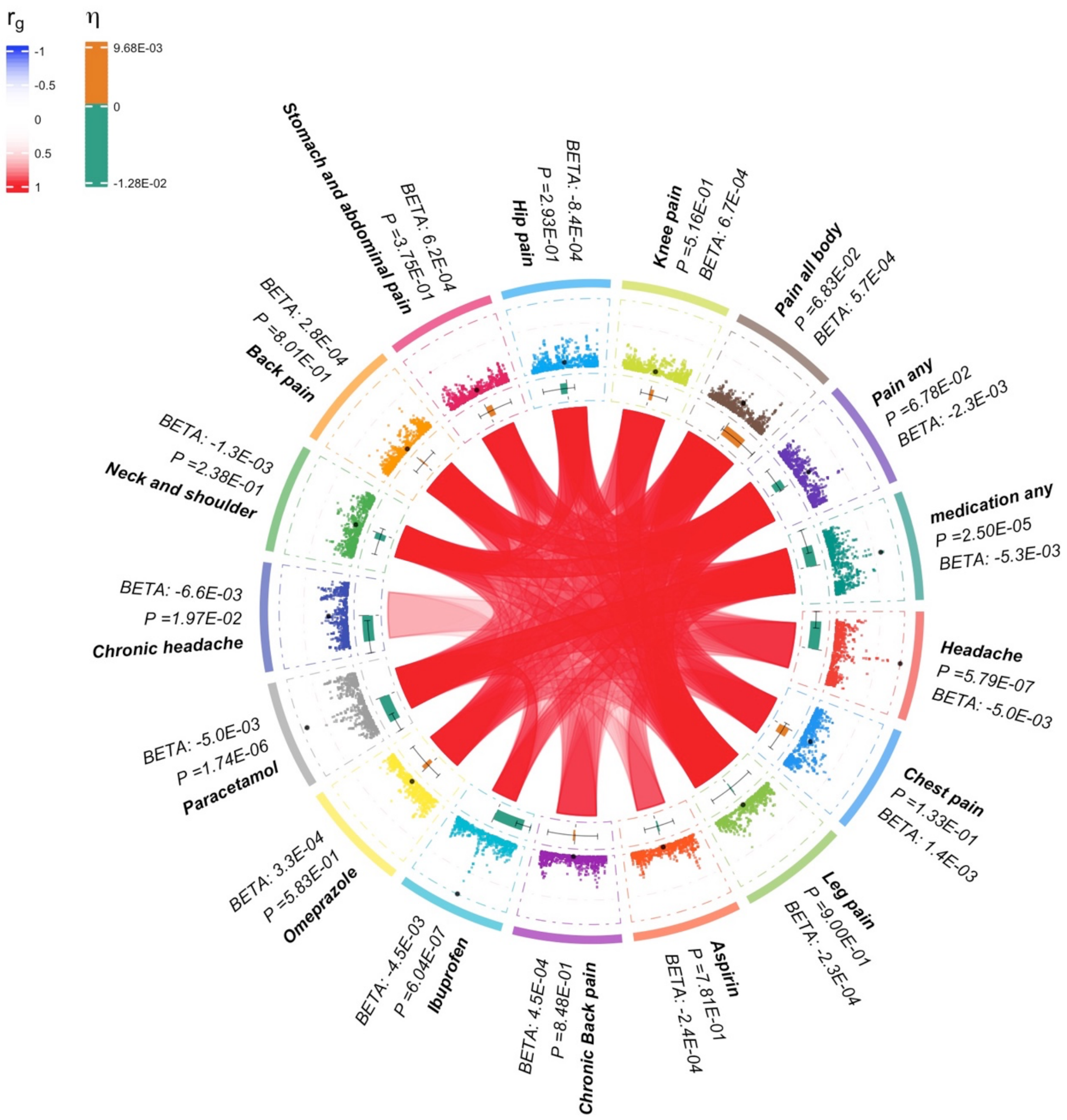

Locus80 15q25.1, rs2627316 Pleio-P= 2.01E-08, intronic to *ABHD17C* gene

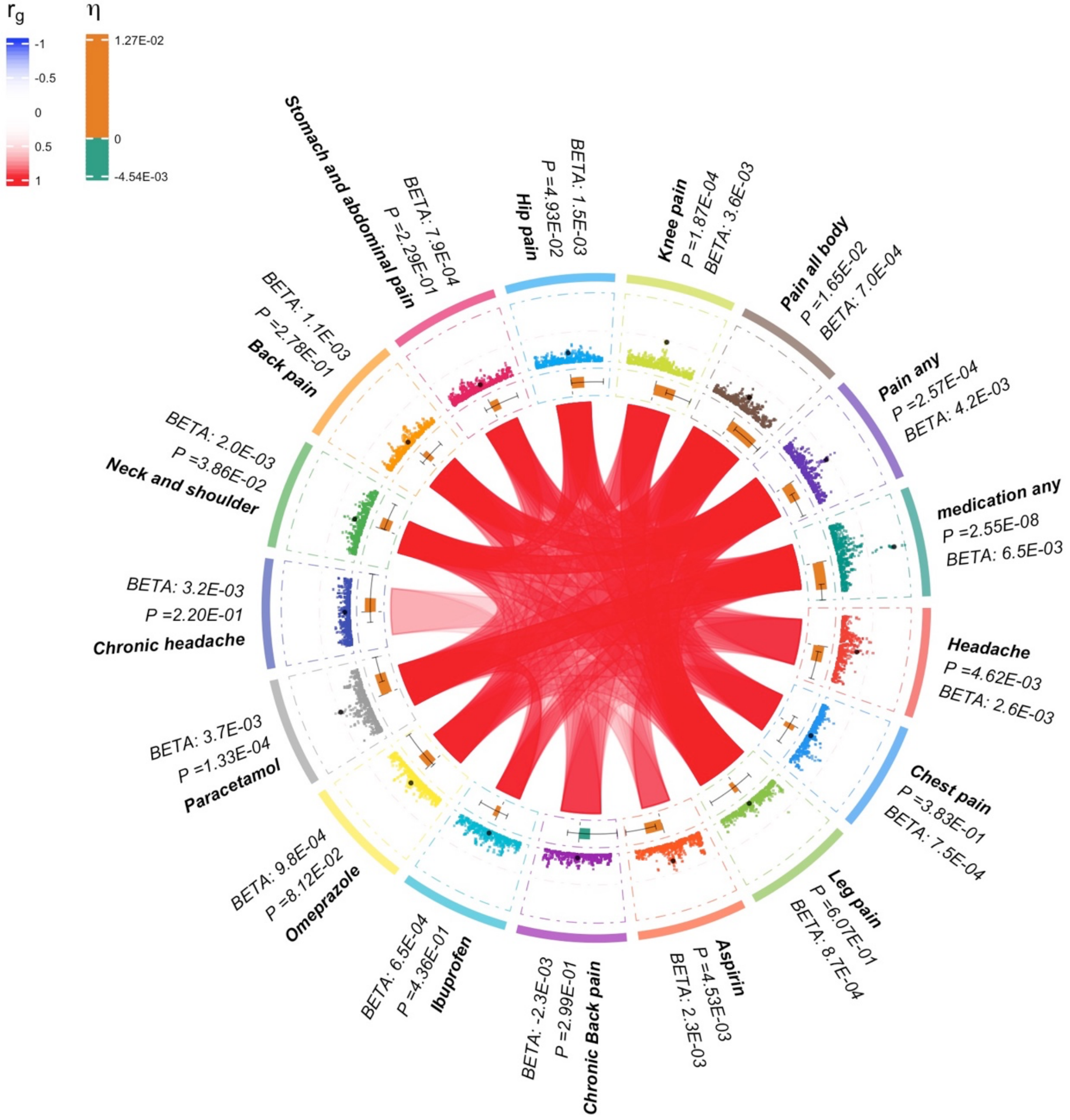

Locus81 15q26.1, rs4244883 Pleio-P= 3.71E-08, intronic to *VPS33B* gene

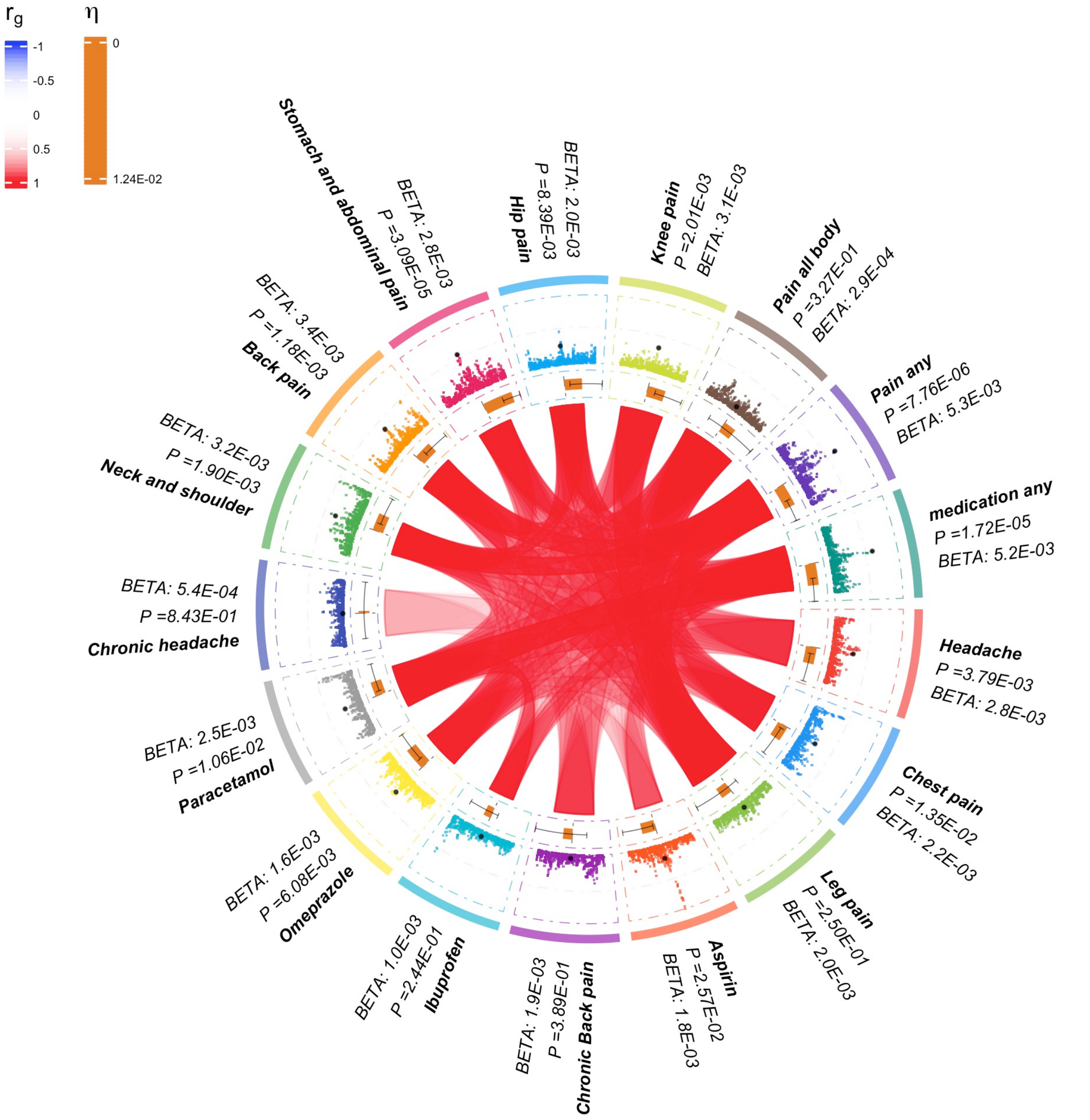

Locus82 16q22.2, rs8057124 Pleio-P= 2.90E-08, ncRNA_intronic to *PKD1L3* gene

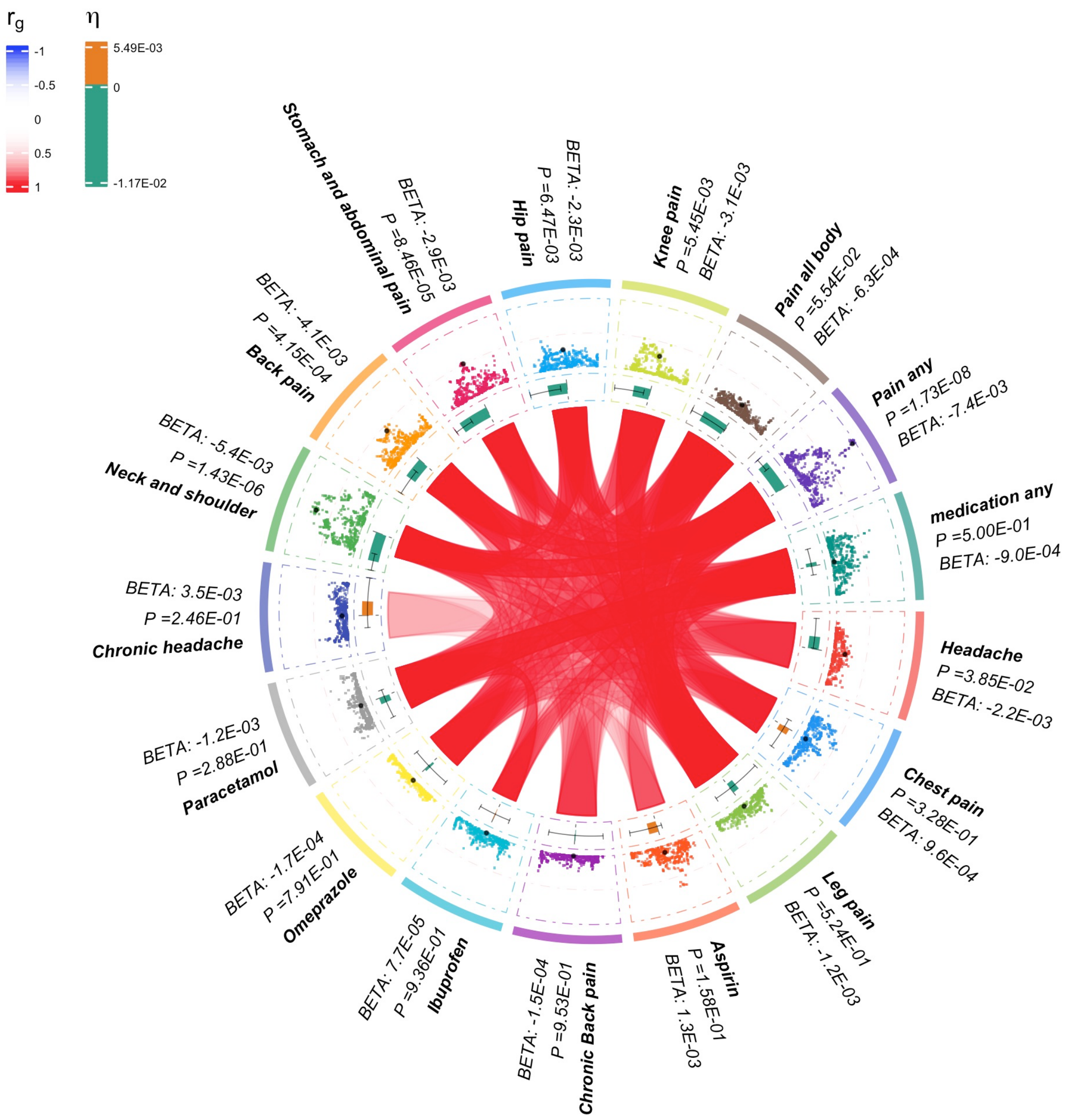

Locus83 16q23.1, rs8046109 Pleio-P= 1.21E-10, intronic to *CFDP1* gene

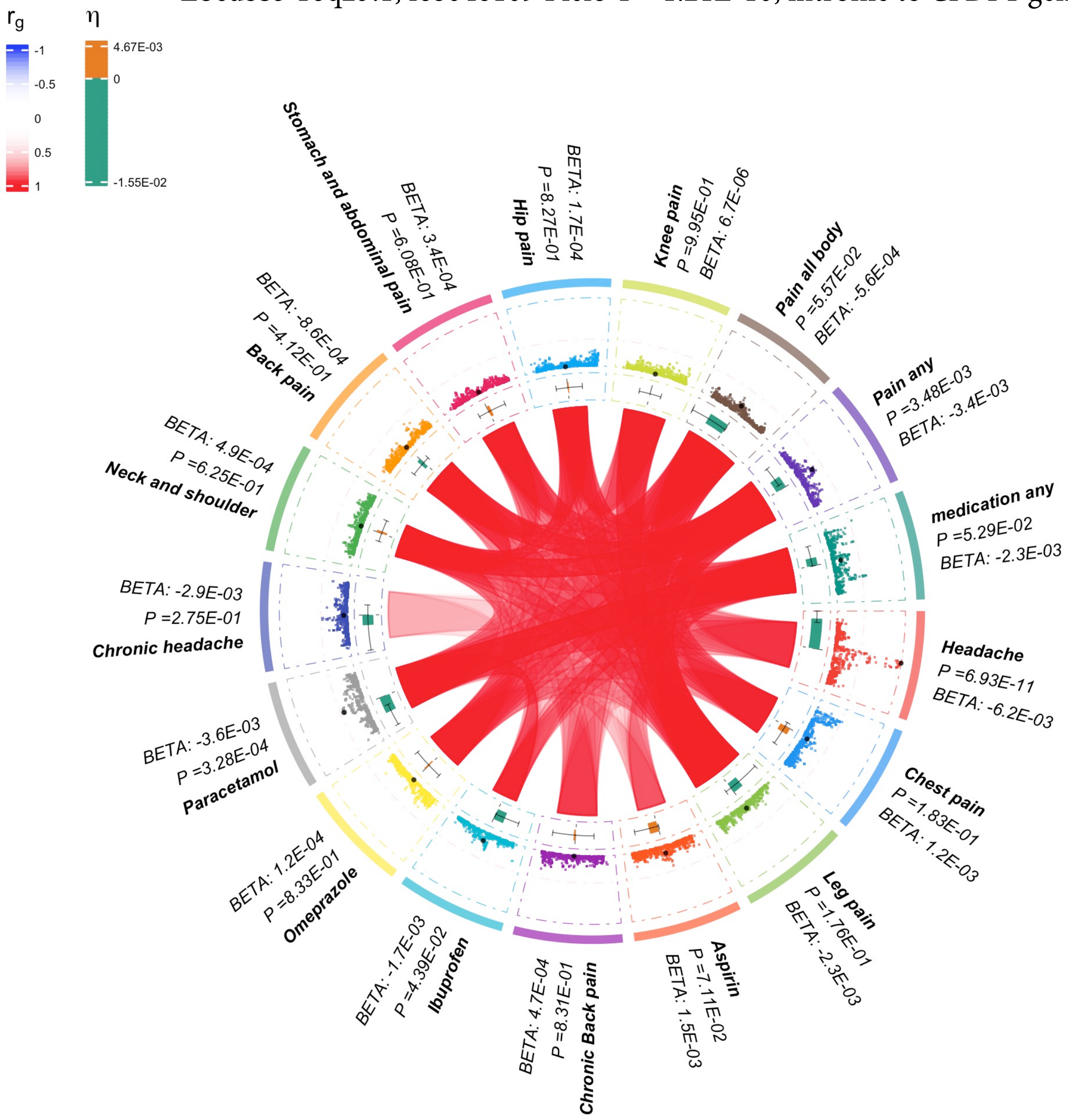

Locus84 16q24.2, rs12445022 Pleio-P= 3.27E-09, intergenic to RP11-482M8.1 gene

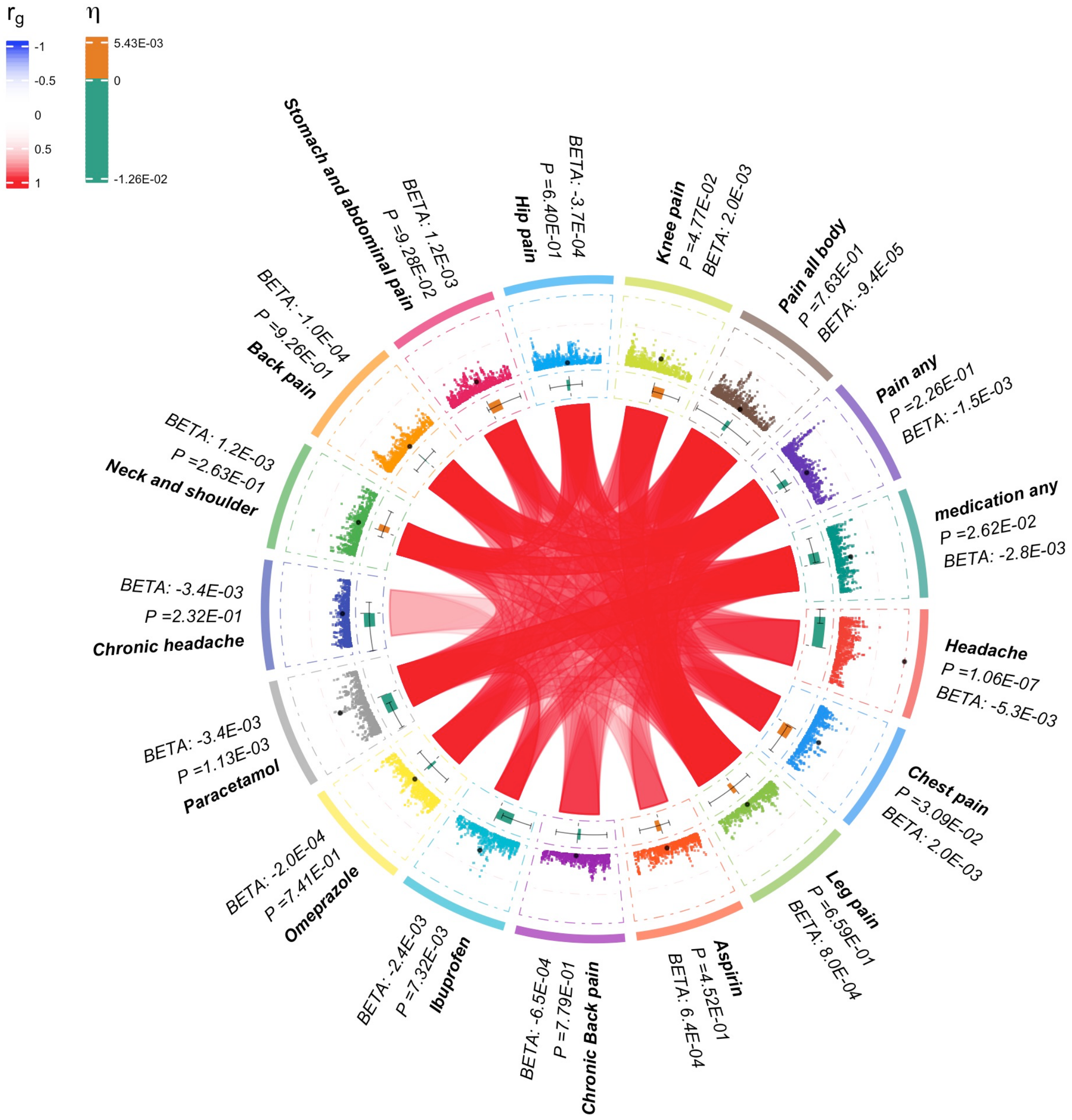

Locus85 16q24.3, rs164741 Pleio-P= 4.38E-10, intronic to *DPEP1* gene

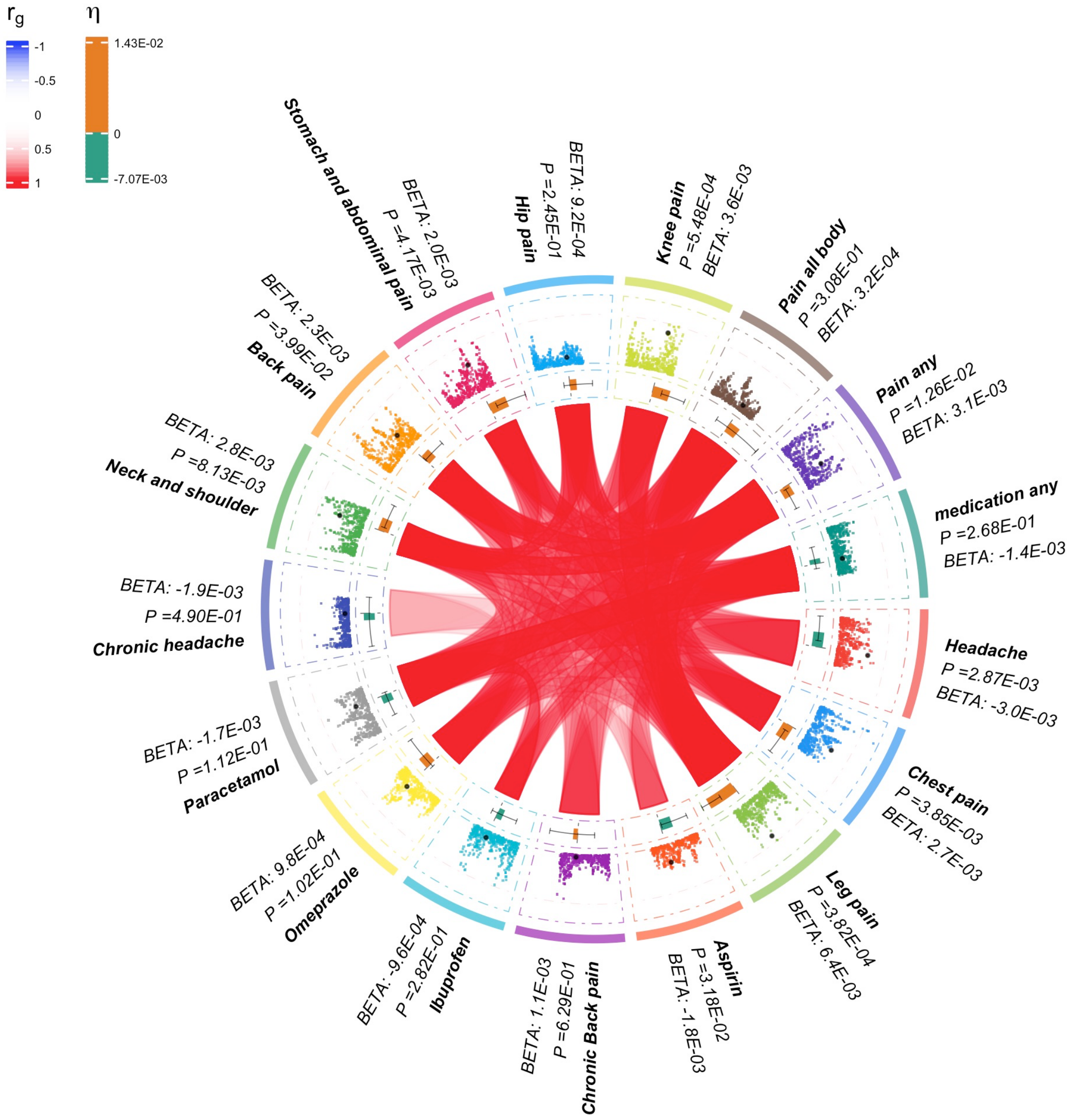

Locus86 17q11.2, rs8614 Pleio-P= 3.09E-08, UTR3 to *NUFIP2* gene

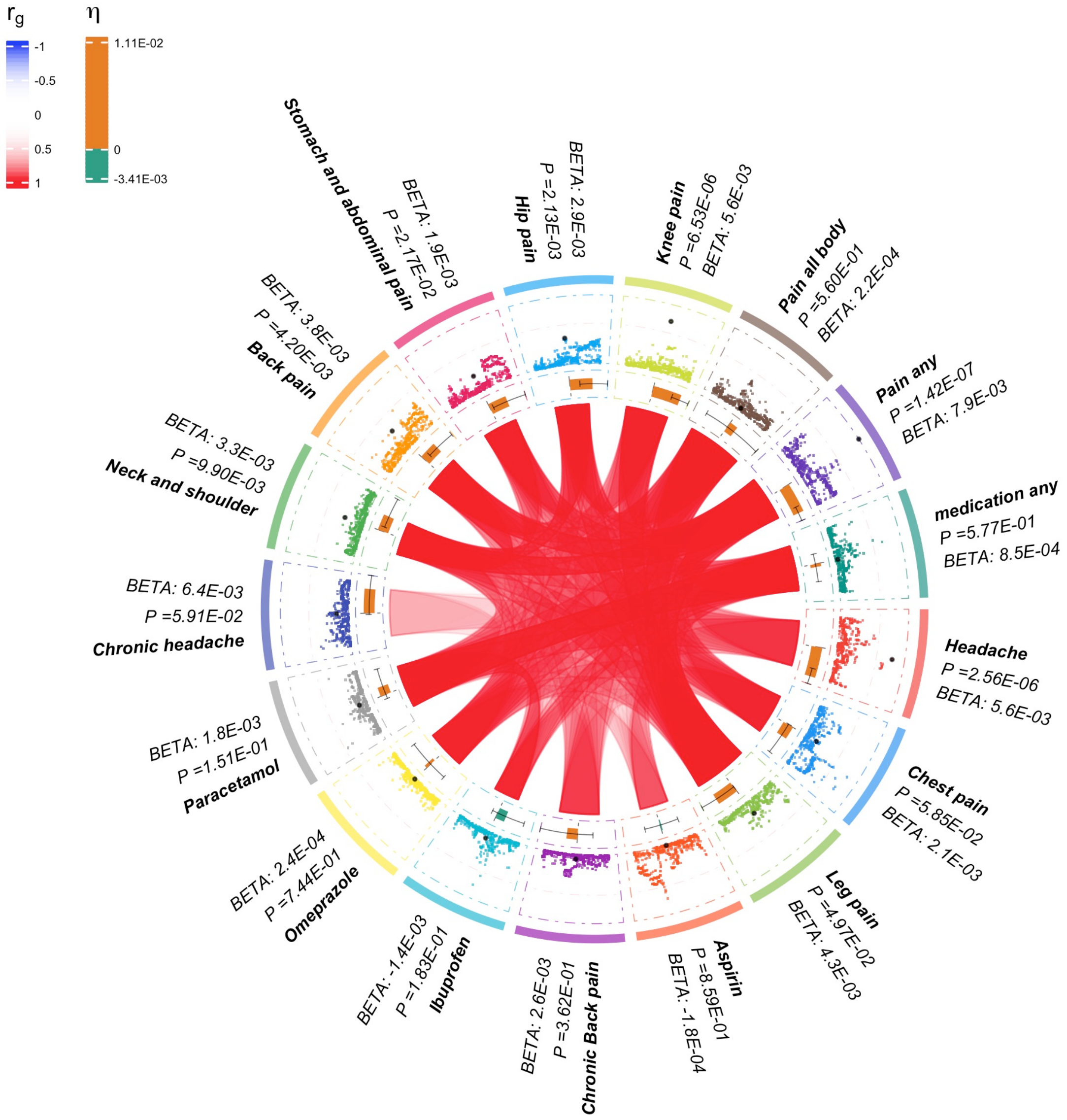

Locus87 17q21.31, rs17689882 Pleio-P= 1.96E-28, intronic to *CRHR1* gene

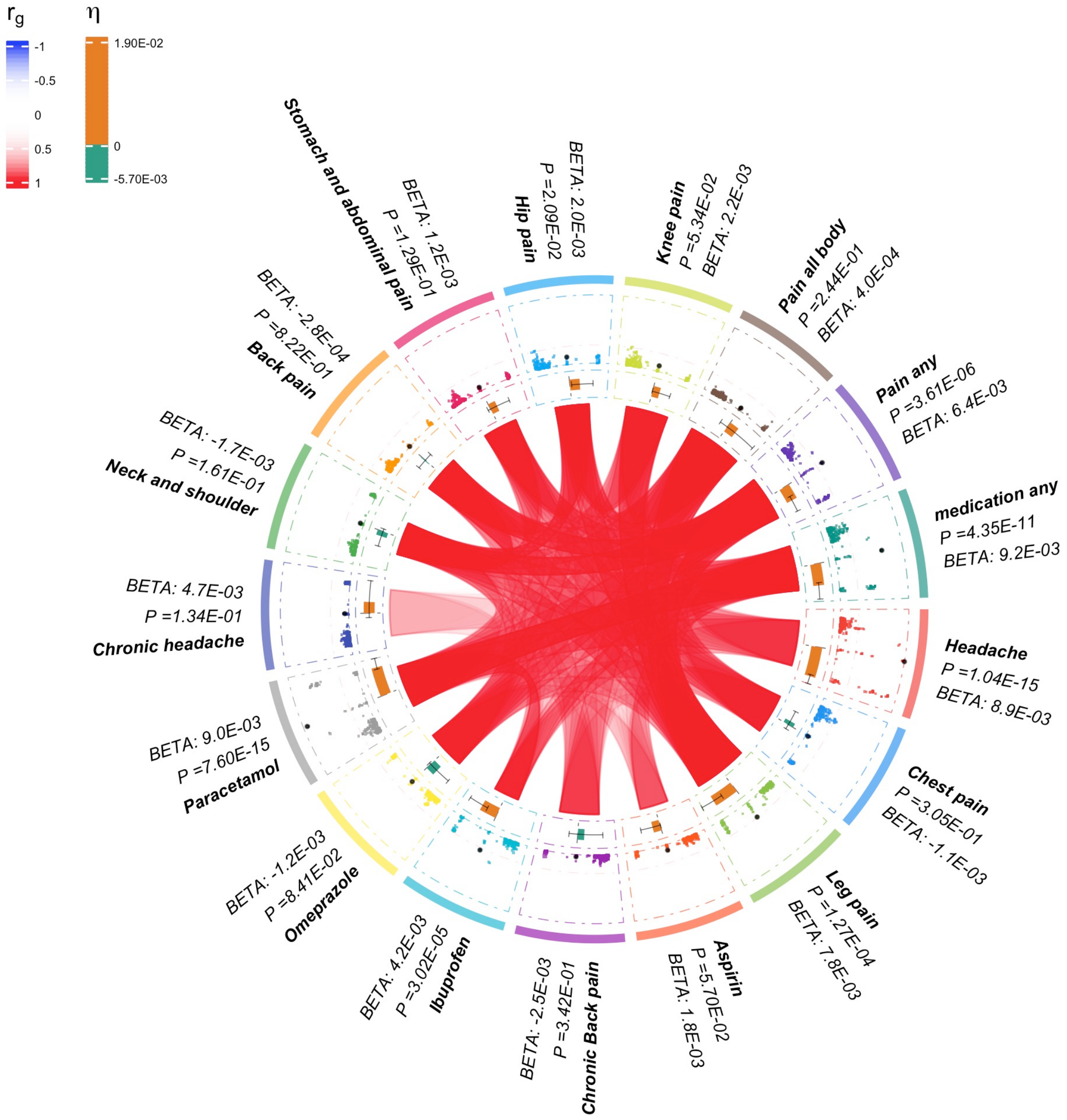

Locus88 17q21.33, rs7222591 Pleio-P= 4.23E-10, intronic to RP11-81K2.1 gene

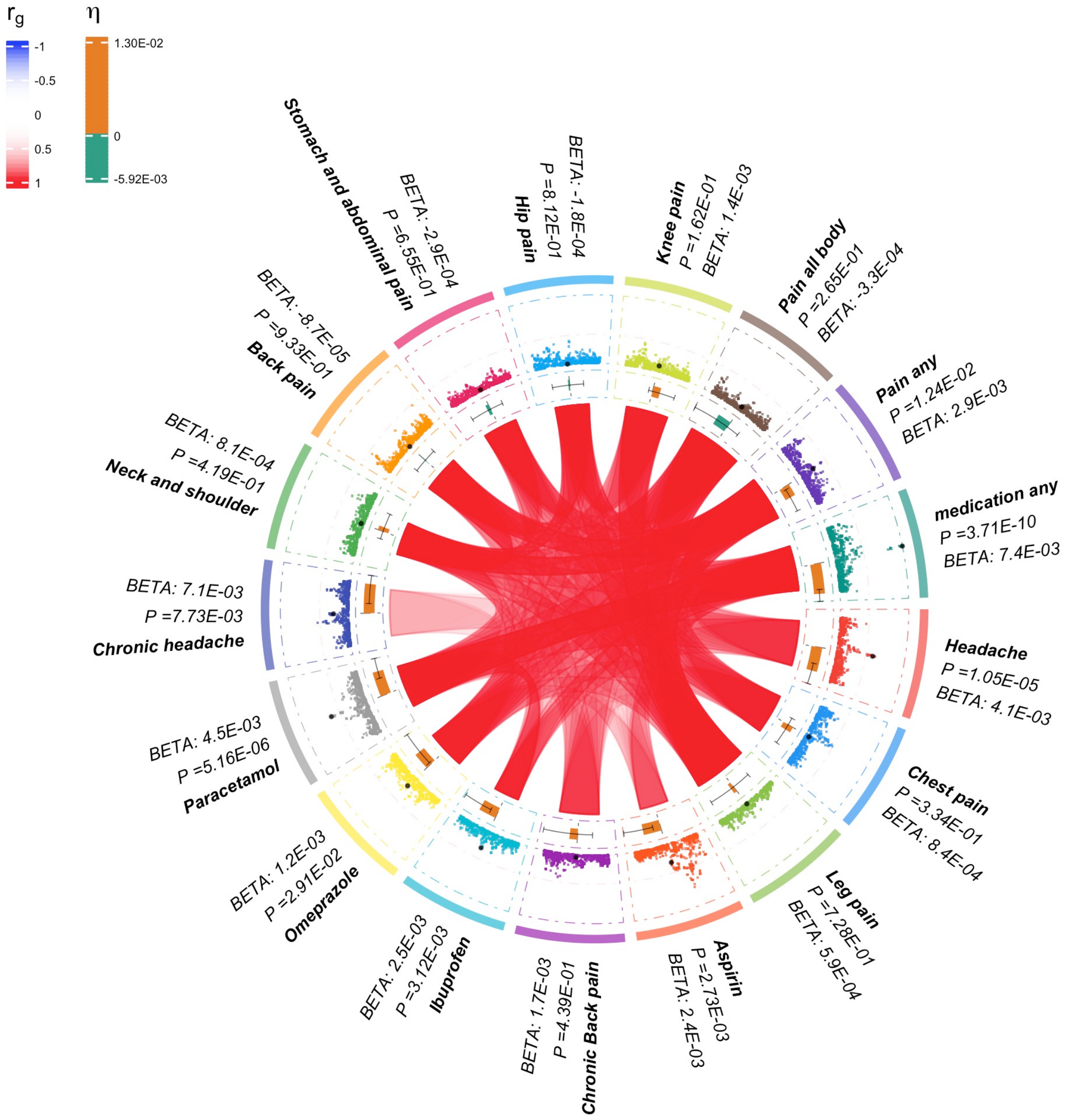

Locus89 17q22, rs967823 Pleio-P= 1.94E-16, intergenic to snoZ178 gene

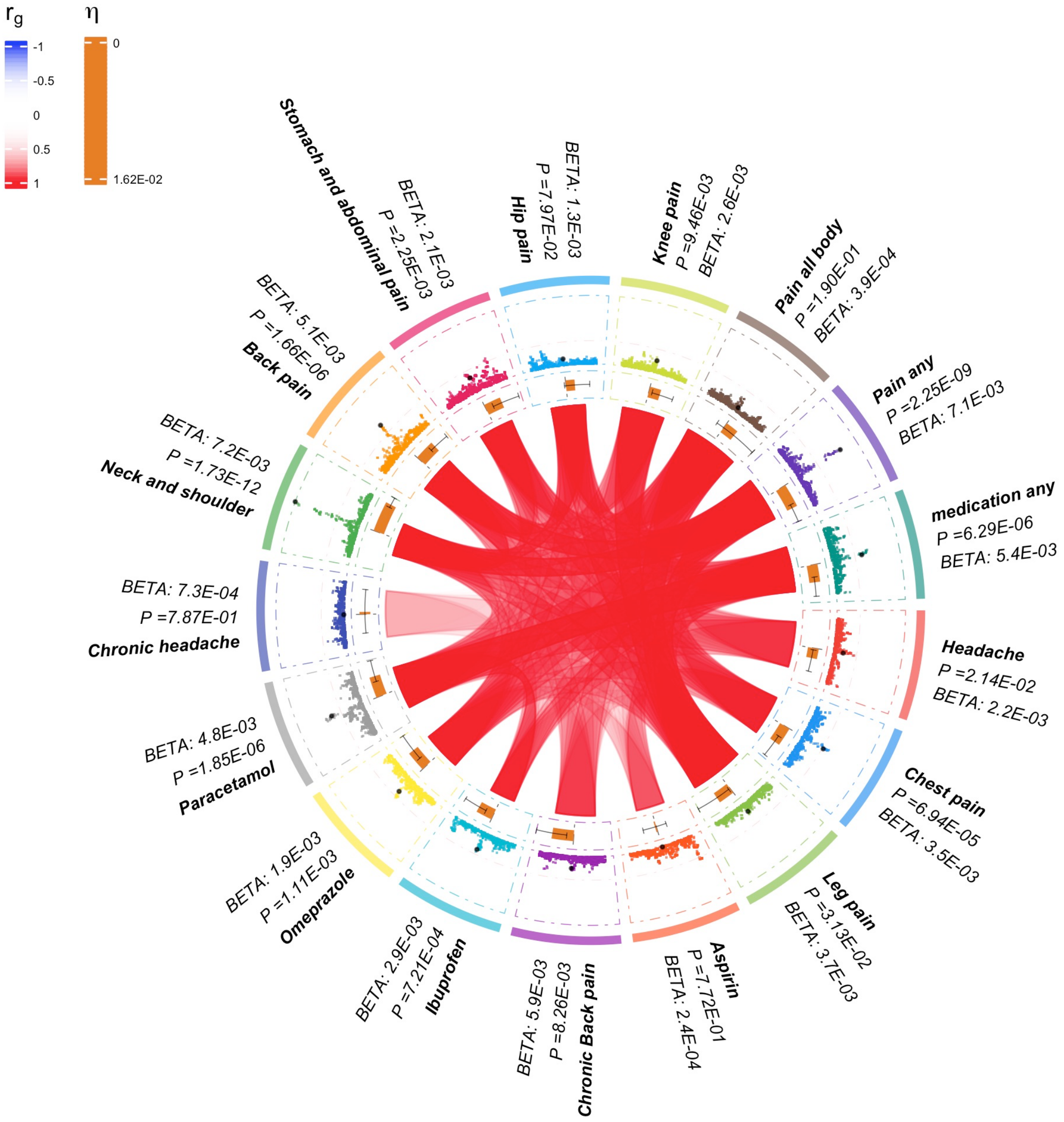

Locus90 18q11.2, rs9653003 Pleio-P= 1.18E-10, intergenic to RPS4XP18 gene

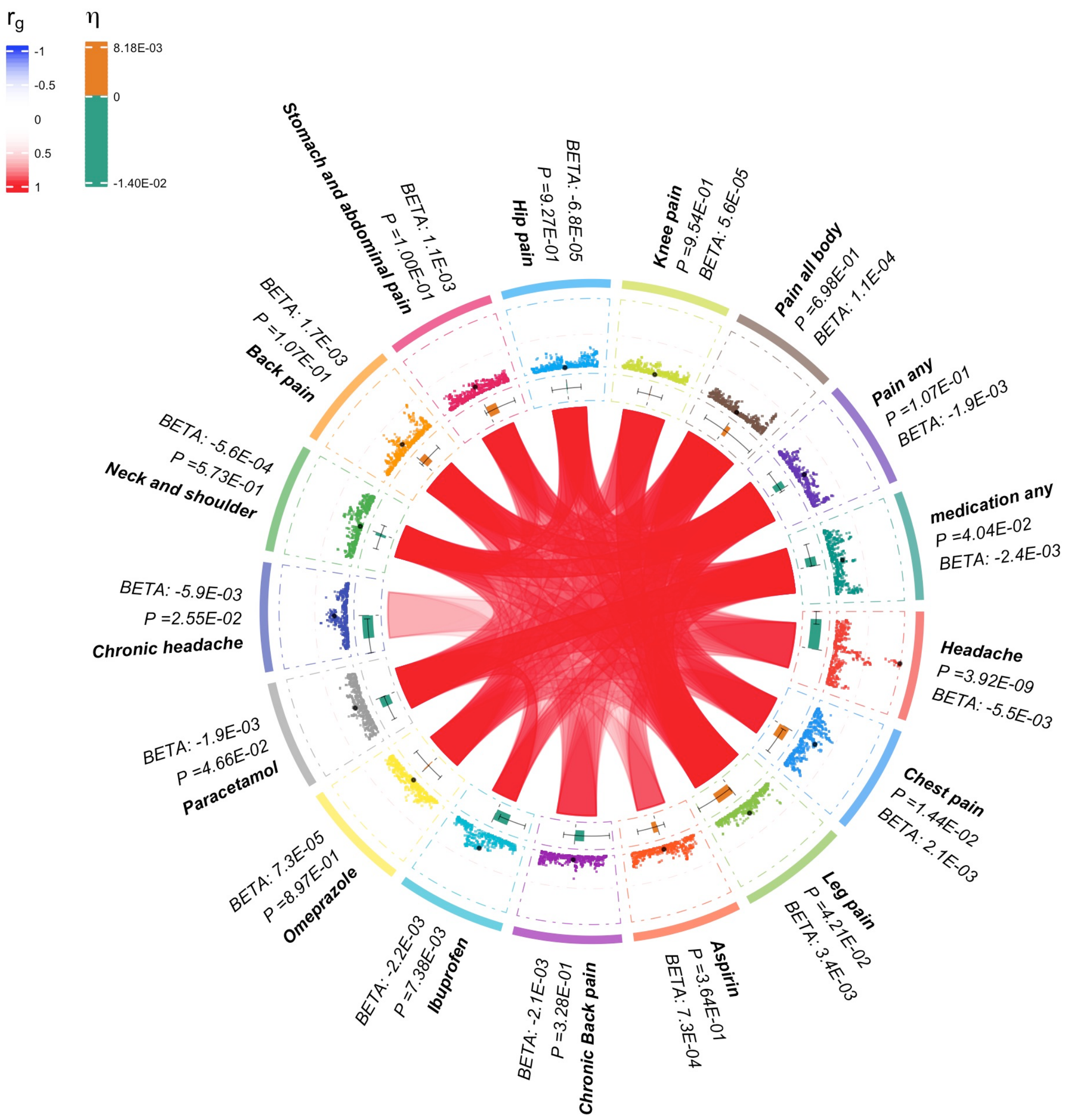

Locus91 18q12.2, rs11665070 Pleio-P= 2.05E-09, intergenic to *CELF4* gene

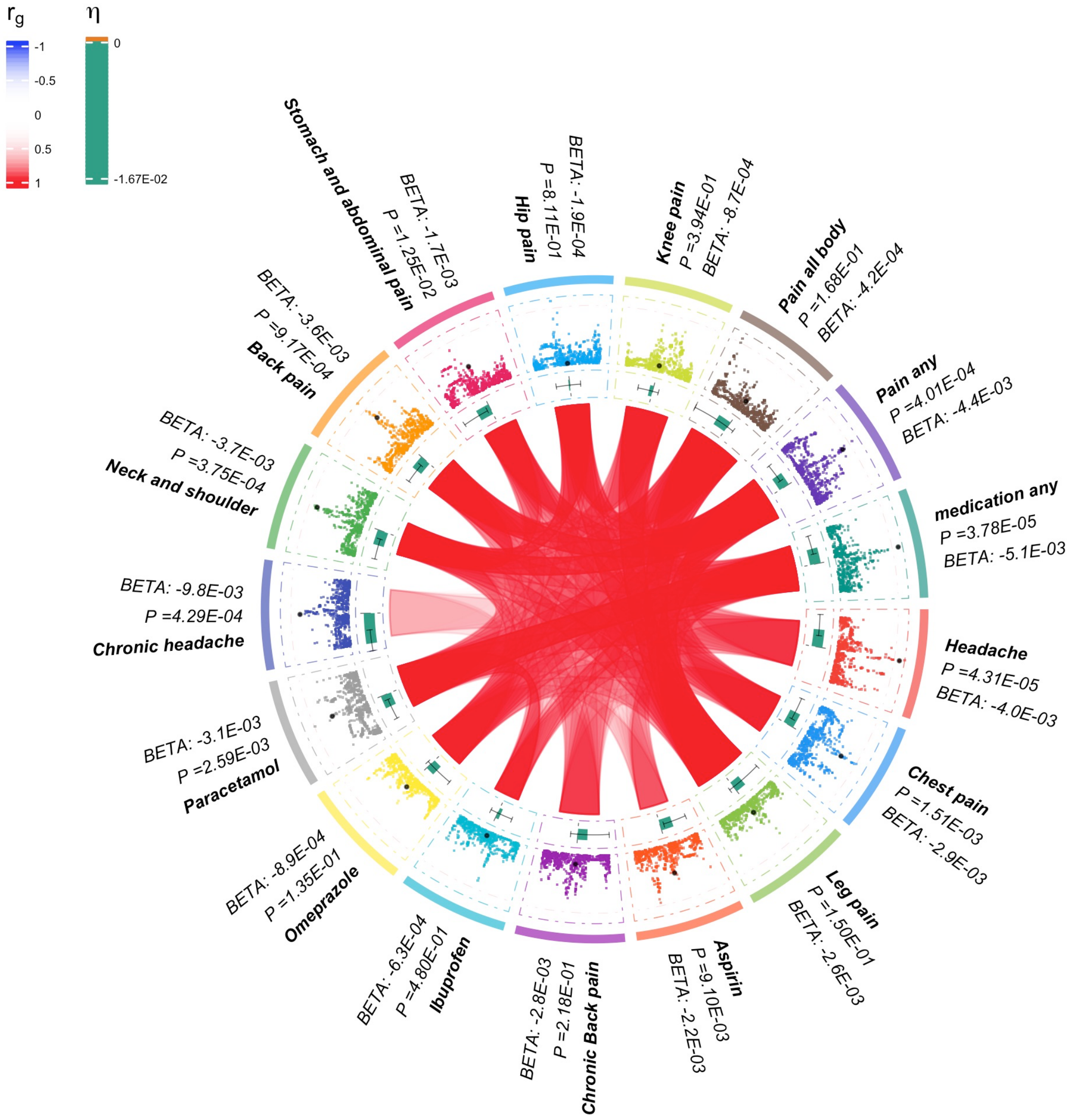

Locus92 18q21.2, rs1431196 Pleio-P= 1.19E-08, intronic to *DCC* gene

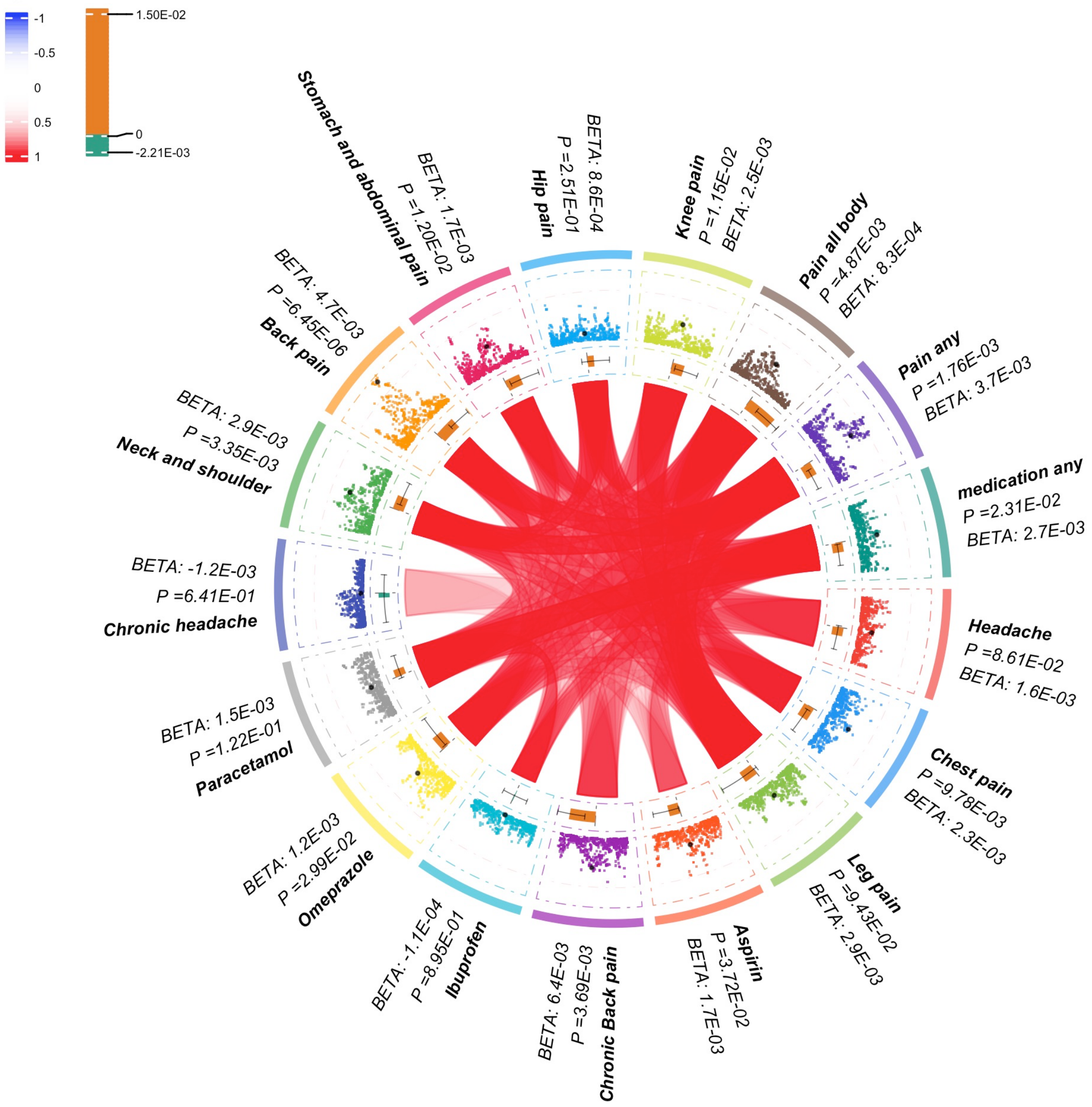

Locus93 18q21.2, rs618869 Pleio-P= 4.14E-08, intronic to *TCF4* gene

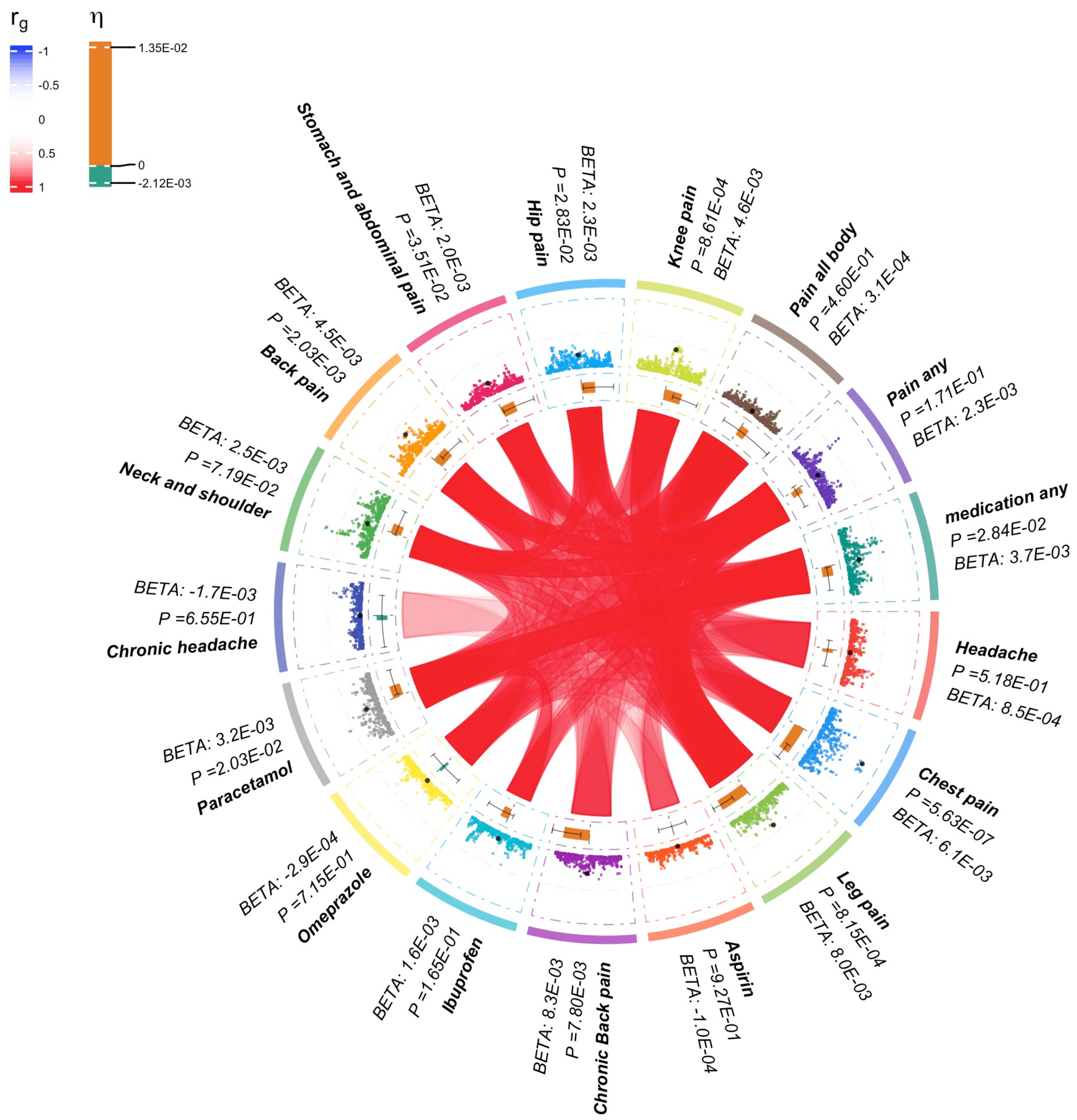

Locus94 18q21.31, rs615030 Pleio-P= 1.75E-09, UTR3 to *ONECUT2* gene

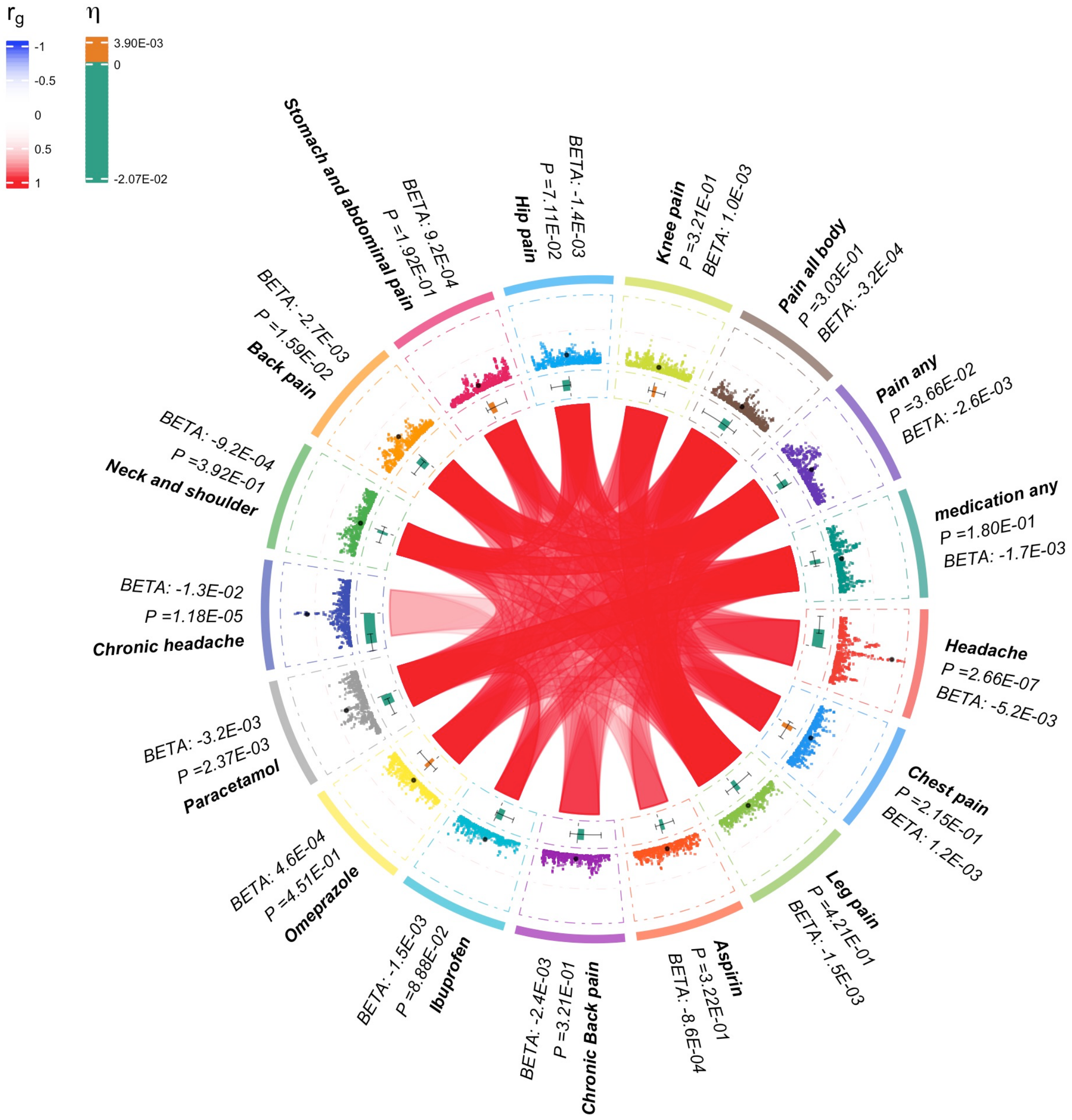

Locus95 19q13.32, rs4420638 Pleio-P= 4.18E-09, downstream to *APOC1* gene

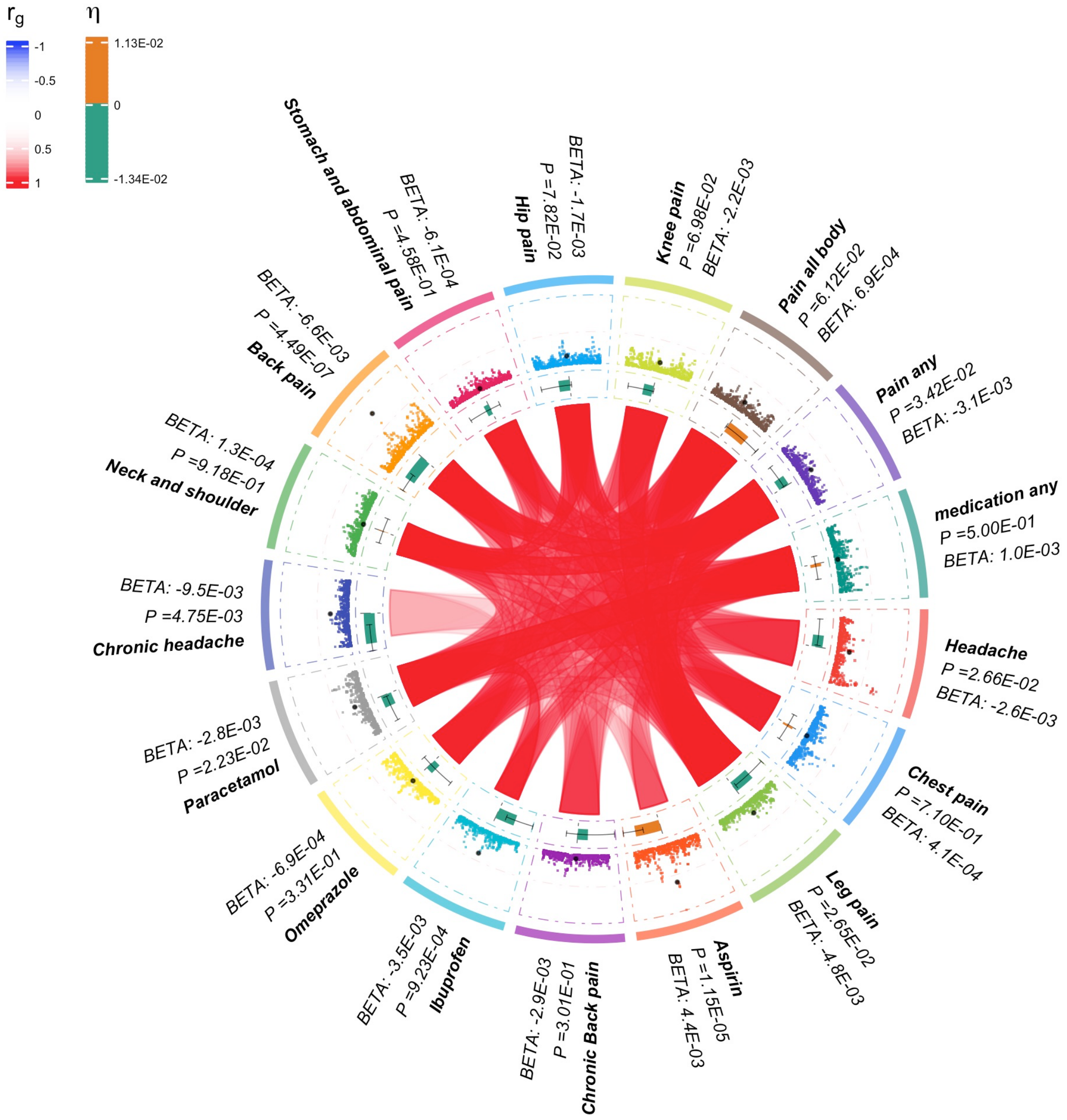

Locus96 20p11.23, rs2424245 Pleio-P= 2.73E-08, intronic to *SLC24A3* gene

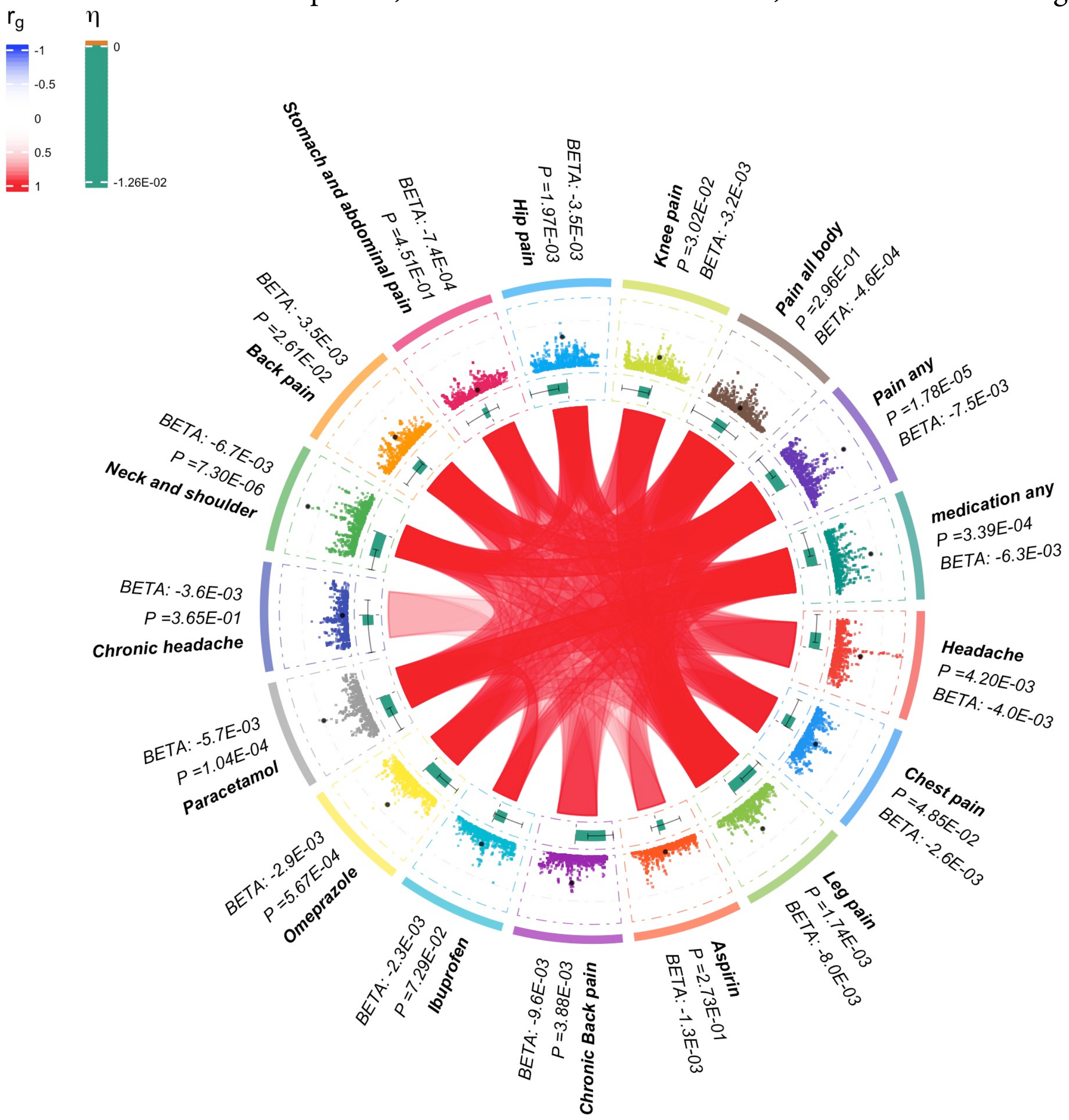

Locus97 20q11.21, rs6119879 Pleio-P= 5.17E-10, intronic to *C20orf112* gene

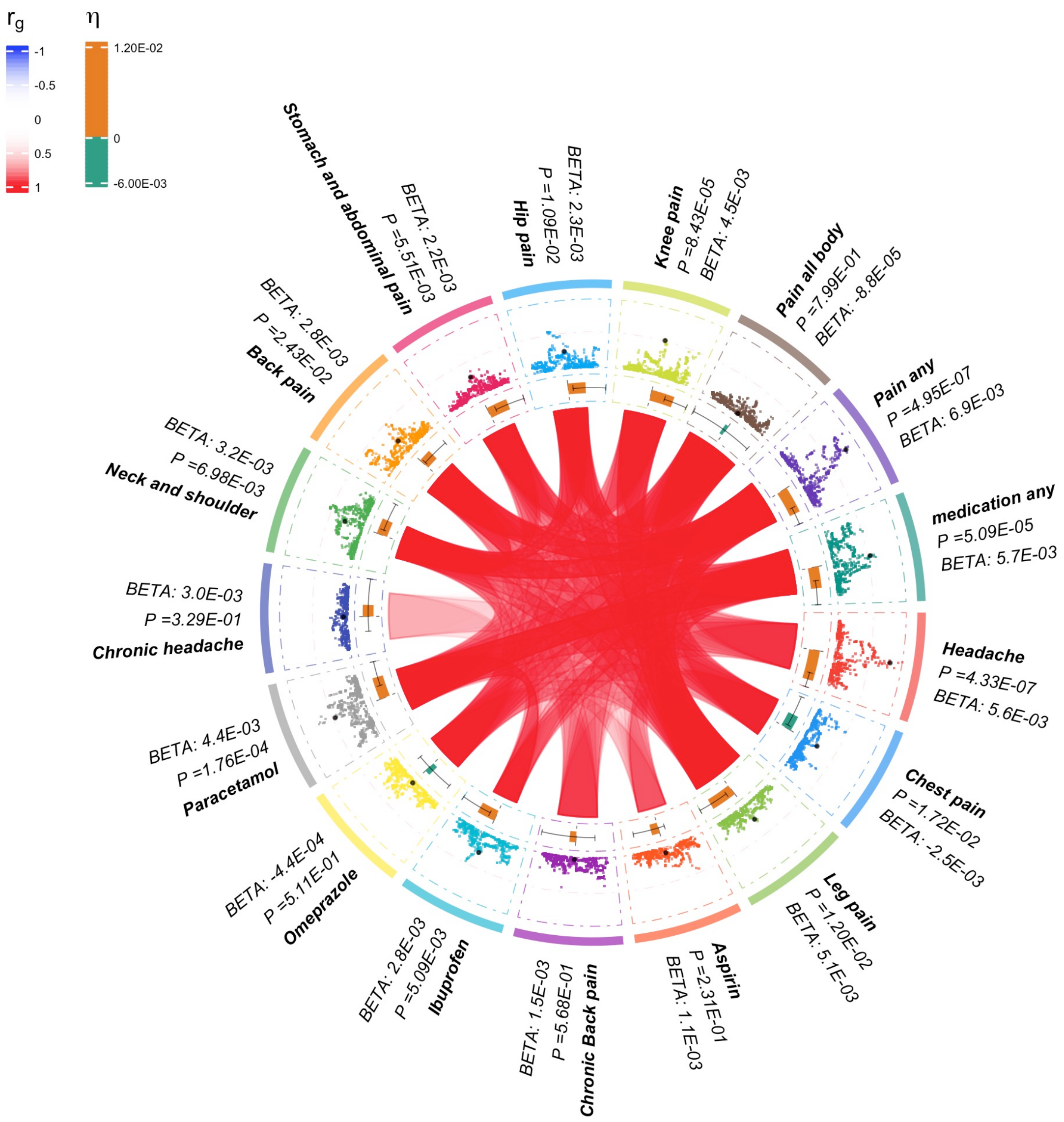

Locus98 20q11.22, rs143384 Pleio-P= 2.80E-17, UTR5 to *GDF5* gene

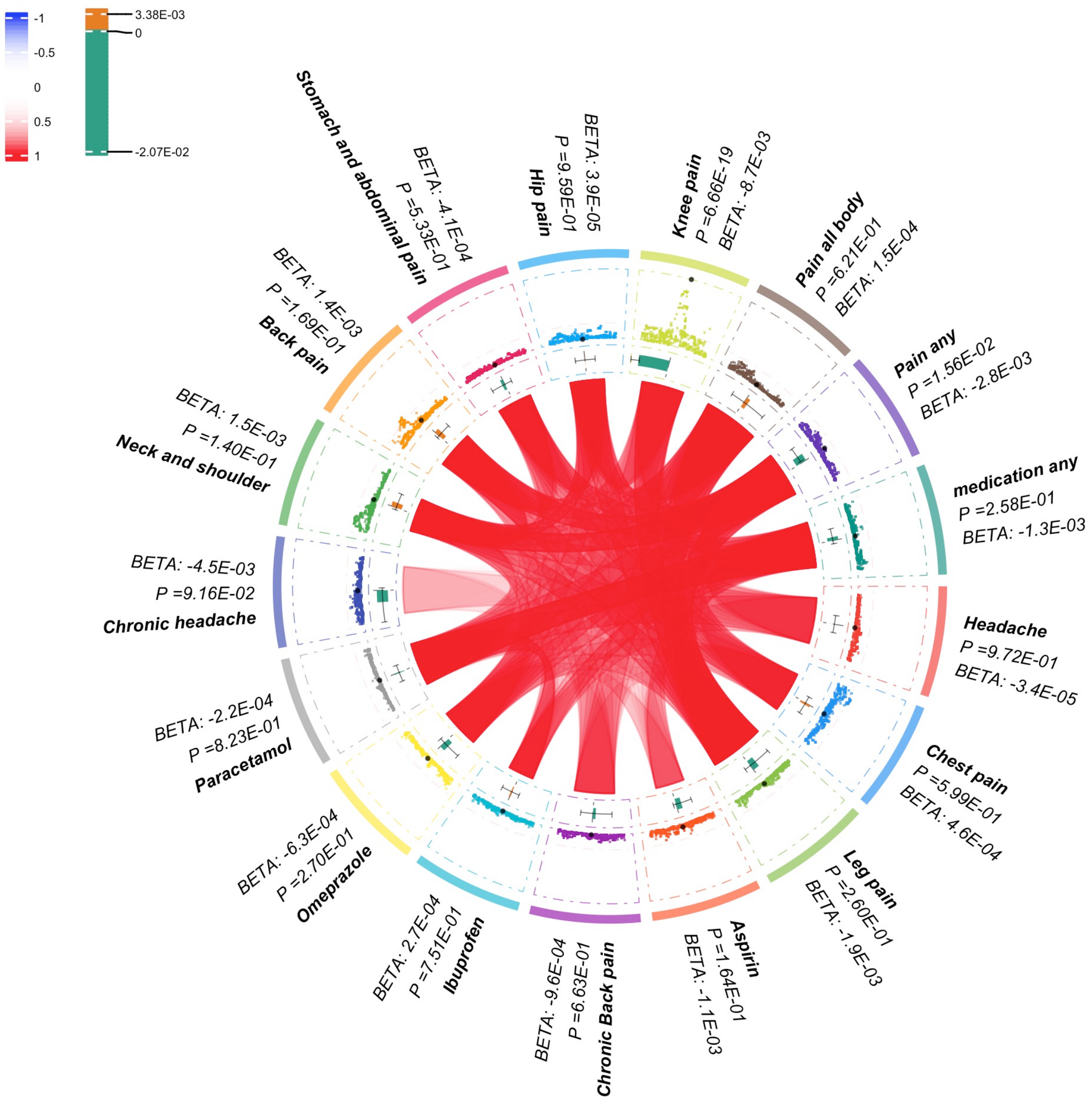

Locus99 22q12.2, rs16988333 Pleio-P= 6.74E-09, intronic to *HORMAD2* gene

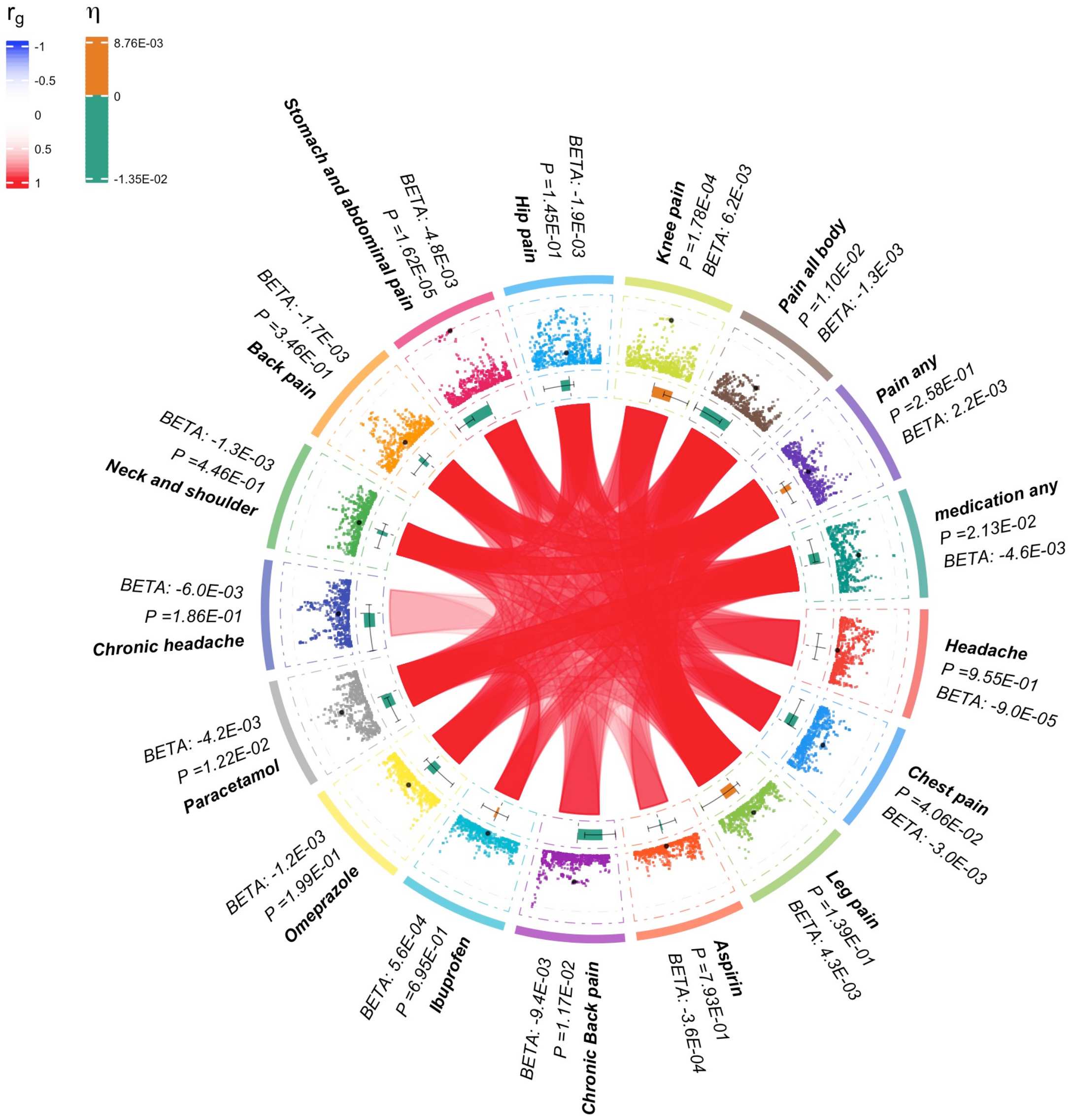

Supplement: S4 Fig — (PDF) [file pgen.1010977.s007.pdf]
