# Supplementary material for: Genome wide association joint analysis reveals 99 risk loci for pain susceptibility and pleiotropic relationships with psychiatric, metabolic, and immunological traits
Source: PLoS Genet. 2023 Oct 16;19(10):e1010977. doi: 10.1371/journal.pgen.1010977 (PMC10602383; doi:10.1371/journal.pgen.1010977)

S5_Figure. Zoom plot of the 99 genome-wide significant loci, including gene-mapping information.

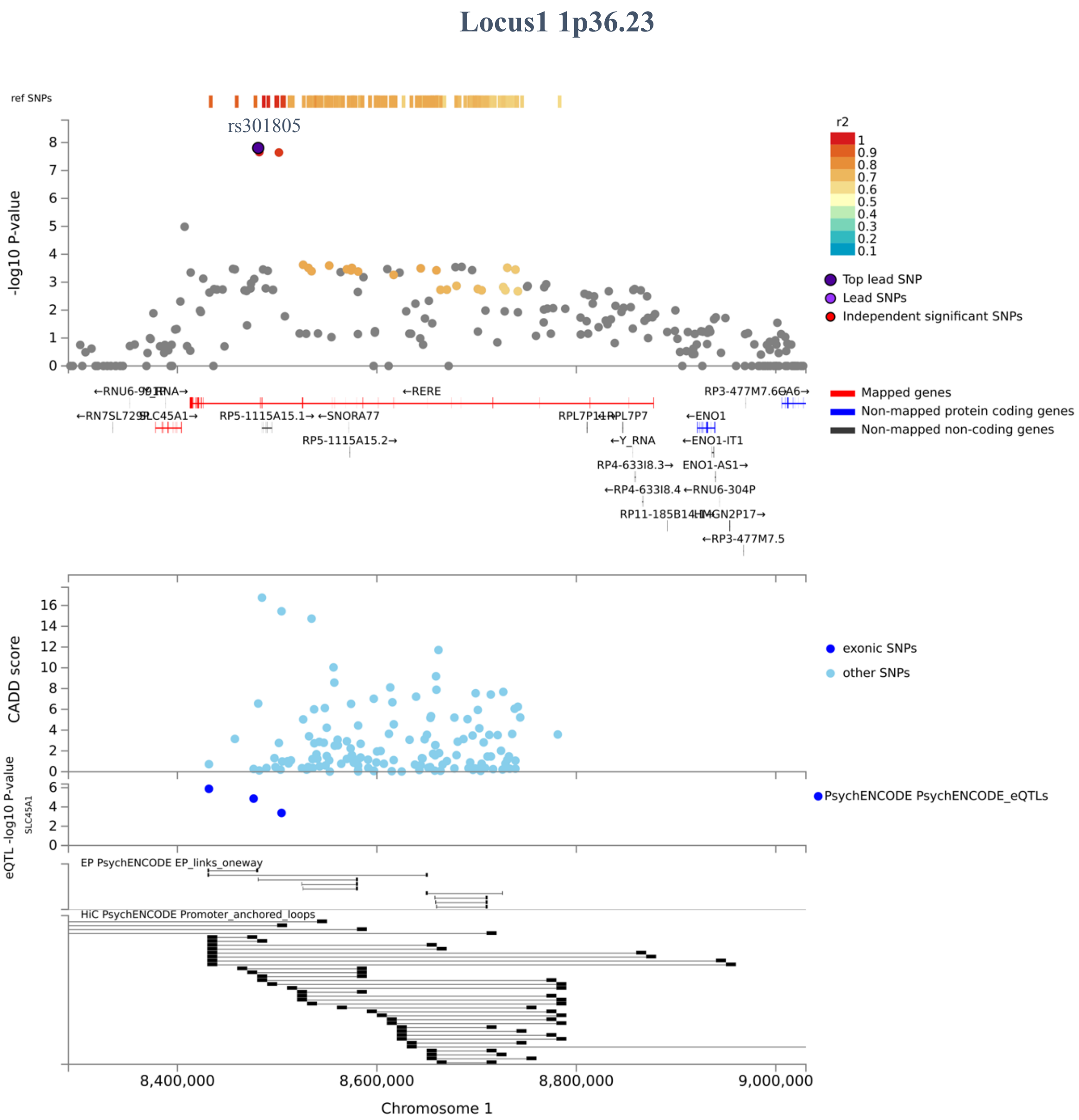

Locus2 1p36.21

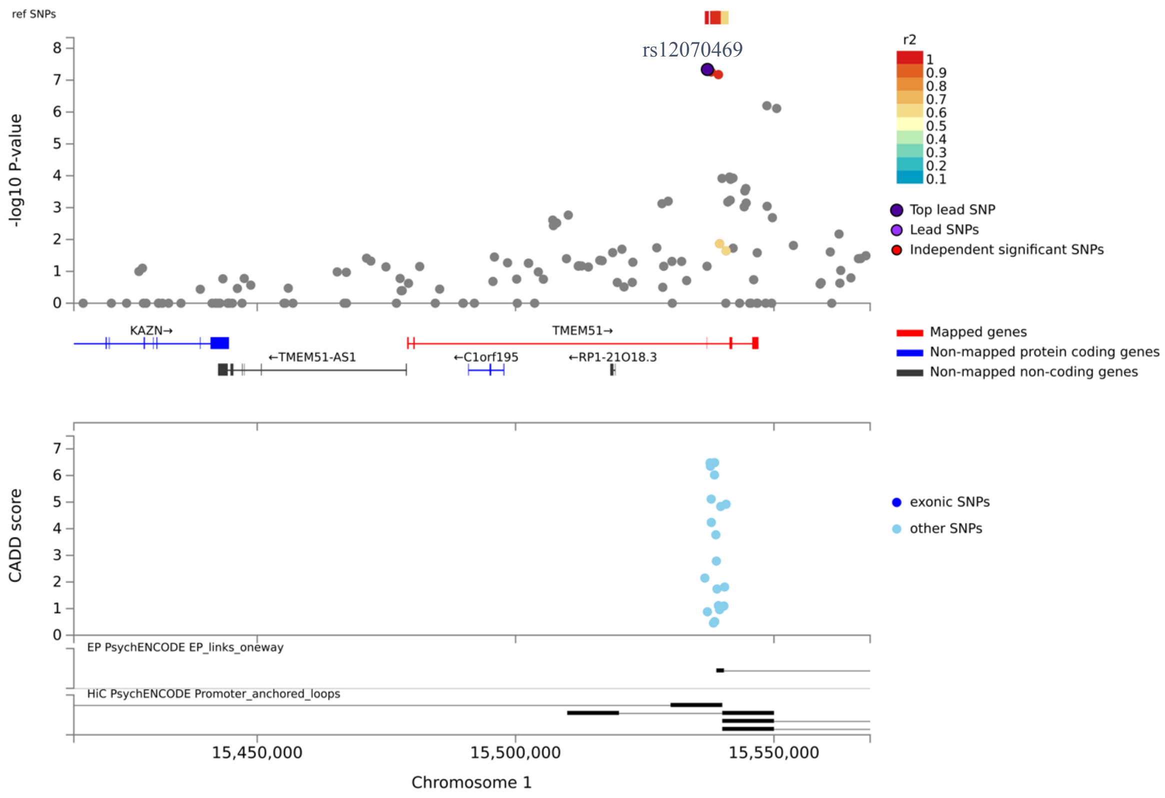

No eQTL of selected tissues exists in this region.

Locus3 1p34.3

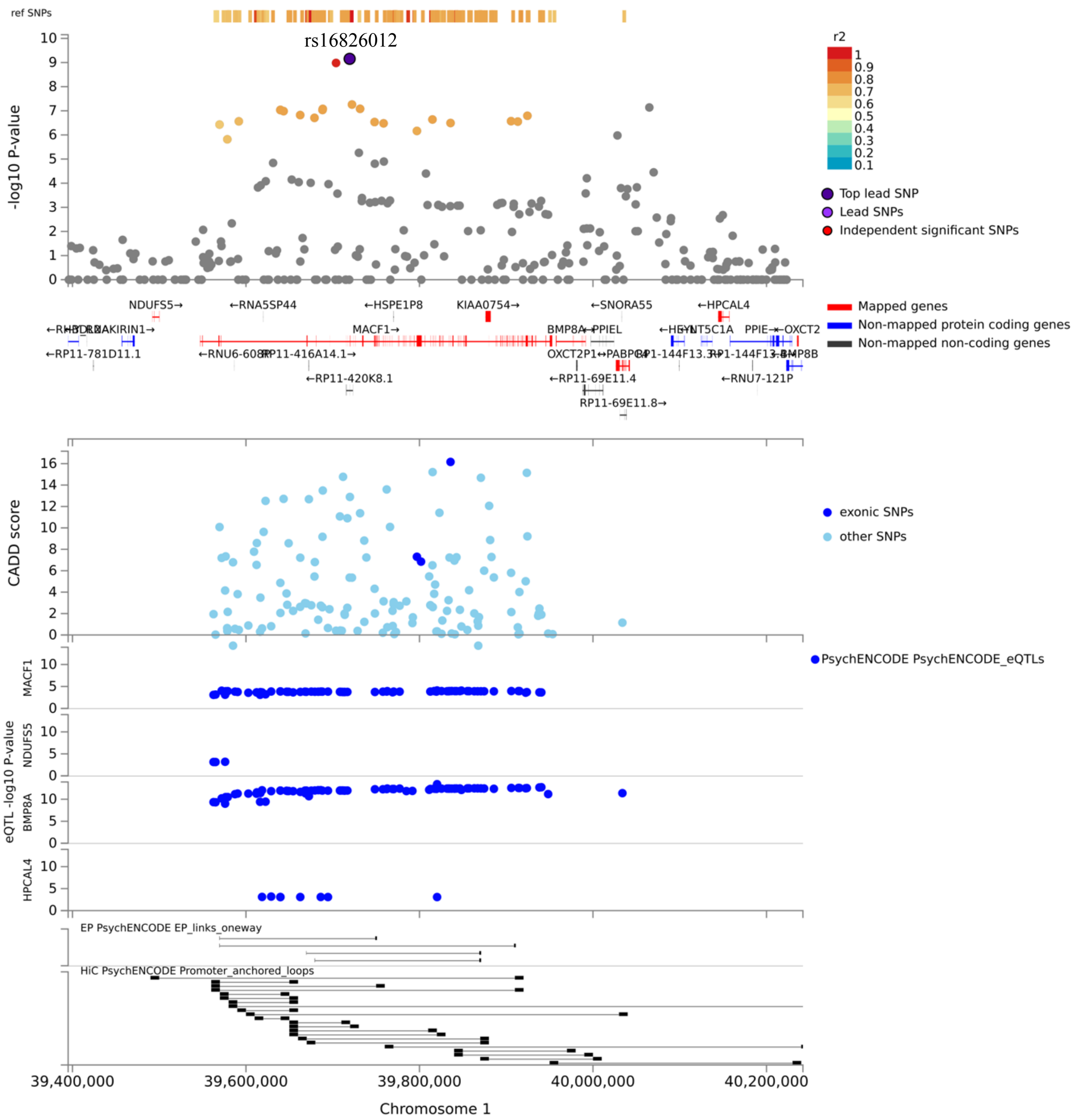

Locus4 1p32.3

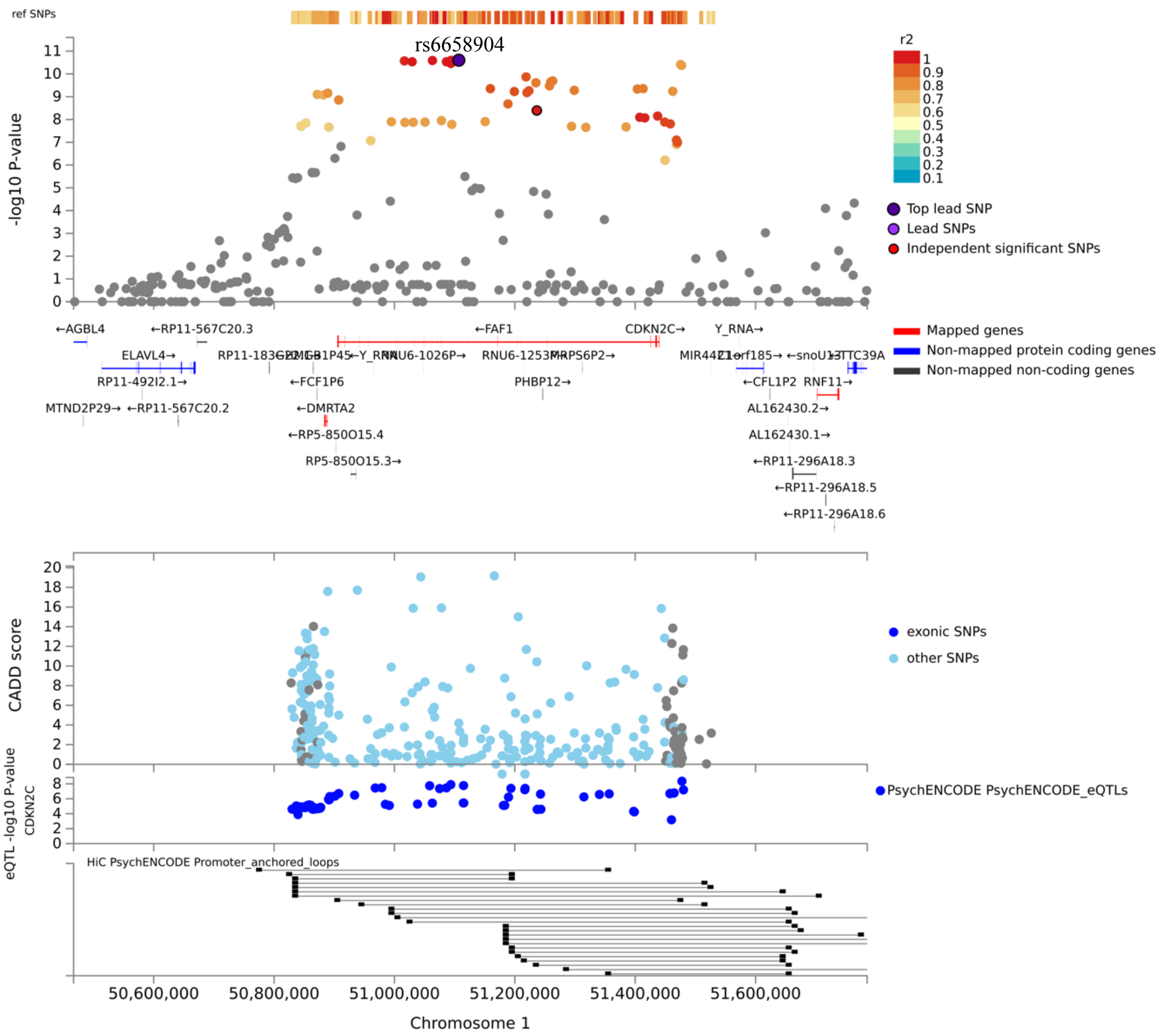

Locus5 1p31.3

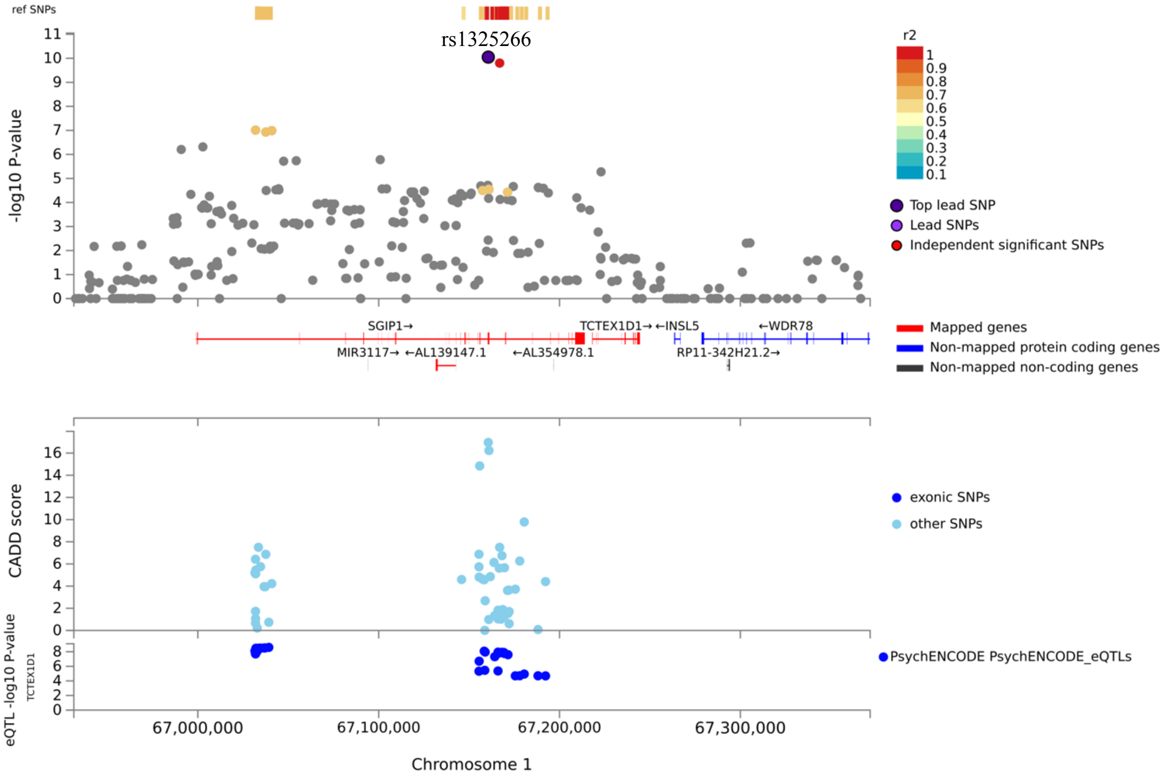

Locus6 1p22.1

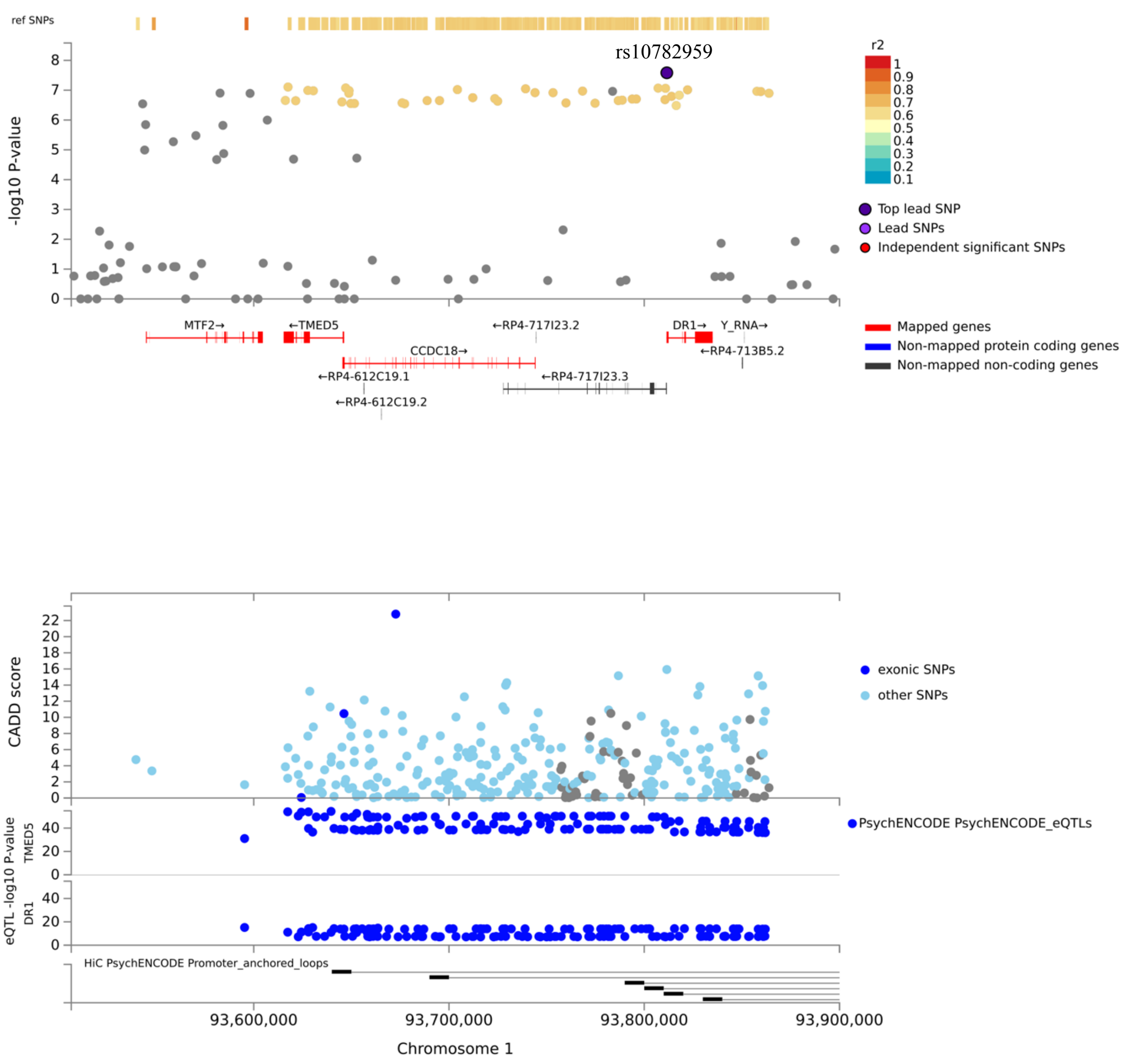

Locus7 1p21.3

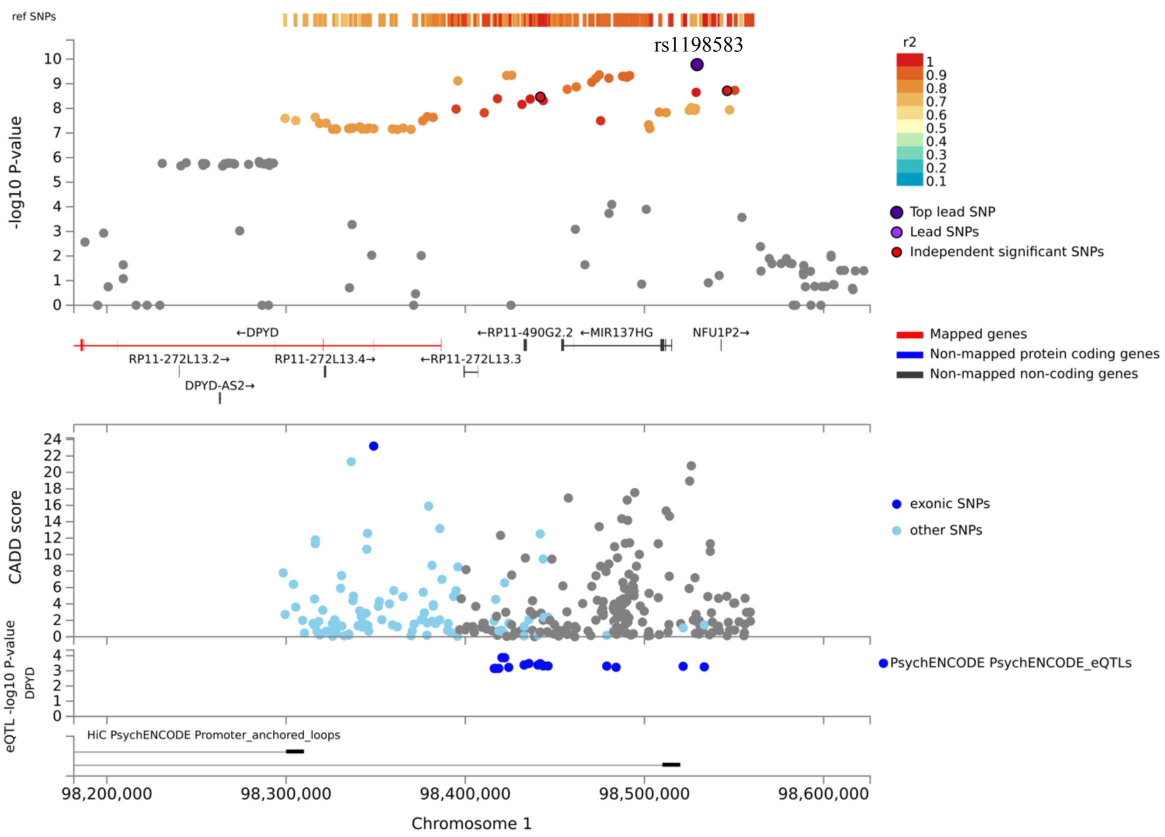

Locus8 1p13.3

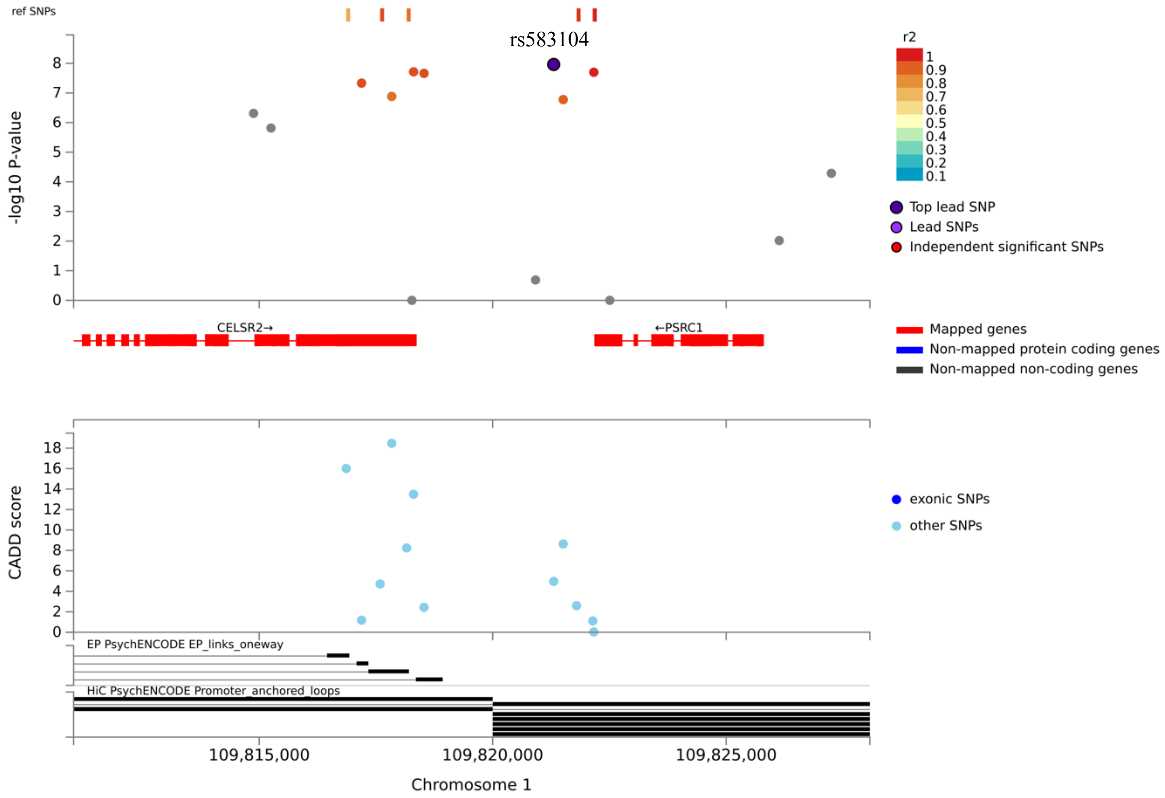

No eQTL of selected tissues exists in this region.

Locus9 1p13.2

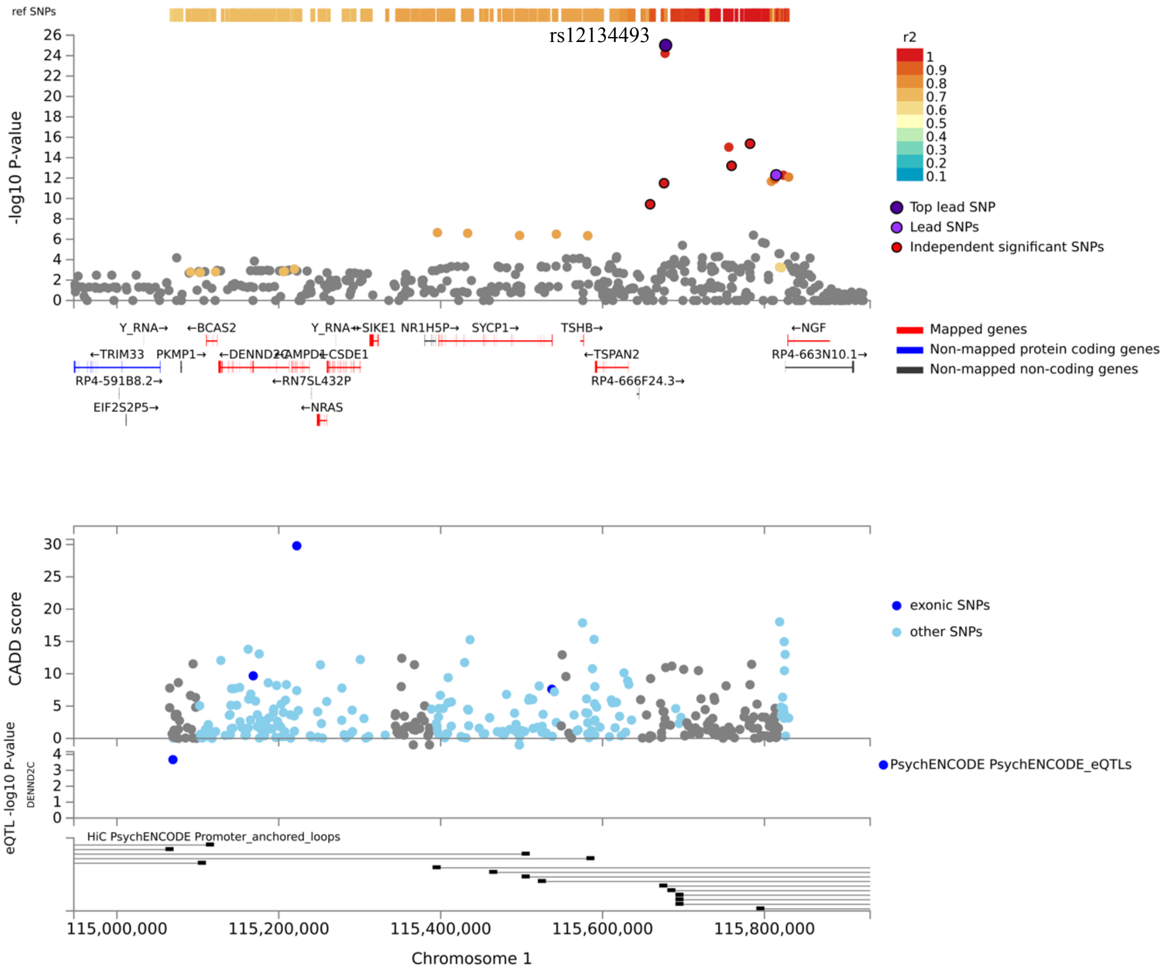

Locus10 1q21.3

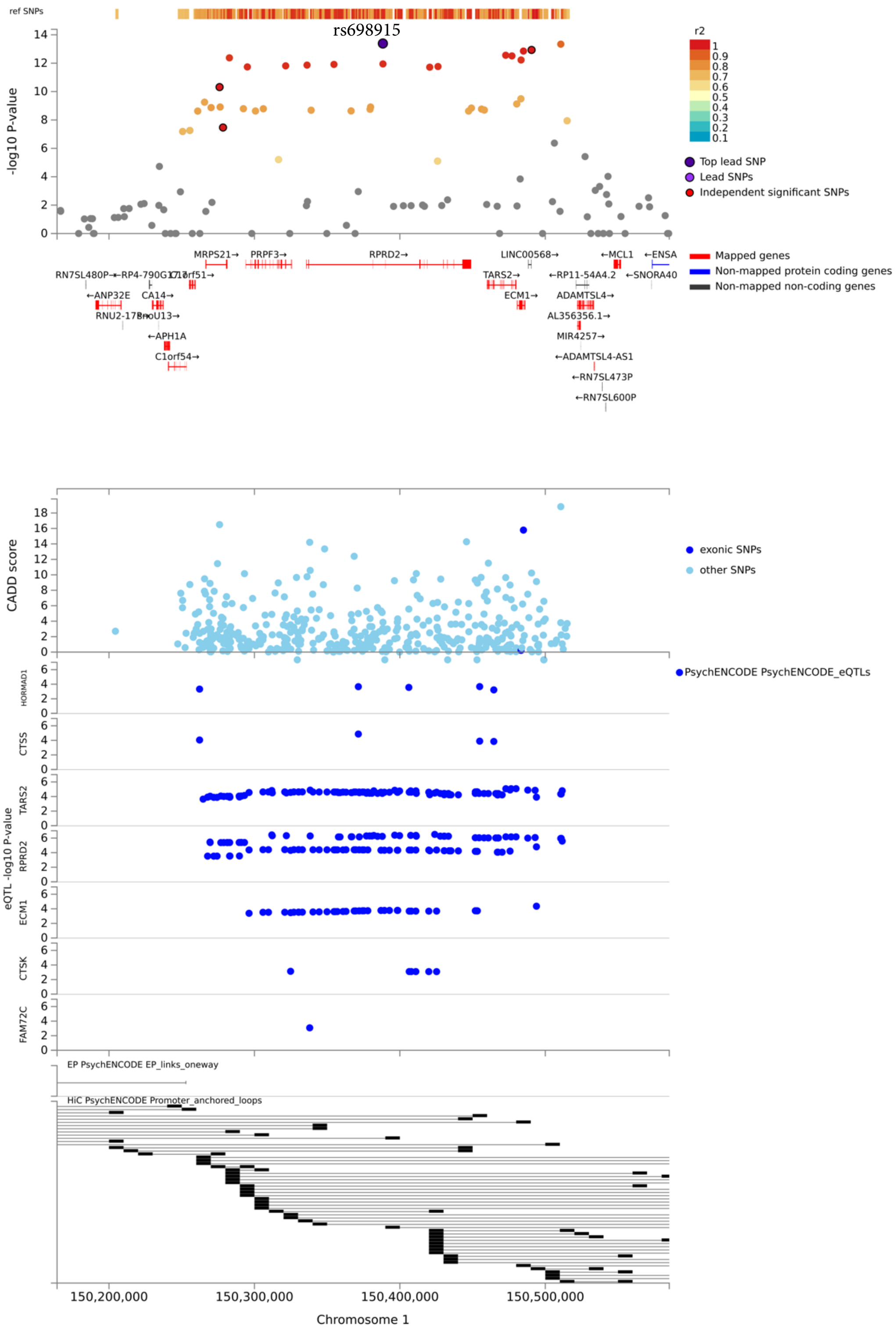

Locus11 1q21.3

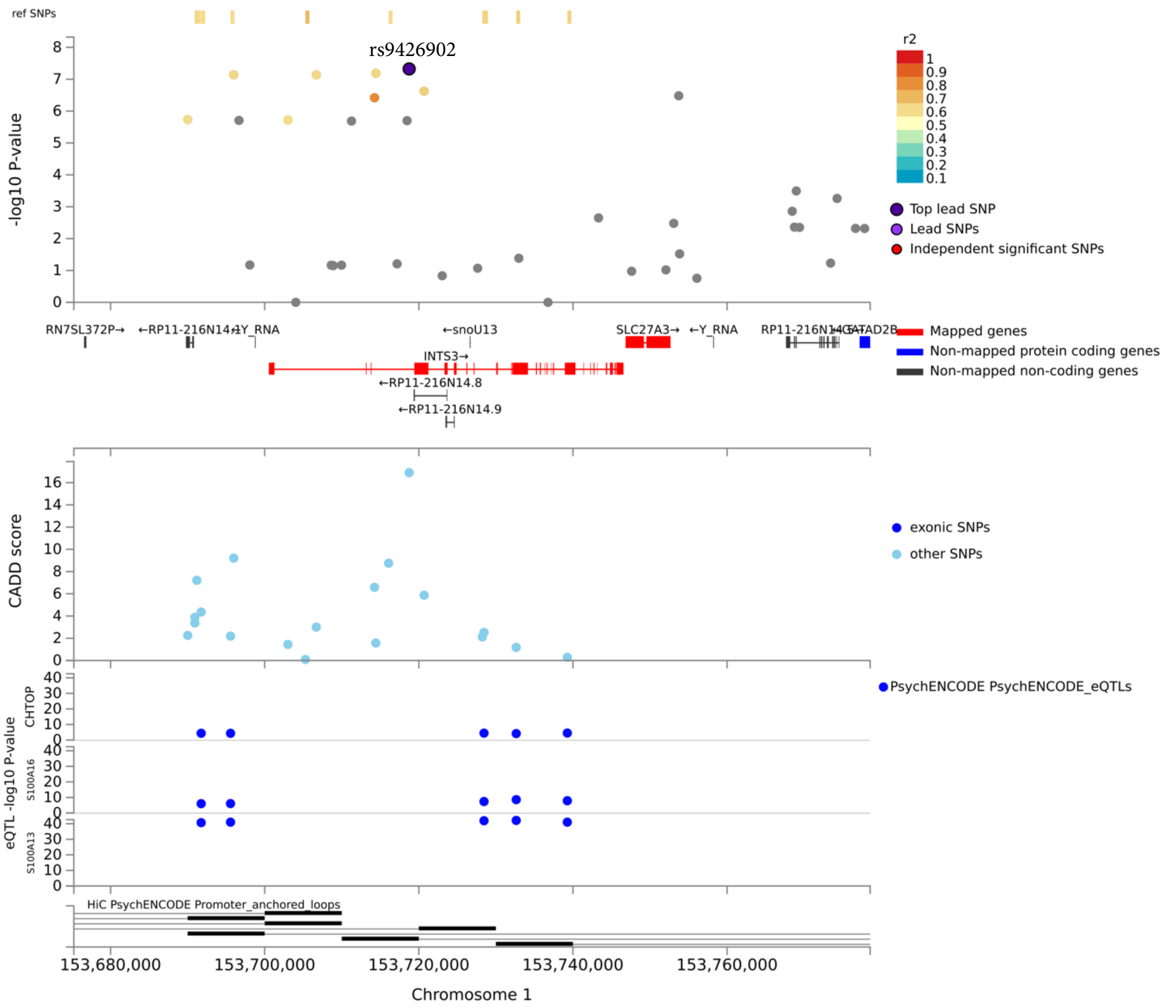

Locus12 1q22

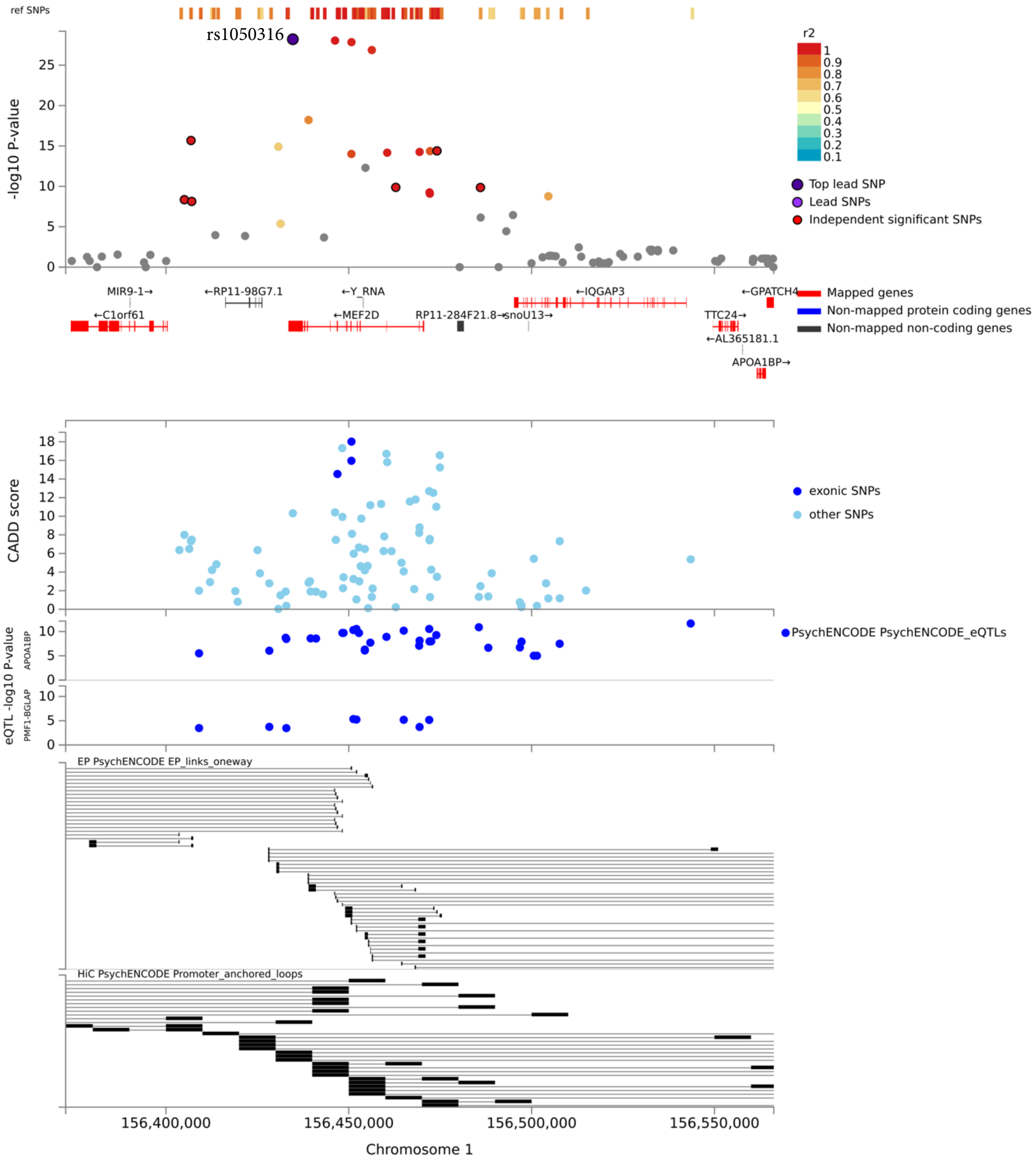

Locus13 1q25.1

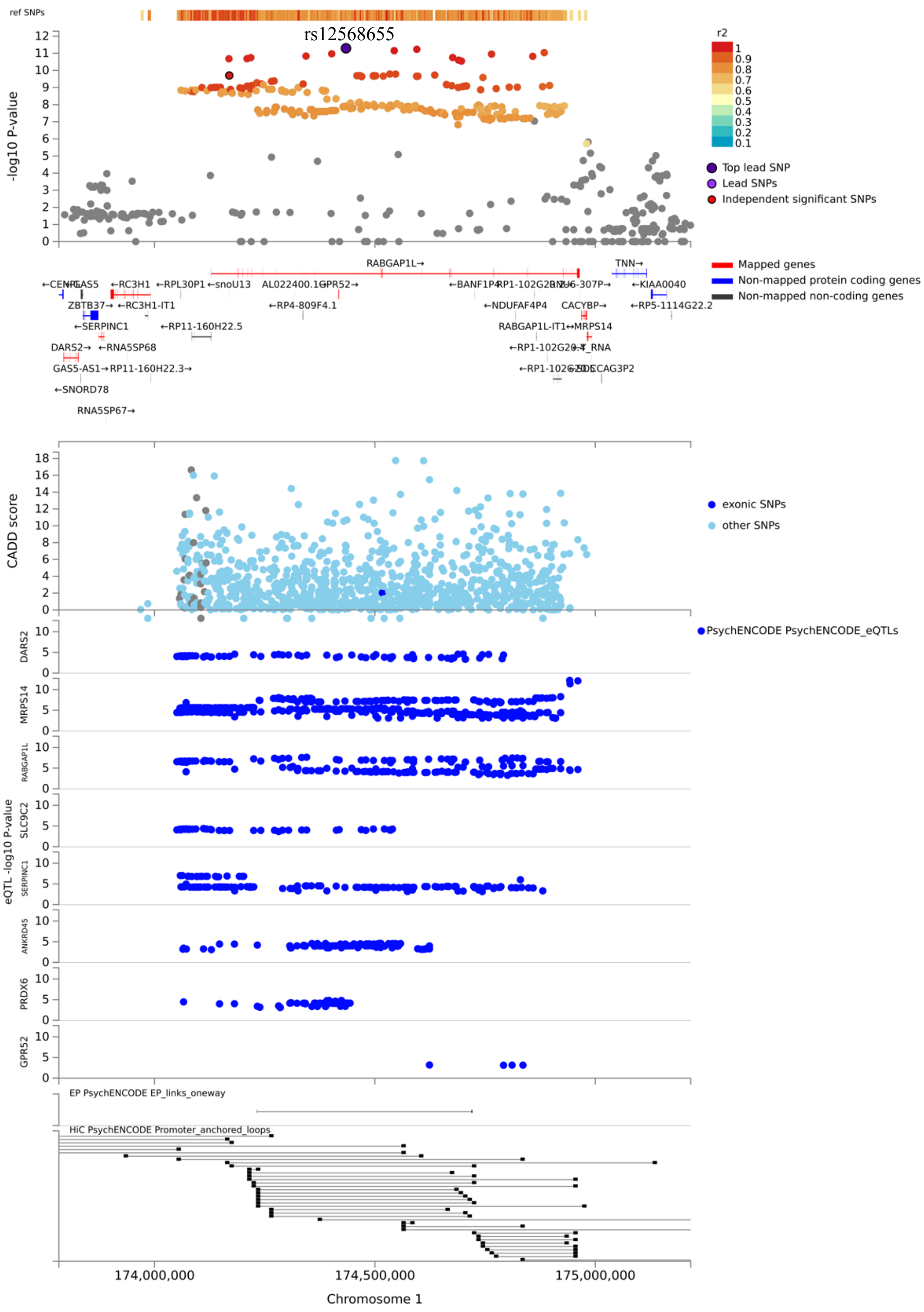

Locus14 1q43

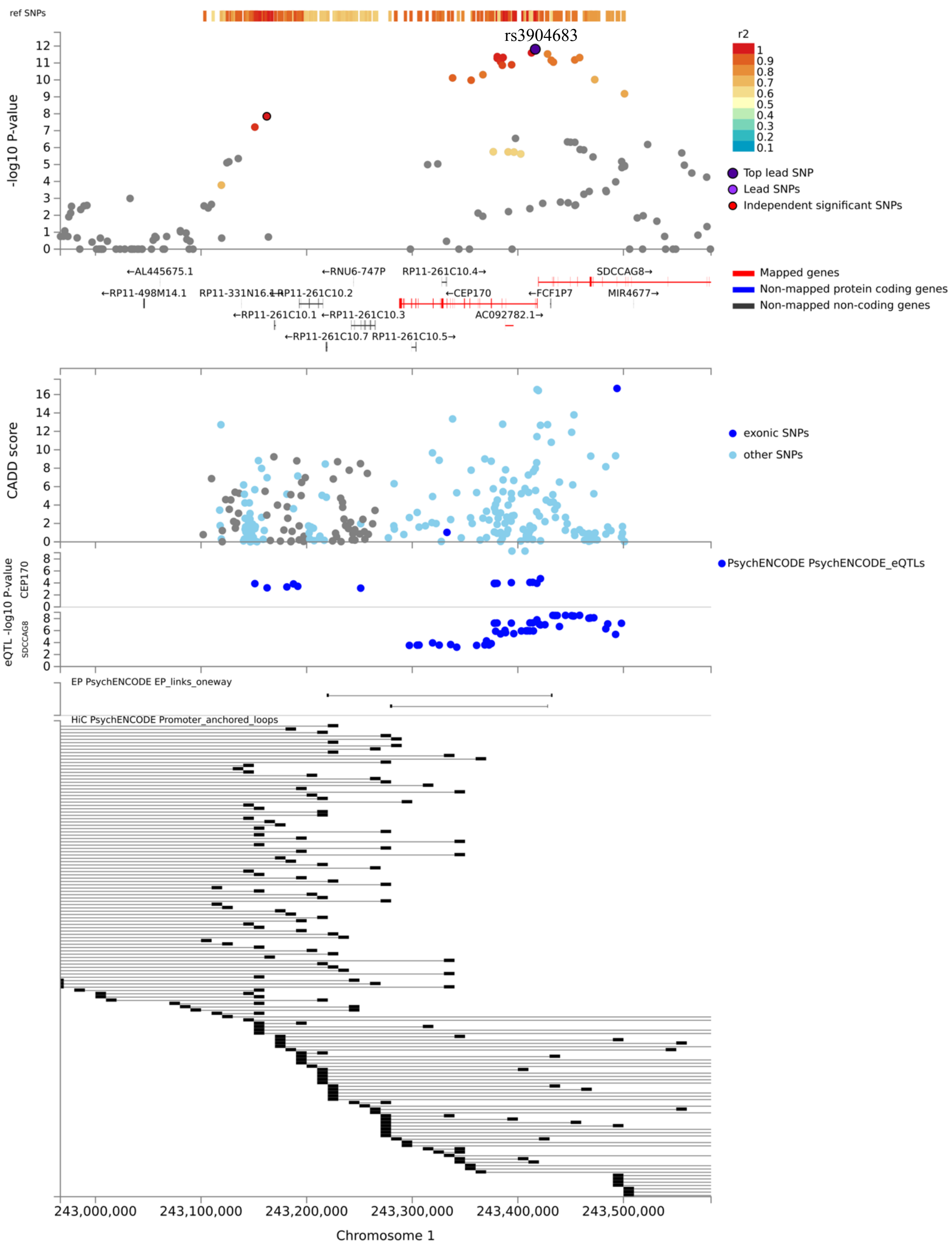

Locus15 2p24.1

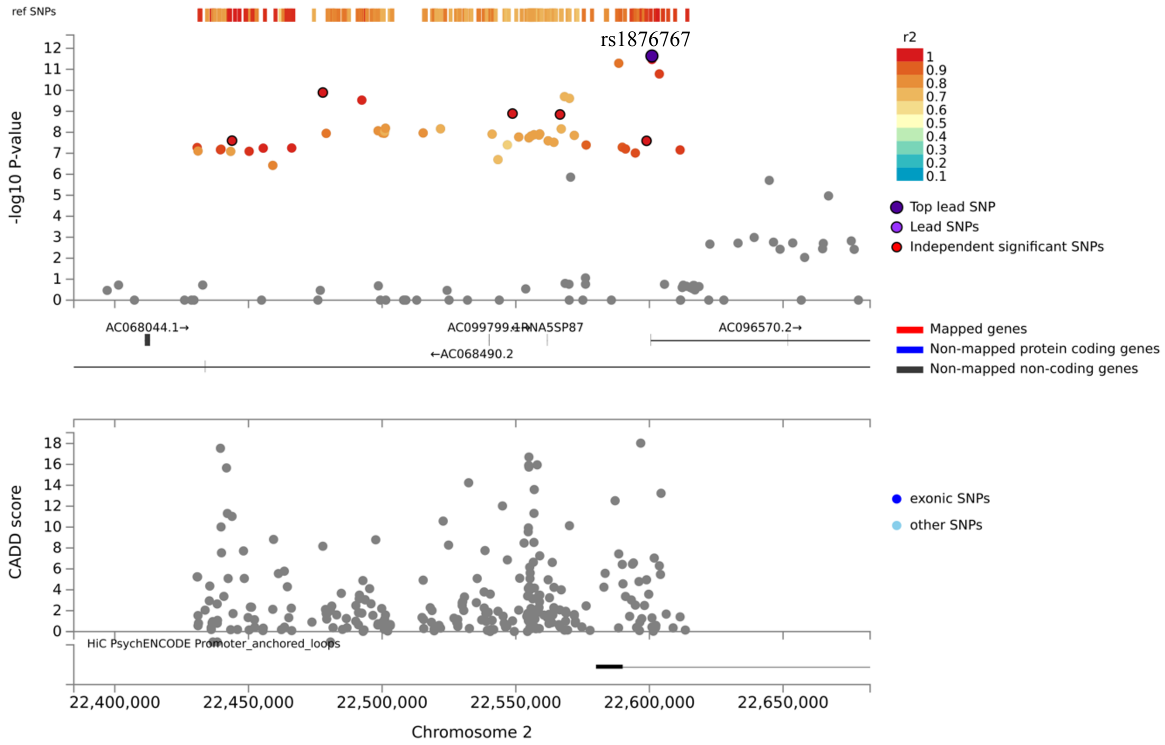

No eQTL of selected tissues exists in this region.

Locus16 2p23.3

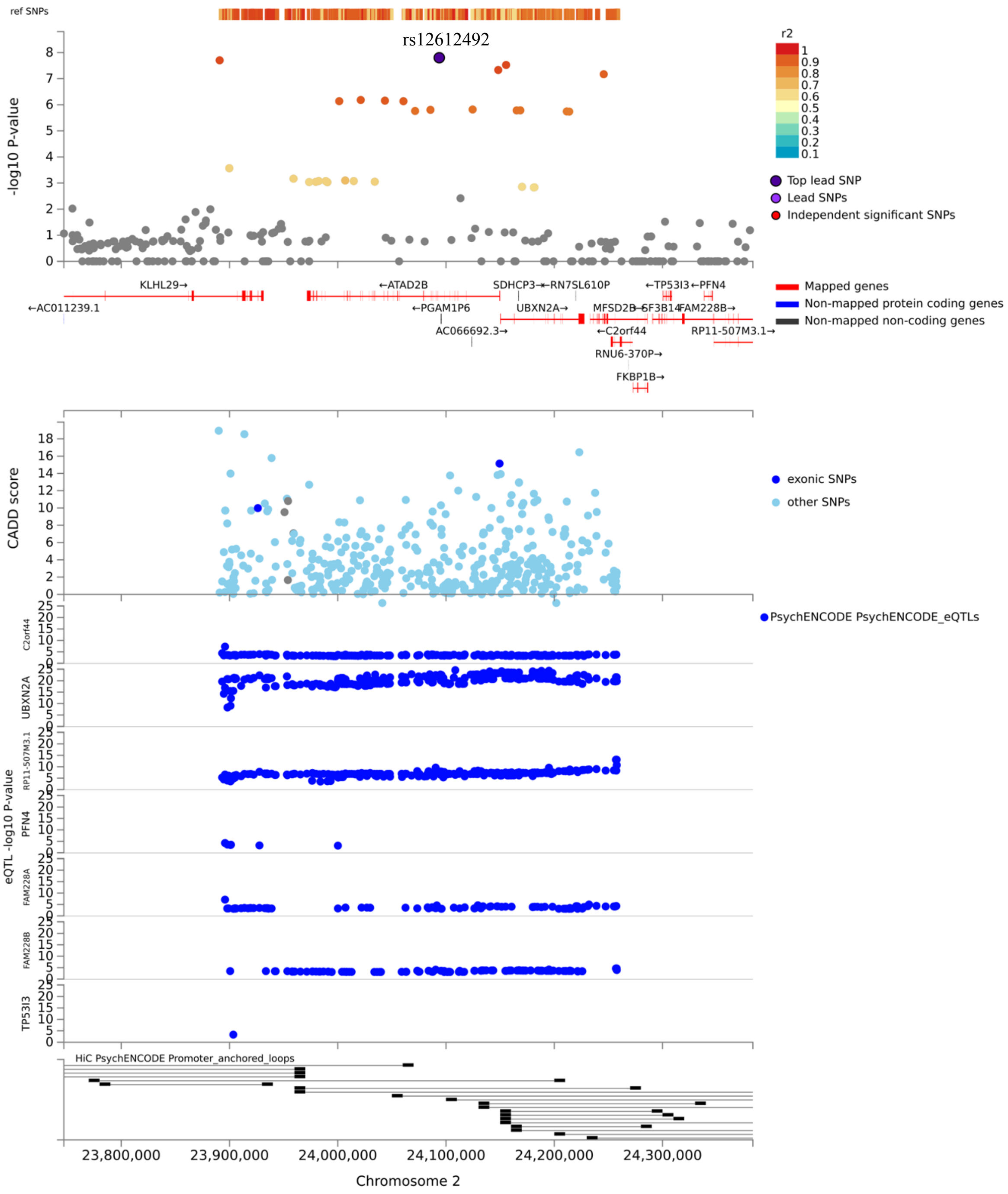

Locus17 2p16.3

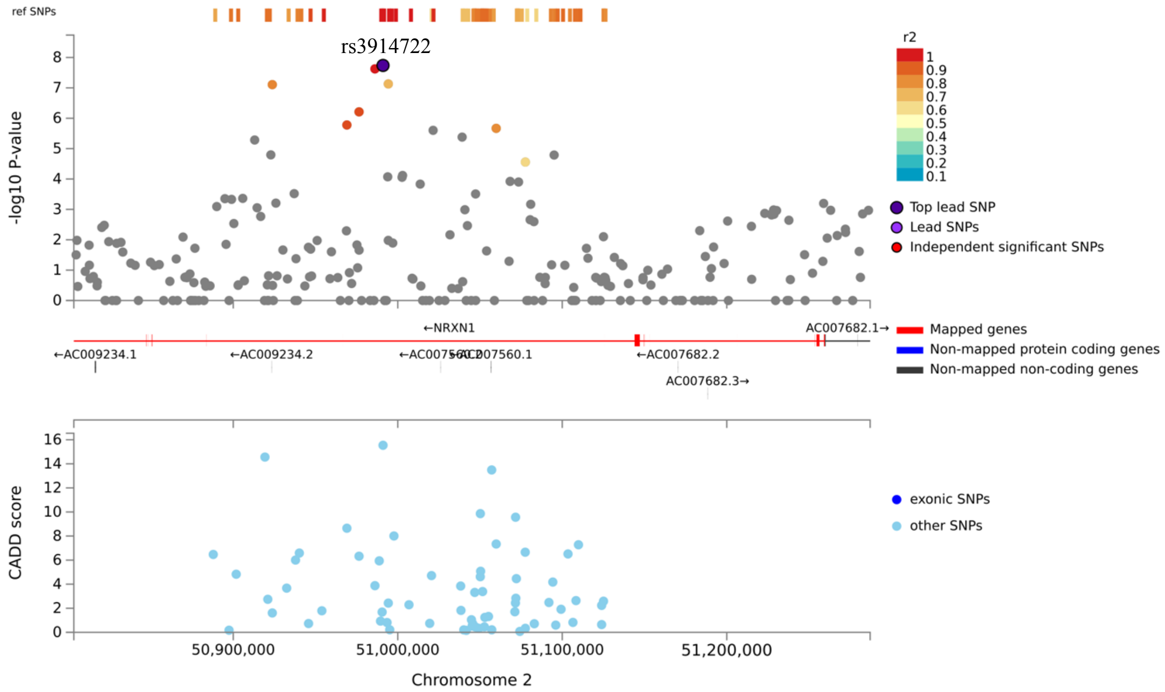

No eQTL of selected tissues exists in this region.

Locus18 2q11.2

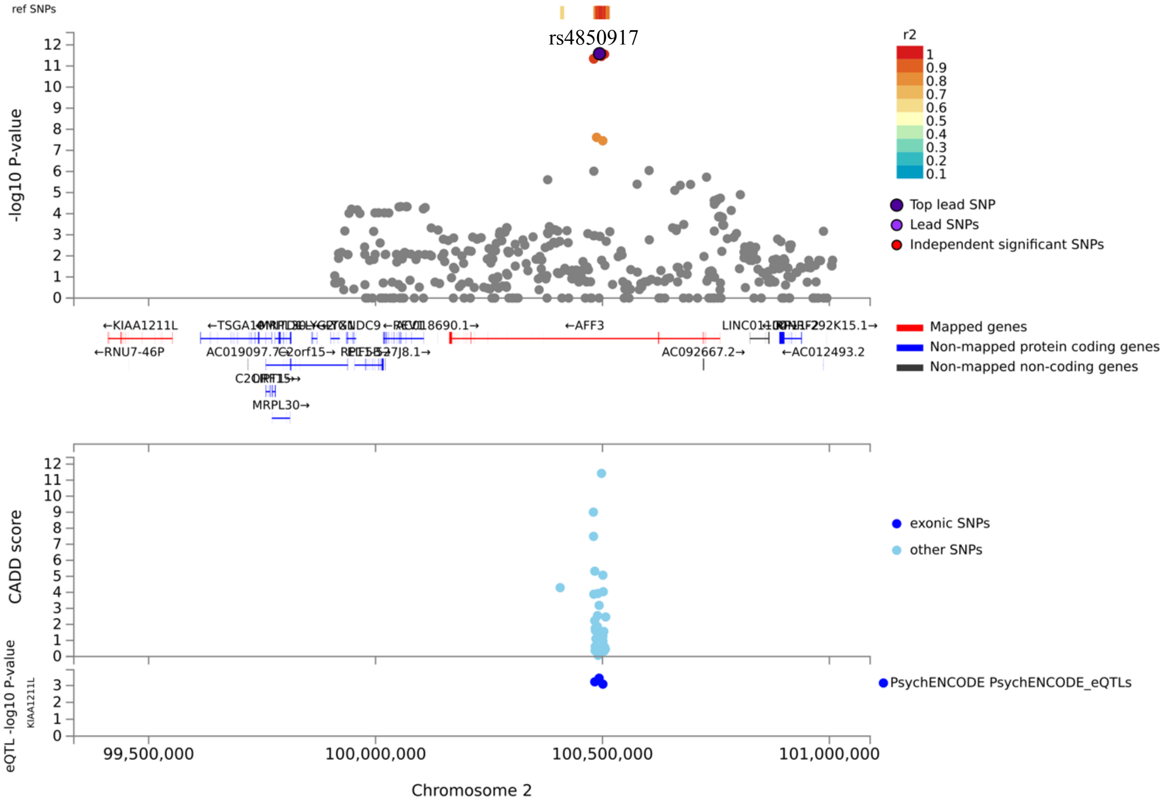

Locus19 2q12.1

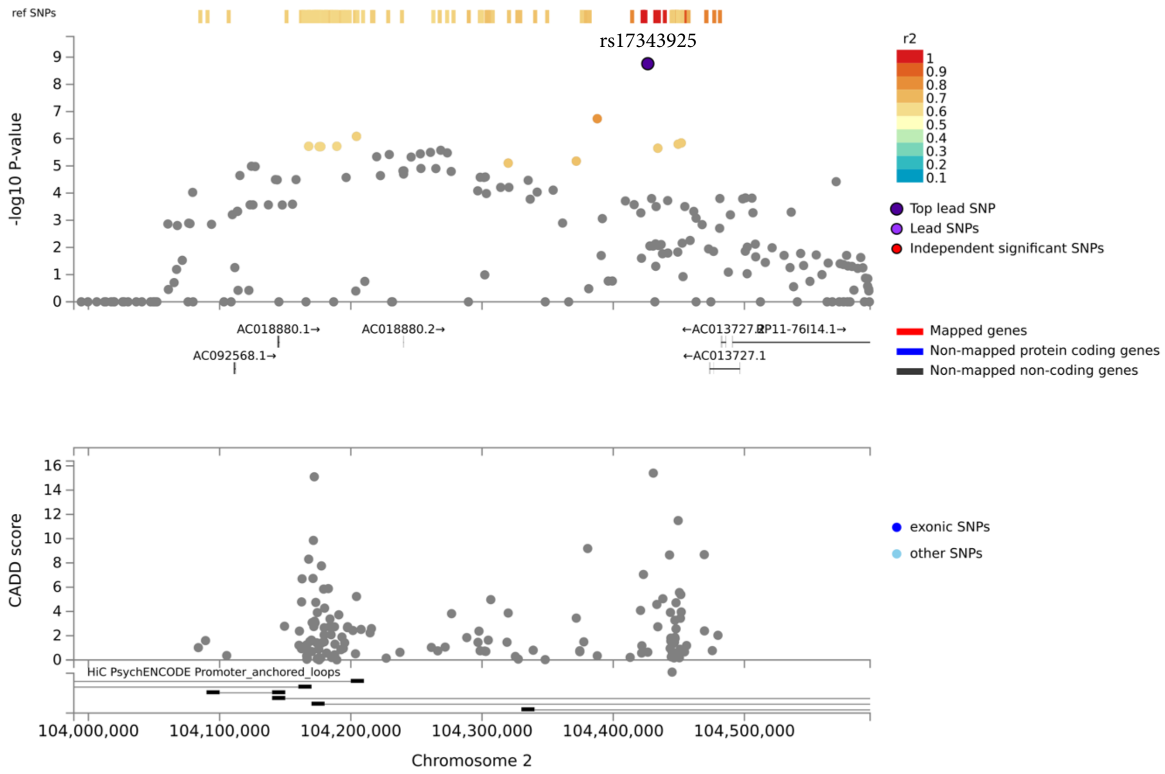

No eQTL of selected tissues exists in this region.

Locus20 2q33.3

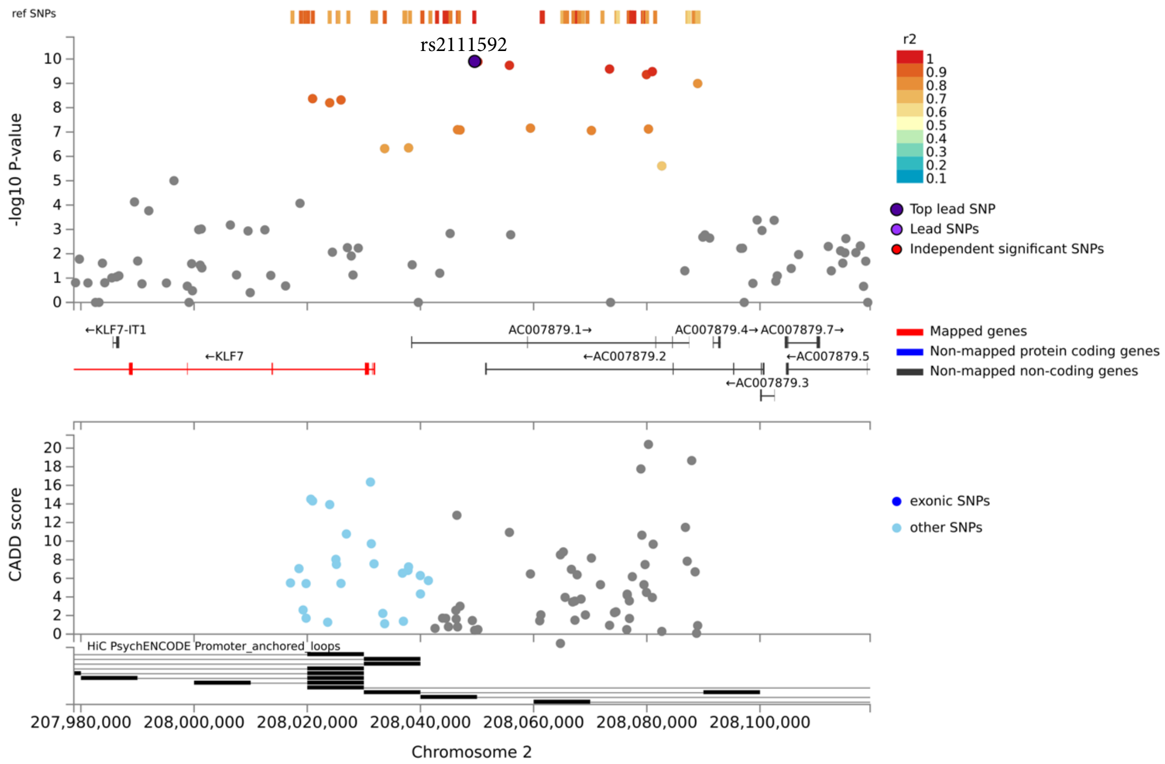

No eQTL of selected tissues exists in this region.

Locus21 2q34

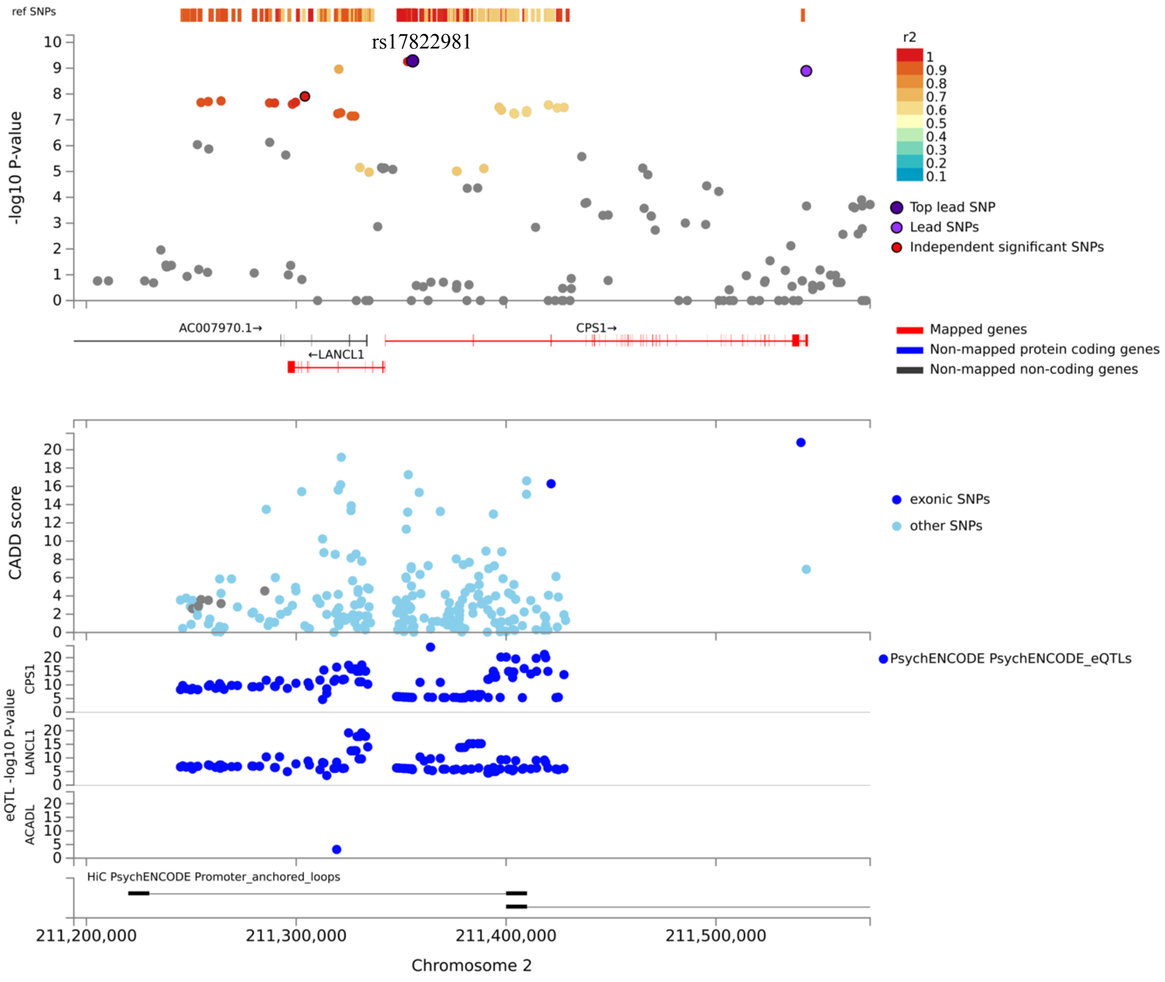

Locus22 2q37.1

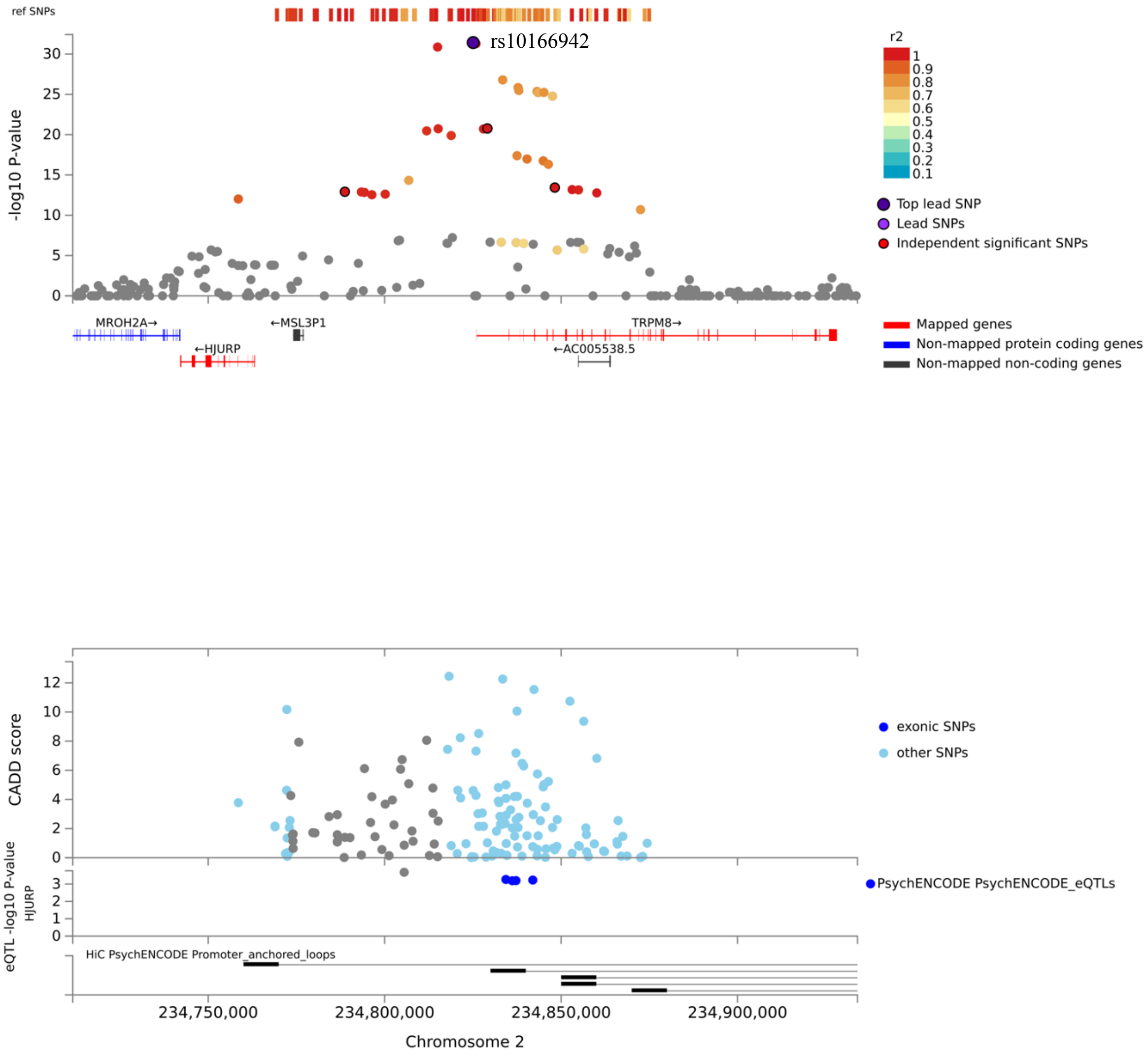

Locus23 3p24.1

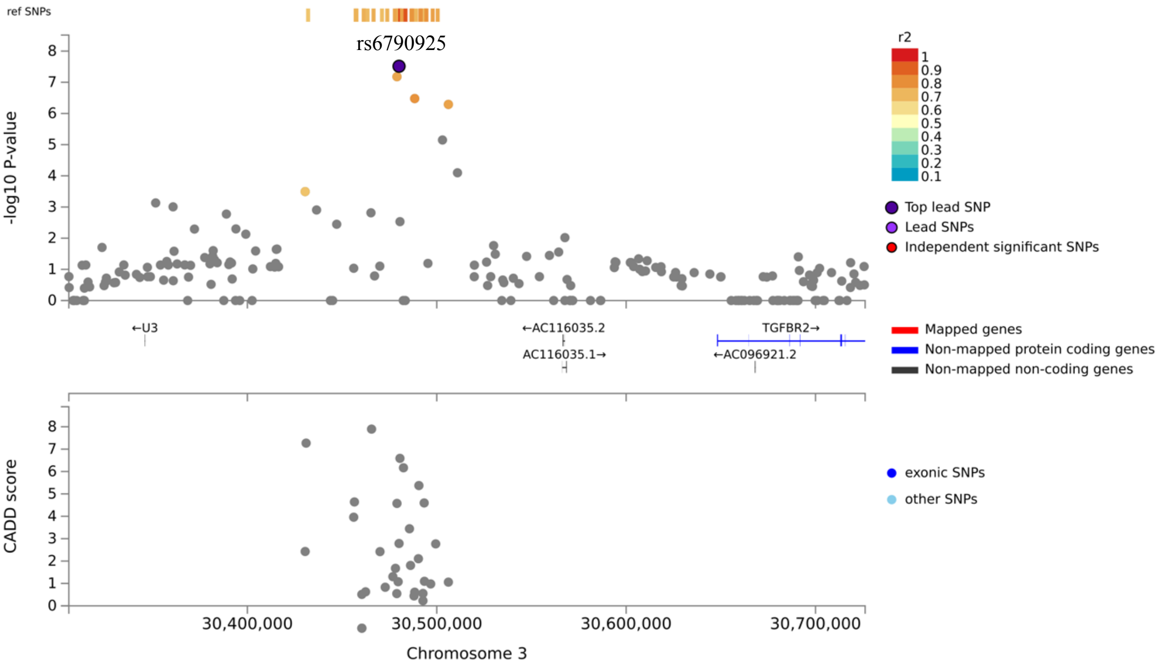

No eQTL of selected tissues exists in this region.

Locus24 3p21.31

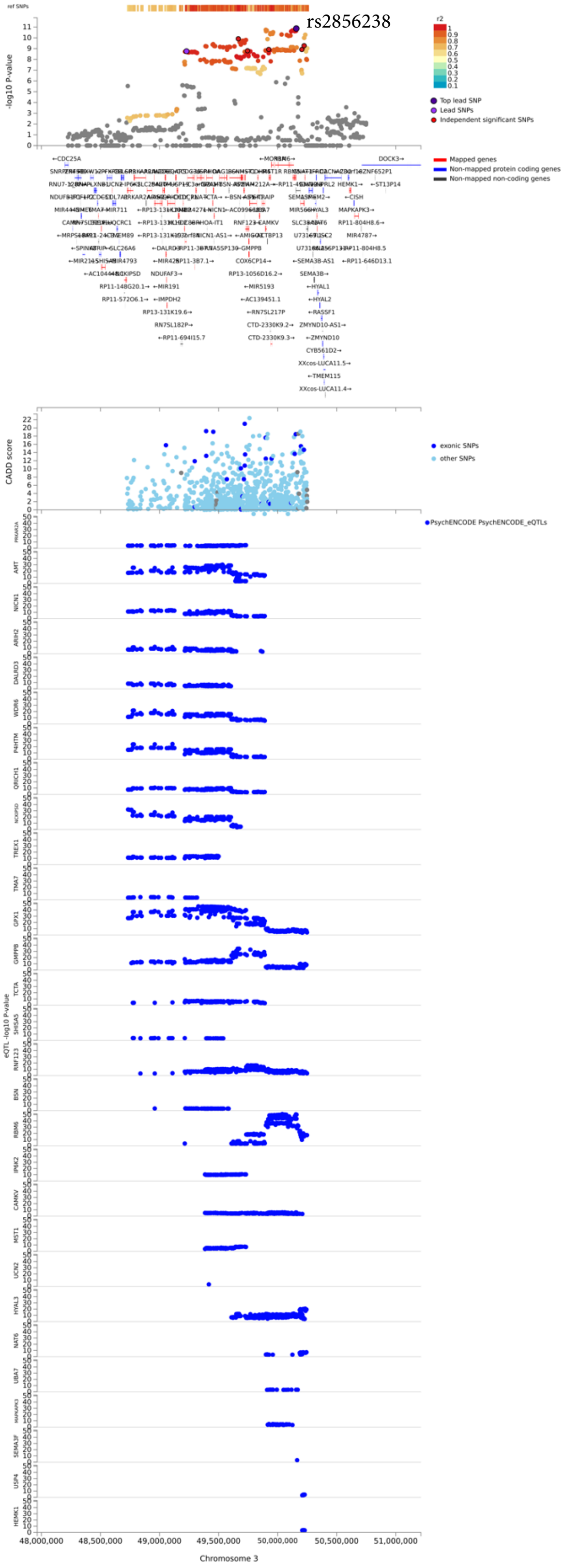

Locus25 3p12.3

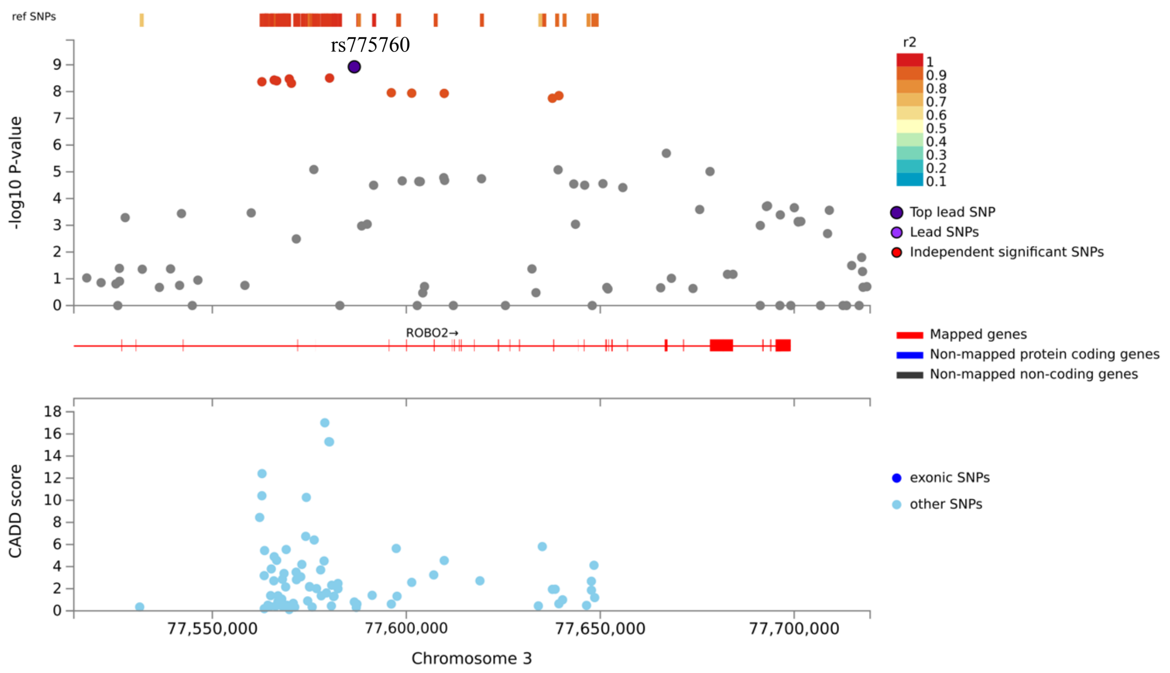

No eQTL of selected tissues exists in this region.

Locus26 3q26.31

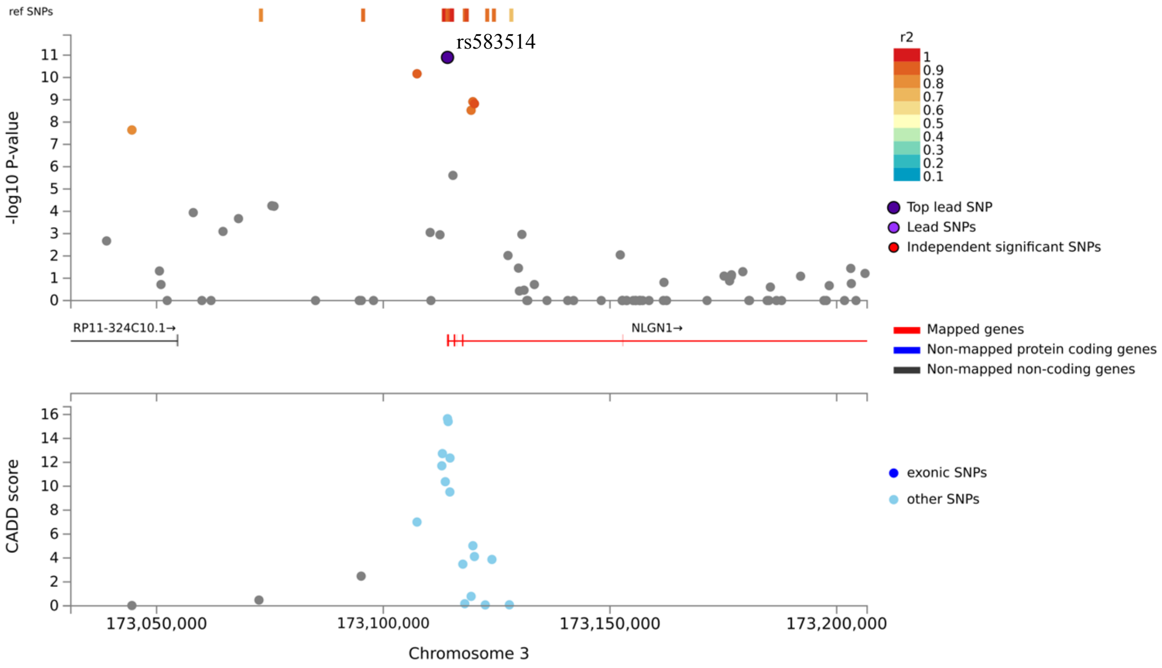

No eQTL of selected tissues exists in this region.

Locus27 4p16.3

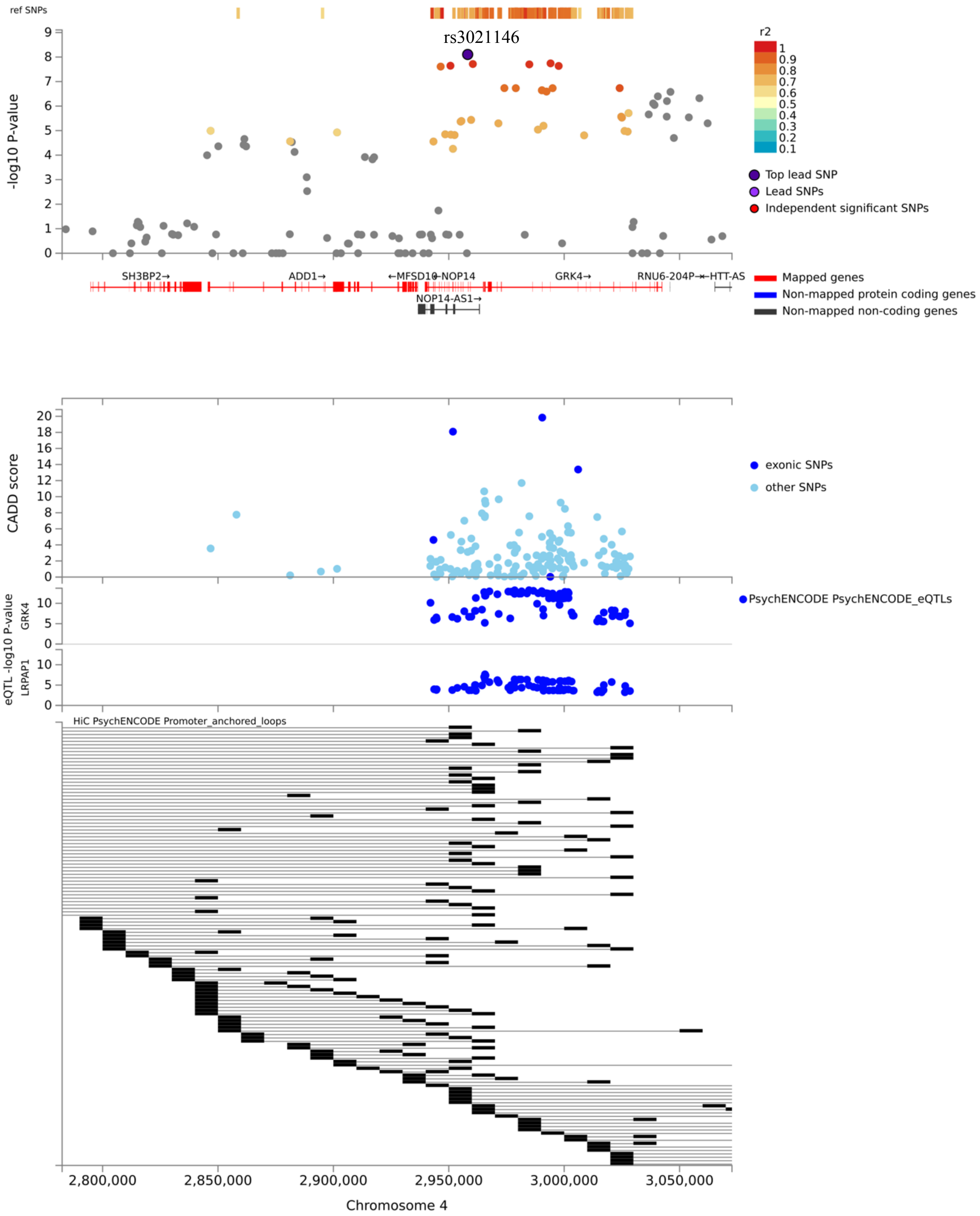

Locus28 4q12

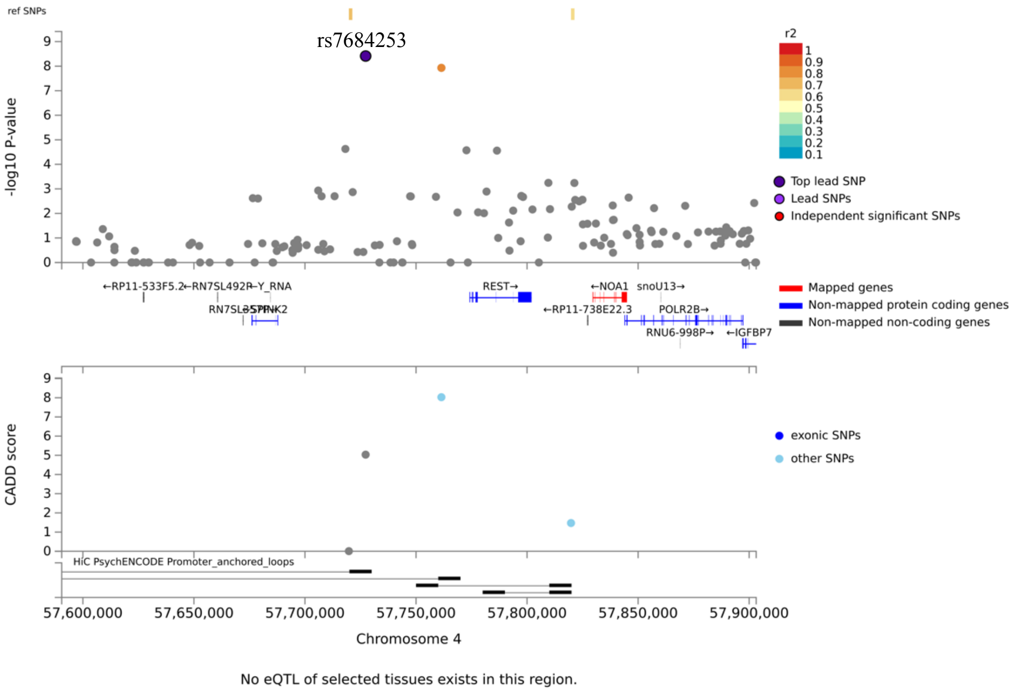

Locus29 4q13.2

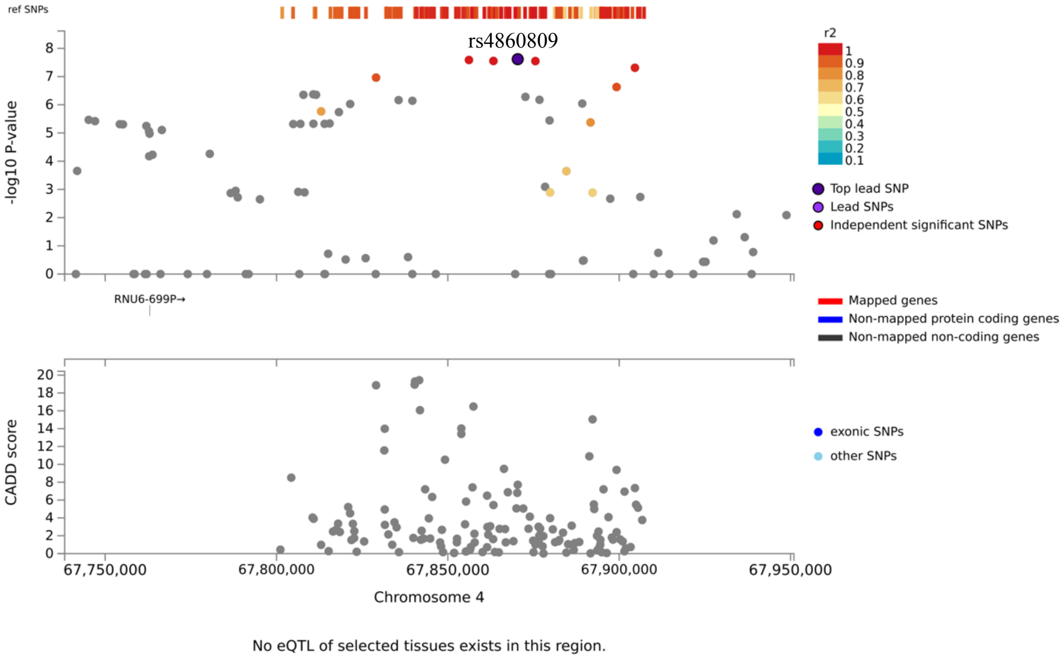

Locus30 4q24

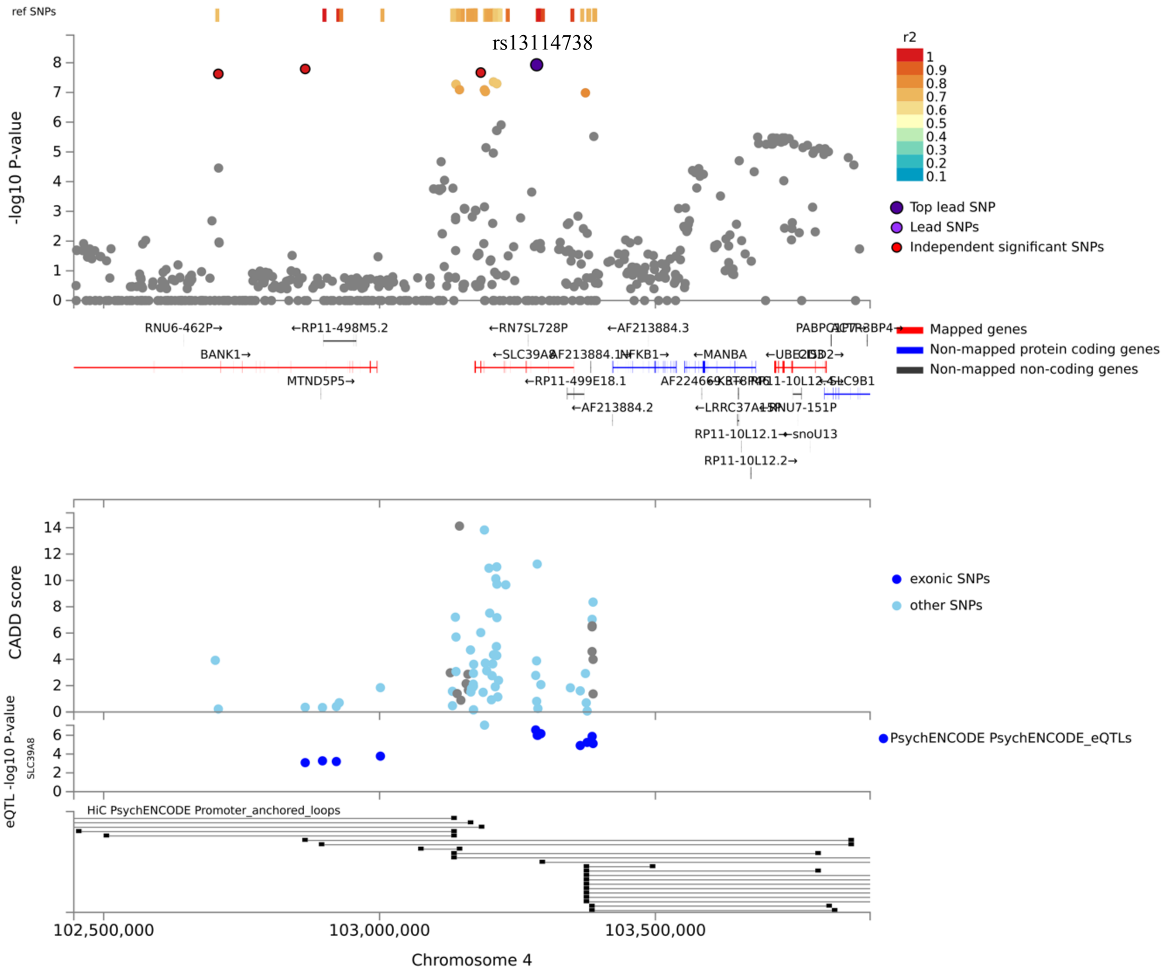

Locus31 4q25

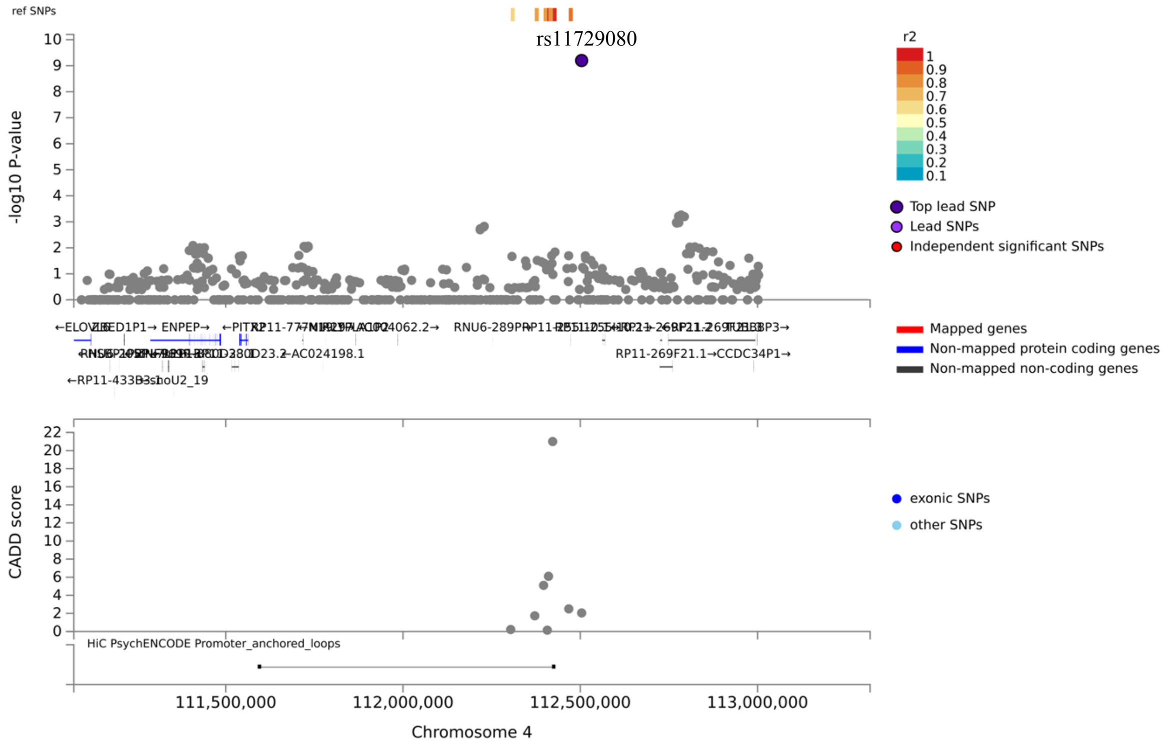

No eQTL of selected tissues exists in this region.

Locus32 5p13.3

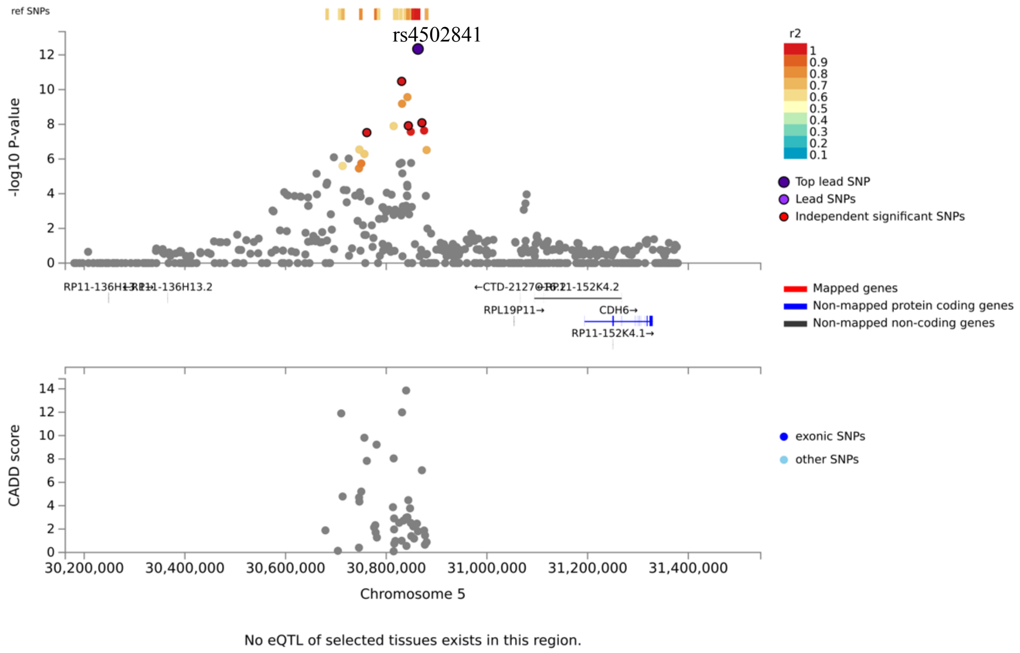

Locus33 5q22.3

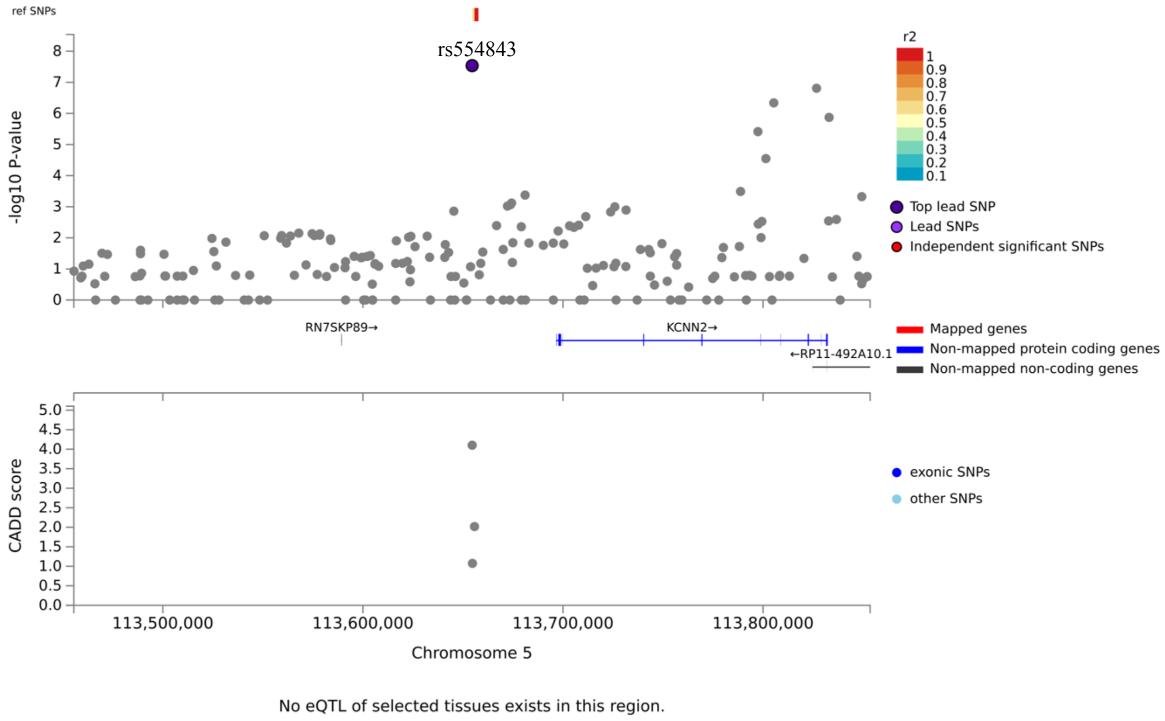

Locus34 5q23.1

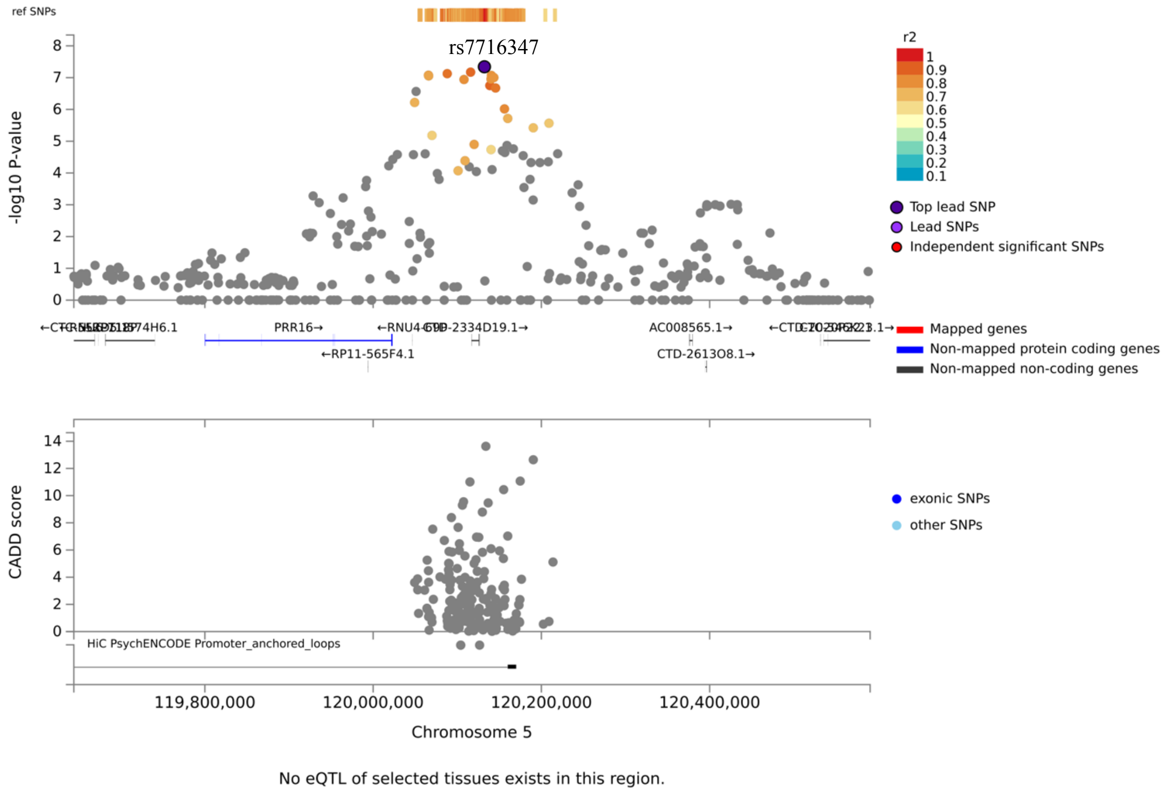

Locus35 5q35.3

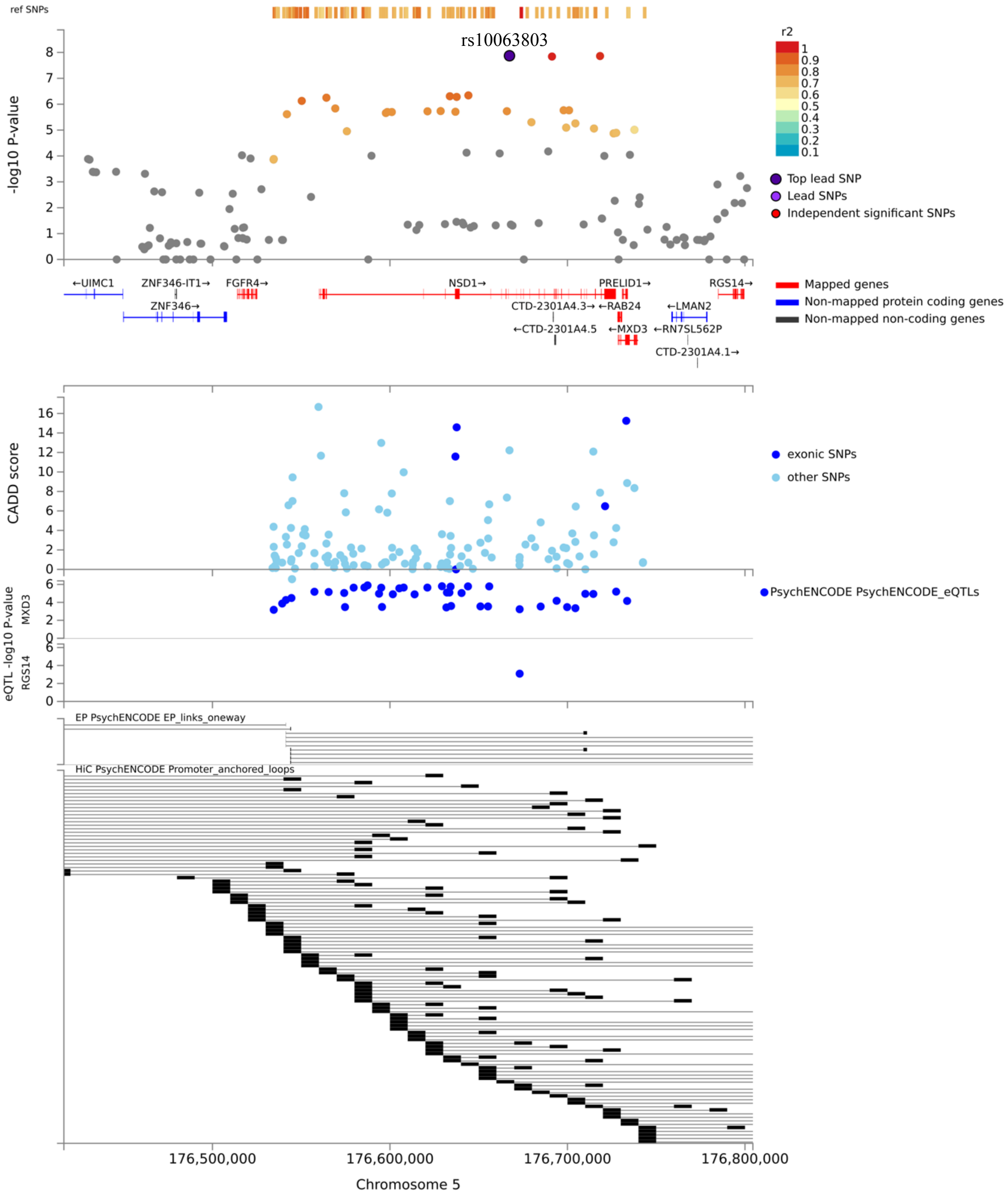

Locus36 6p24.1

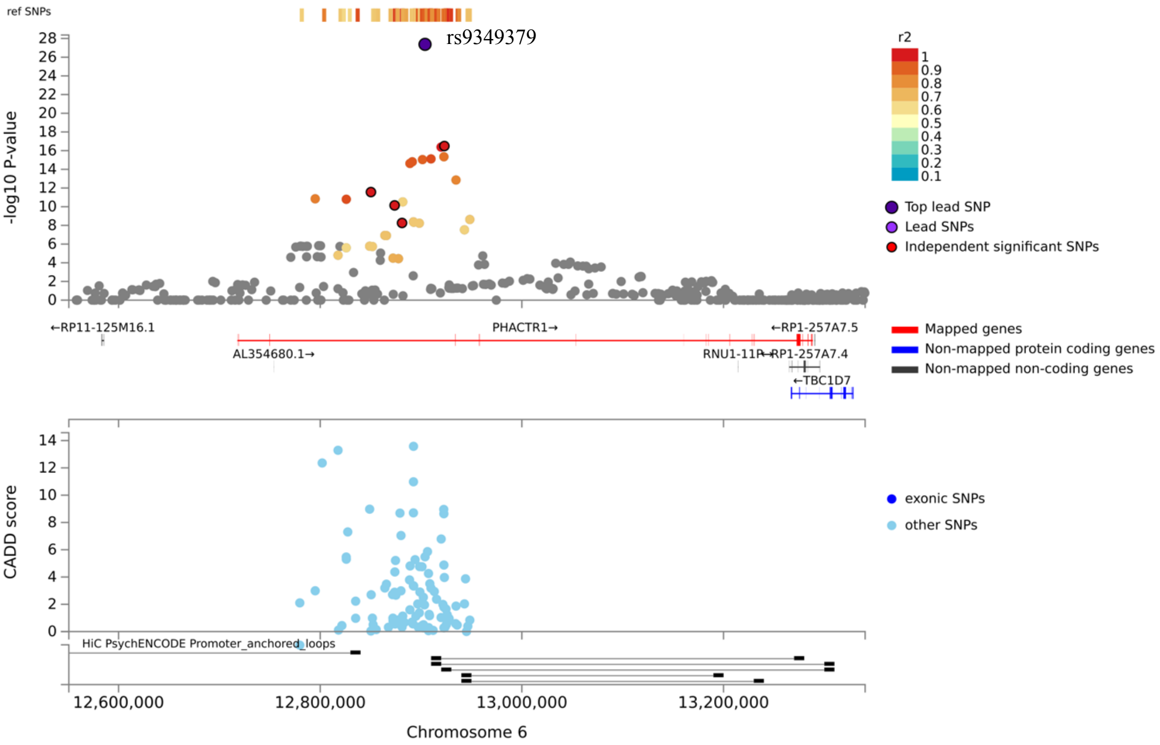

No eQTL of selected tissues exists in this region.

Locus37 6p22.1

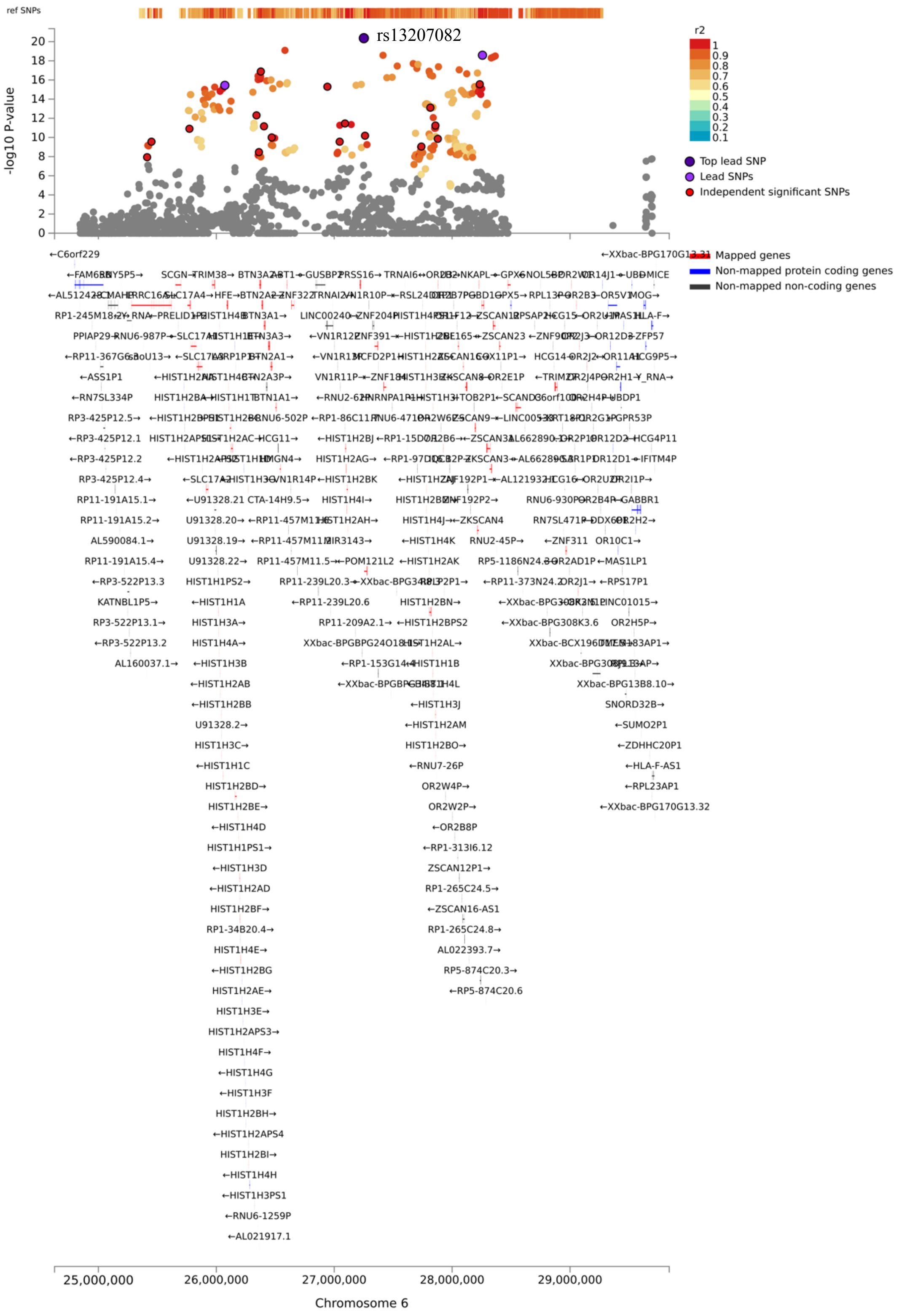

Locus38 6p21.31

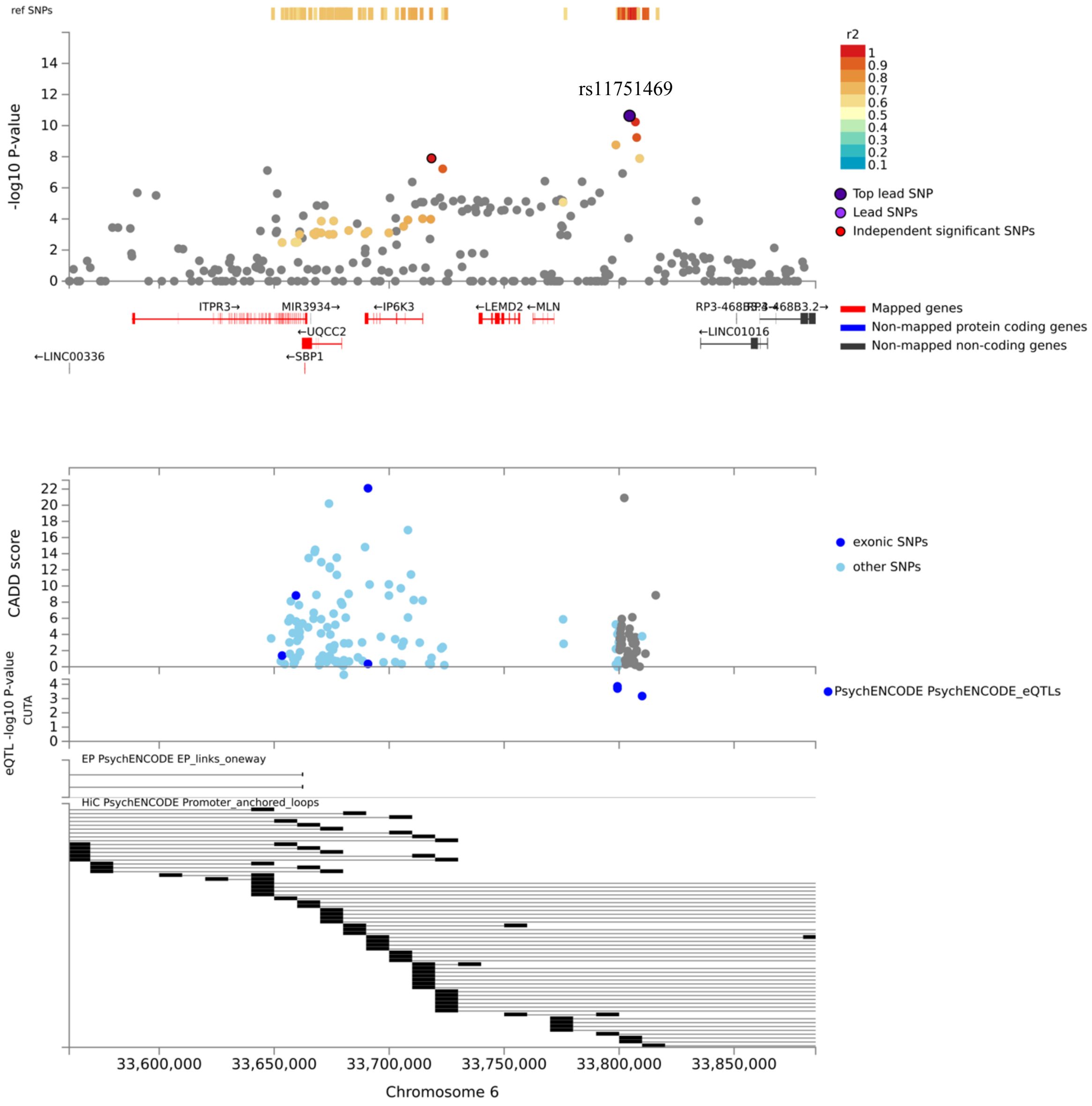

Locus39 6p21.31

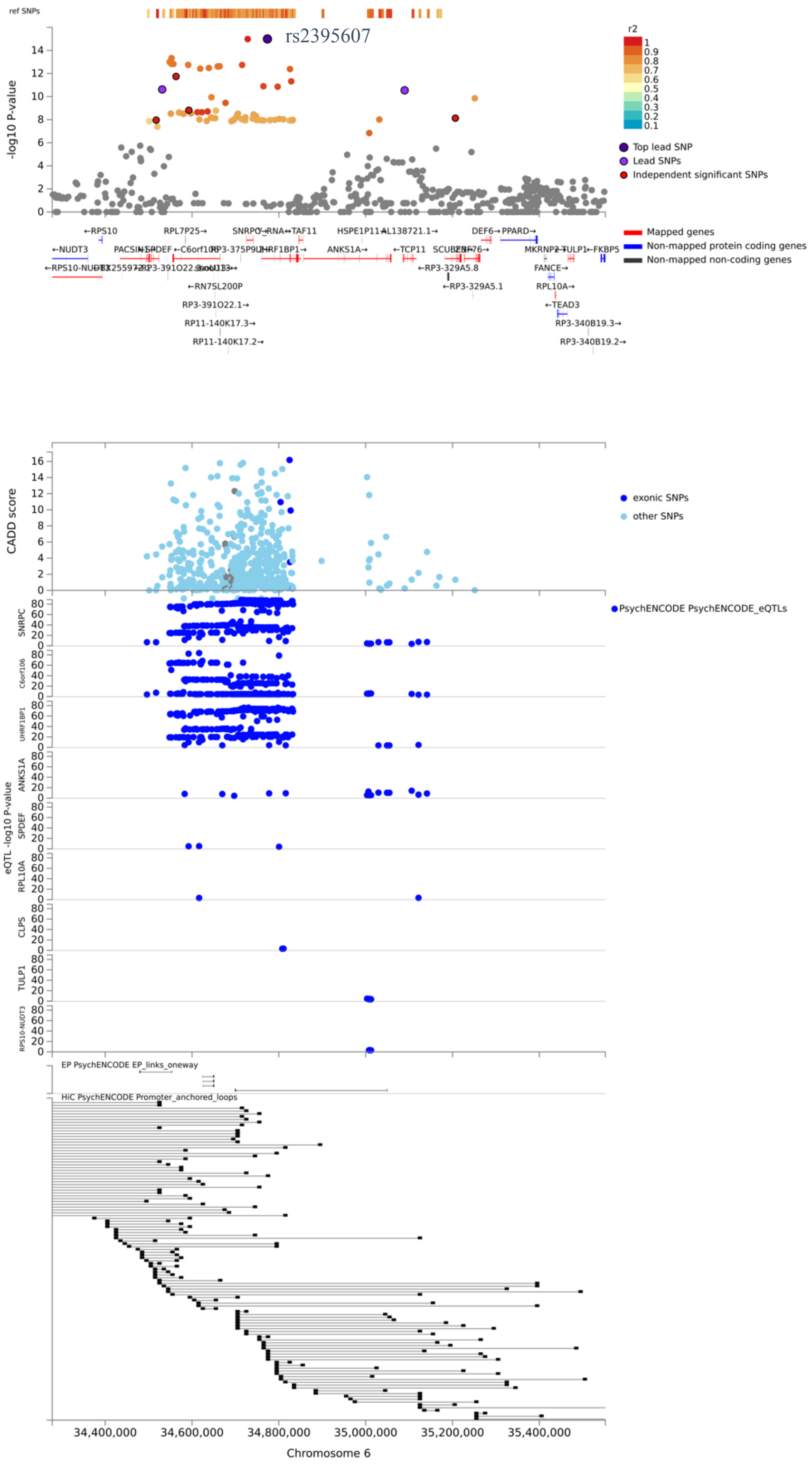

Locus40 6p21.1

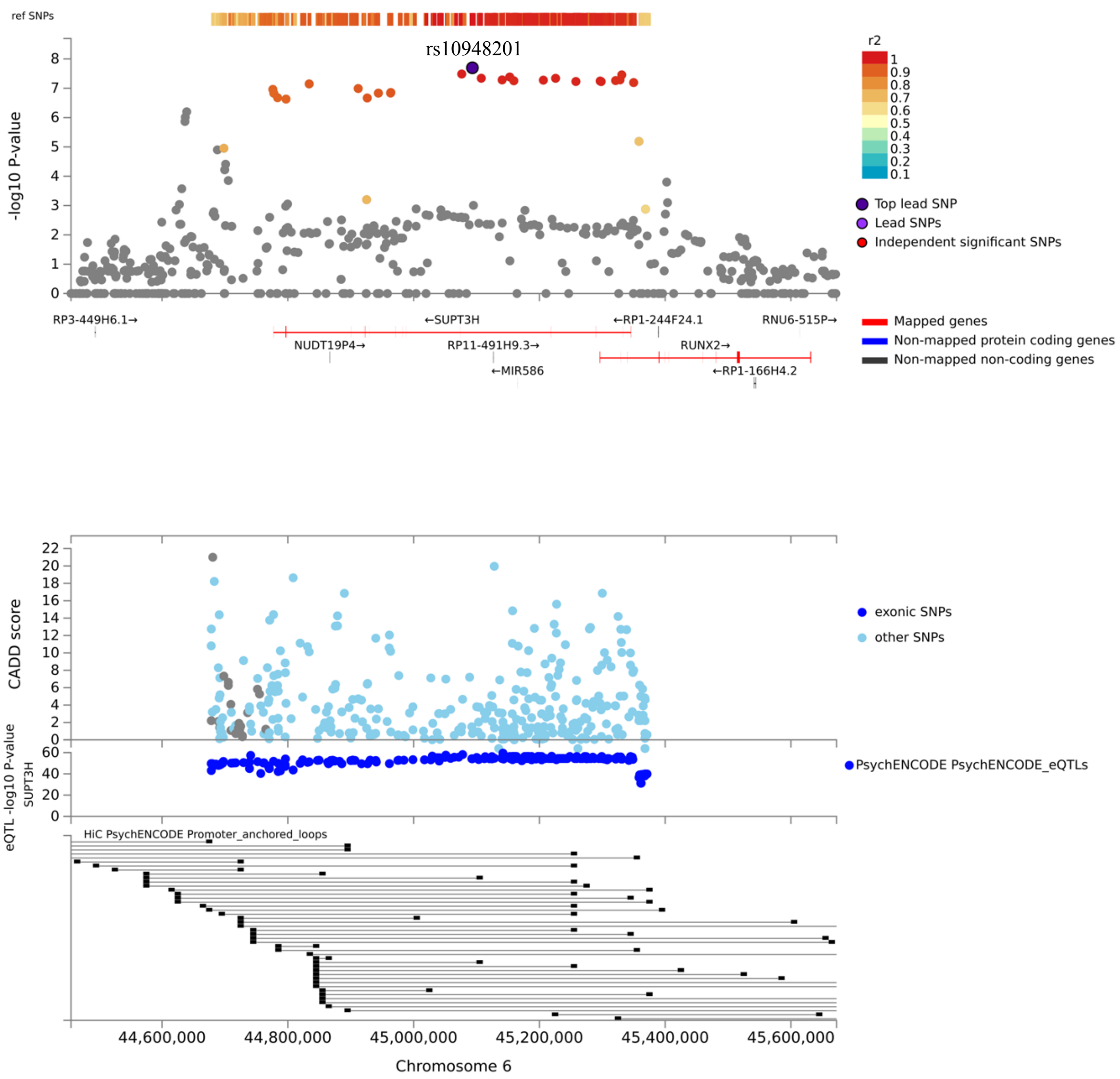

Locus41 6q16.1

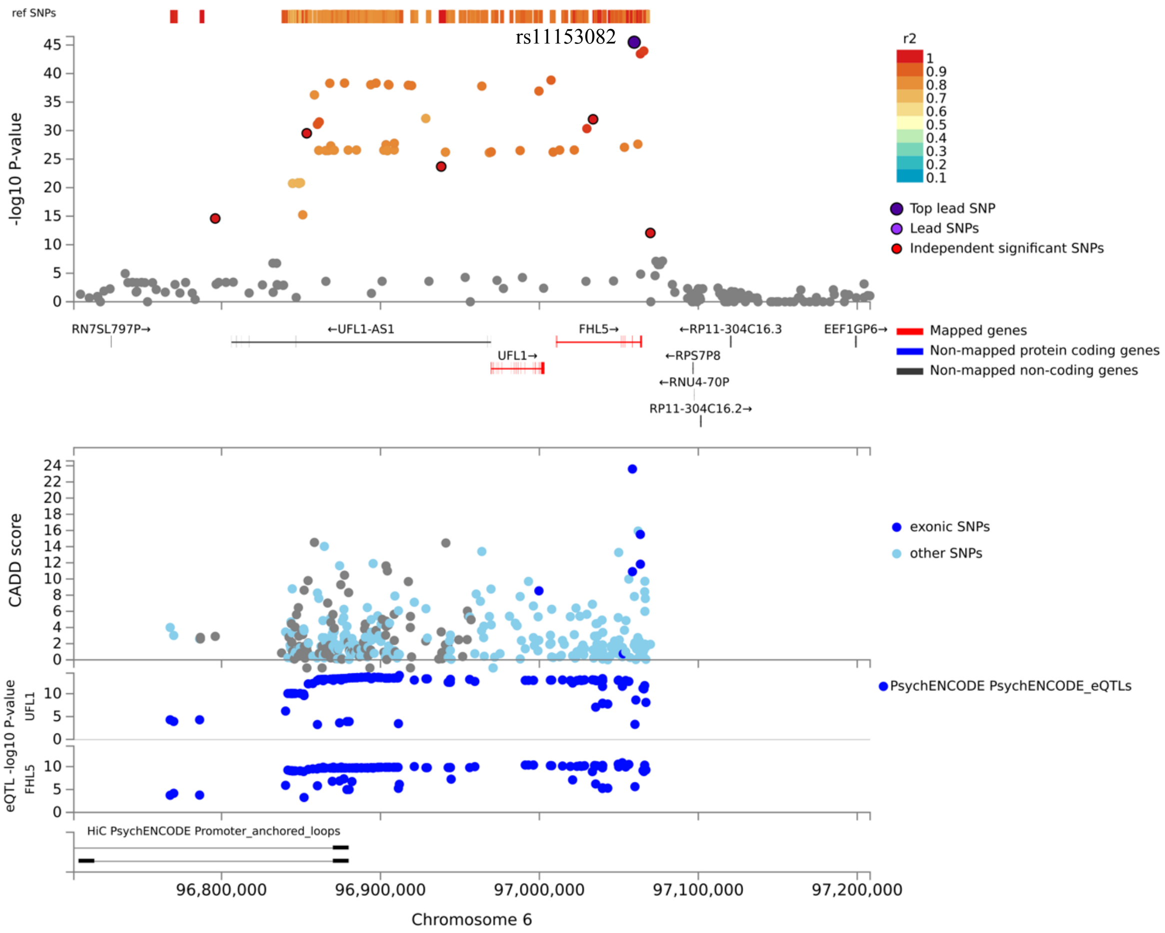

Locus42 6q22.31

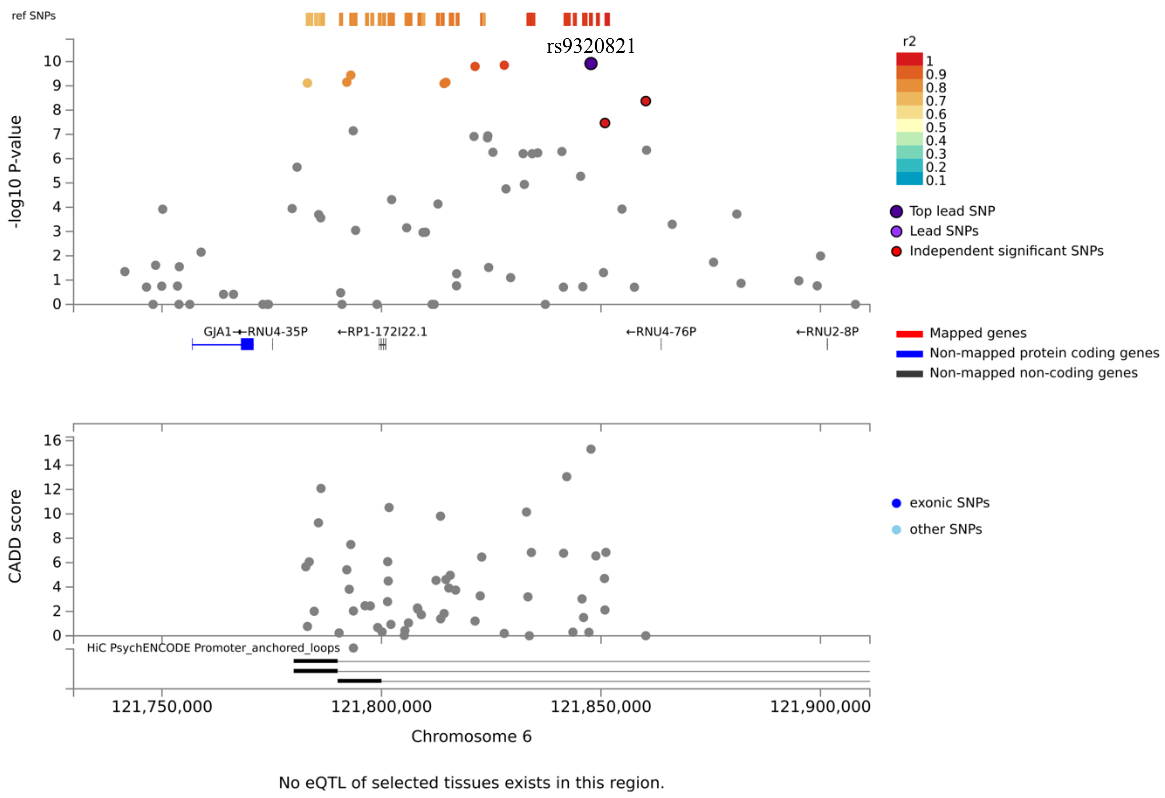

Locus43 6q22.33

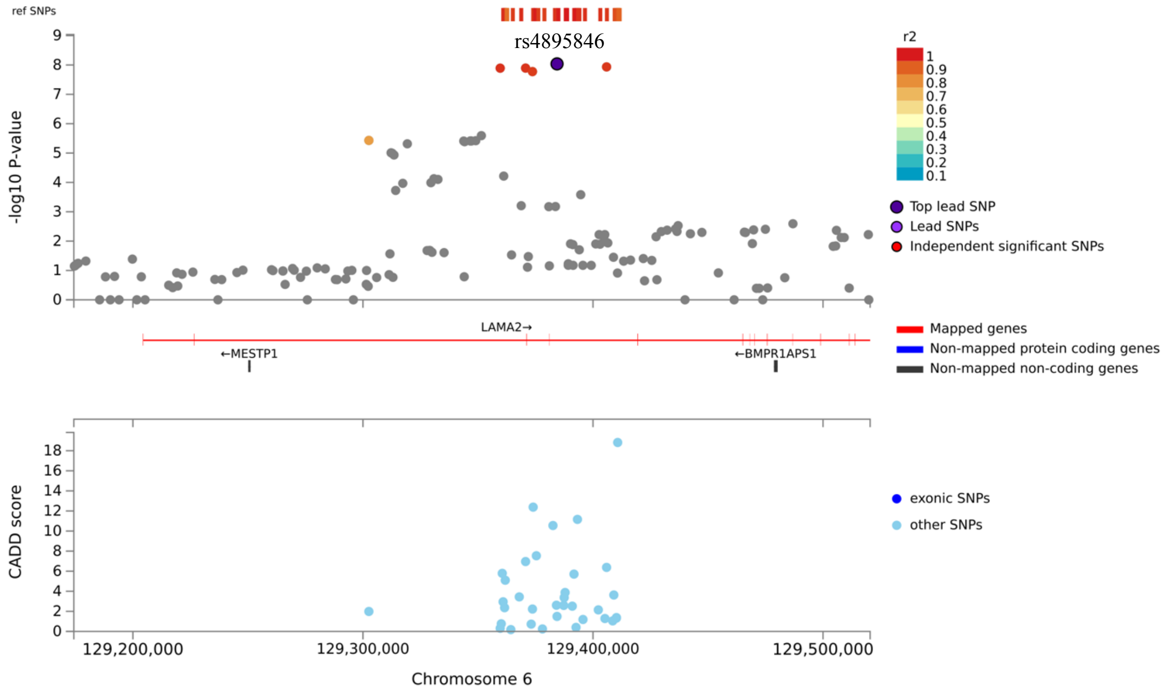

No eQTL of selected tissues exists in this region.

Locus44 6q25.1

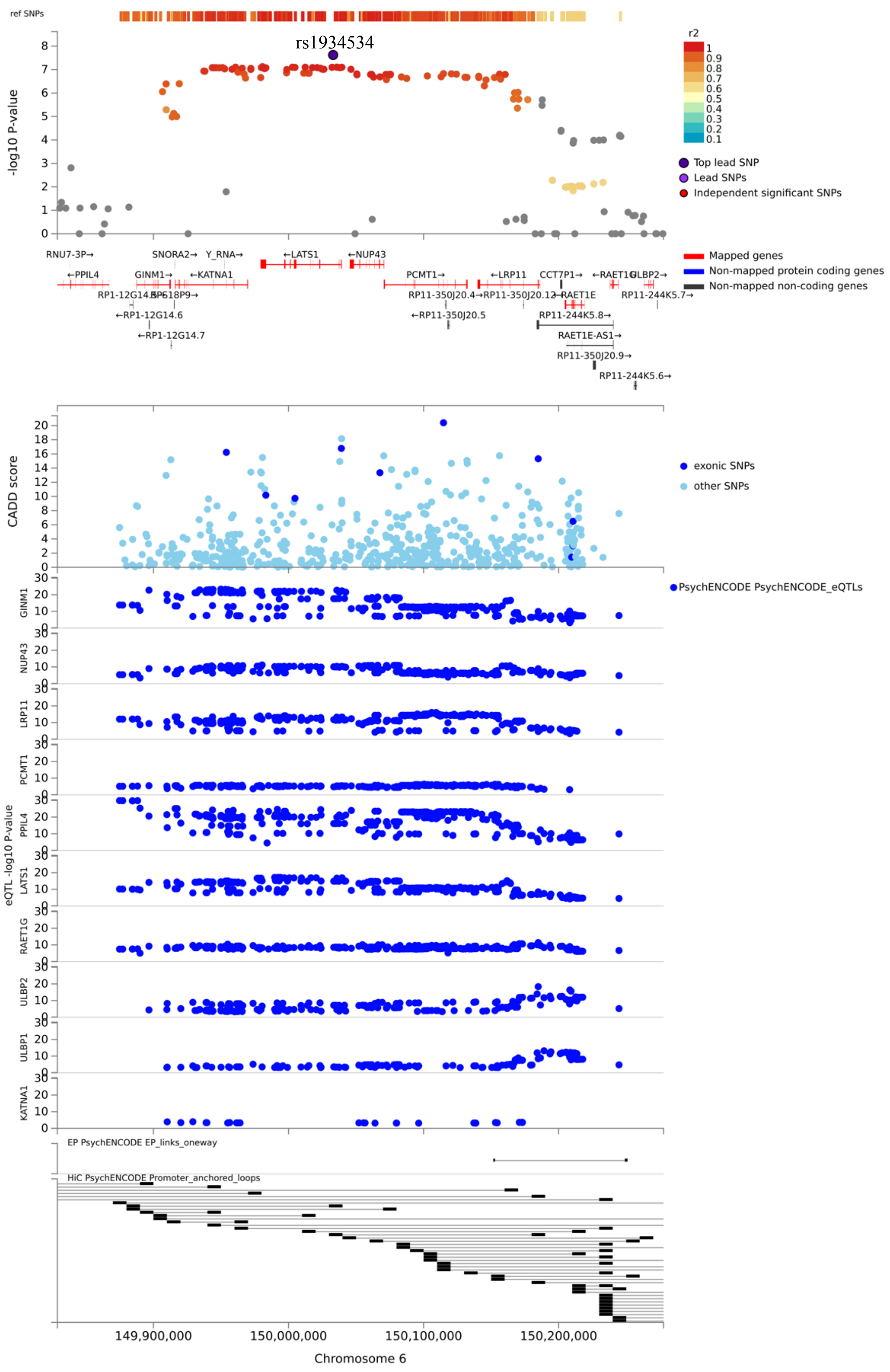

Locus45 6q25.3

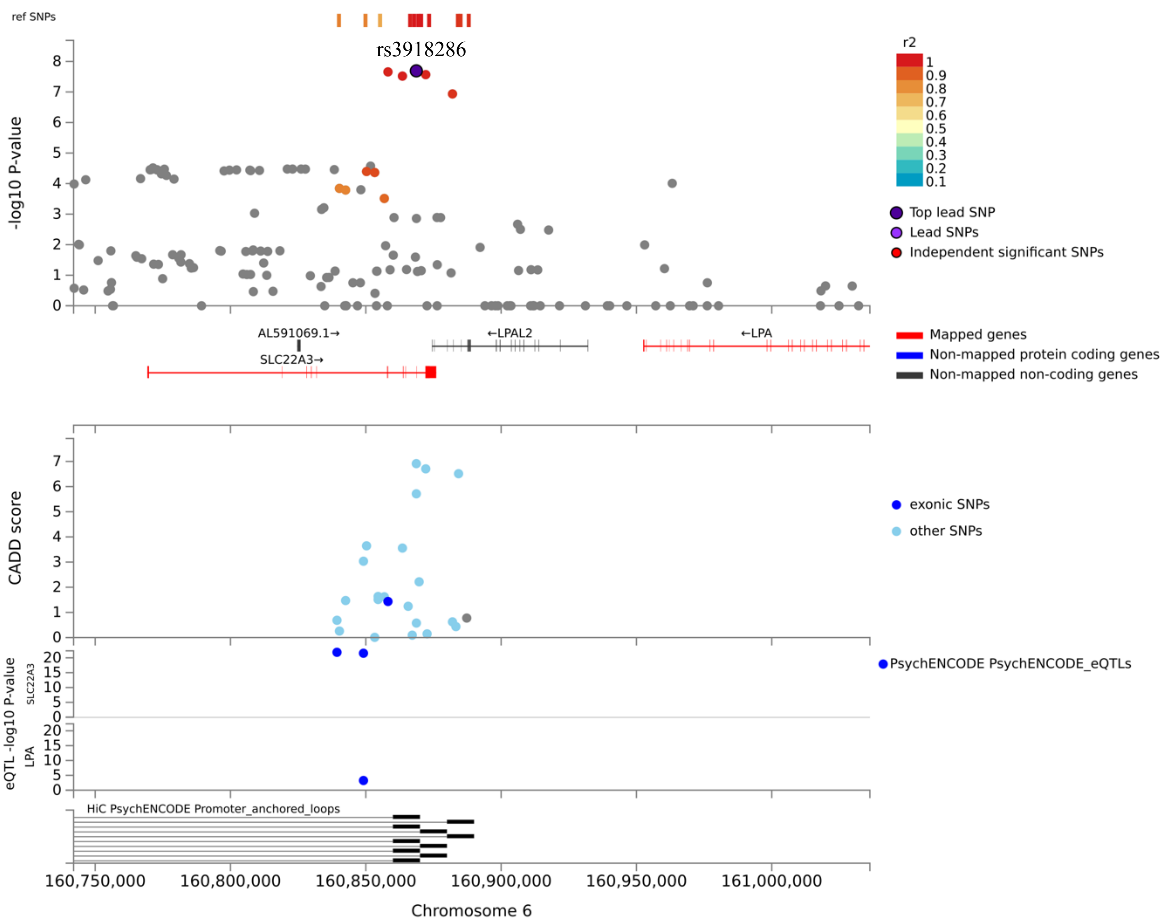

Locus46 7p22.3

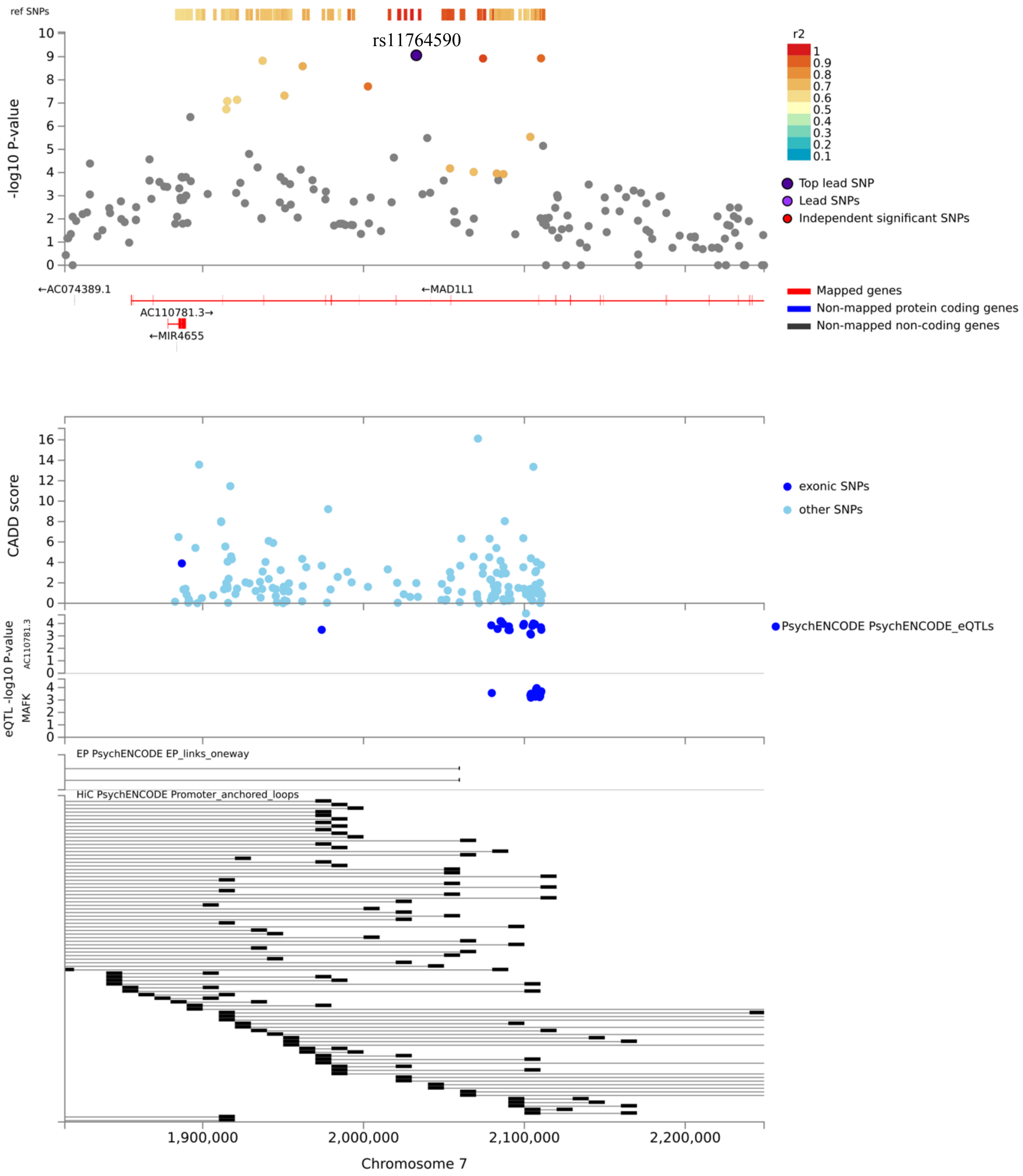

Locus47 7p15.3

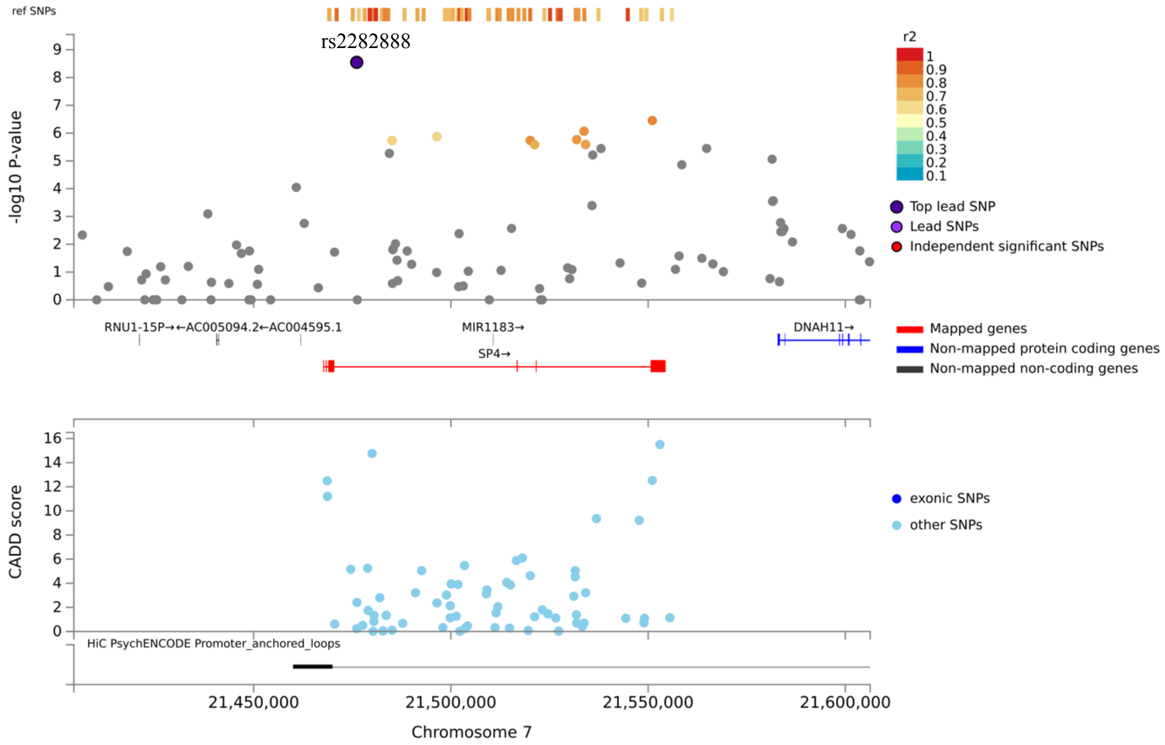

No eQTL of selected tissues exists in this region.

Locus48 7p14.1

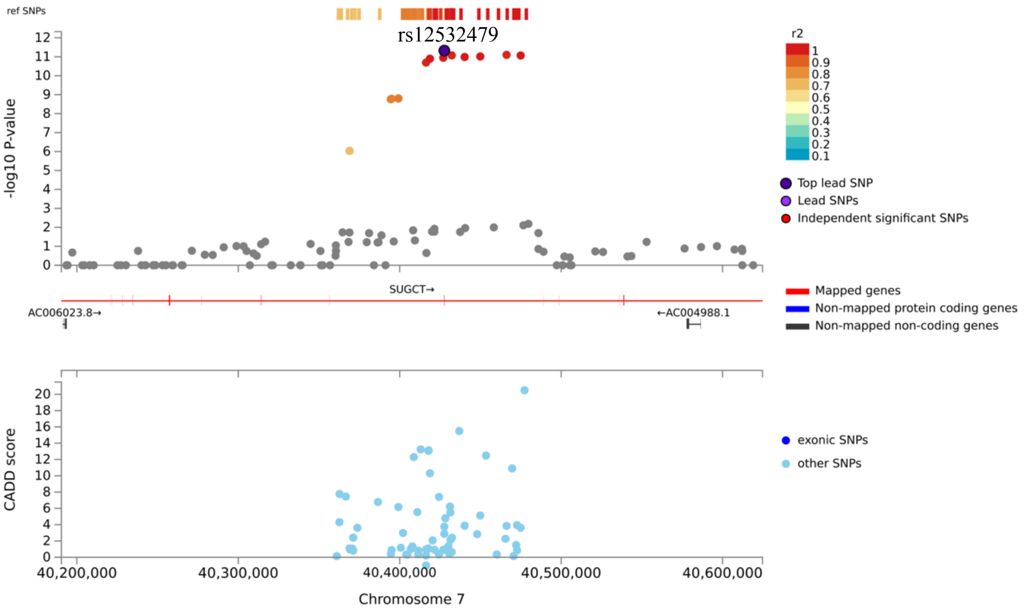

No eQTL of selected tissues exists in this region.

Locus49 7q31.1

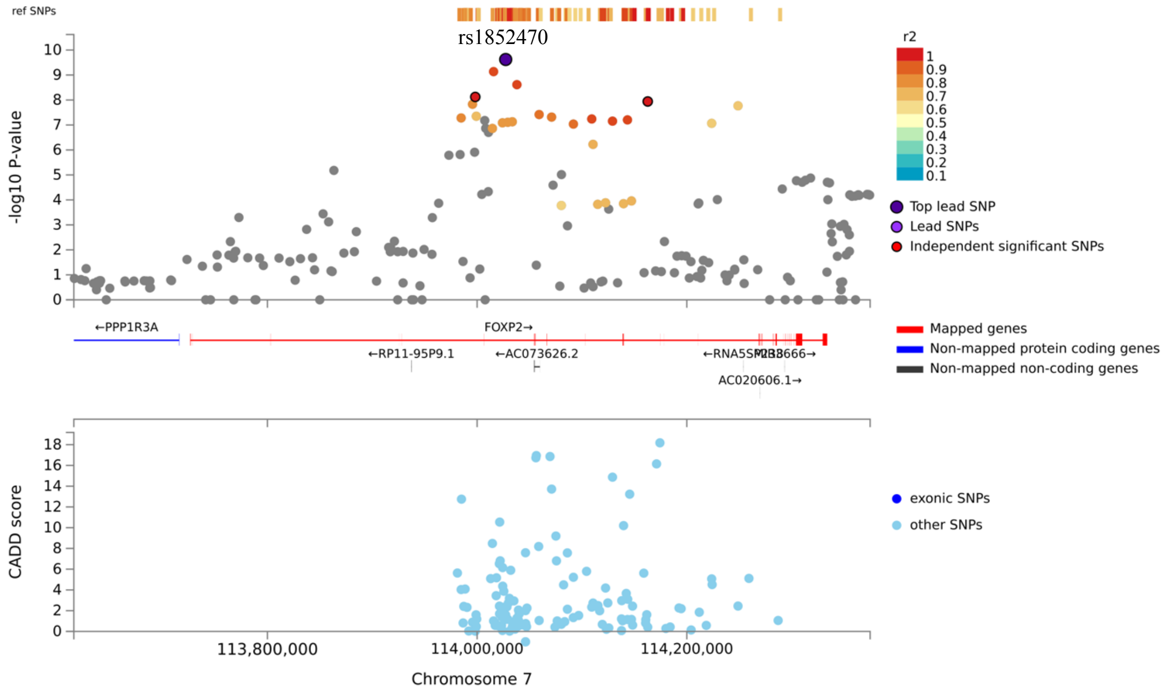

No eQTL of selected tissues exists in this region.

Locus50 7q31.31

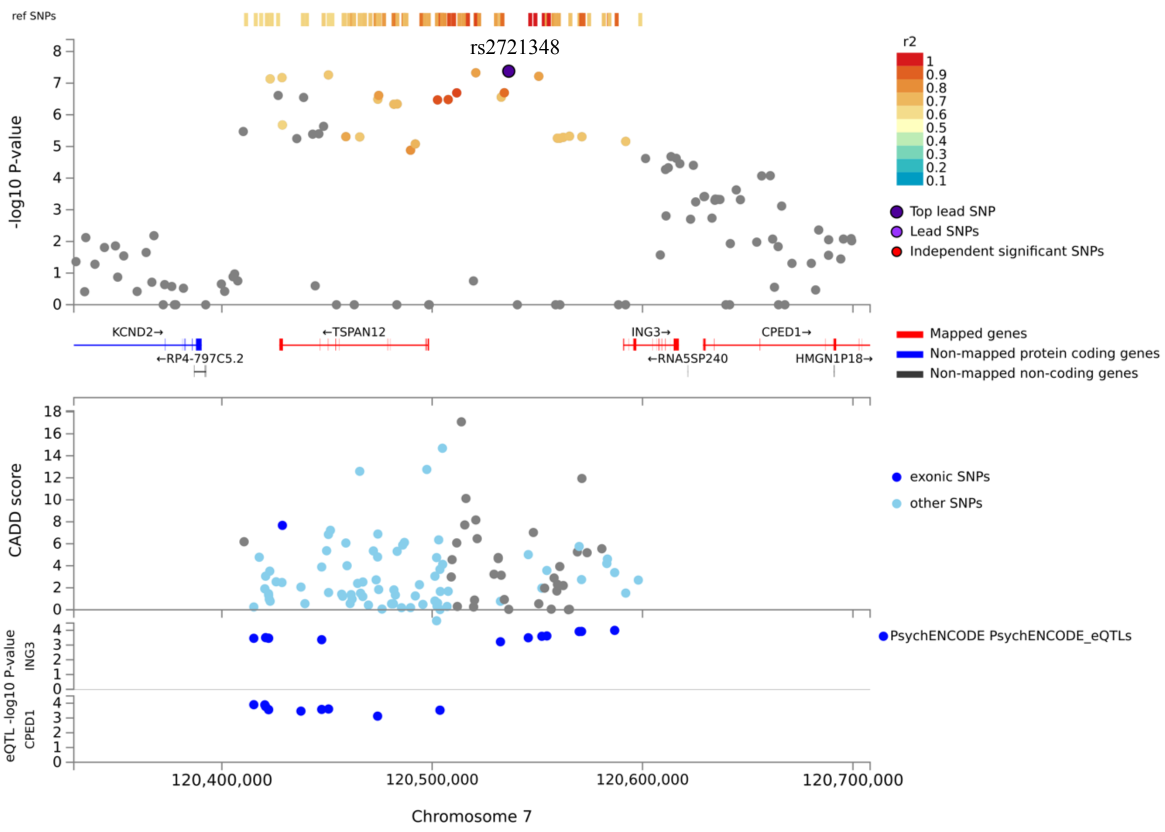

Locus51 8p23.1

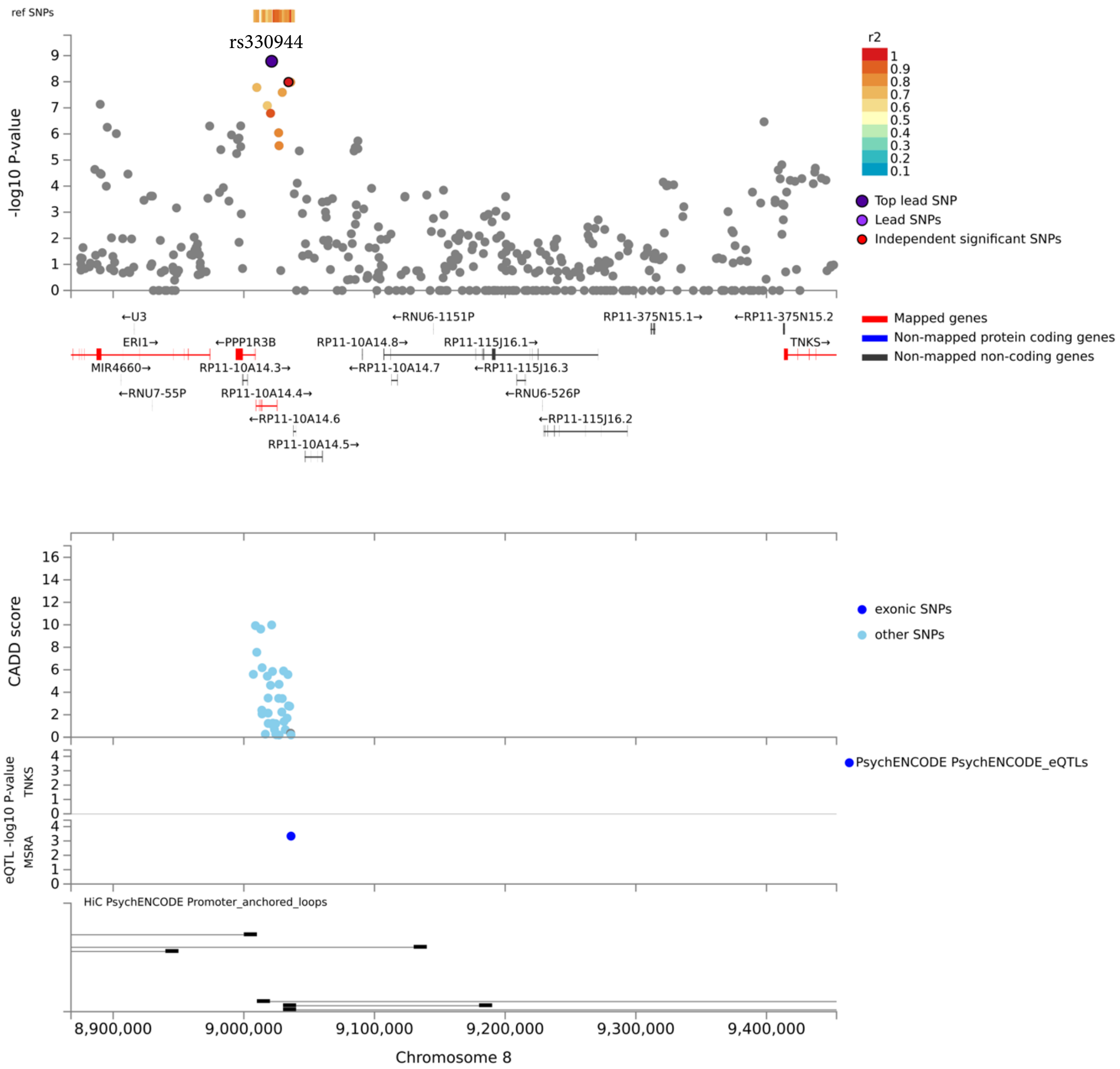

Locus52 8p21.3

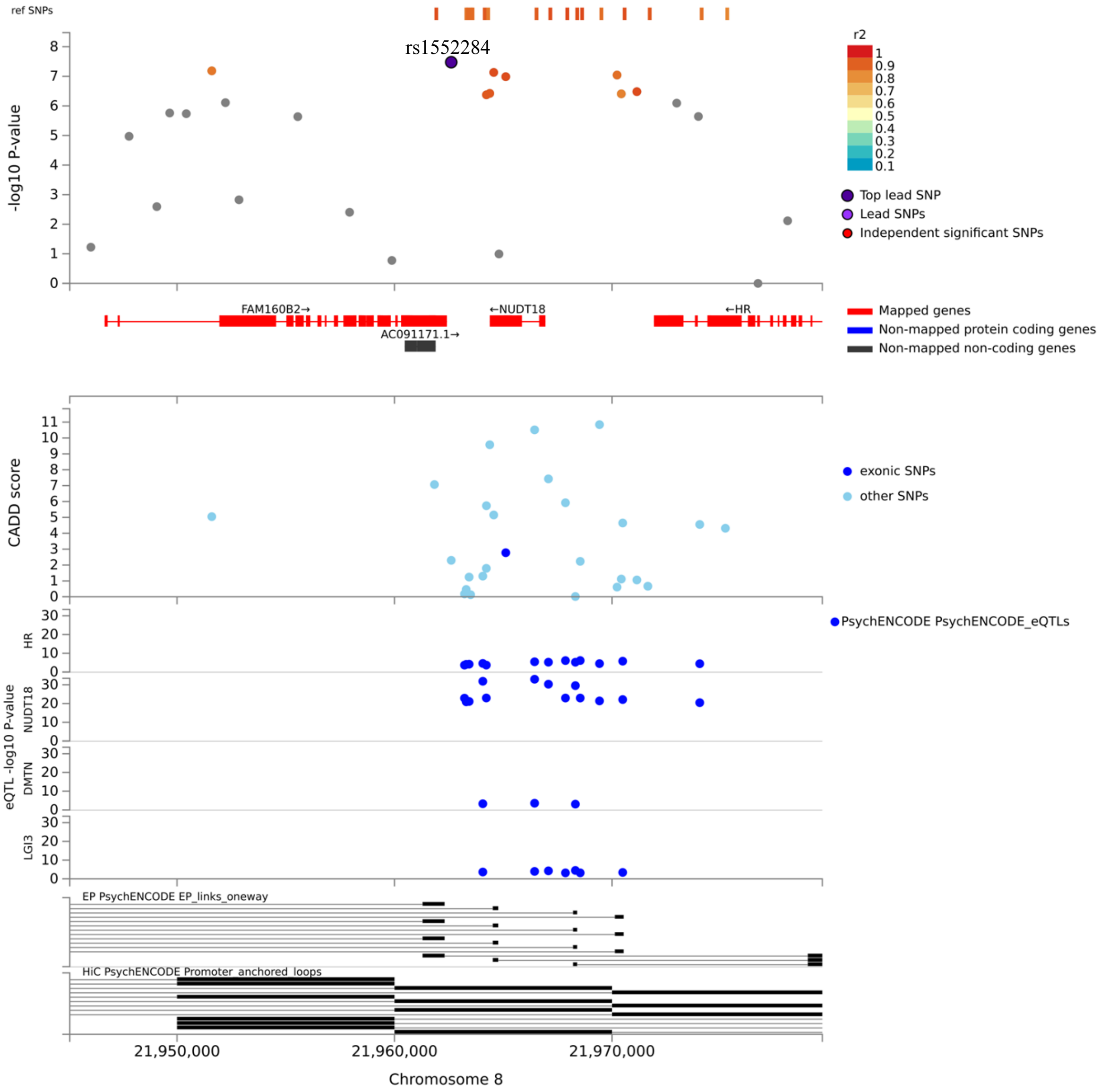

Locus53 8q13.3

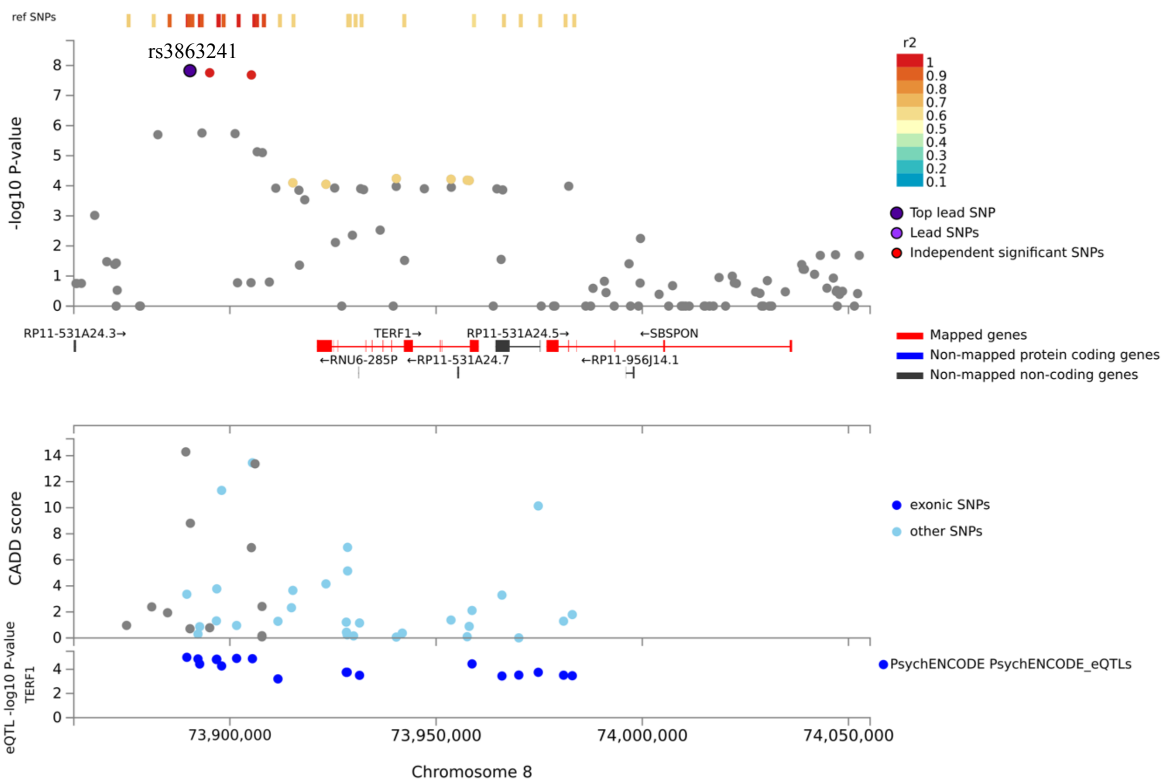

Locus54 8q24.3

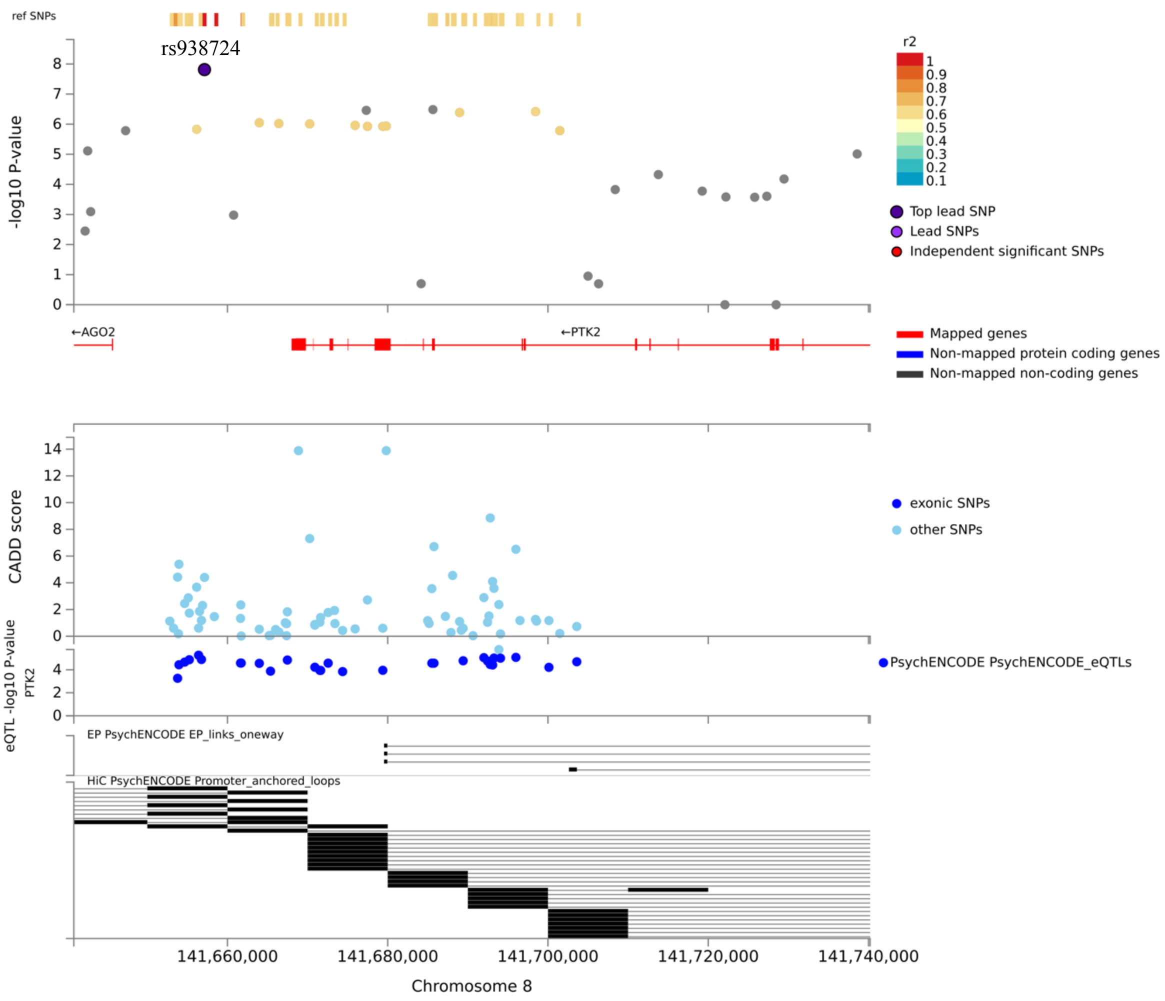

Locus55 9p21.3

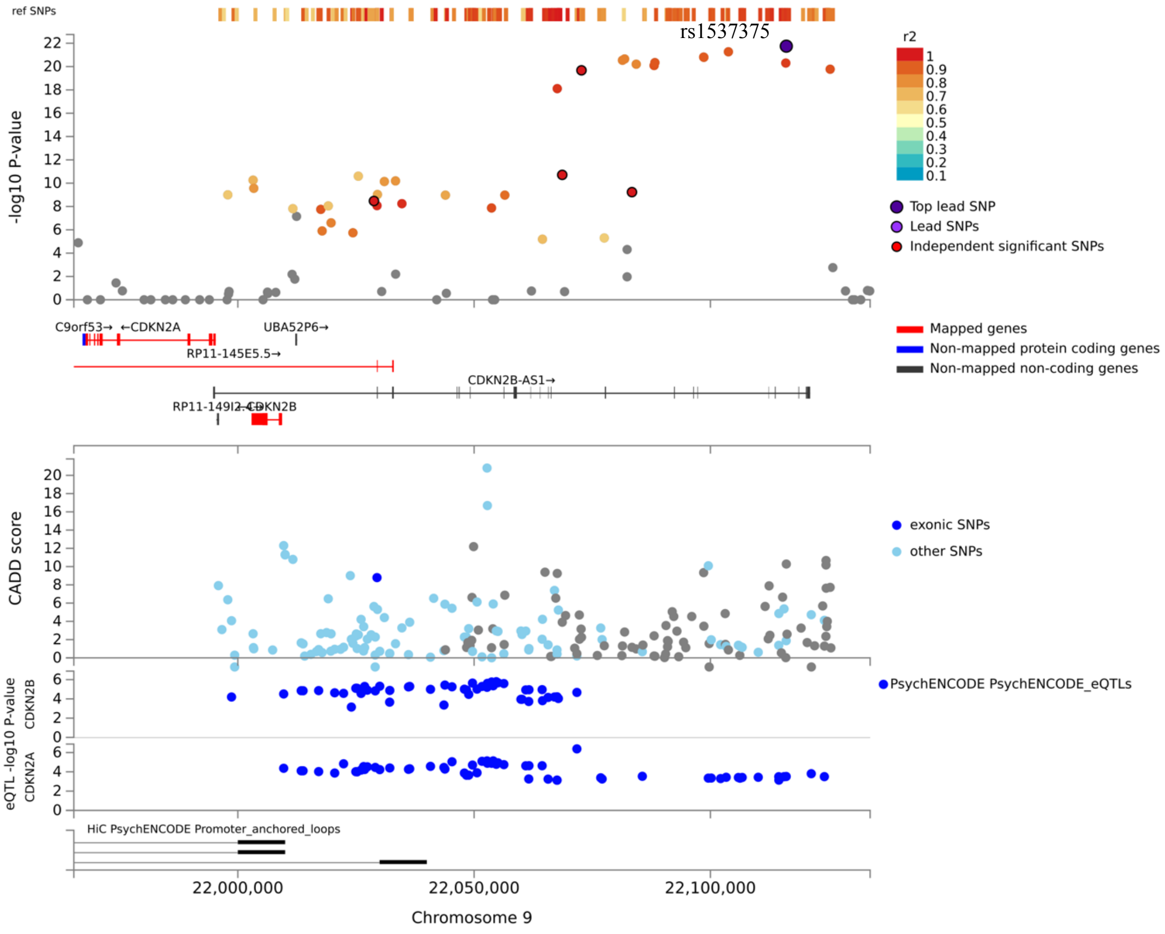

Locus56 9q21.11

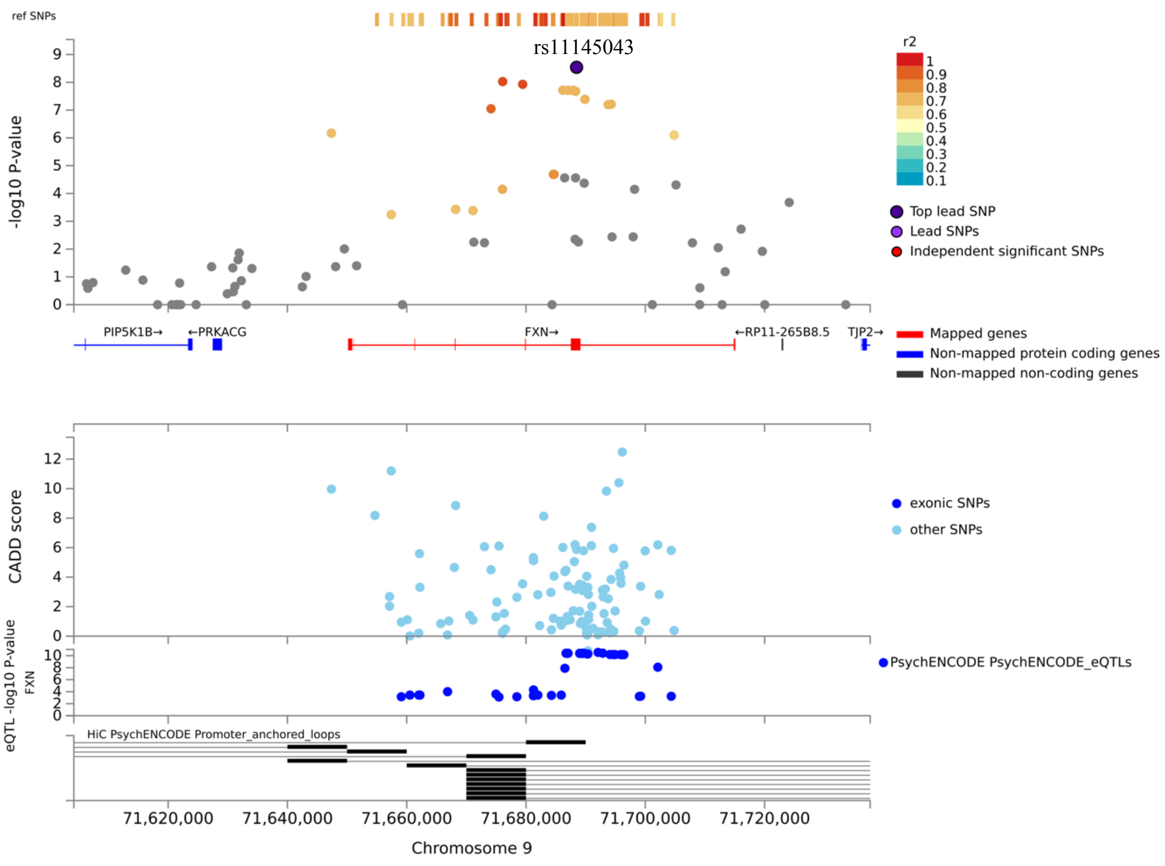

Locus57 9q22.31

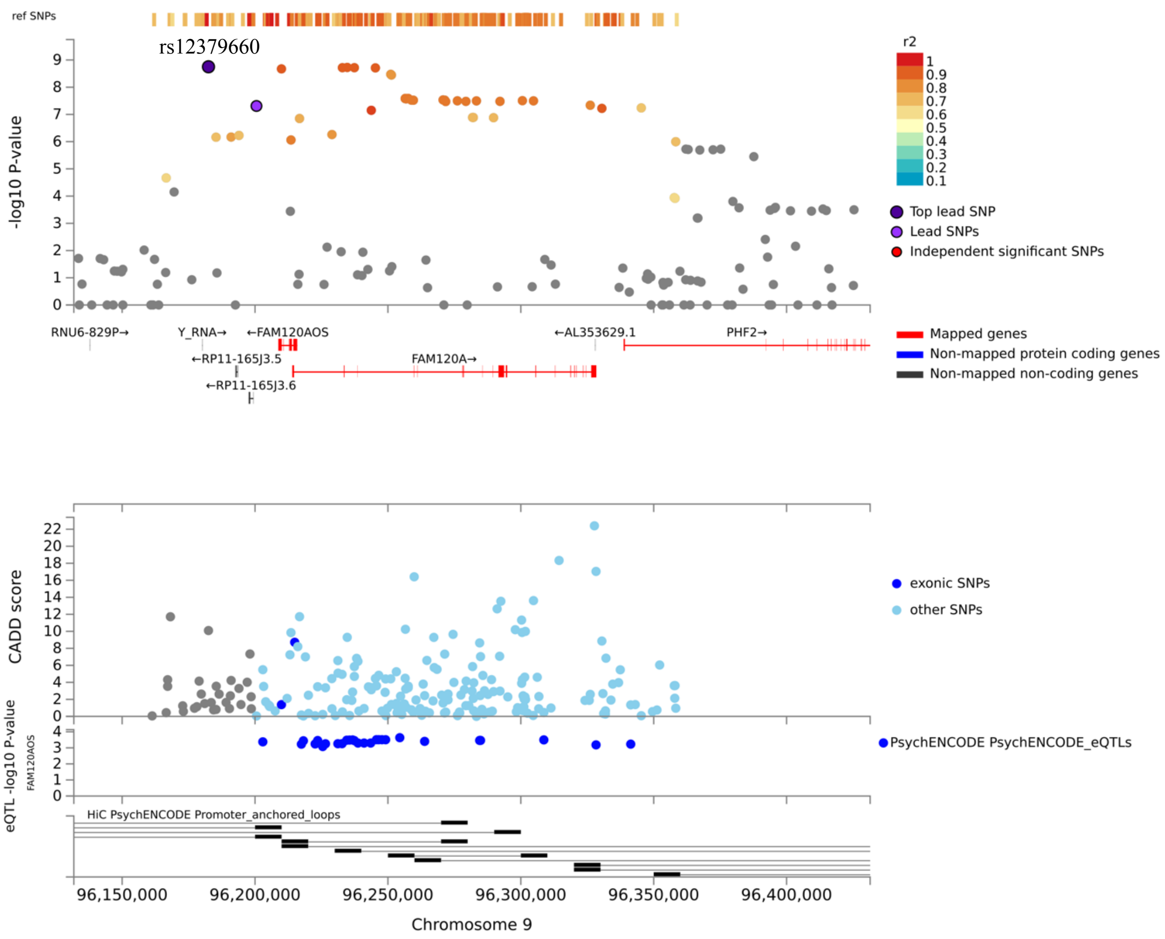

Locus58 9q33.1

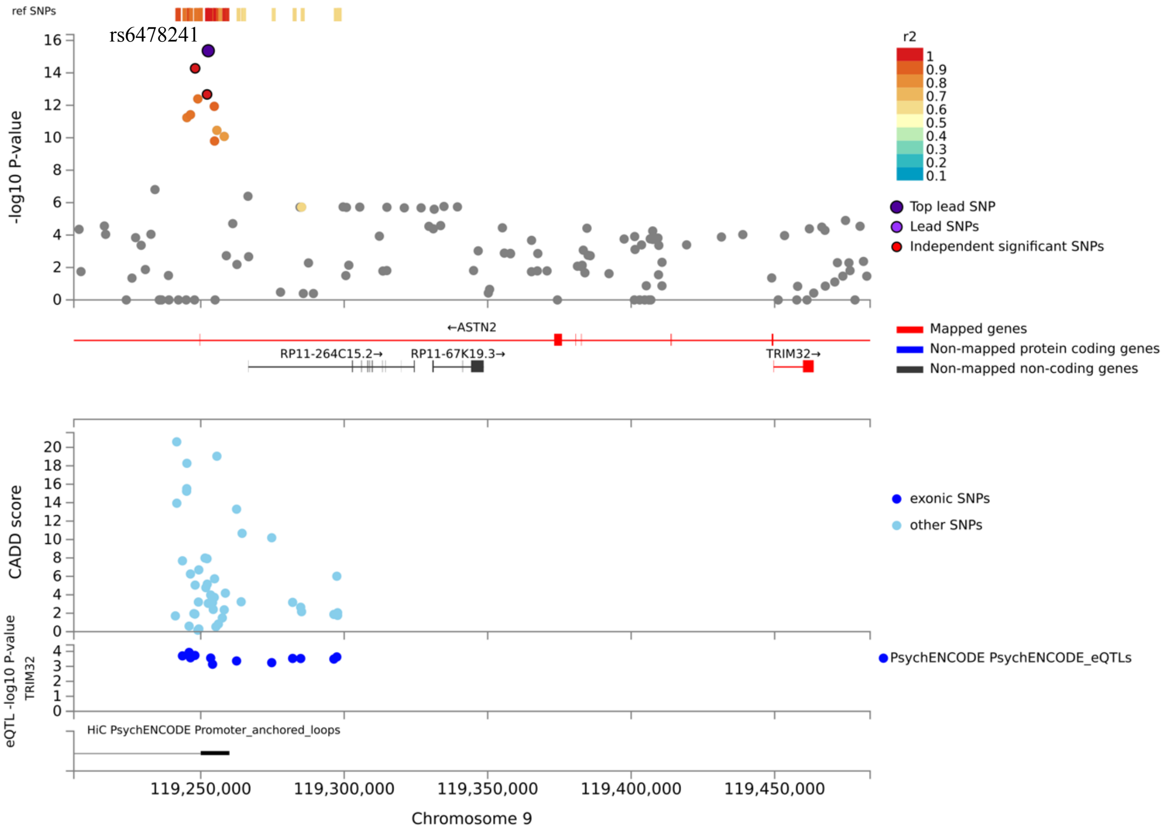

Locus59 10q22.1

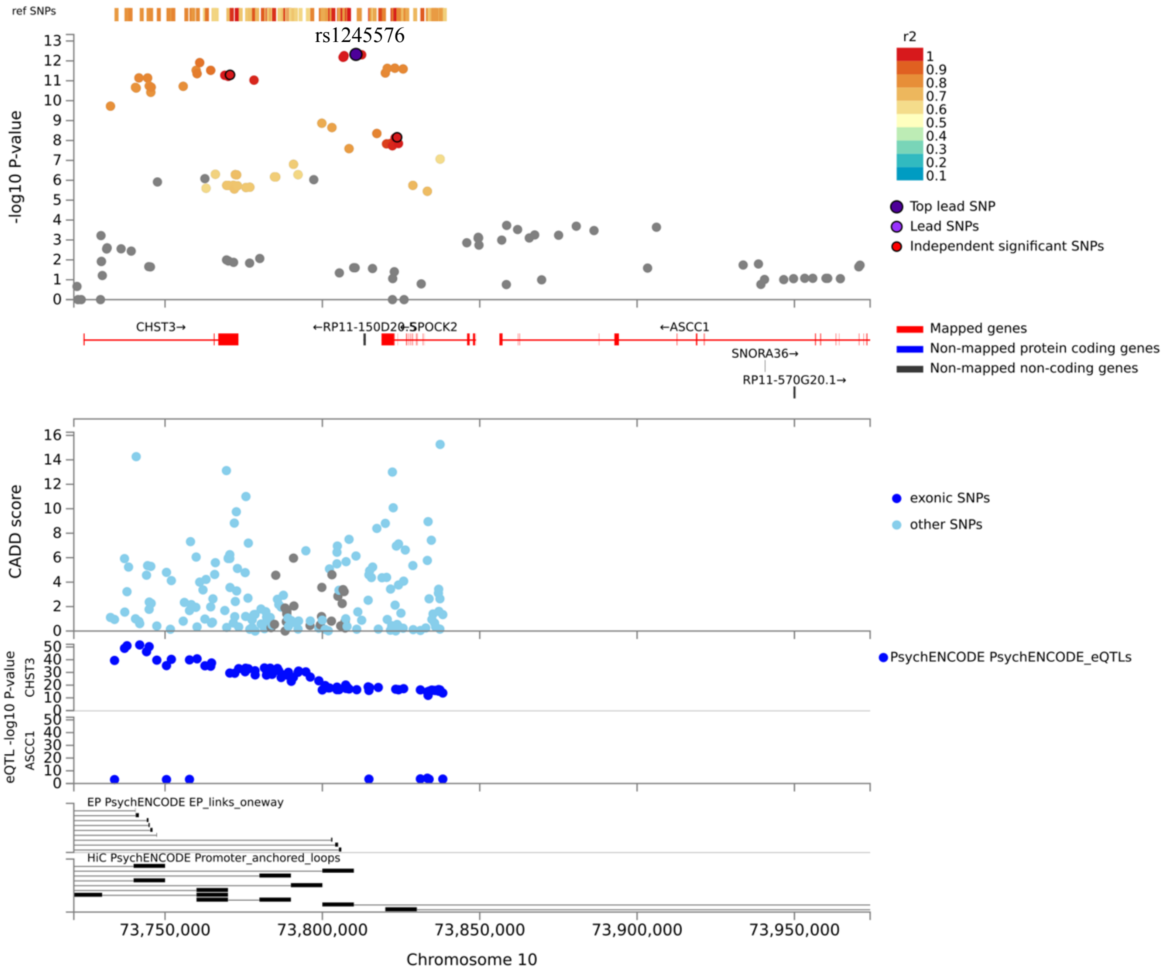

Locus60 10q24.2

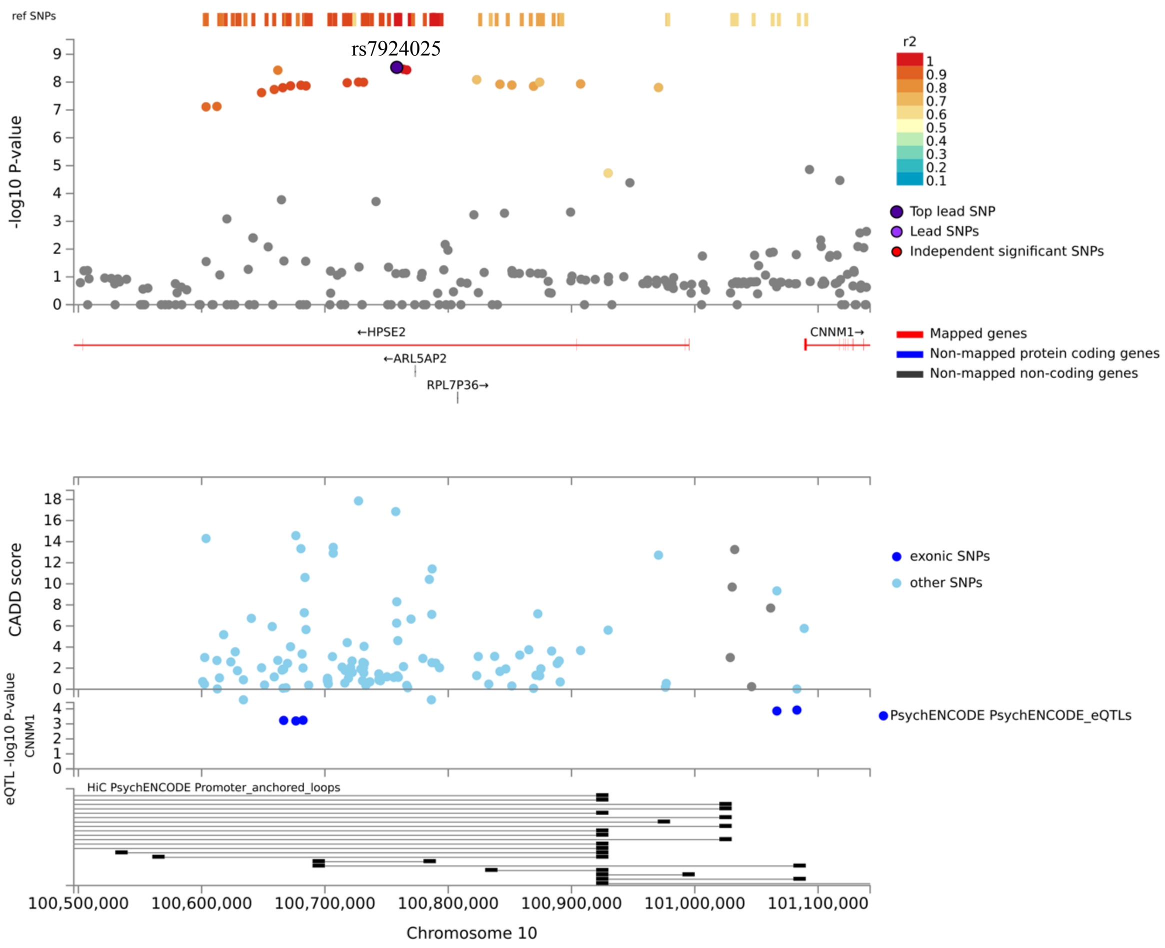

Locus61 10q25.1

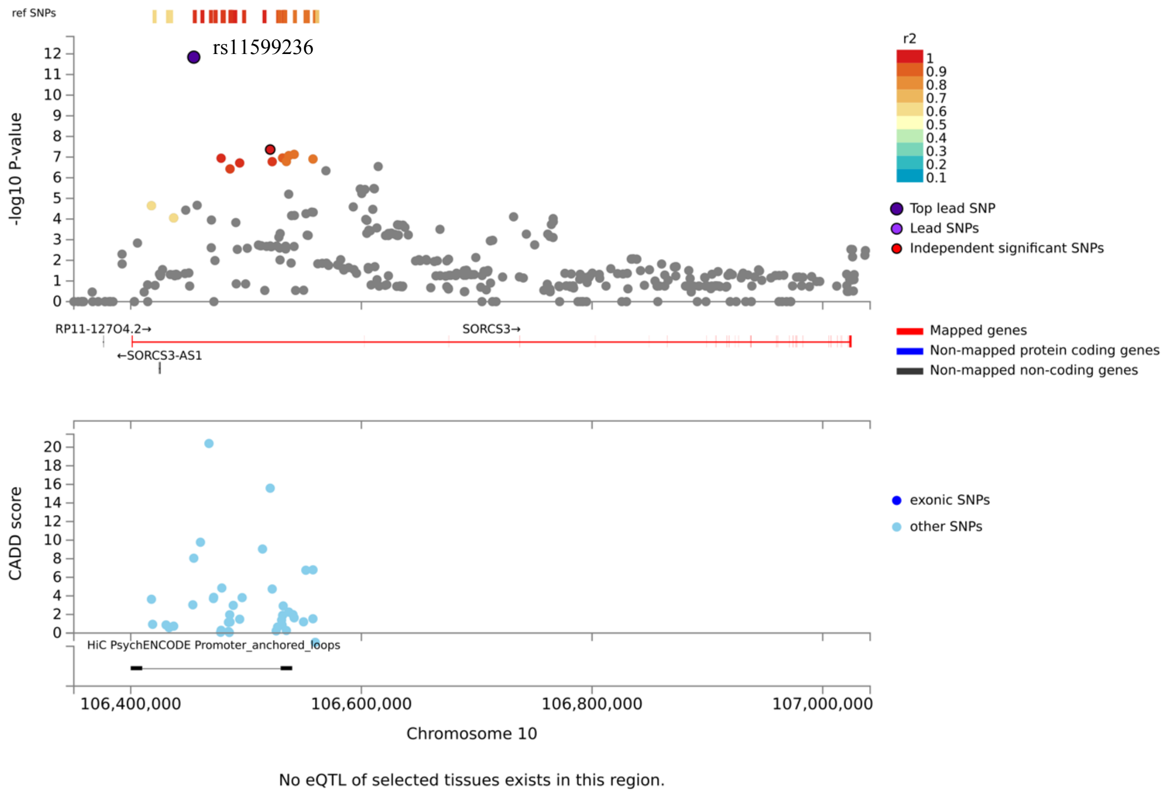

Locus62 10q26.13

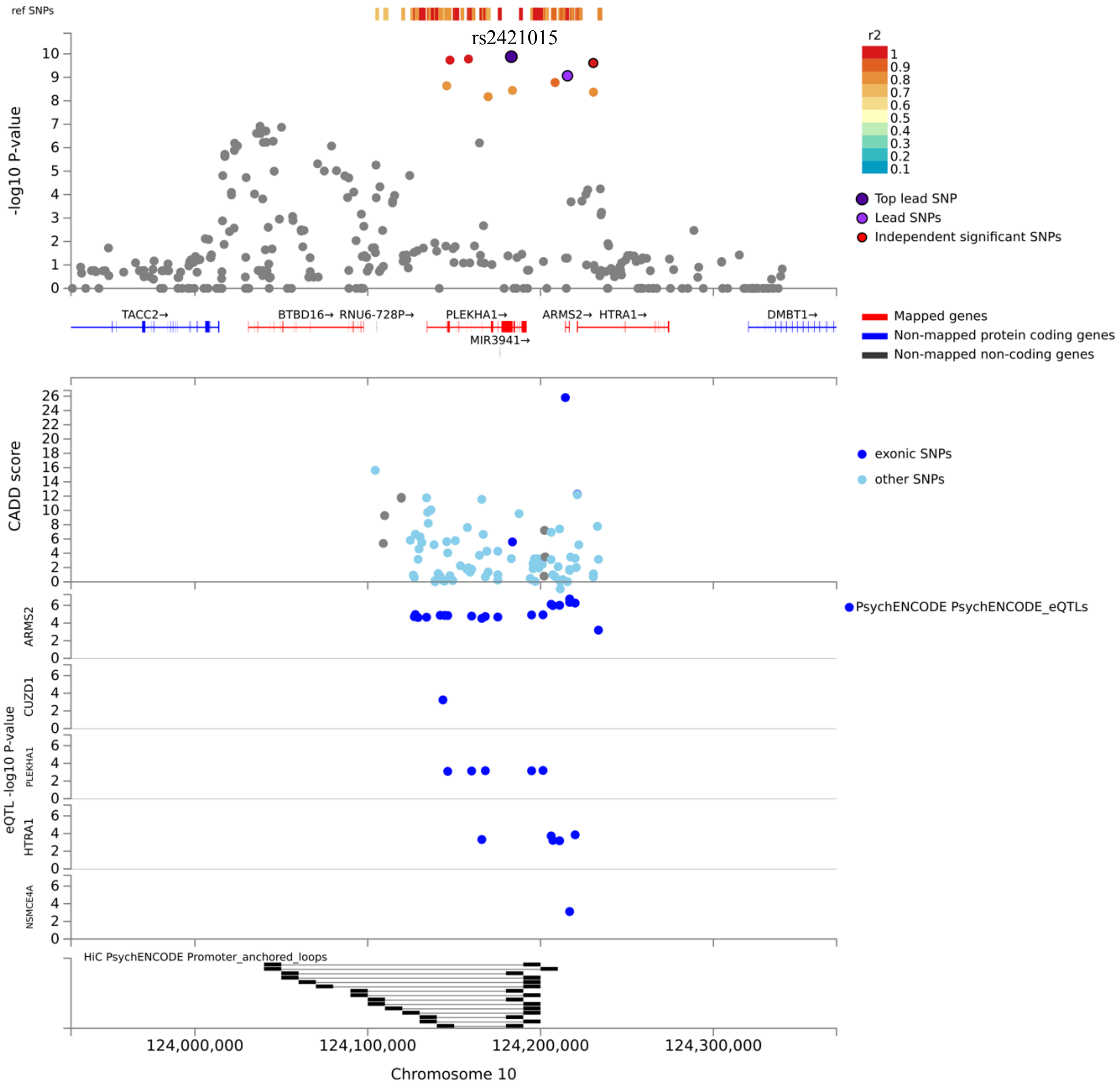

Locus63 11p15.4

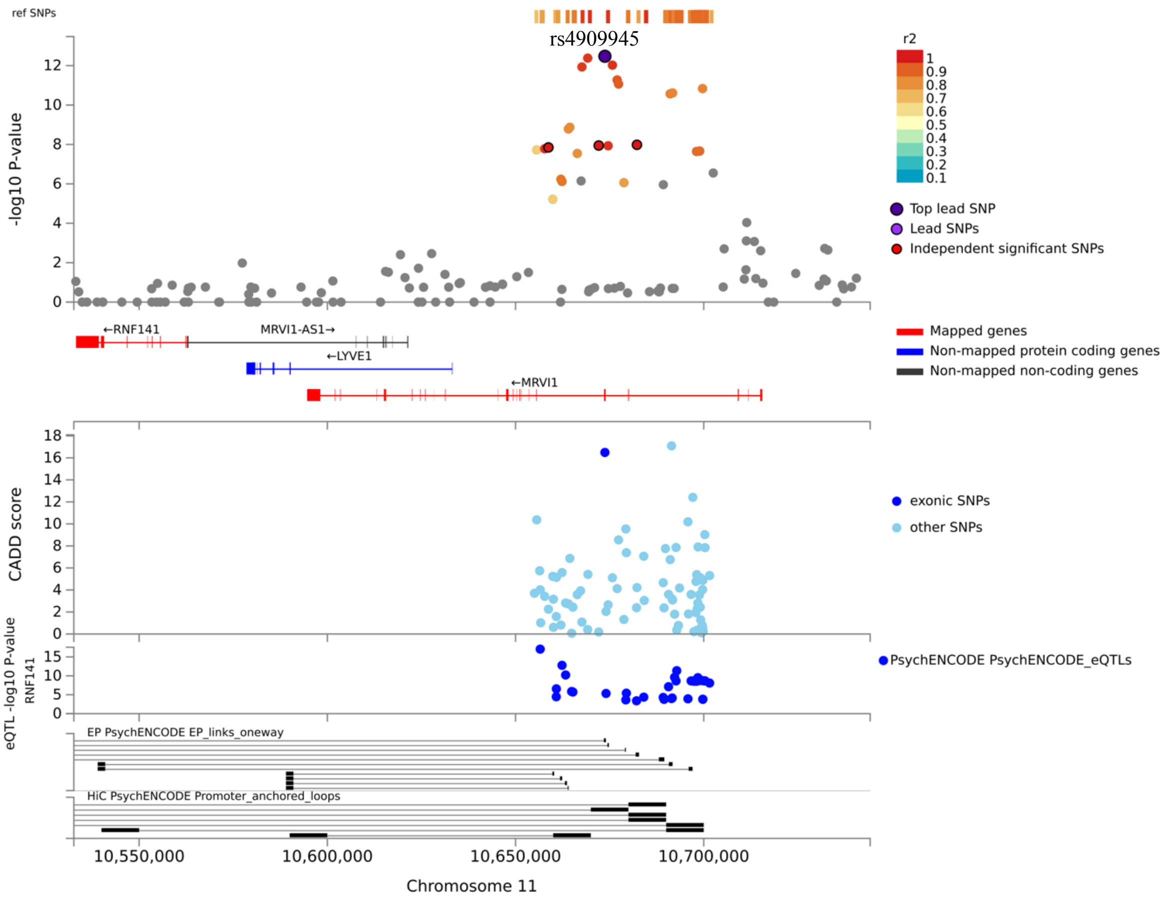

Locus64 11q23.2

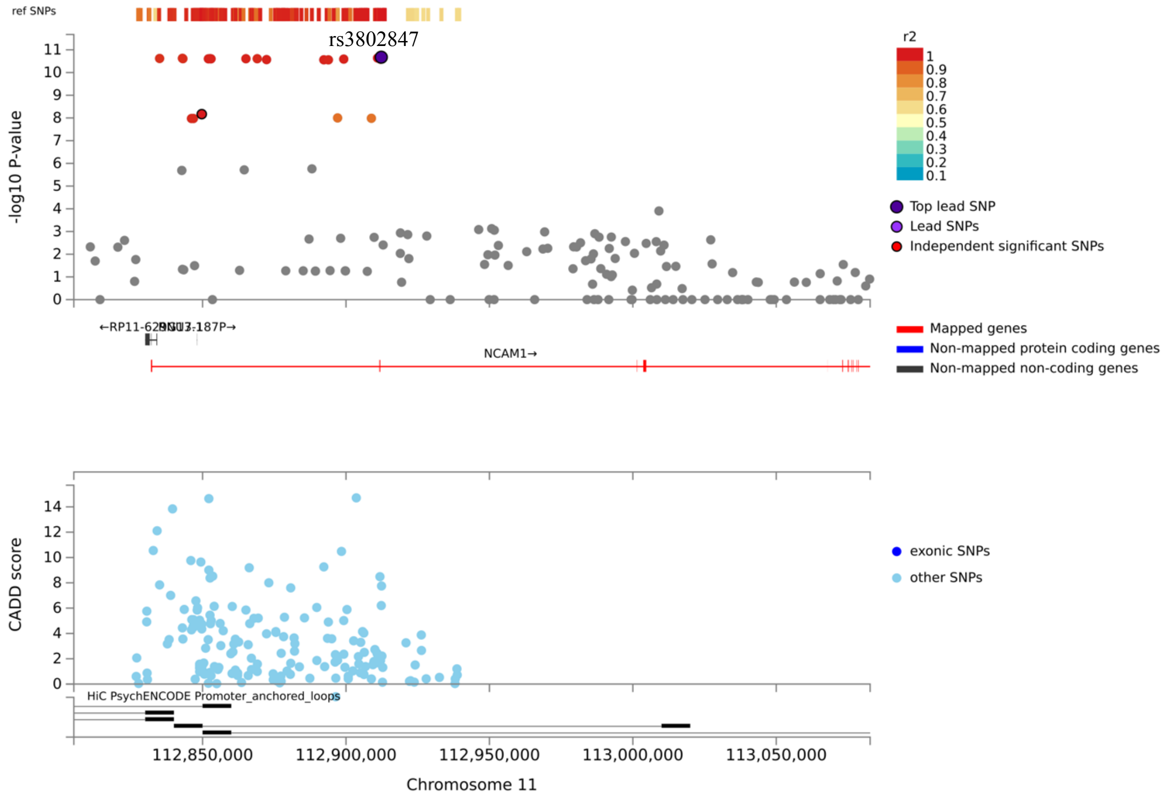

No eQTL of selected tissues exists in this region.

Locus65 12p13.33

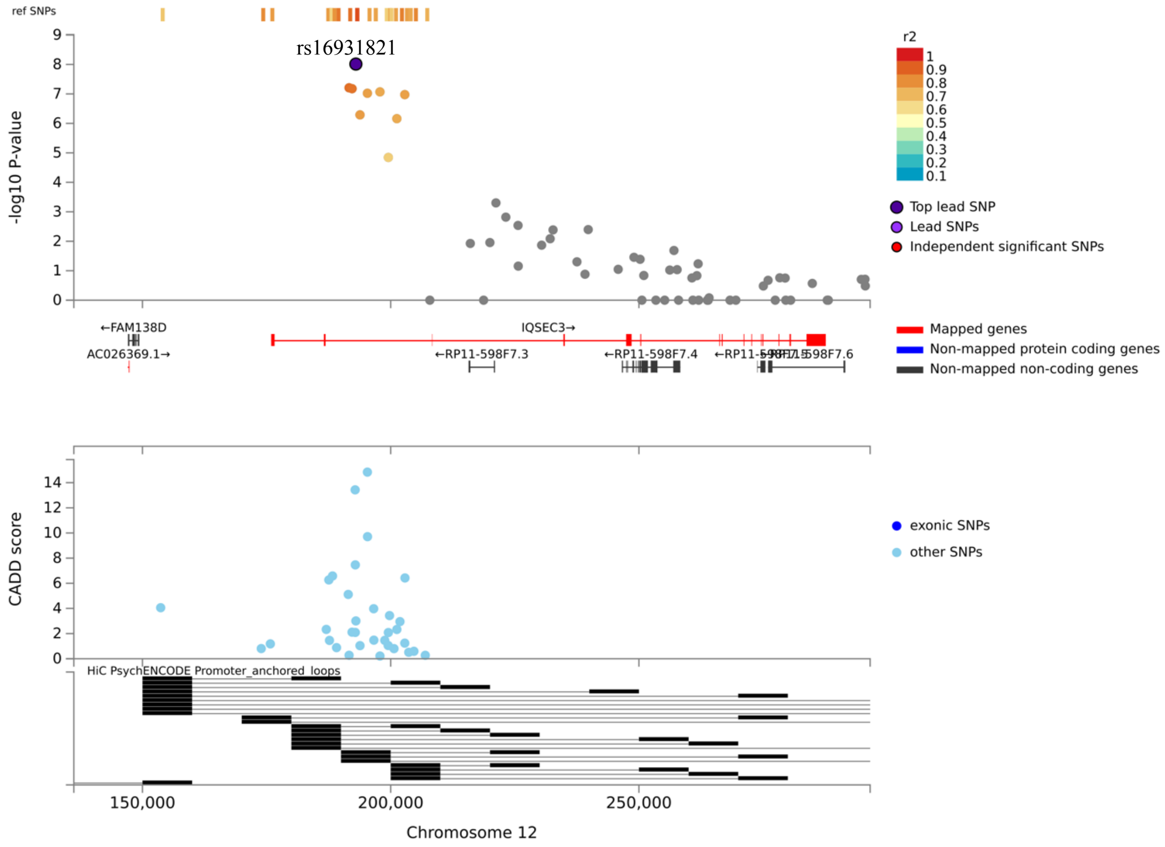

No eQTL of selected tissues exists in this region.

Locus66 12p13.32

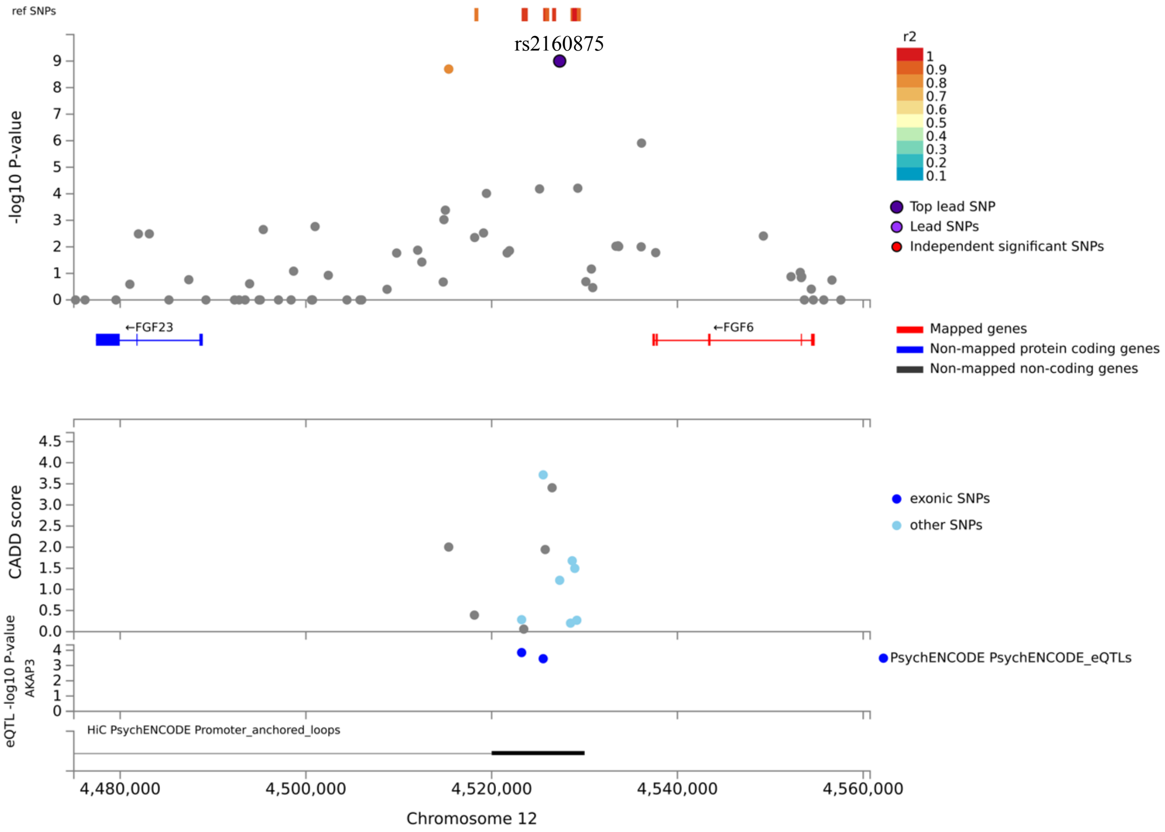

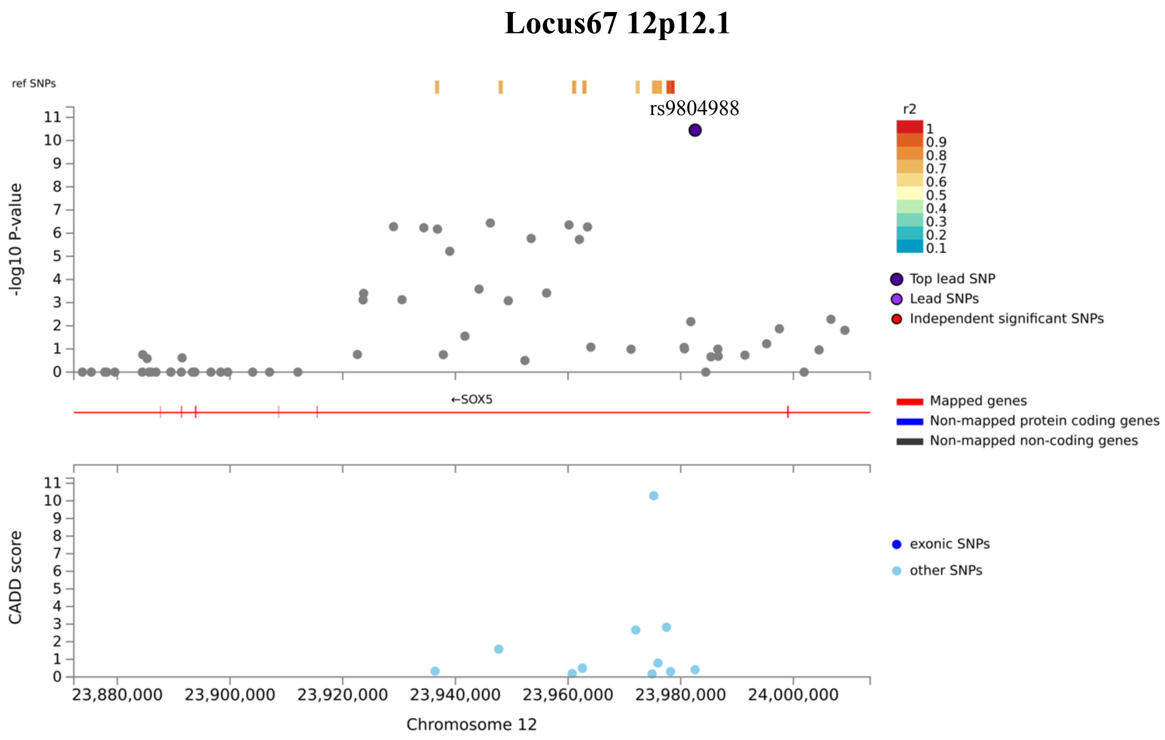

No eQTL of selected tissues exists in this region.

Locus68 12q13.2

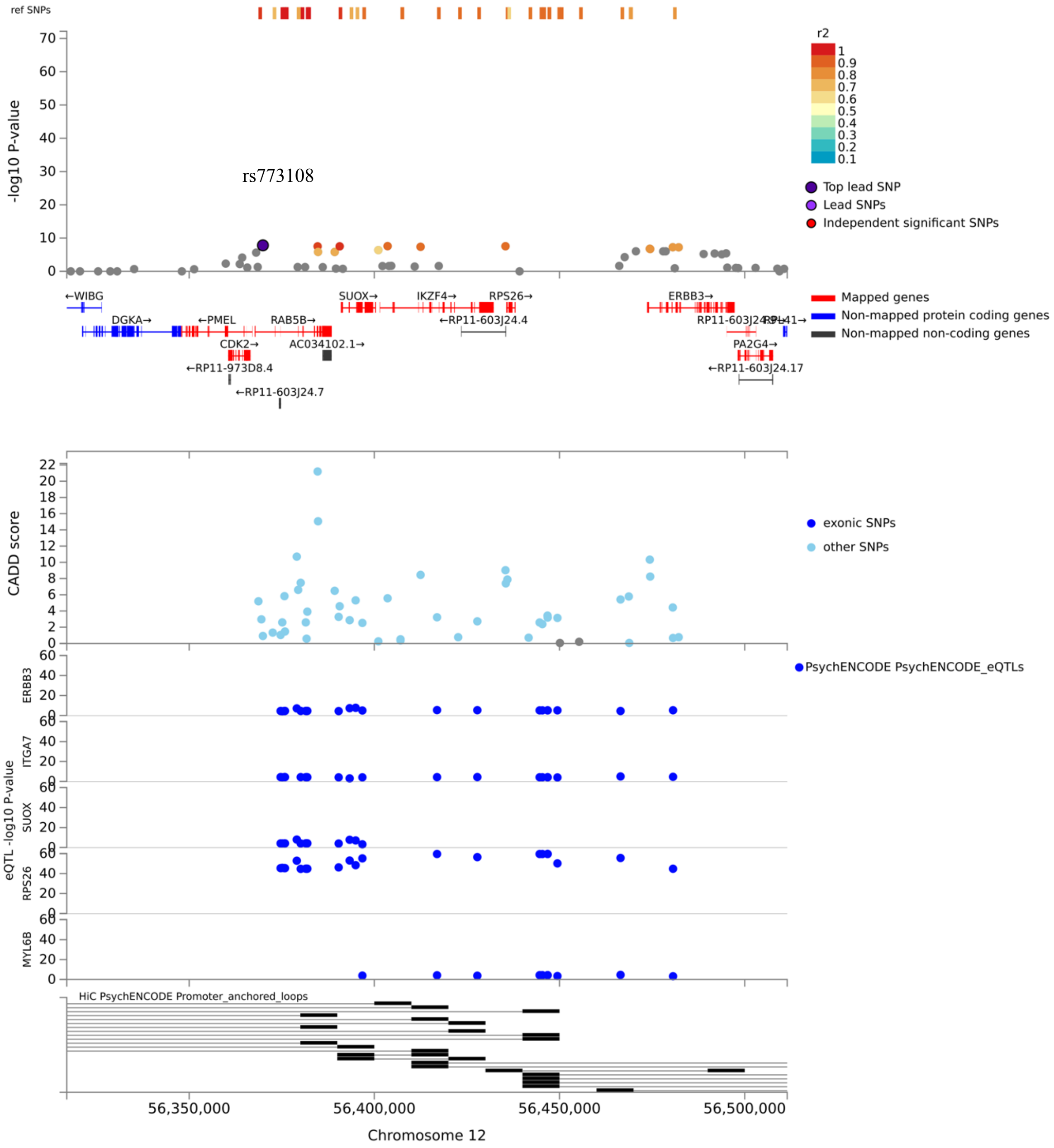

Locus69 12q13.3

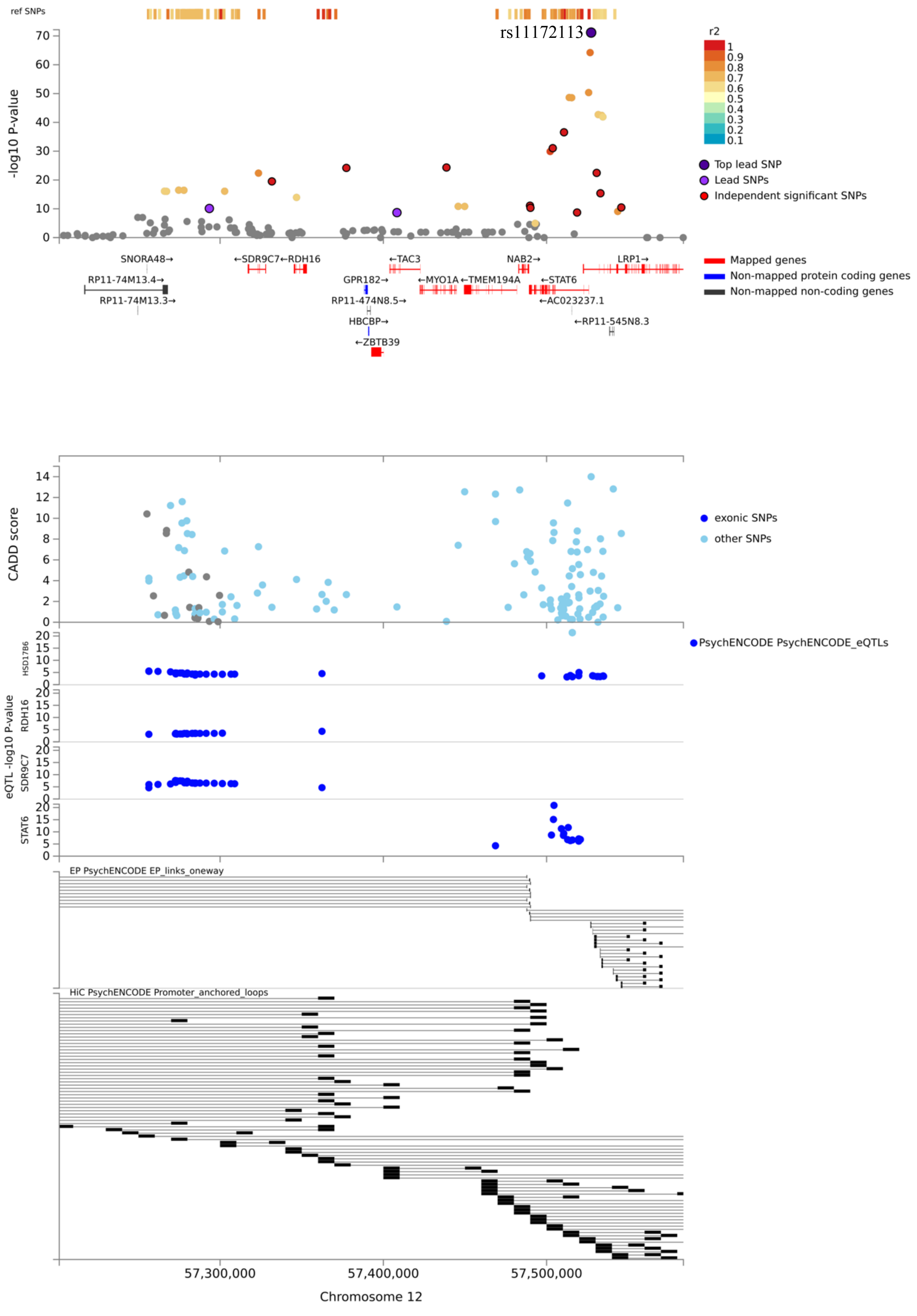

Locus70 12q15

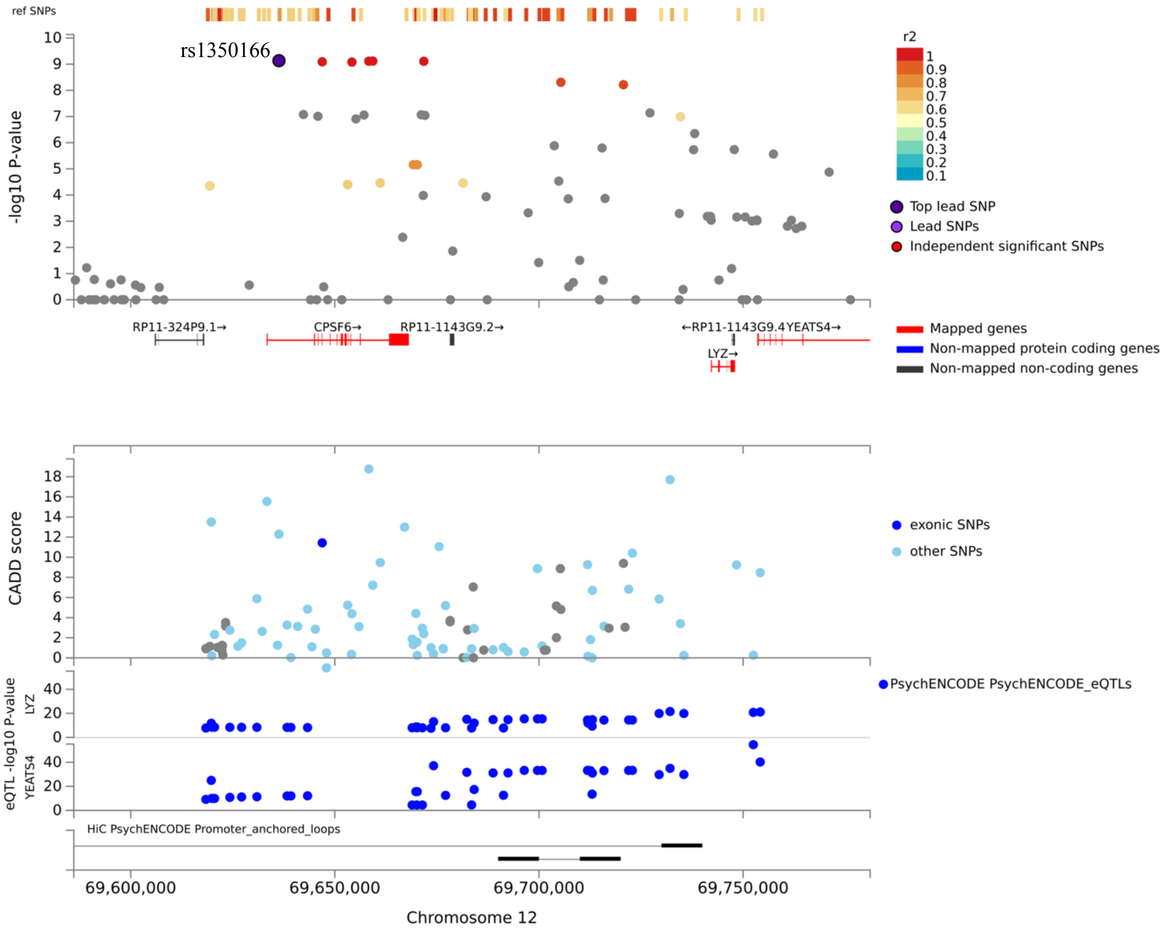

Locus71 12q23.3

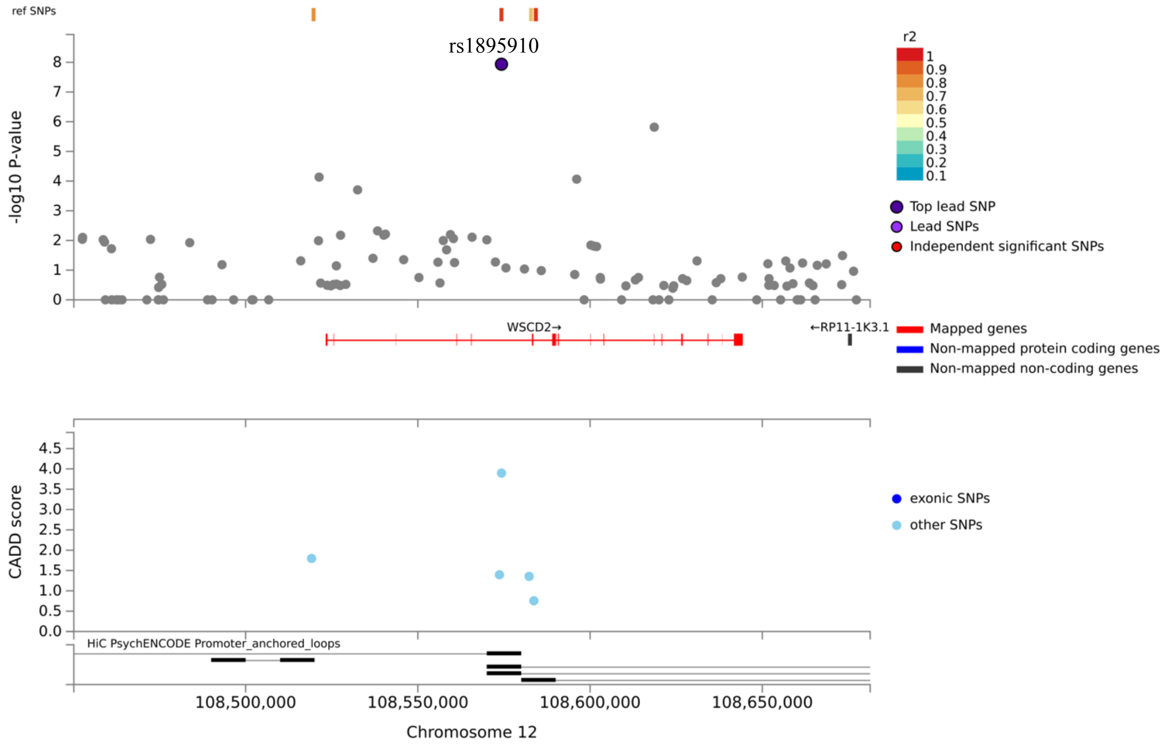

No eQTL of selected tissues exists in this region.

Locus72 12q24.11

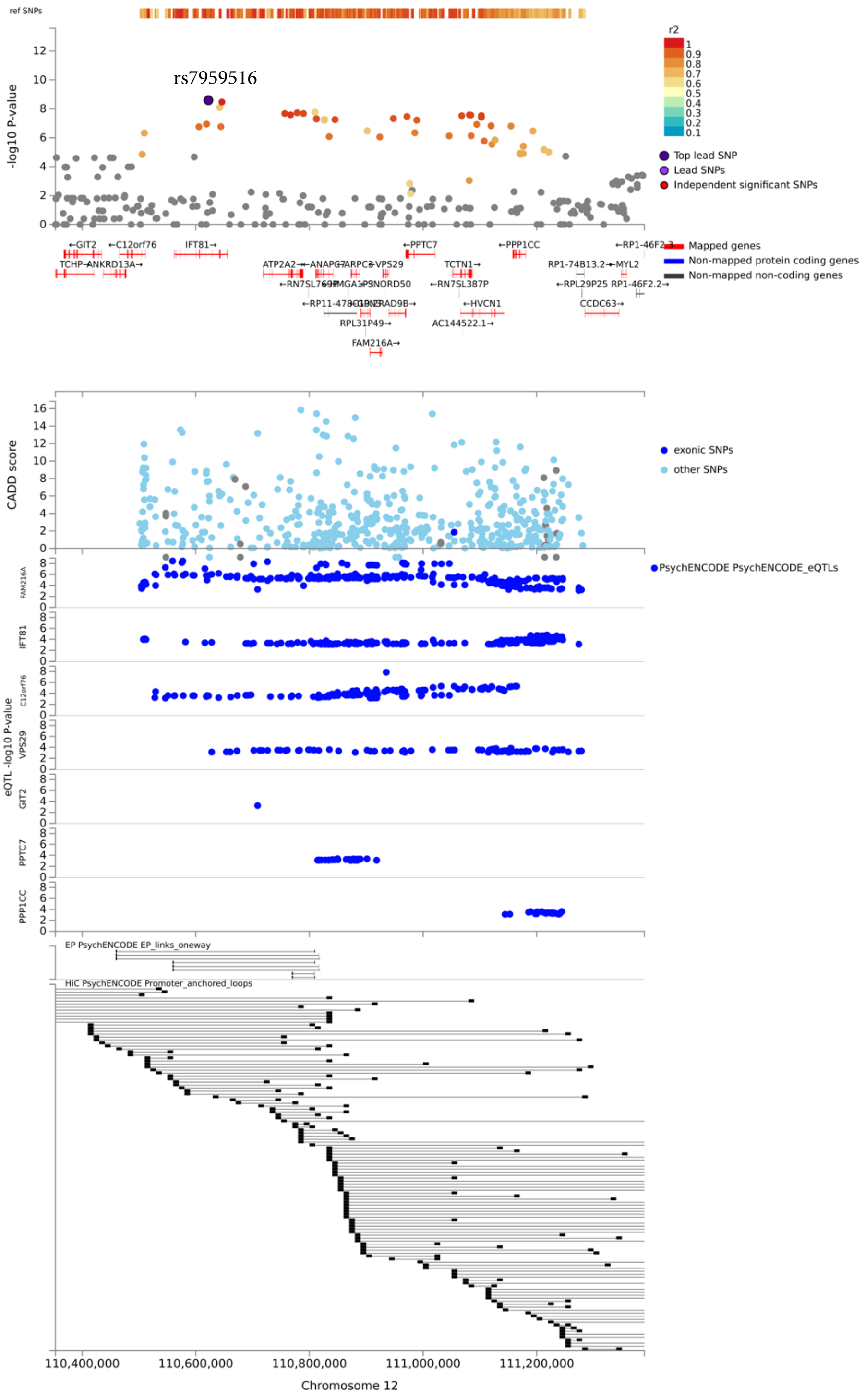

Locus73 12q24.12

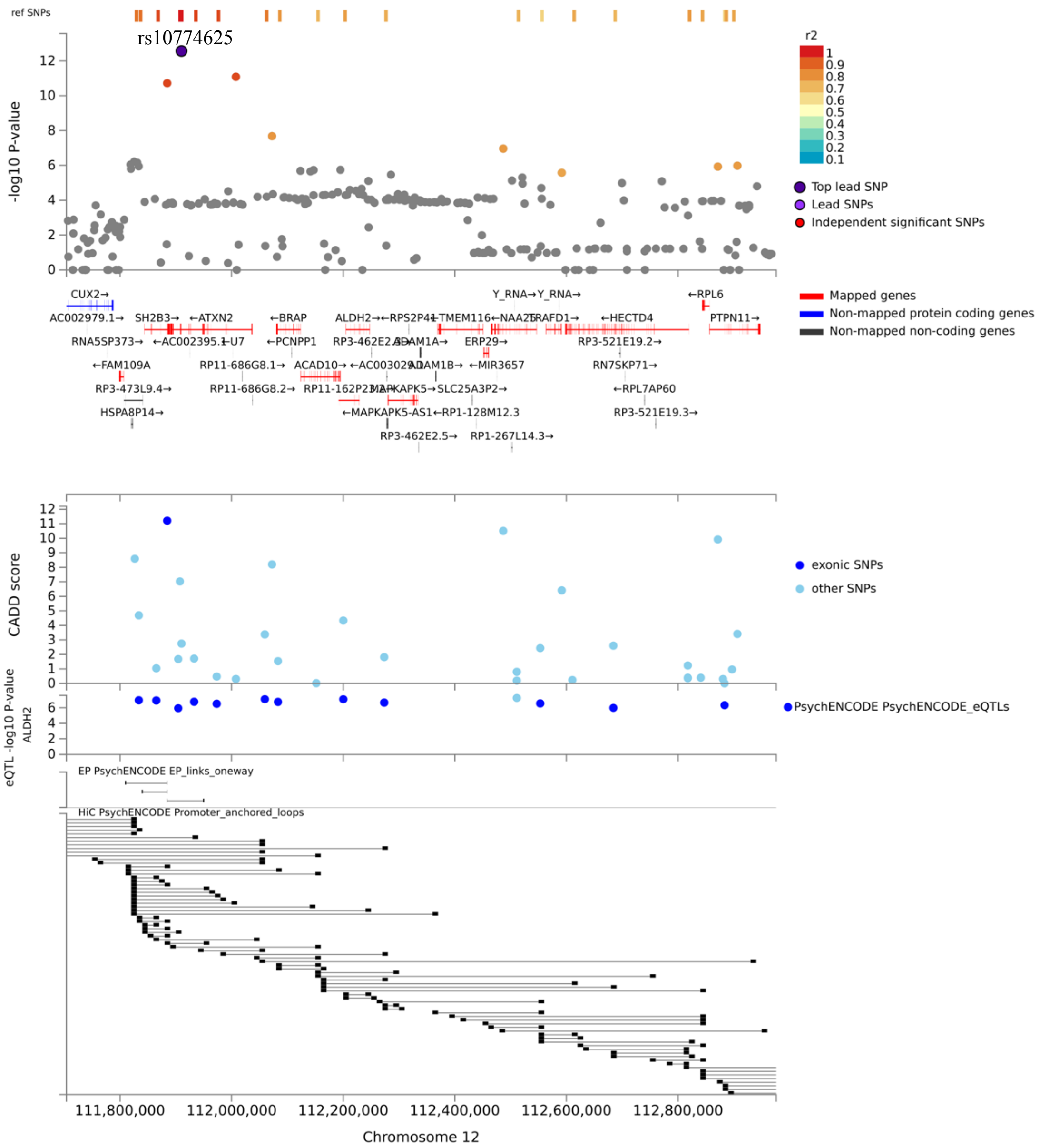

Locus74 13q14.13

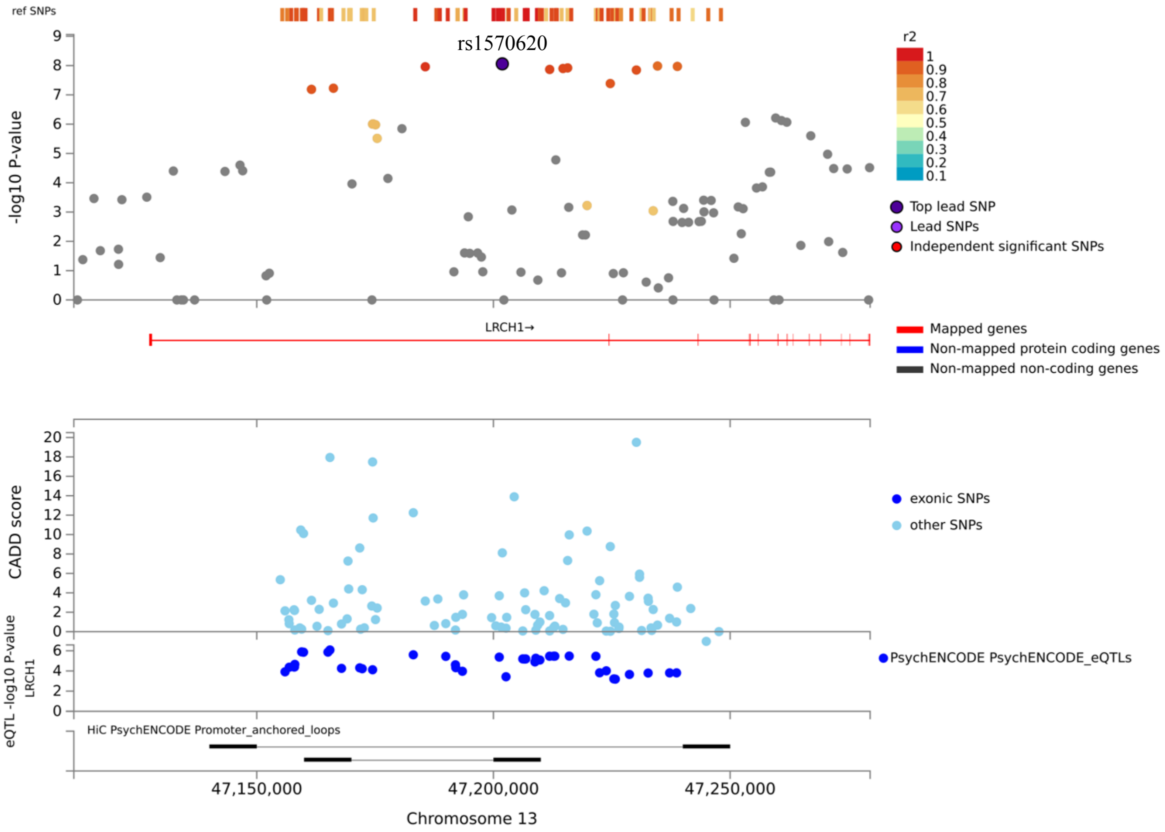

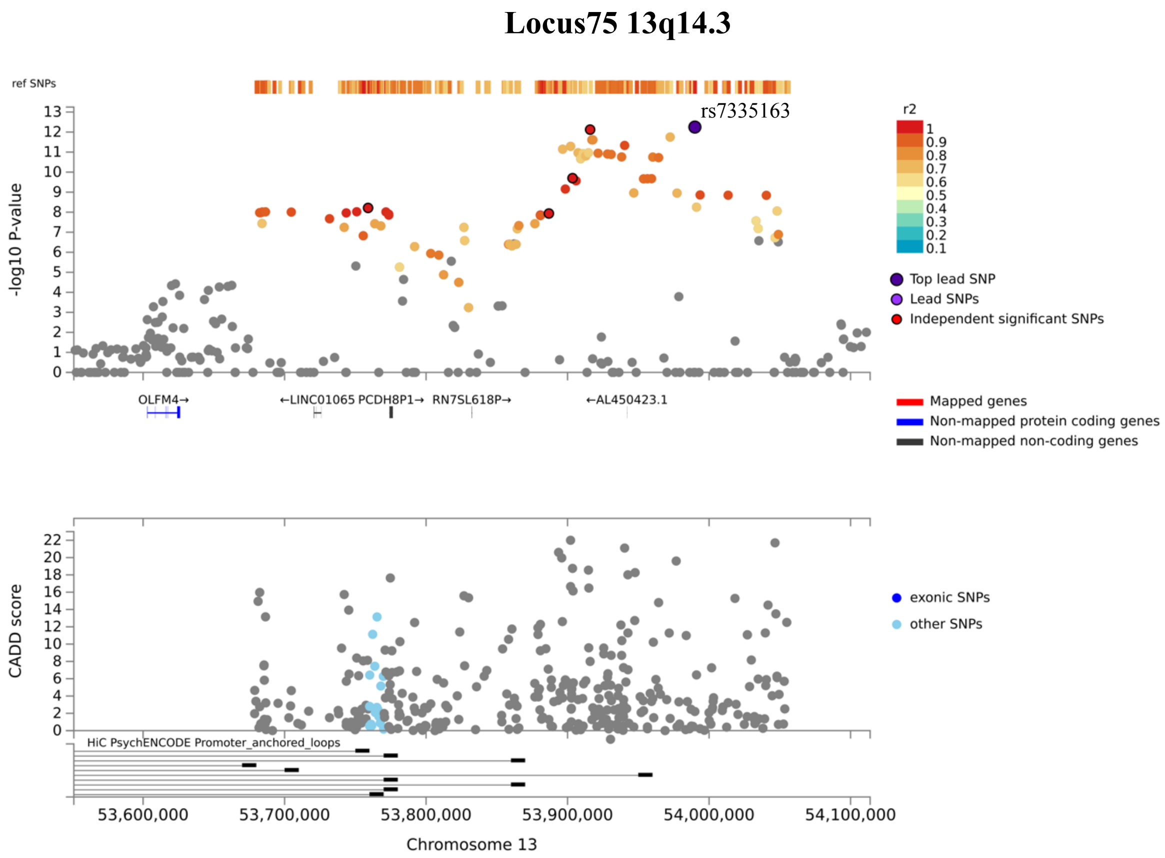

No eQTL of selected tissues exists in this region.

Locus76 13q21.32

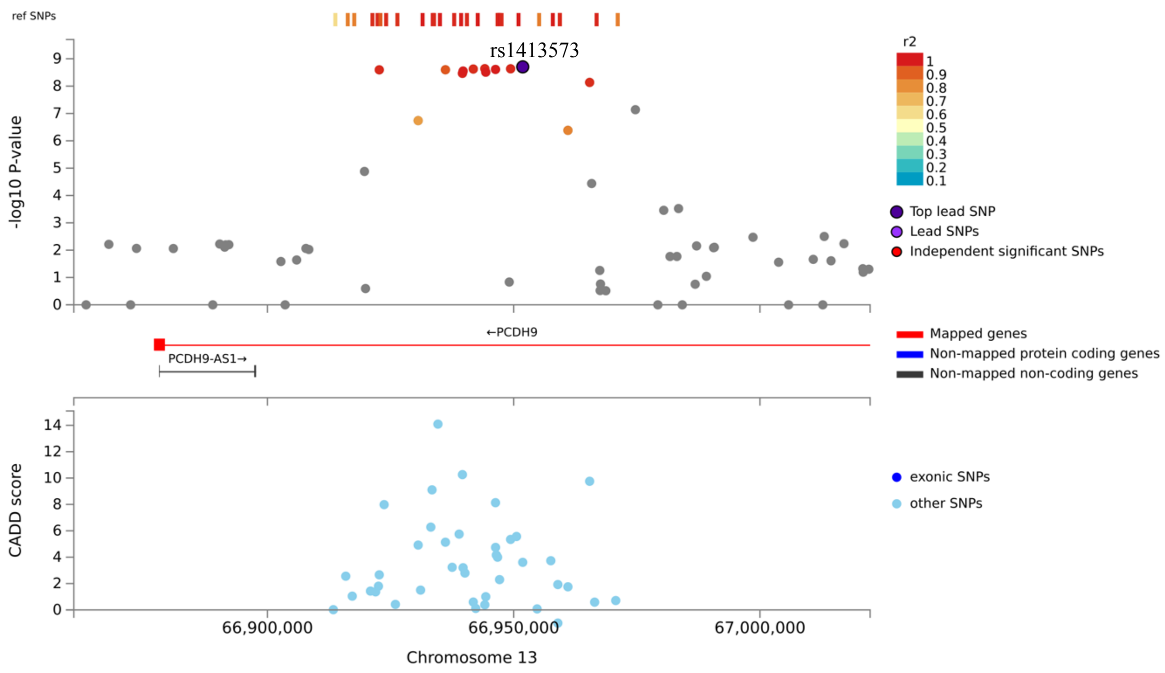

No eQTL of selected tissues exists in this region.

Locus77 14q12

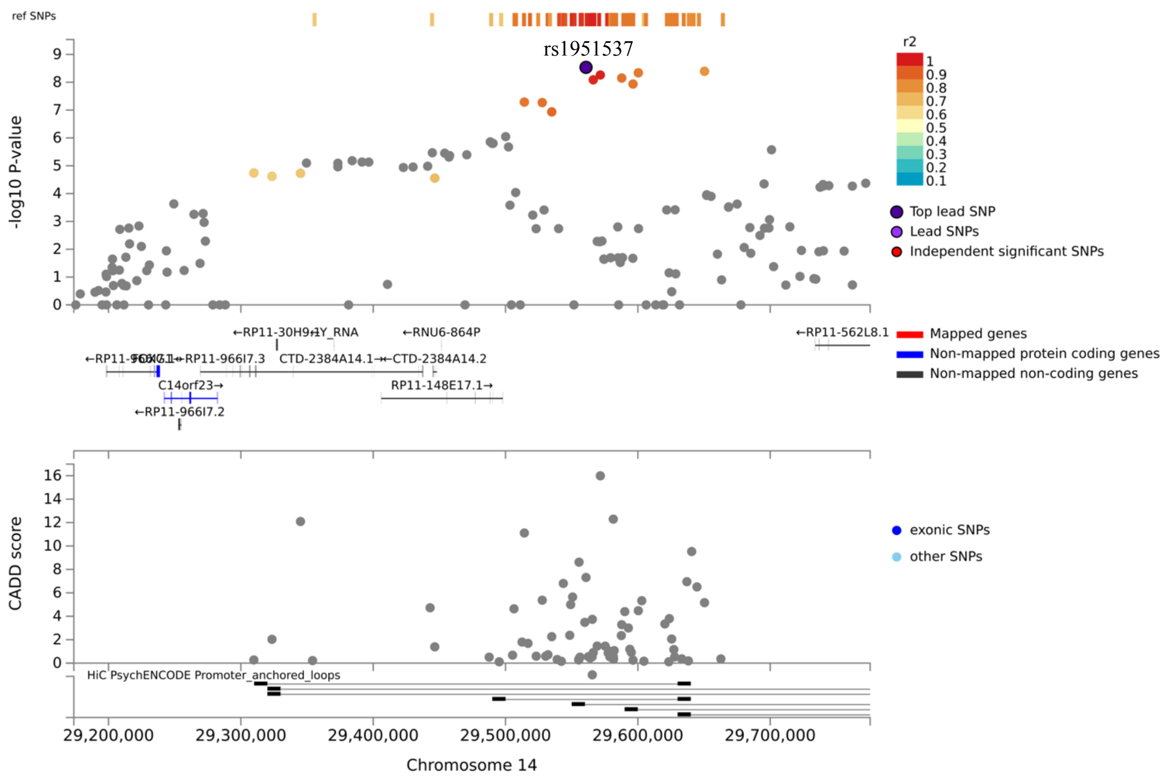

No eQTL of selected tissues exists in this region.

Locus78 14q13.3

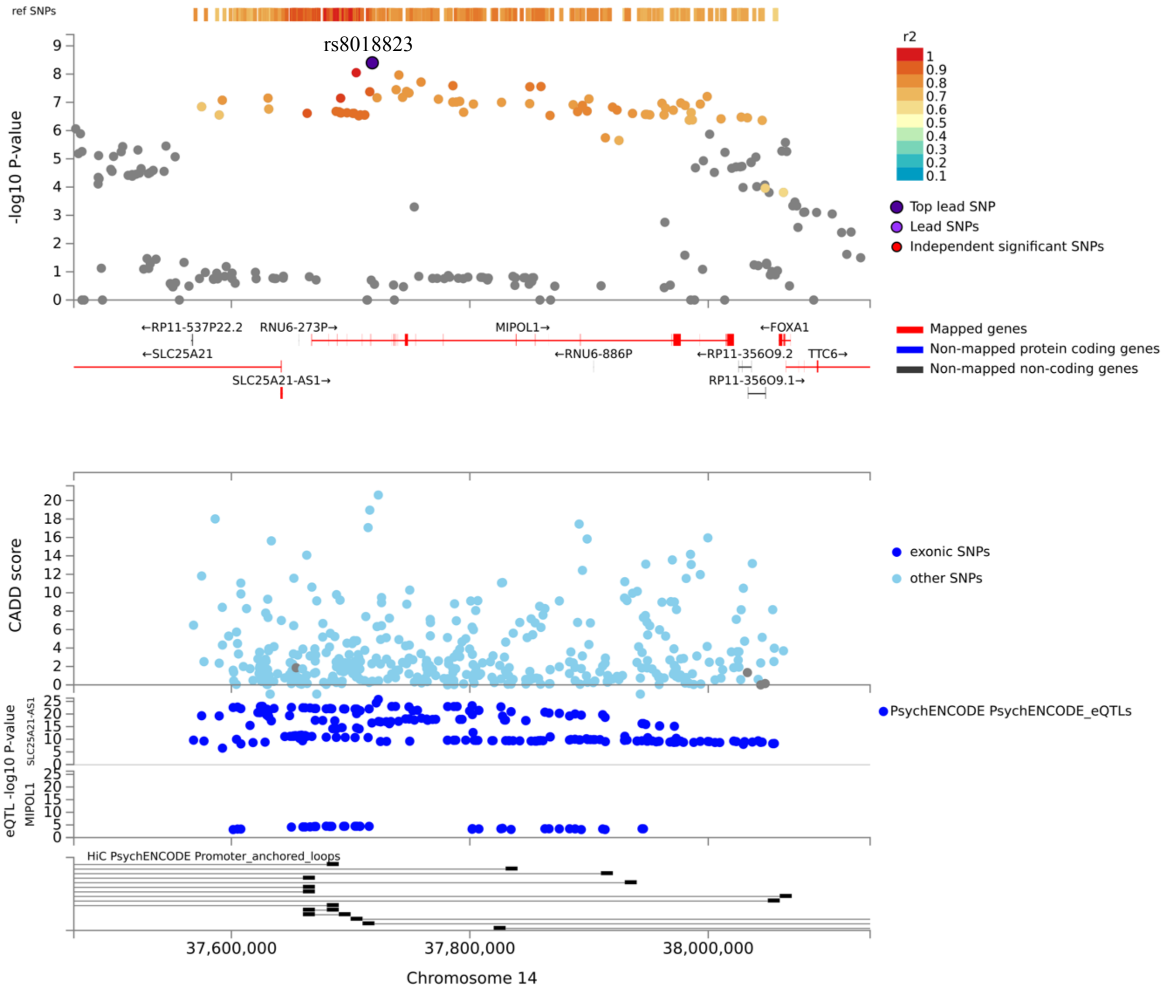

Locus79 14q32.12

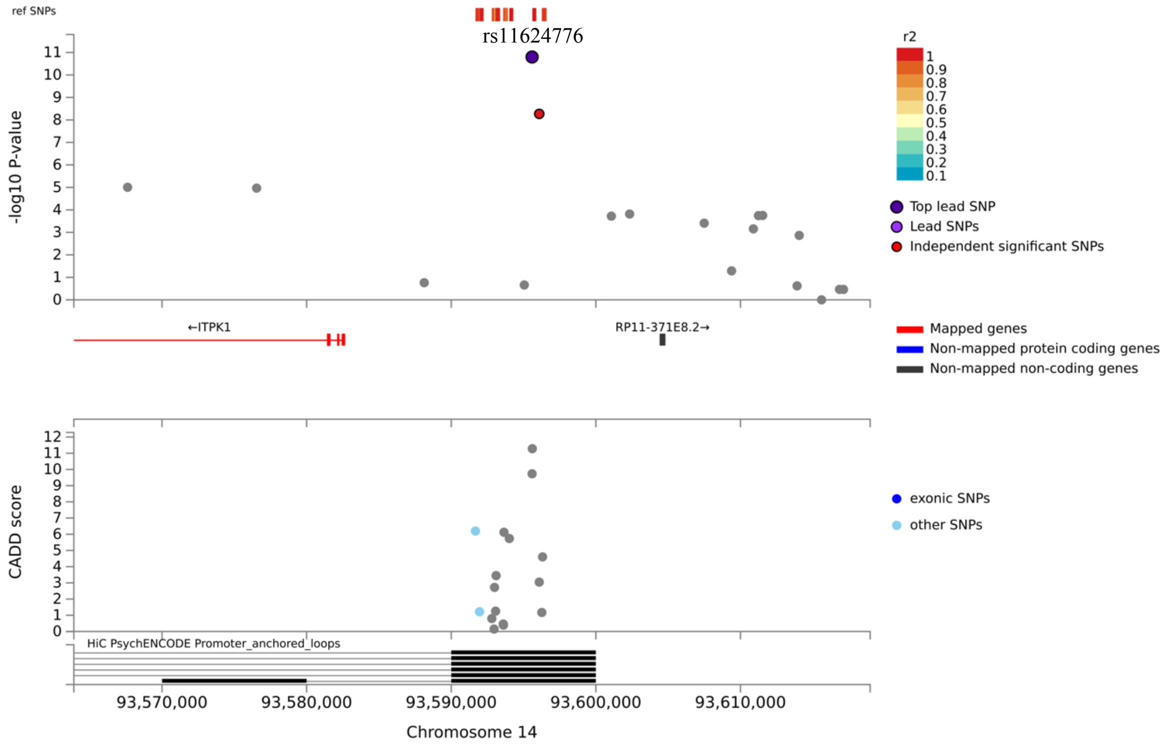

No eQTL of selected tissues exists in this region.

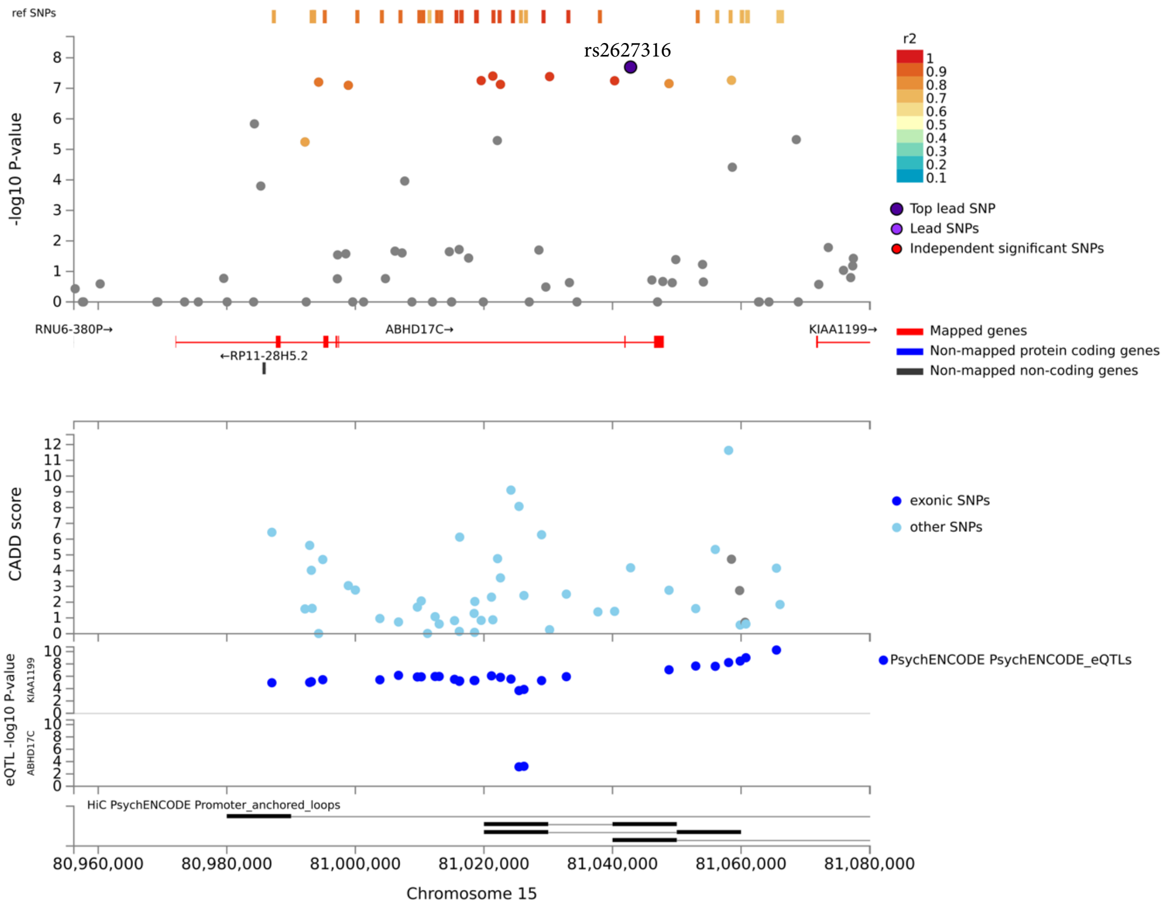
ref SNPs
rs2627316
-log10 P-value
r2
1
0.9
0.8
0.7
0.6
0.5
0.4
0.3
0.2
0.1
Top lead SNP
Lead SNPs
Independent significant SNPs
RNU6-380P→
ABHD17C→
KIAA1199→
←RP11-28H5.2
Mapped genes
Non-mapped protein coding genes
Non-mapped non-coding genes
CADD score
exonic SNPs
other SNPs
PsychENCODE PsychENCODE_eQTLs
eQTL -log10 P-value
KIAA1199
ABHD17C
HiC PsychENCODE Promoter_anchored_loops
80,960,000
80,980,000
81,000,000
81,020,000
81,040,000
81,060,000
81,080,000
Chromosome 15

Locus81 15q26.1

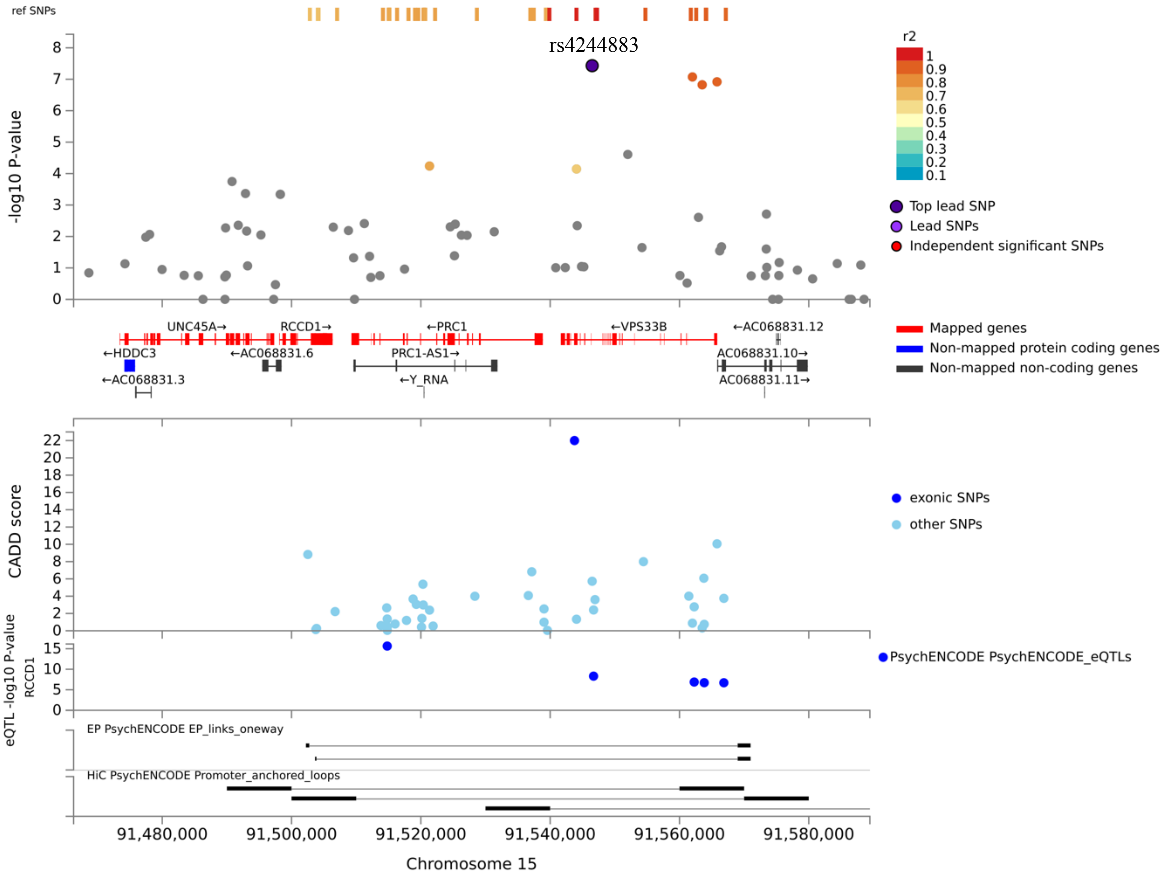

Locus82 16q22.2

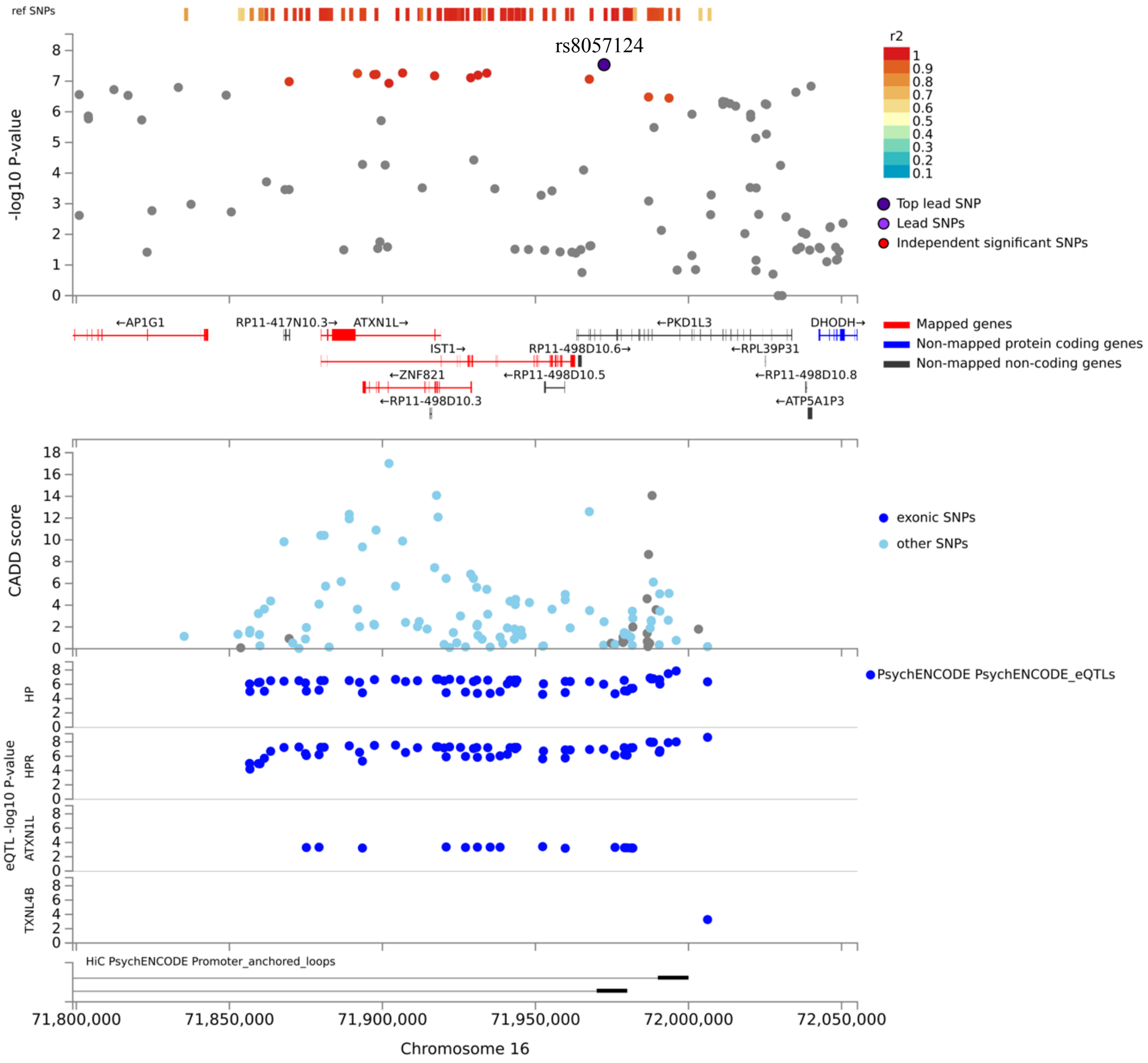

Locus83 16q23.1

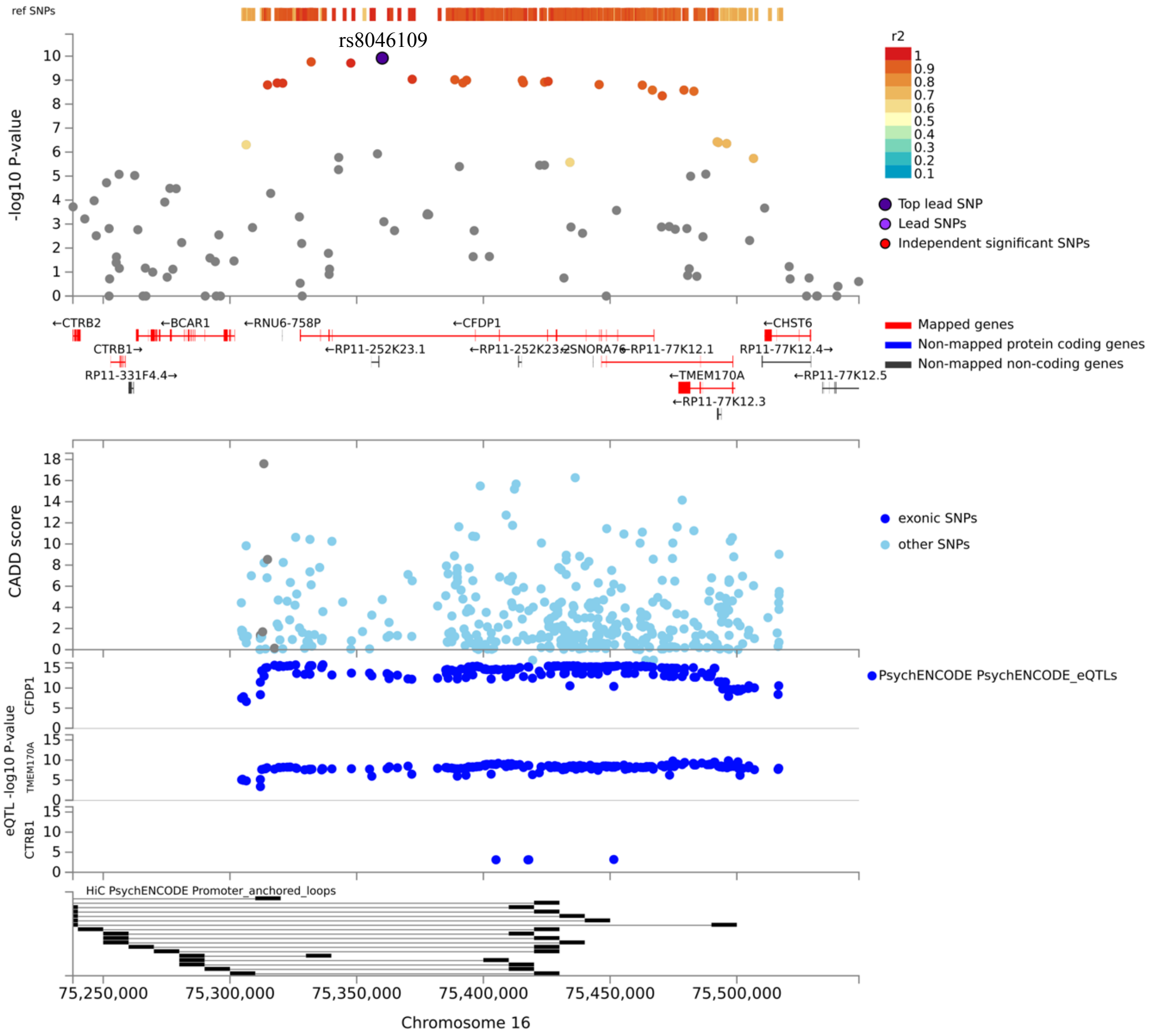

Locus84 16q24.2

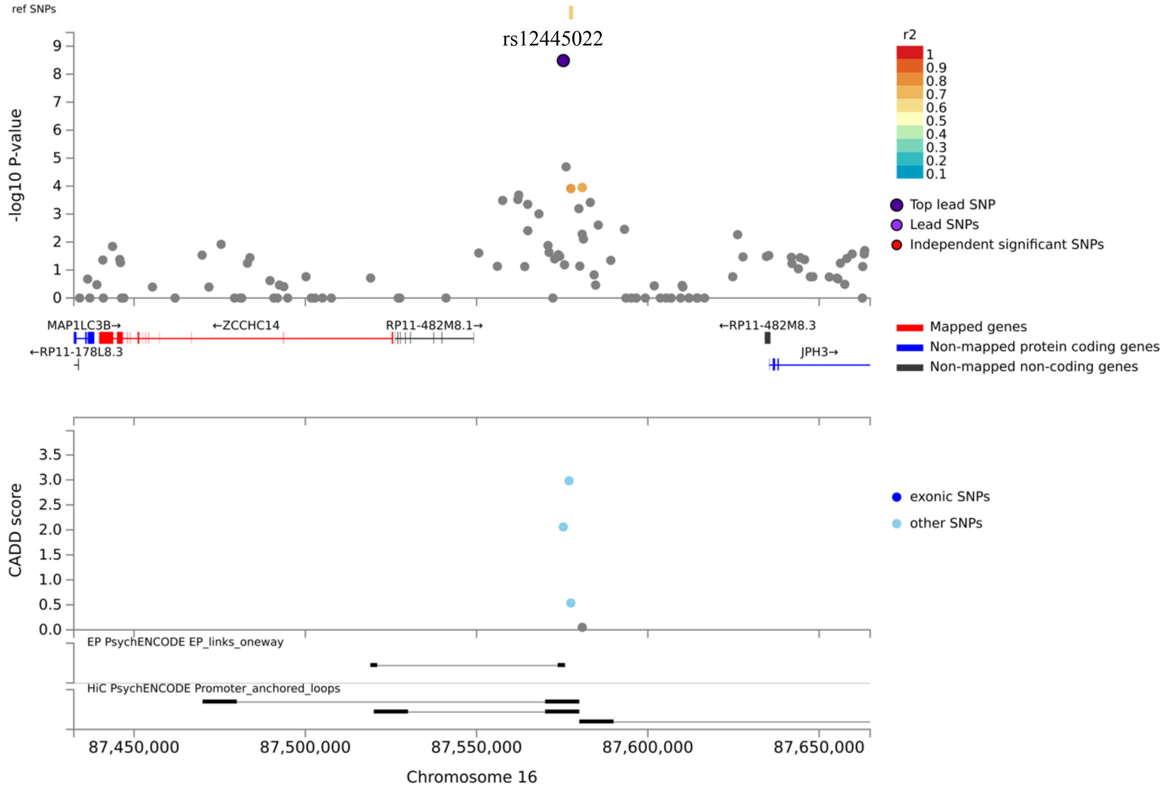

No eQTL of selected tissues exists in this region.

Locus85 16q24.3

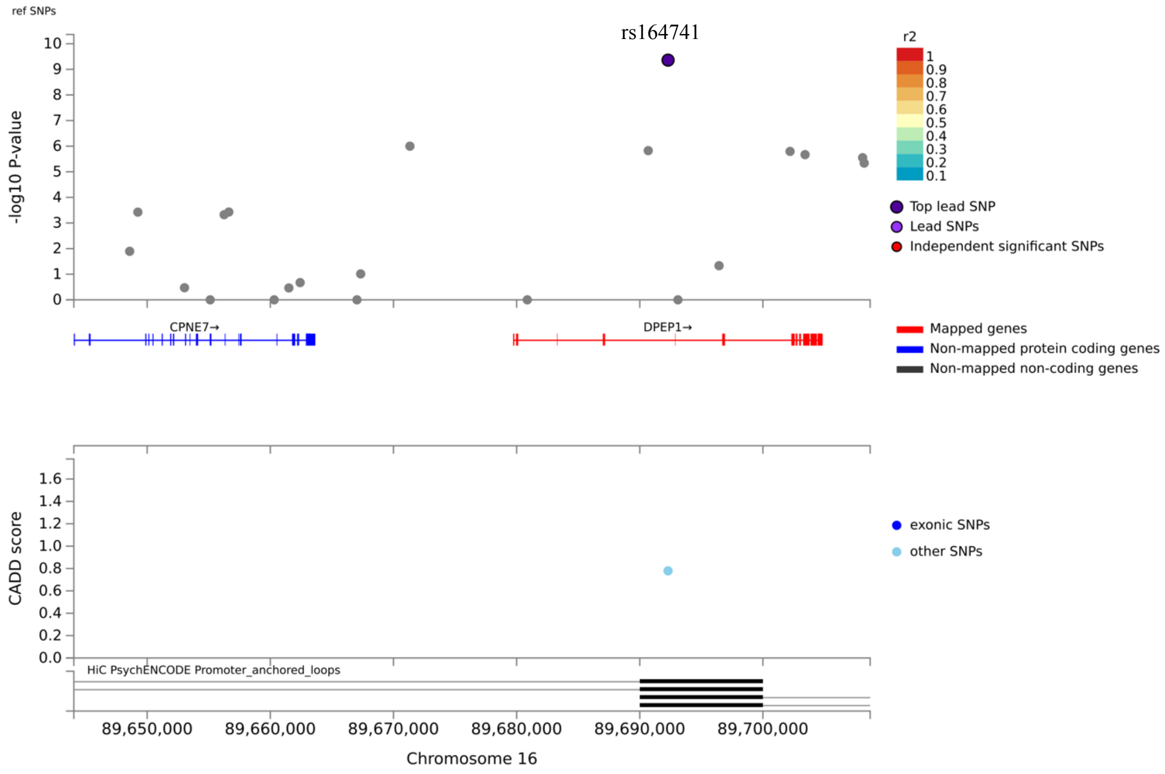

No eQTL of selected tissues exists in this region.

Locus86 17q11.2

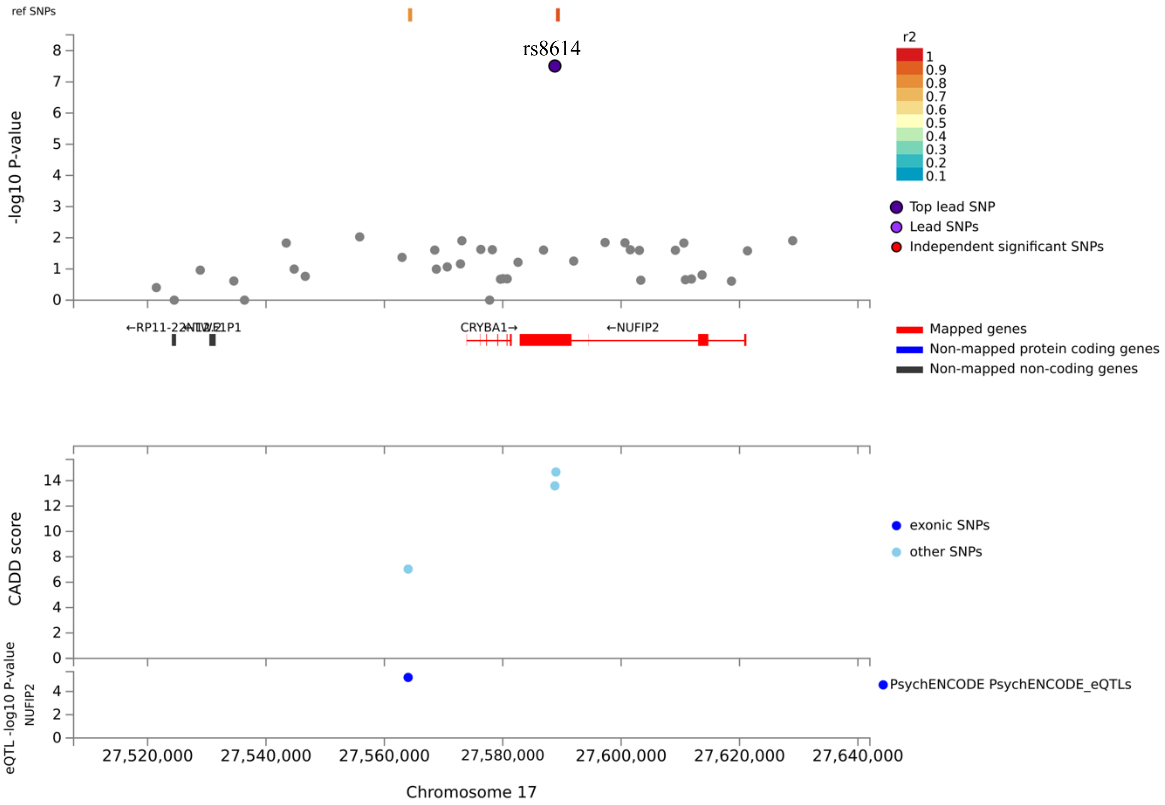

Locus87 17q21.31

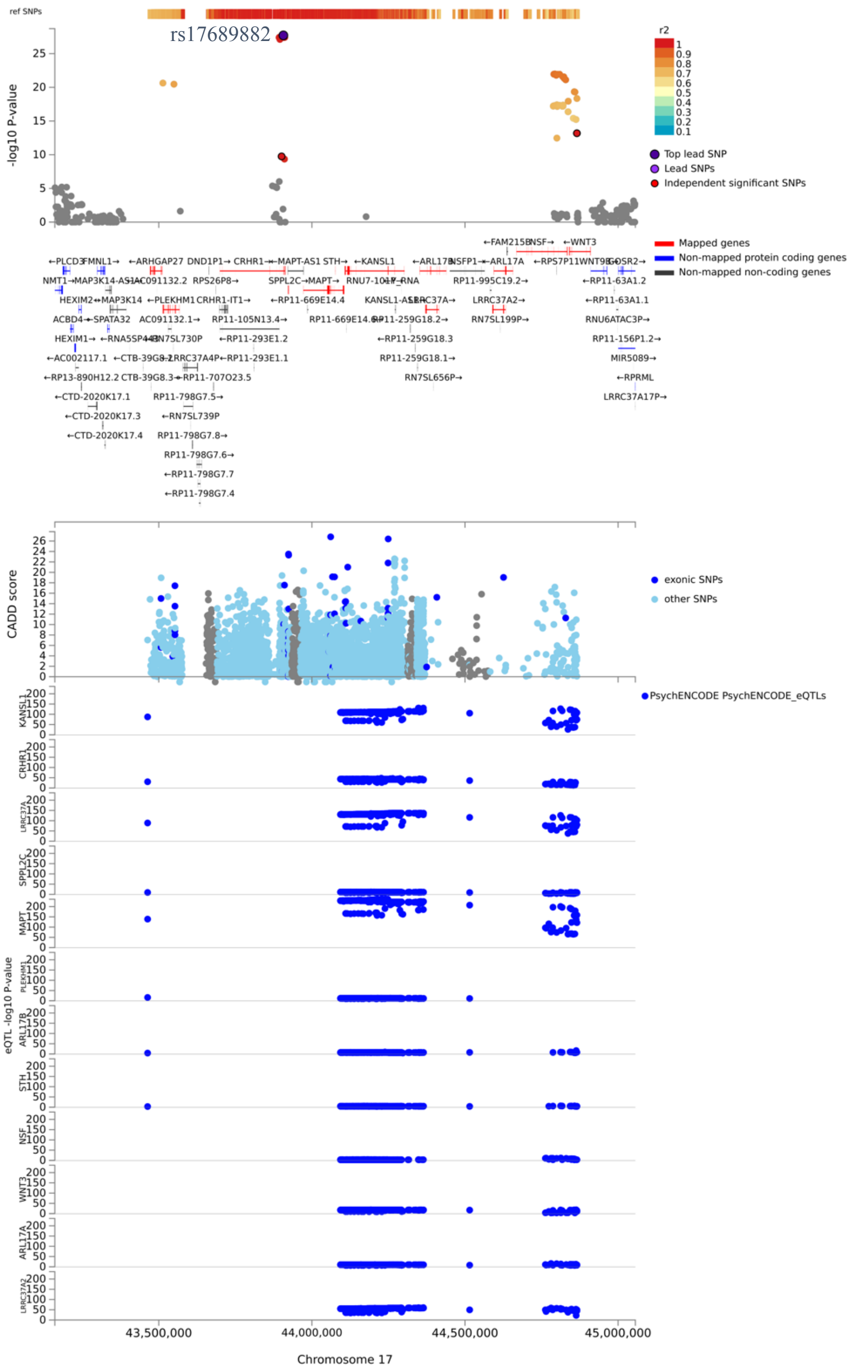

Locus88 17q21.33

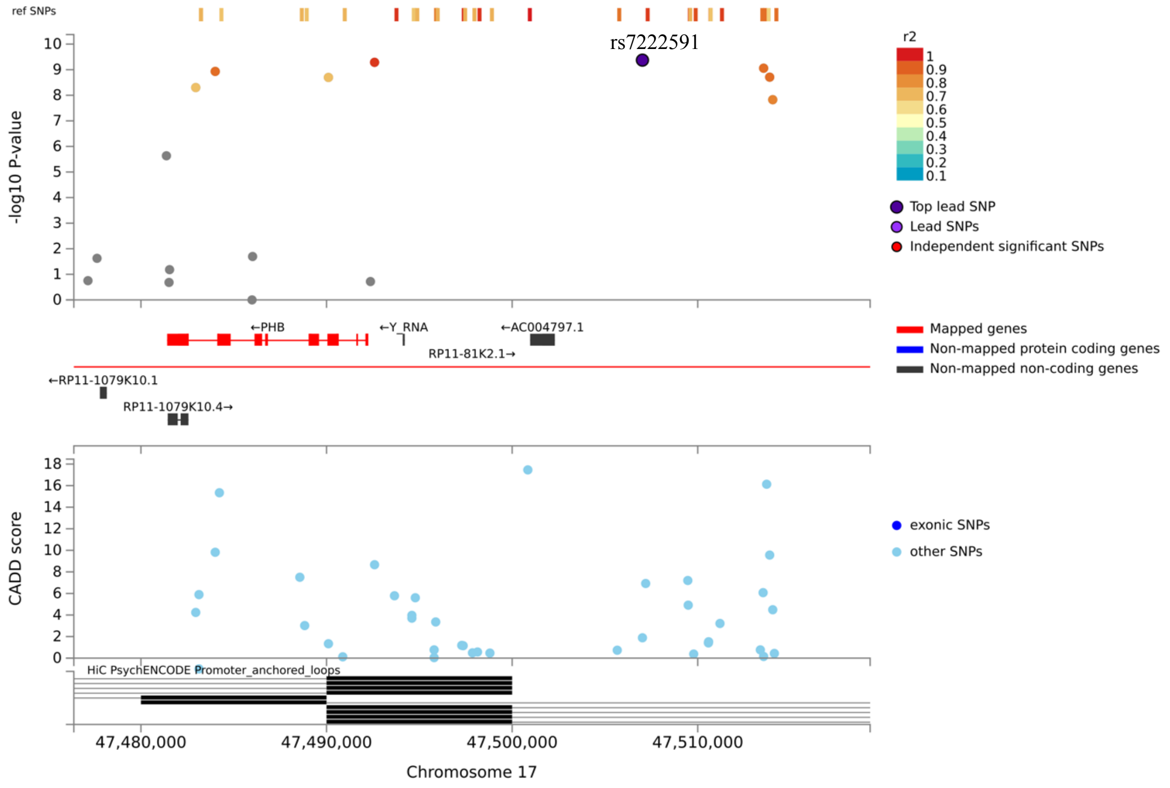

No eQTL of selected tissues exists in this region.

Locus89 17q22

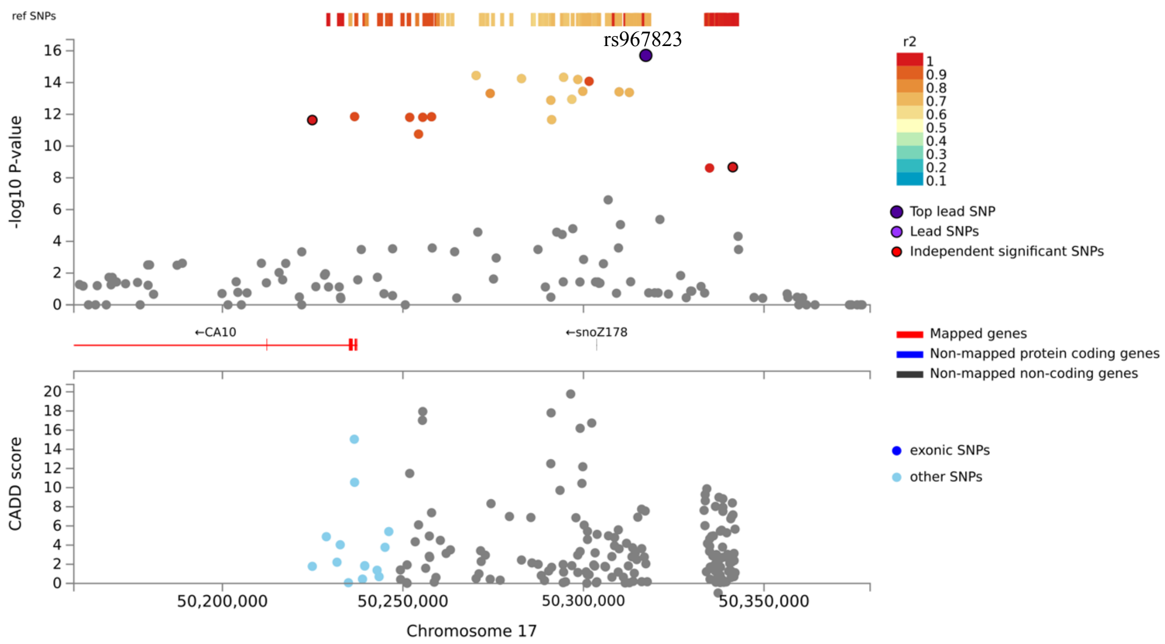

No eQTL of selected tissues exists in this region.

Locus90 18q11.2

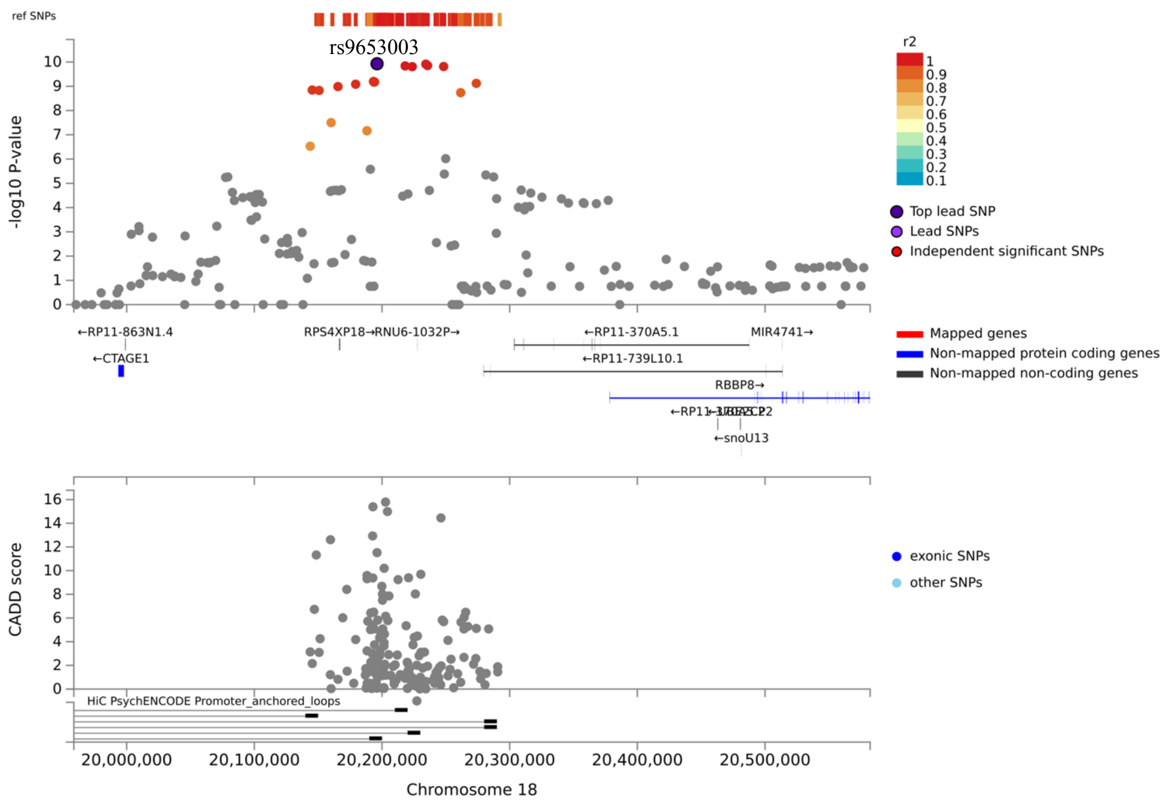

No eQTL of selected tissues exists in this region.

Locus91 18q12.2

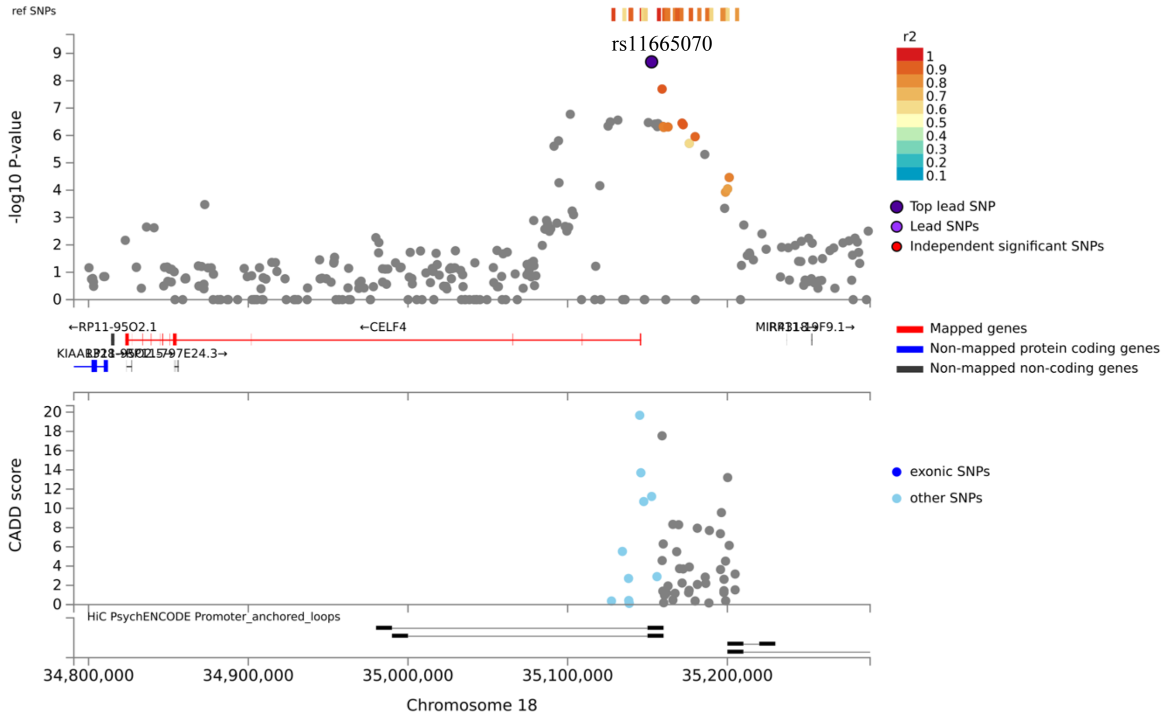

No eQTL of selected tissues exists in this region.

Locus92 18q21.2

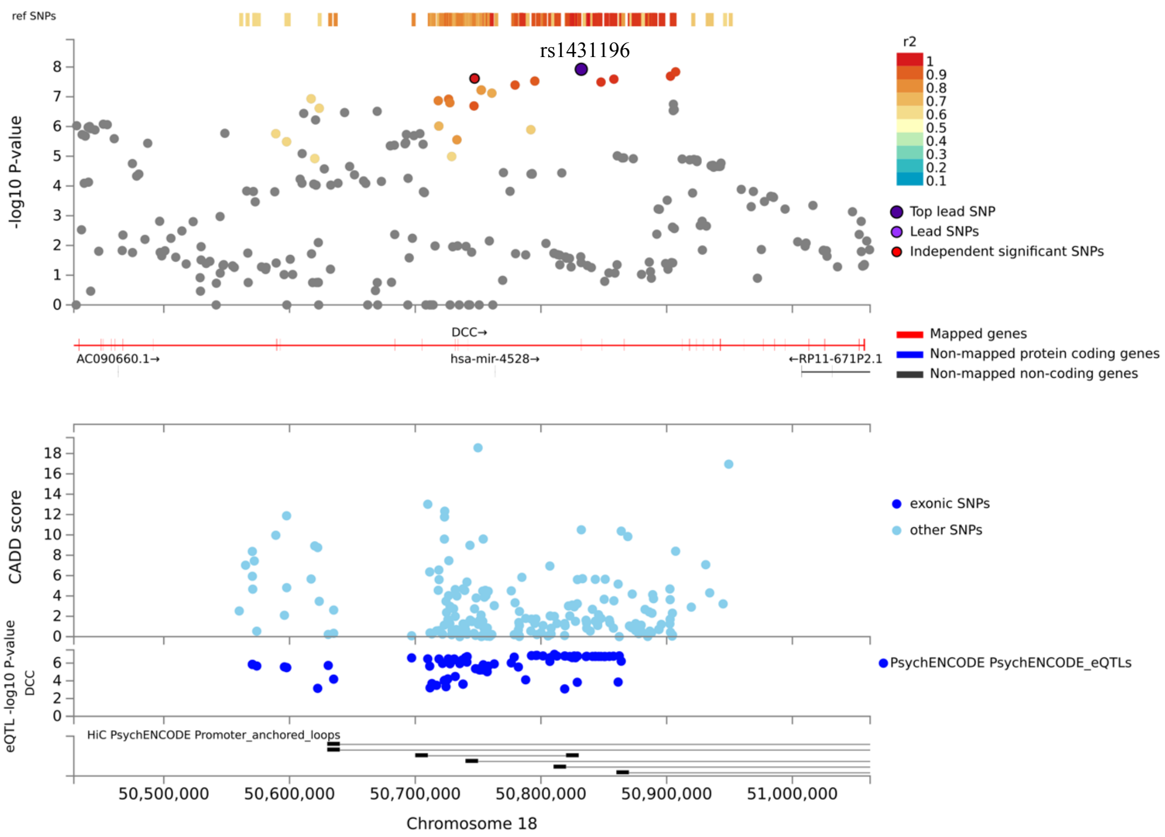

Locus93 18q21.2

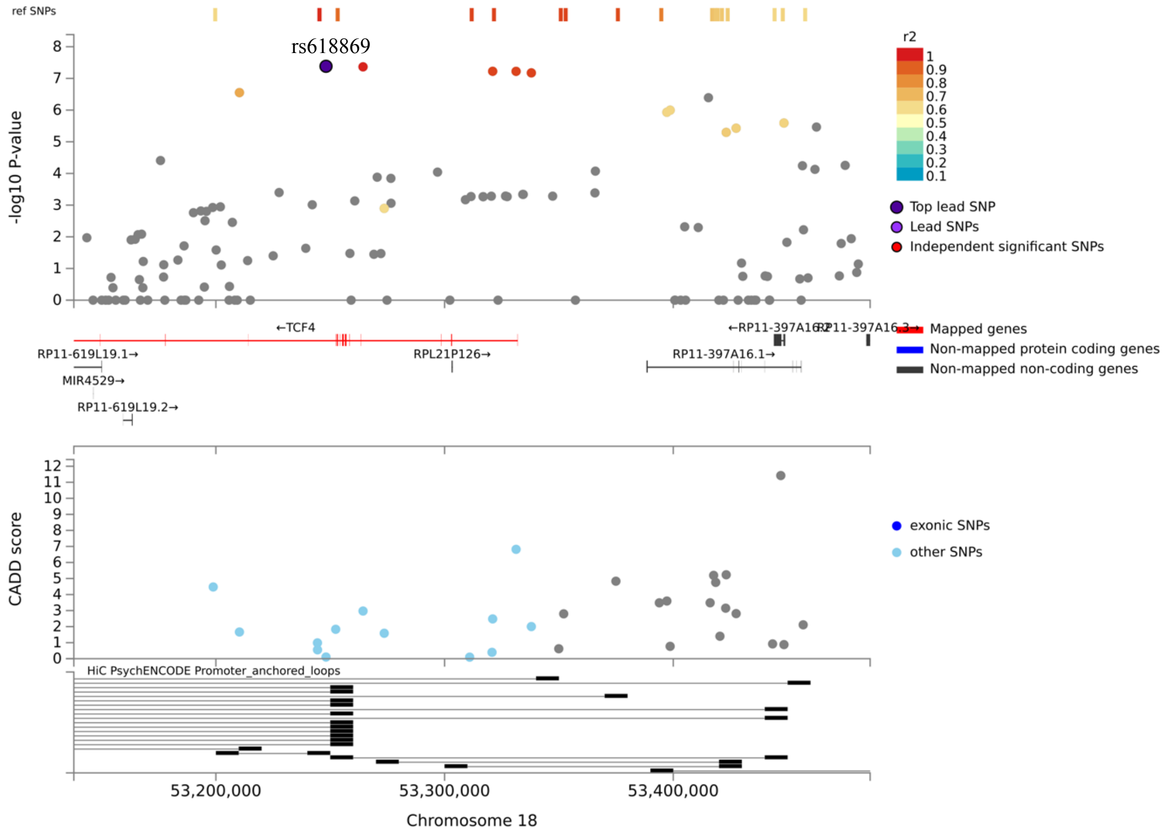

No eQTL of selected tissues exists in this region.

Locus94 18q21.31

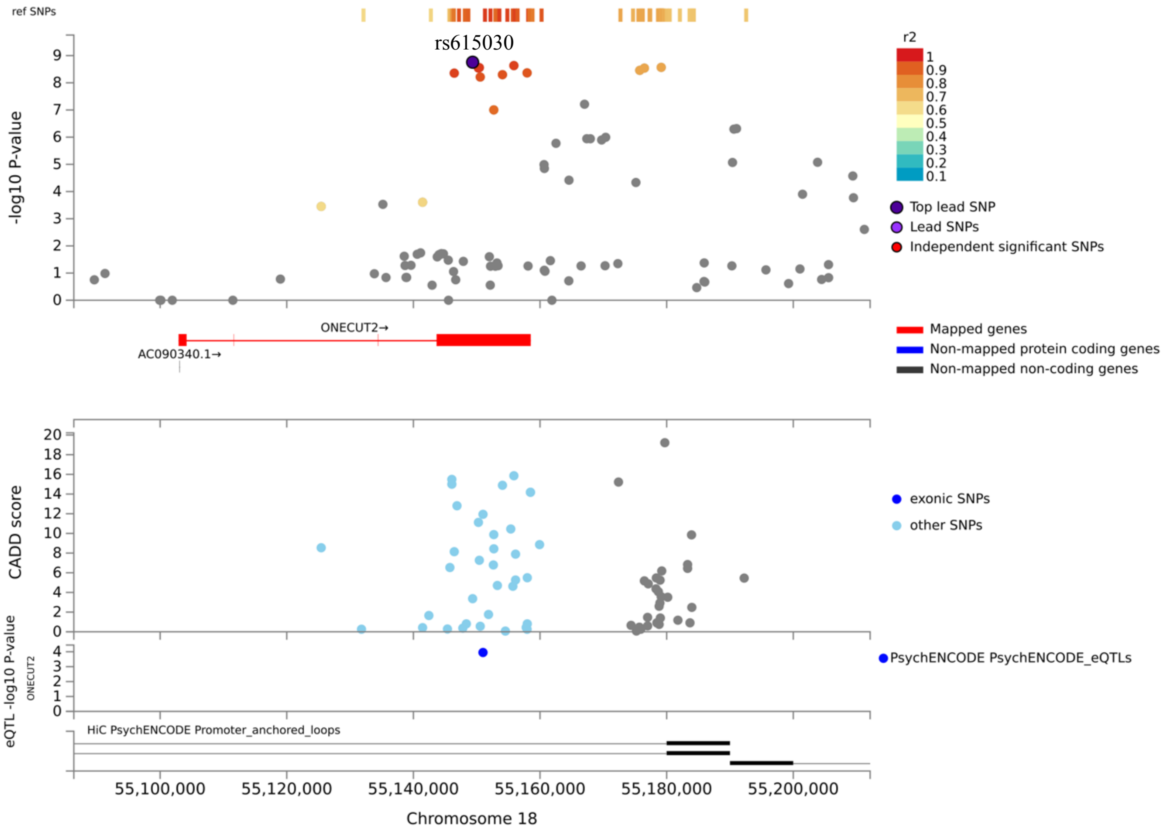

Locus95 19q13.32

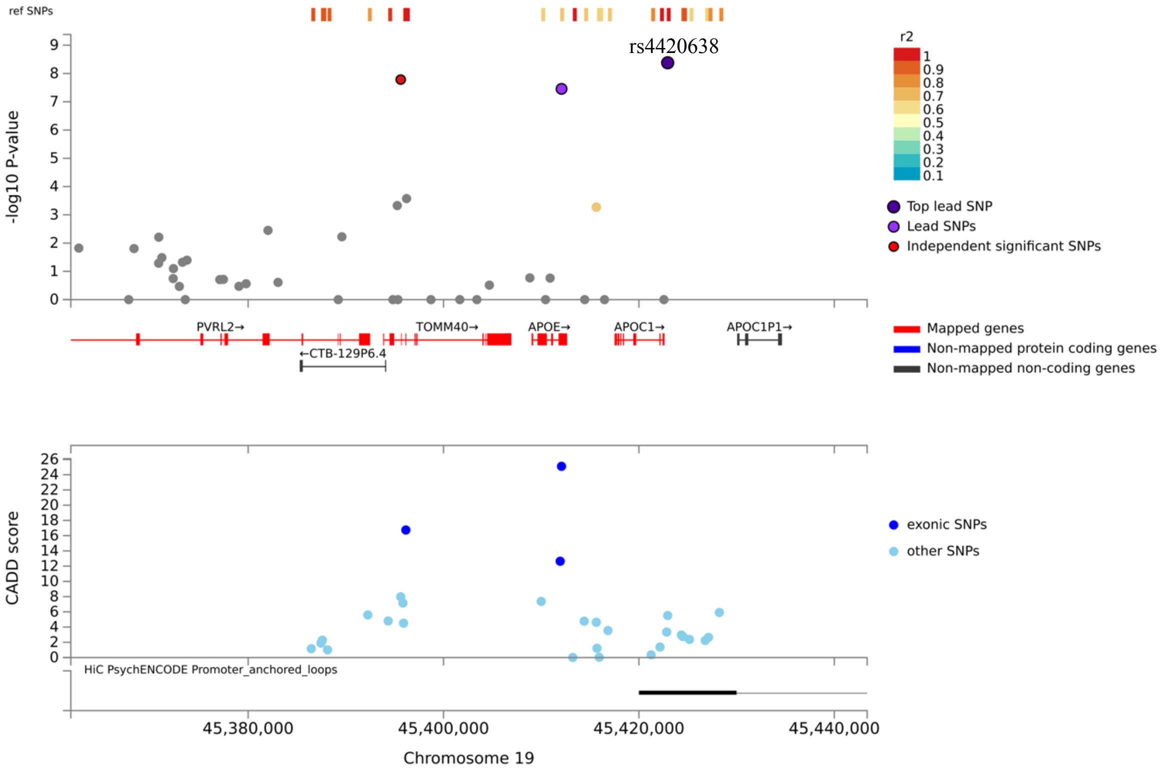

No eQTL of selected tissues exists in this region.

Locus96 20p11.23

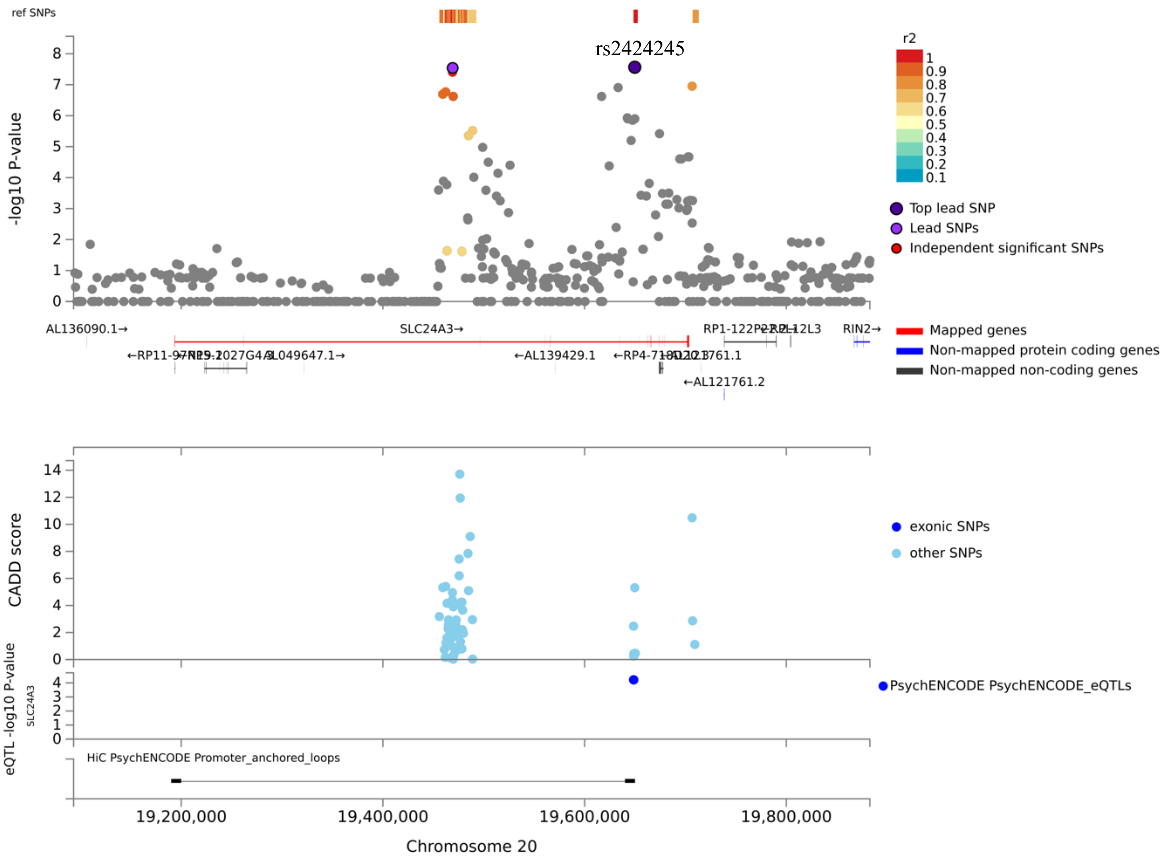

Locus97 20q11.21

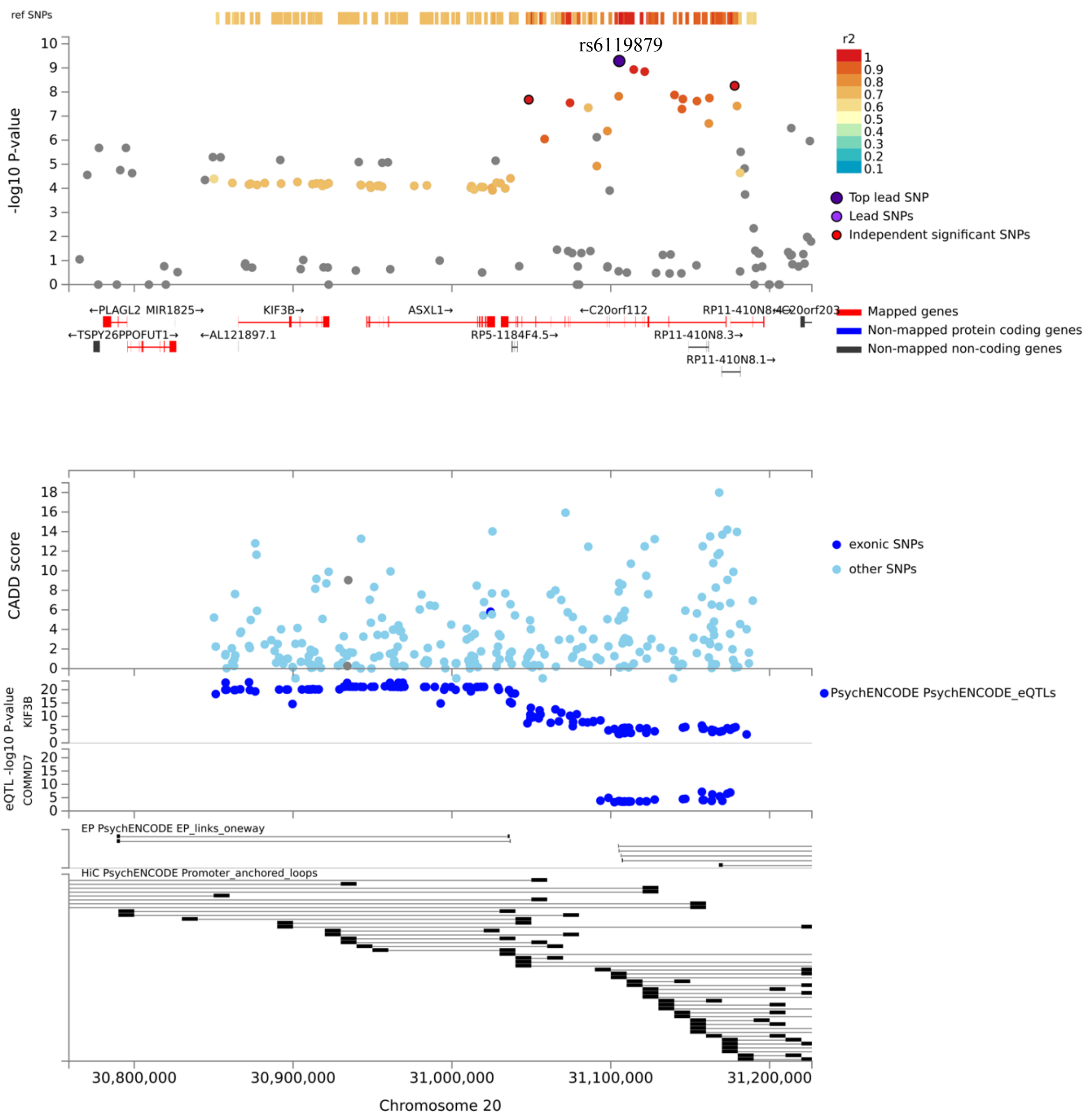

Locus98 20q11.22

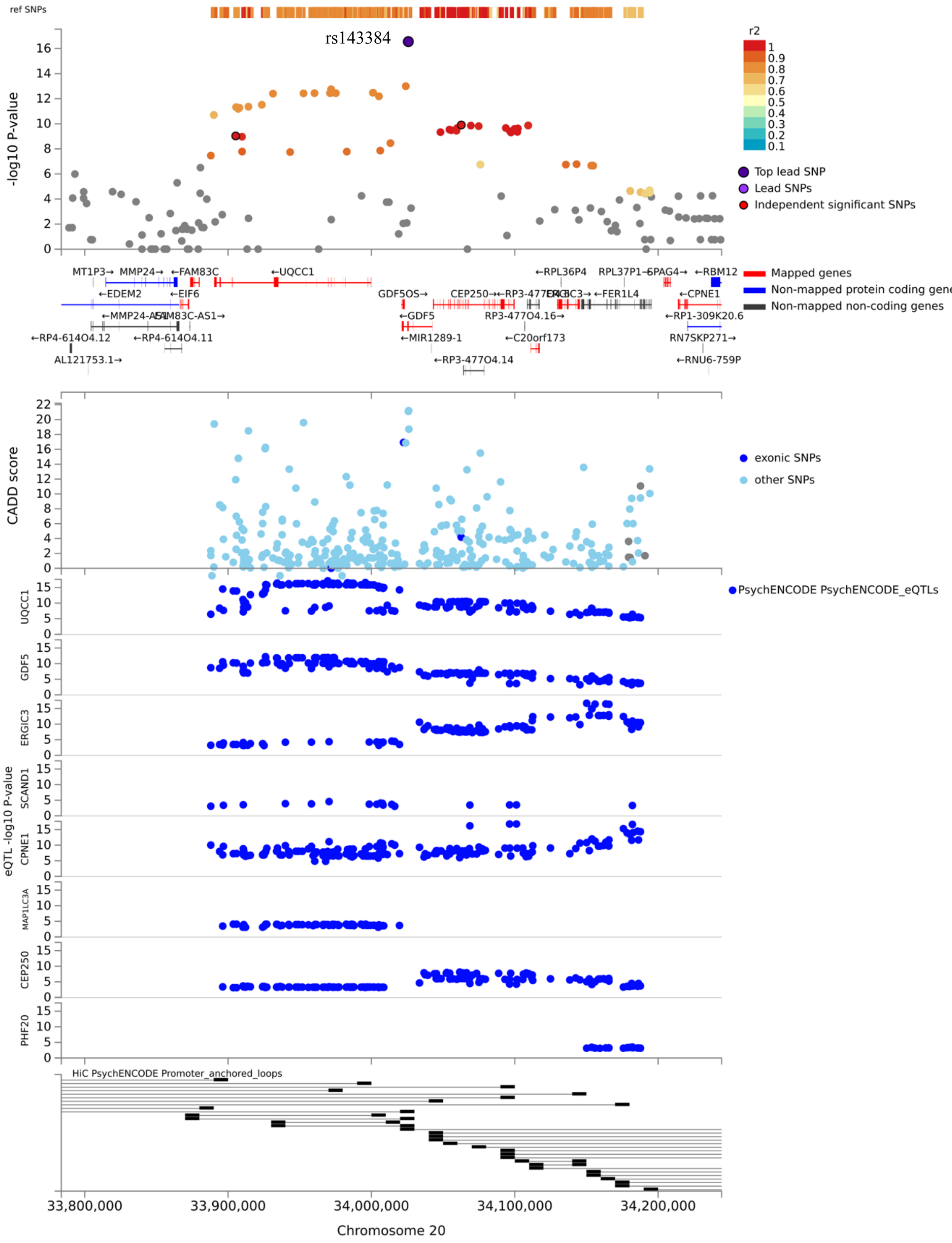

Locus99 22q12.2

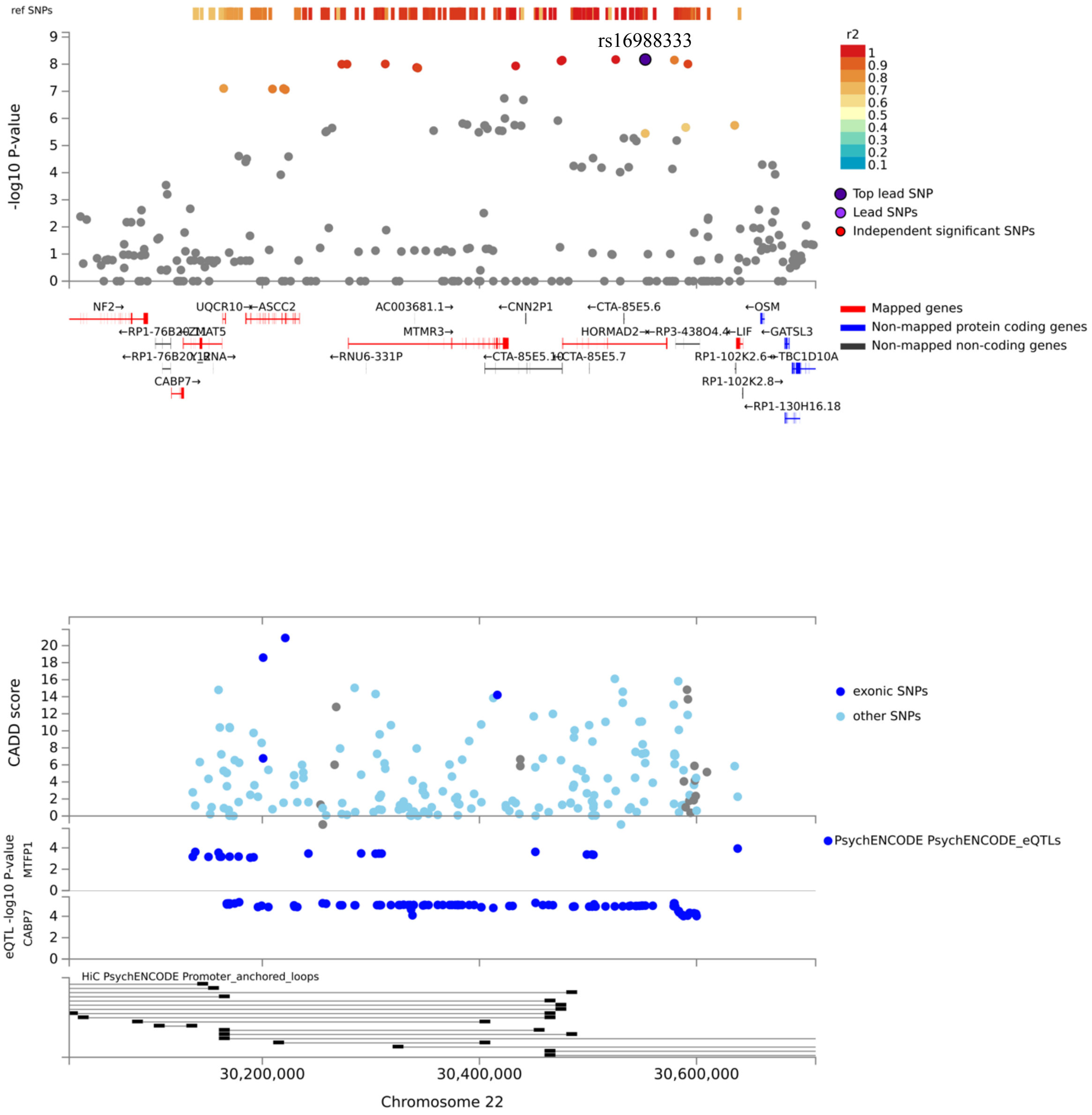

Supplement: S5 Fig — Each plot illustrates the genes mapping the locus, CADD score of the top significant genes, eQTL and chromatin interaction information estimated using PsychENCODE database. (PDF) [file pgen.1010977.s008.pdf]
