# Supplementary material for: Genome wide association joint analysis reveals 99 risk loci for pain susceptibility and pleiotropic relationships with psychiatric, metabolic, and immunological traits
Source: PLoS Genet. 2023 Oct 16;19(10):e1010977. doi: 10.1371/journal.pgen.1010977 (PMC10602383; doi:10.1371/journal.pgen.1010977)

**S7_Figure. Sex-specific joint GWAS analysis of pain-related traits.**

**A.** Manhattan plot of the meta-analysis performed in women only samples.

**B.** Manhattan plot of the meta-analysis performed in men only samples.

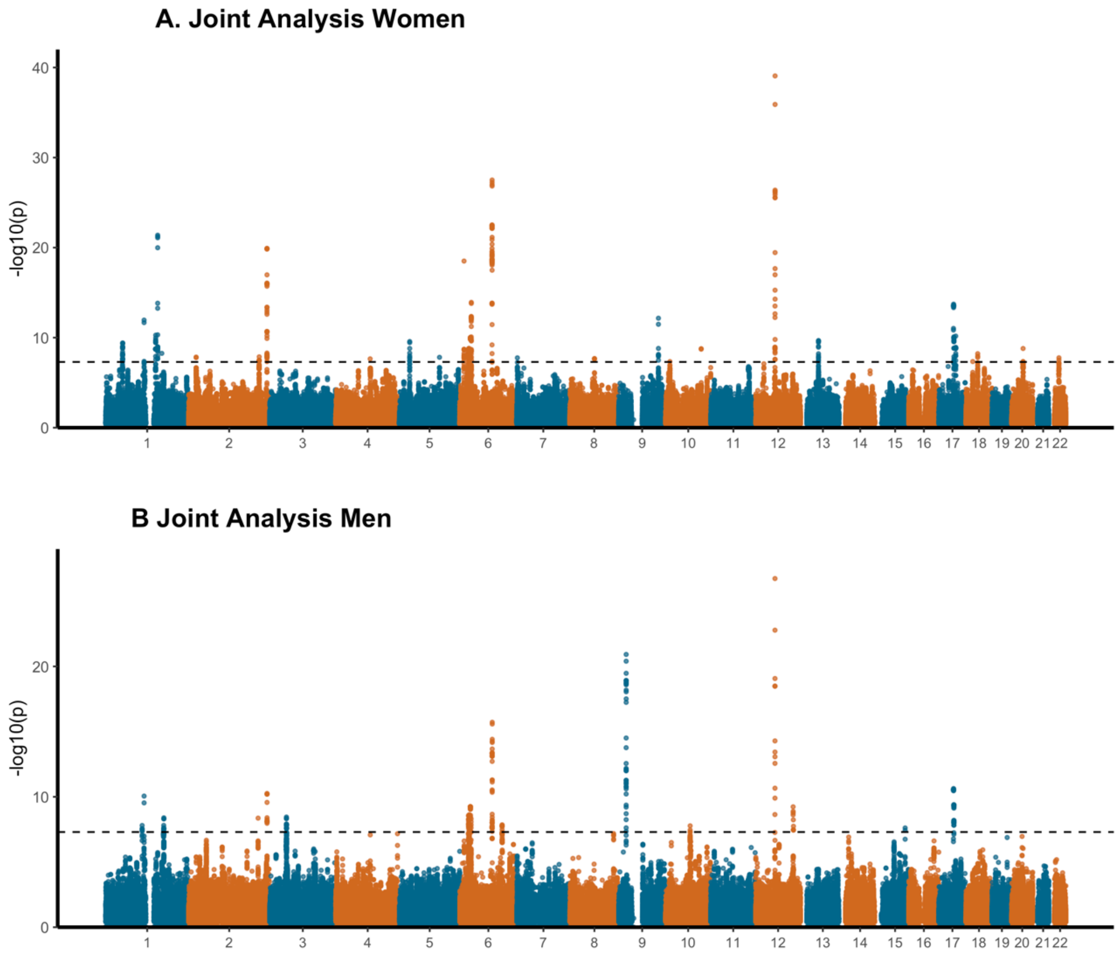

Supplement: S7 Fig — (PDF) [file pgen.1010977.s010.pdf]
