# Supplementary material for: Genome wide association joint analysis reveals 99 risk loci for pain susceptibility and pleiotropic relationships with psychiatric, metabolic, and immunological traits
Source: PLoS Genet. 2023 Oct 16;19(10):e1010977. doi: 10.1371/journal.pgen.1010977 (PMC10602383; doi:10.1371/journal.pgen.1010977)
Supplement: S9 Fig — (PDF) [file pgen.1010977.s012.pdf]

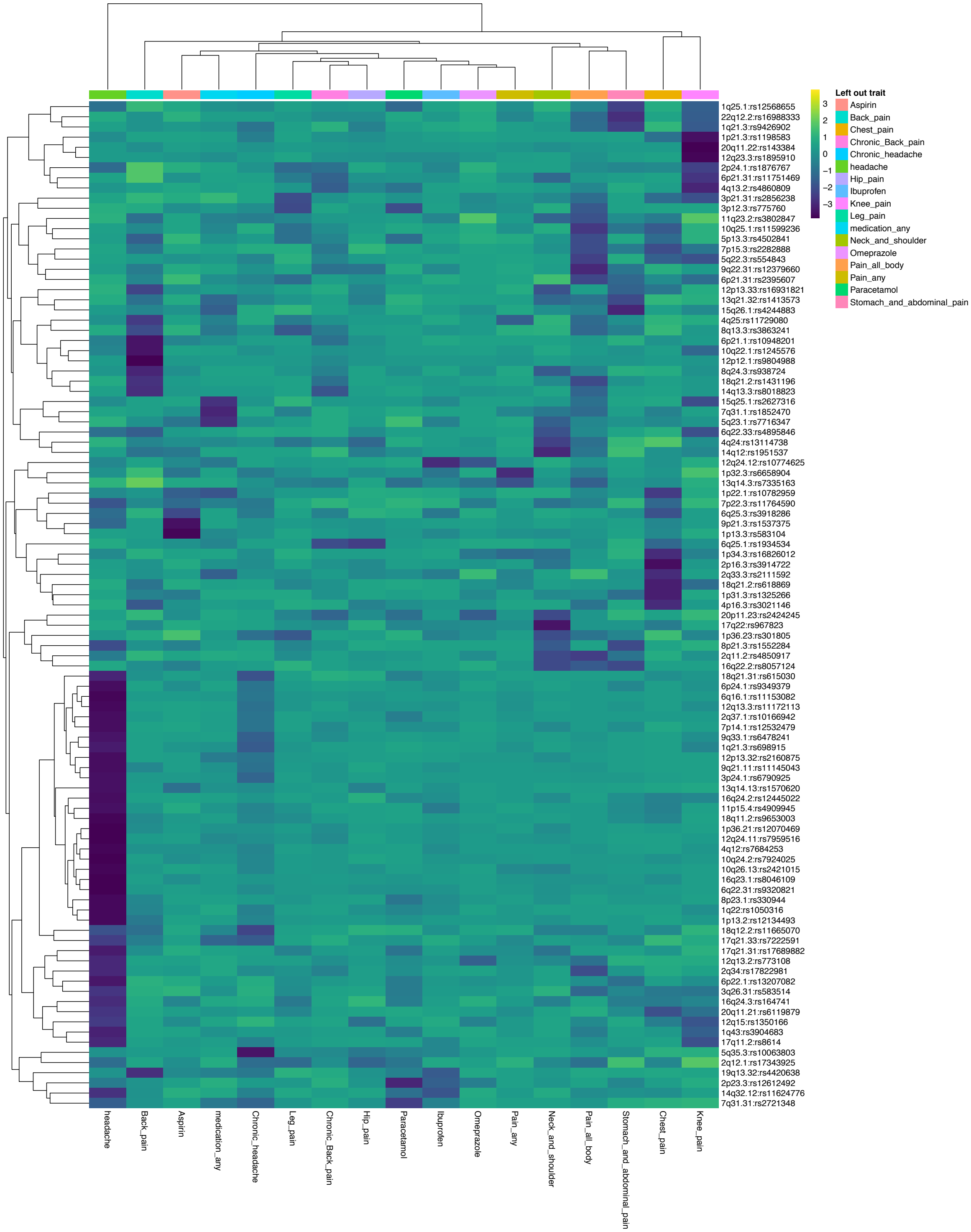


## S9_Figure. Sensitivity analysis heatmap.

The matrix is represented by the difference in significance (-log10P-value) between the value obtained in the meta-analysis run with the correspondent left out trait (columns) and the higher significance value at that locus. All 99 are listed in the rows.
