# Supplementary material for: Genome wide association joint analysis reveals 99 risk loci for pain susceptibility and pleiotropic relationships with psychiatric, metabolic, and immunological traits
Source: PLoS Genet. 2023 Oct 16;19(10):e1010977. doi: 10.1371/journal.pgen.1010977 (PMC10602383; doi:10.1371/journal.pgen.1010977)

S10_Figure. Venn diagram showing the overlap of genes selected by position, eQTLs and chromatin interaction gene mapping methods

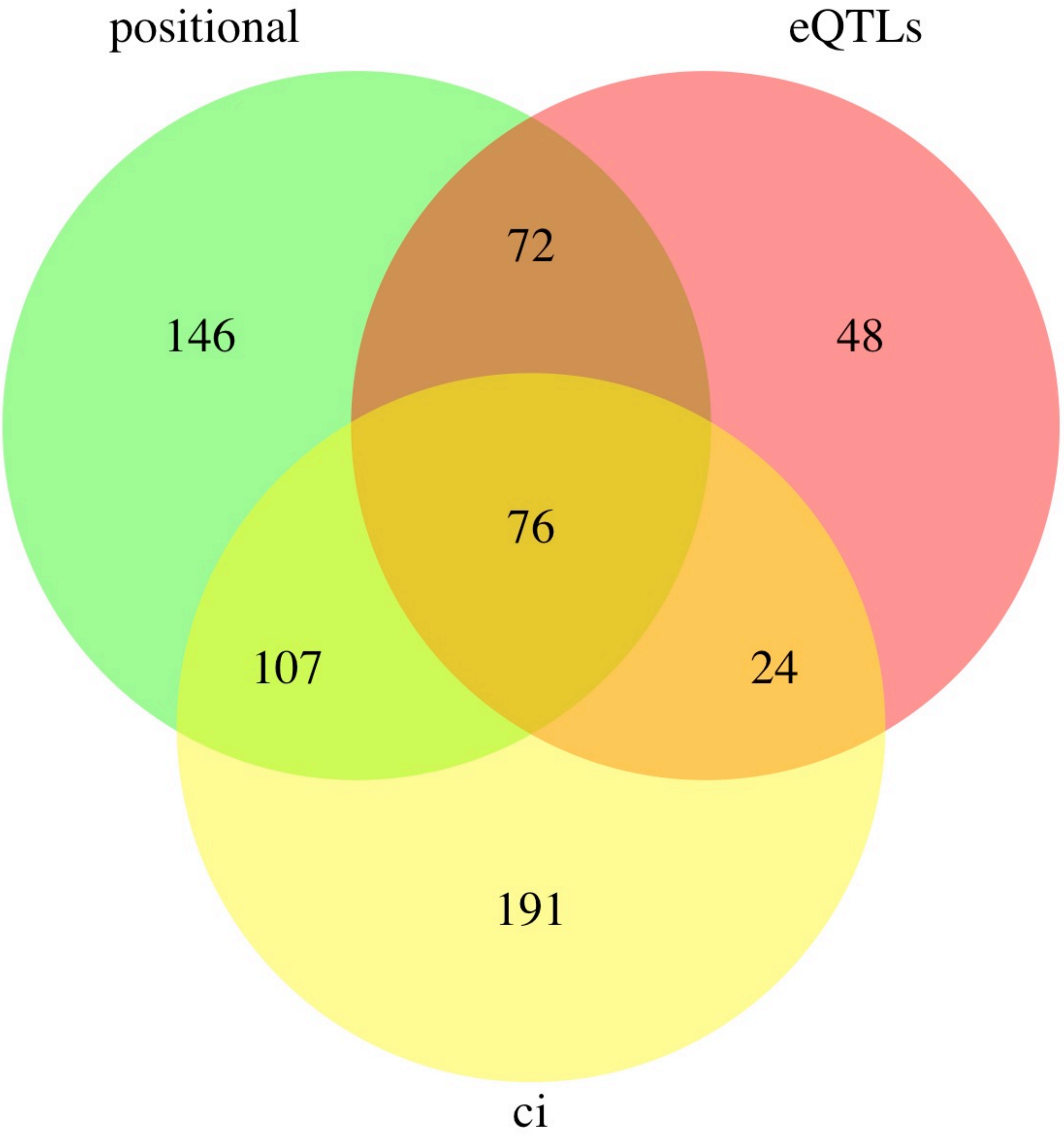

Supplement: S10 Fig — (PDF) [file pgen.1010977.s013.pdf]
