# Supplementary material for: Genome wide association joint analysis reveals 99 risk loci for pain susceptibility and pleiotropic relationships with psychiatric, metabolic, and immunological traits
Source: PLoS Genet. 2023 Oct 16;19(10):e1010977. doi: 10.1371/journal.pgen.1010977 (PMC10602383; doi:10.1371/journal.pgen.1010977)
Supplement: S11 Fig — (PDF) [file pgen.1010977.s014.pdf]

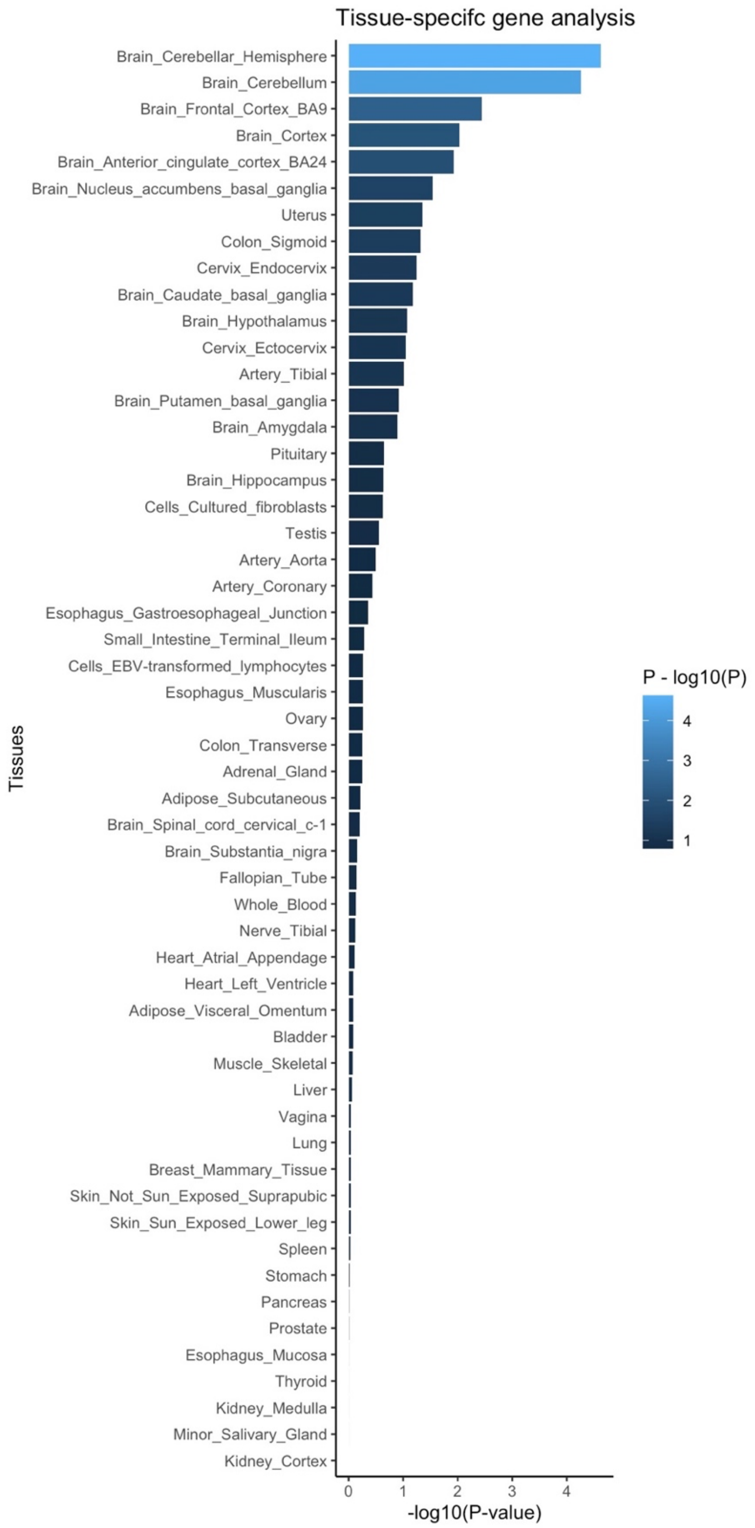

S11 Figure. Enrichment of pain risk loci for tissue-specific gene expression. X axis indicates the significance of the test and Y axis indicates tissues analyzed. After adjusting the P-value for multiple testing only the cerebellum was significantly enriched.
