# Supplementary material for: Genome wide association joint analysis reveals 99 risk loci for pain susceptibility and pleiotropic relationships with psychiatric, metabolic, and immunological traits
Source: PLoS Genet. 2023 Oct 16;19(10):e1010977. doi: 10.1371/journal.pgen.1010977 (PMC10602383; doi:10.1371/journal.pgen.1010977)
Supplement: S13 Fig — (PDF) [file pgen.1010977.s016.pdf]

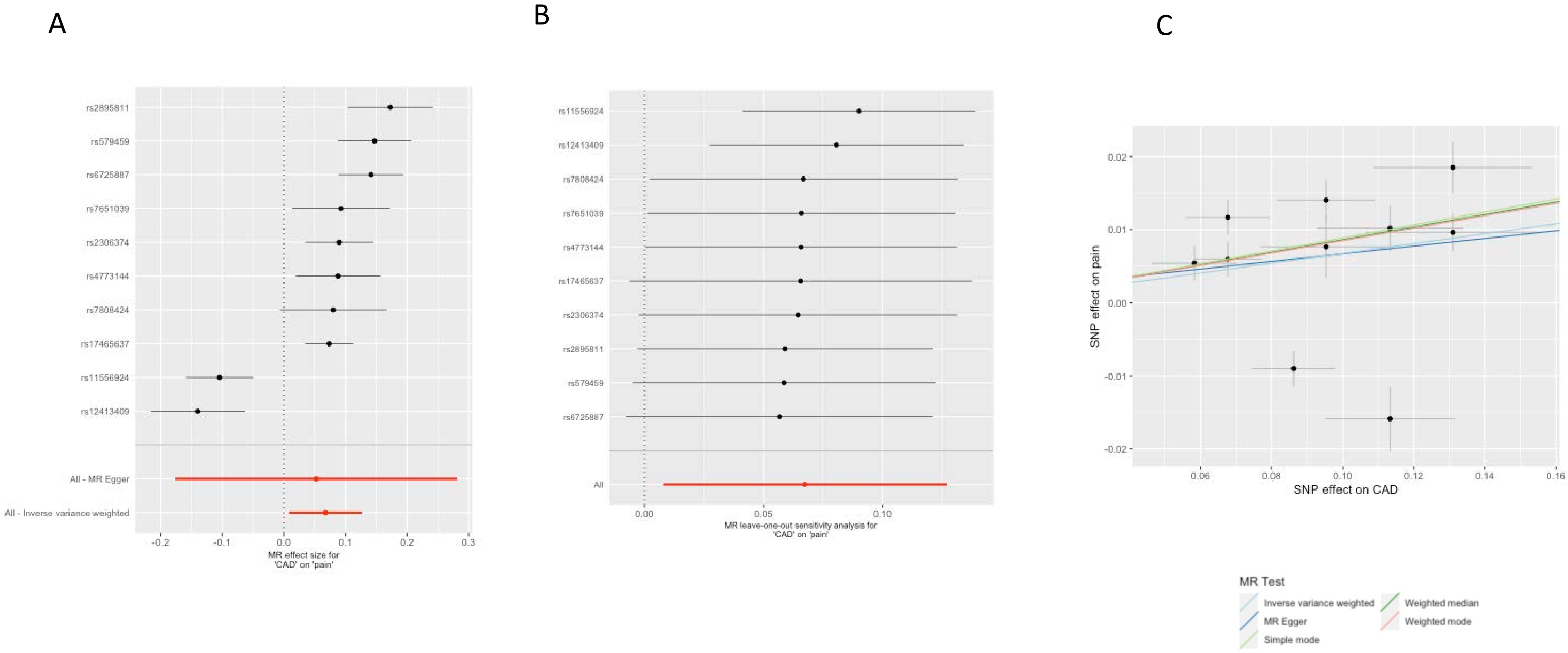

S13 Figure. Mendelian randomization coronary heart disease exposure pain outcome

A. Forest plot of single SNP MR, **B**. Leave-one-out sensitivity analysis, **C**. Comparison of results using different MR methods
